# Theoretical properties of distance distributions and novel metrics for nearest-neighbor feature selection

**DOI:** 10.1371/journal.pone.0246761

**Published:** 2021-02-08

**Authors:** Bryan A. Dawkins, Trang T. Le, Brett A. McKinney

**Affiliations:** 1 Genes and Human Disease, Oklahoma Medical Research Foundation, Oklahoma City, Oklahoma, United States of America; 2 Department of Biostatistics, Epidemiology and Informatics, University of Pennsylvania, Philadelphia, PA, United States of America; 3 Department of Mathematics, University of Tulsa, Tulsa, OK, United States of America; 4 Tandy School of Computer Science, University of Tulsa, Tulsa, OK, United States of America; Roswell Park Cancer Institute, UNITED STATES

## Abstract

The performance of nearest-neighbor feature selection and prediction methods depends on the metric for computing neighborhoods and the distribution properties of the underlying data. Recent work to improve nearest-neighbor feature selection algorithms has focused on new neighborhood estimation methods and distance metrics. However, little attention has been given to the distributional properties of pairwise distances as a function of the metric or data type. Thus, we derive general analytical expressions for the mean and variance of pairwise distances for *L*_*q*_ metrics for normal and uniform random data with *p* attributes and *m* instances. The distribution moment formulas and detailed derivations provide a resource for understanding the distance properties for metrics and data types commonly used with nearest-neighbor methods, and the derivations provide the starting point for the following novel results. We use extreme value theory to derive the mean and variance for metrics that are normalized by the range of each attribute (difference of max and min). We derive analytical formulas for a new metric for genetic variants, which are categorical variables that occur in genome-wide association studies (GWAS). The genetic distance distributions account for minor allele frequency and the transition/transversion ratio. We introduce a new metric for resting-state functional MRI data (rs-fMRI) and derive its distance distribution properties. This metric is applicable to correlation-based predictors derived from time-series data. The analytical means and variances are in strong agreement with simulation results. We also use simulations to explore the sensitivity of the expected means and variances in the presence of correlation and interactions in the data. These analytical results and new metrics can be used to inform the optimization of nearest neighbor methods for a broad range of studies, including gene expression, GWAS, and fMRI data.

## 1 Introduction

Statistical models can deviate from expected behavior depending on whether certain properties of the underlying data are satisfied, such as being normally distributed. The expected behavior of nearest neighbor models is further influenced by the choice of metric, such as Euclidean or Manhattan. For random normal data (N(0,1)), for example, the variance of the pairwise distances of a Manhattan metric is proportional to the number of attributes (*p*) whereas the variance is constant for a Euclidean metric. Relief methods [1–3] and nearest-neighbor projected-distance regression (NDPR) [4] use nearest neighbors to compute attribute importance scores for feature selection and often use adaptive neighborhoods that rely on the mean and variance of the distance distribution. The ability of this class of methods to identify association effects, like main effects or interaction effects, depends on parameters such as neighborhood radii or number of neighbors k [5, 6]. Thus, knowing the expected pairwise distance values for a given metric and data distribution may improve the performance of these feature selection methods by informing the choice of neighborhood parameters.

For continuous data, the metrics most commonly used in nearest neighbor methods are *L*_*q*_ with *q* = 1 (Manhattan) or *q* = 2 (Euclidean). For data from standard normal (N(0,1)) or standard uniform (U(0,1)) distributions, the asymptotic behavior of the *L*_*q*_ metrics is known. The mathematical formalism used to derive these known asymptotic results, however, are not readily available in the literature and the details are needed for the novel extreme value results to be derived in the current study. Thus, we first provide detailed derivations of generalized expressions parameterized by metric *q*, attributes *p*, and samples *m*. We then extend the derivations to *L*_*q*_ metrics normalized by the range of the attributes using Extreme Value Theory (EVT). These range (max-min) normalized metrics are often used in Relief-based algorithms [3], but the current study is the first to characterize the metric’s asymptotic distributions.

In addition to the novel moment estimates using extreme value theory, we also derive novel asymptotic results for metrics we recently developed for genome-wide association study (GWAS) data [7]. Various metrics have been developed for feature selection and for computing similarity between individuals based on shared genetic variation in GWAS data. We build on the mathematical formalism for continuous data to derive the asymptotic properties of various categorical (genotypic) data metrics for GWAS. We derive asymptotic formulas for the mean and variance for three recently introduced GWAS metrics [7]. These metrics were developed for Relief-based feature selection to account for binary genotype differences (two levels), allelic differences (three levels), and transition/transversion differences (five levels). The mean and variance expressions we derive for these multi-level categorical data types are parameterized by the minor allele frequency and the transition/transversion ratio.

We also introduce a novel metric for correlation data computed from time series, which is motivated by the application of resting-state functional MRI (rs-fMRI) data. We further derive asymptotic estimates for the mean and variance of distance distributions for this new metric. Unlike structural MRI (magnetic resonance imaging) of the brain, which produces a high resolution static image, rs-fMRI produces time-series brain activity. The correlation of this activity between pairs of brain Regions of Interest (ROIs) can be computed from the time series and the pairs used as attributes for machine learning and feature selection [8–11]. An ROI is composed of many smaller brain volumes known as voxels, which may be used as the spatial units, but typically ROIs are used that correspond to larger collections of voxels with known function for emotion or cognition.

For a given subject in an rs-fMRI study, a correlation matrix is computed between ROIs from the ROI time series, resulting in an overall dataset composed of ROI-ROI pairwise correlations for each of the *m* subjects. Nearest-neighbor based feature selection was applied to rs-fMRI with the private evaporative cooling method [12], where the predictors were pairwise correlations between ROIs. The use of pairwise correlation predictors is a common practice because of convenience for detection of differential connectivity between brain regions that may be of biological importance [13]. However, one may be interested in the importance of attributes at the individual ROI level. The new metric in the current study may be used in NPDR [4] feature selection or other machine learning methods for rs-fMRI correlation matrices to provide attribute importance at the level of individual ROIs. This metric is applicable to general time-series derived correlation data.

To summarize the contributions of this study, we provide multiple resources and novel results. We provide a summary of the asymptotic means and variances of pairwise distances for commonly used metrics and data types. In addition, we provide the mathematical details for deriving these quantities. We derive novel analytical results for range-normalized metrics using extreme value theory. We derive novel analytical results for new metrics for GWAS data. Most asymptotic analysis is for continuous data, but GWAS data is categorical, which requires slightly different approaches. We introduce a novel metric for correlation data derived from rs-fMRI time series, and we derive the metric’s analytical means and variances. We test the accuracy of analytical formulas for means and variances under various simulated conditions, including correlation.

In Section 2, we introduce preliminary notation and apply the Central Limit Theorem (CLT) and the Delta Method to derive asymptotics for pairwise distances. In Section 3, we present general derivations for continuously distributed data sets with *m* instances and *p* attributes. Using our more general results, we then consider the special cases of standard normal (N(0,1)) and standard uniform (U(0,1)) data distributions, for which we derive analytical expressions parameterized by metric *q*, number of attributes *p*, and number of instances *m*. In Section 4 we use Extreme Value Theory (EVT) to derive attribute range-normalized (max-min) versions of *L*_*q*_ metrics. In Section 5, we extend the derivations to categorical data with a binomial distribution for GWAS data with multiple metric types. In Section 6, we present a new time series correlation-based distance metric, with a particular emphasis on rs-fMRI data, and we derive the corresponding asymptotic distance distribution results. In Section 8, we demonstrate the effect of correlation in the attribute space on distance distributional properties. In Section 9, we demonstrate the effect of using distance distribution information on nearest-neighbor feature selection.

## 2 Limit distribution for *L*_*q*_ on null data

For continuously distributed data, nearest-neighbor feature selection algorithms most commonly define distance between instances (i,j∈I,|I|=m) in a data set *X*^*m*×*p*^ of *m* instances (or samples) and *p* attributes (or features) as the following transformation of the sum that is indexed over all attributes (a∈A, |A|=p)

$$\begin{array}{c} D_{ij}^{\left(q\right)}={\left(\sum_{a\in A}{\left|{\text{d}}_{ij}\left(a\right)\right|}^{q}\right)}^{1/q}, \end{array}$$ (1)

which is typically Manhattan (*q* = 1) in Relief-based methods and sometimes Euclidean (*q* = 2). We use the terms “feature” and “attribute” interchangeably for the remainder of this work. The metric d_*ij*_(*a*), referred to as “diff” in the context of Relief, can be viewed as the one-dimensional projection of the distance $D_{ij}^{\left(q\right)}$ onto a single attribute dimension a∈A. The function d_*ij*_(*a*) is chosen in accordance with the type of attribute (e.g., continuous or discrete). For continuous data, the projection d_*ij*_(*a*) with respect to instances i,j∈I and a fixed attribute a∈A is often defined as

$$\begin{array}{c} \begin{array}{cc} {\text{d}}_{ij}\left(a\right) & =\text{diff}(a,(i,j))\\ & =|{\hat{X}}_{ia}-{\hat{X}}_{ja}|, \end{array} \end{array}$$ (2)

where $\hat{X}$ represents the standardized data matrix *X*. Our more concise d_*ij*(*a*)_ notation is convenient for mathematical statistics than the diff(*a*, (*i*, *j*)) notation that is standard in Relief-based algorithms. NPDR does not require the division by attribute range (max(*a*) − min(*a*)) as in the case of Relief-based algorithms to constrain scores to the interval from −1 to 1, where $\max\left(a\right)=\max_{k\in I}\left\{X_{ka}\right\}$ and $\min\left(a\right)=\min_{k\in I}\left\{X_{ka}\right\}$. The diff metric d_*ij*(*a*)_ is just the magnitude difference between instance i,j∈I data values with respect to a single attribute a∈A. This one-dimensional projection can take on a multitude of formulations depending on the data distribution and various experimental characteristics.

### 2.1 Nearest-neighbor projected-distance regression

Like other nearest-neighbor feature selection algorithms, the performance of NPDR depends on appropriate choice of neighborhood optimization criteria. The size of neighborhoods must be chosen appropriately for optimal detection of important statistical effects. It has been shown using simulations that neighborhood size should be as large as possible to optimally detect main effects, whereas smaller neighborhoods are necessary to detecting interactions [6]. NPDR allows for any neighborhood algorithm to be used, such as fixed or adaptive *k*, and fixed or adaptive radius. Especially in the case of radius methods, one needs some sense of central tendency with respect to pairwise distances between a given target instance and its neighbors. Similar to the radius problem, for fixed-*k* neighborhoods we need to choose *k* so that the average distance within neighborhoods is not too large or too small with respect to the empirical average pairwise distance between pairs of instances. In order for the appropriate choice of neighborhood size to be made, we need to know the central tendency and scale of the distance distribution generated on our data.

Although we do not use NPDR in the current study, it is an important motivation for derivations herein, so we briefly describe how NPDR computes importance scores for classification problems. In the case of dichotomous outcomes, NPDR estimates regression coefficients of the following model

$$\begin{array}{c} \text{logit}\left(p_{ij}^{\text{miss}}\right)=\beta_{0}+\beta_{a}{\text{d}}_{ij}\left(a\right)+\epsilon_{ij}, \end{array}$$ (3)

where $p_{ij}^{\text{miss}}$ is the probability of instances i,j∈I being in different classes, *β*_*a*_ indicates the relative importance of attribute a∈A for predicting the binary outcome, and d_*ij*_(*a*) is the attribute diff (Eq 2). The outcome of NPDR, modeled by $p_{ij}^{\text{miss}}$, is the diff computed as a function of instance i,j∈I class labels, which is given by the following

$$\begin{array}{c} {\text{d}}_{ij}^{\text{miss}}\left(\vec{y}\right)=\{\begin{array}{c} 0,\;y_{i}=y_{j}\\ 1,\;\text{else}, \end{array} \end{array}$$ (4)

where $\vec{y}$ is the binary response (or outcome). The purpose of NPDR is ultimately testing the one-sided hypotheses given by

$$\begin{array}{c} \begin{array}{cc} H_{0} & :\beta_{a}\leq 0\\ H_{1} & :\beta_{a}>0, \end{array} \end{array}$$ (5)

where rejecting the null hypothesis (*H*_0_) implies that there is significant evidence to conclude that attribute a∈A is important for classification.

All derivations in the following sections are applicable to nearest-neighbor distance-based methods in general, which includes not only NPDR, but also Relief-based algorithms. Each of these methods uses a distance metric (Eq 1) to compute neighbors for each instance i∈I. Therefore, our derivations of asymptotic distance distributions are applicable to all methods that compute neighbors in order to weight features. The predictors used by NPDR (Eq 3), however, are the one-dimensional projected distances between two instances i,j∈I (Eq 2). Hence, all asymptotic estimates we derive for diff metrics (Eq 2) are particularly relevant to NPDR. Since the standard distance metric (Eq 1) is a function of the one-dimensional projection (Eq 2), asymptotic estimates derived for this projection (Eq 2) are implicitly relevant to older nearest-neighbor distance-based methods like Relief-based algorithms.

We proceed in the following section by applying the Classical Central Limit Theorem and the Delta Method to derive the limit distribution of pairwise distances on any data distribution that is induced by the standard distance metric (Eq 1). We assume independent samples in order to derive closed-form moment estimates and to show that distances are asymptotically normal. In real data, it is obviously not the case that samples or attributes will be independent; however, the normality assumption for distances is approximately satisfied in a large number of cases. For example, it has been shown using 100 real gene expression data sets from microarrays, that approximately 80% of the data sets are either approximately normal or log-normal in distribution [14]. We generated Manhattan distances (Eq 1, *q* = 1) on 99 of the same 100 gene expression data sets after applying a pre-processing pipeline. We excluded GSE67376 because this data included only a single sample. Before generating distance matrices, we transformed the data using quantile normalization, removed genes with high coefficient of variation, and standardized samples to have zero mean and unit variance.

We computed densities for each distance matrix, as well as quantile-quantile plots to visually assess normality (S26-S124 Figs in S1 File). The estimated densities and quantile-quantile plots indicate that most of the gene expression data sets yield approximately normally distributed distances between instances. Another example involves real resting-state fMRI data from a study of mood and anxiety disorders [15], where the data was generated both from a spherical ROI parcellation [16] and a graph theoretic parcellation [17]. The data consists of correlation matrices between ROI time series with respect to each parcellation and each subject. Each subject correlation matrix, excluding the diagonal entries, was vectorized and combined into a single matrix containing all subject ROI correlations. We then applied a Fisher r-to-z transformation and standardized samples to be zero mean and unit variance. The output of this process was two data matrices corresponding to each parcellation, respectively. Analogous to the gene expression microarray data, we computed Manhattan distance matrices for each of the two resting-state fMRI data sets. We generated quantile-quantile and density plots for each matrix (S125 and S126 Figs in S1 File). Both sets of pairwise distances were approximately normal.

### 2.2 Asymptotic normality of pairwise distances

Suppose that $X_{ia},X_{ja}\overset{iid}{∼}F_{X}(\mu_{X},\sigma_{X}^{2})$ for two fixed and distinct instances i,j∈I and a fixed attribute a∈A. $F_{X}$ represents any data distribution with mean *μ*_*X*_ and variance $\sigma_{X}^{2}$.

It is clear that |*X*_*ia*_ − *X*_*ja*_|^*q*^ = |d_*ij*_(*a*)|^*q*^ is another random variable, so we let $Z_{a}^{q}∼F_{Z_{a}^{q}}(\mu_{z_{a}^{q}},\sigma_{z_{a}^{q}}^{2})$ be the random variable such that

$$\begin{array}{c} Z_{a}^{q}=|{\text{d}}_{ij}{\left(a\right)|}^{q}={\left|X_{ia}-X_{ja}\right|}^{q},\;a\in A. \end{array}$$ (6)

Furthermore, the collection $\left\{Z_{a}^{q}|a\in A\right\}$ is a random sample of size *p* of mutually independent random variables. Hence, the sum of $Z_{a}^{q}$ over all a∈A is asymptotically normal by the Classical Central Limit Theorem (CCLT). More explicitly, this implies that

$$\begin{array}{c} {\left(D_{ij}^{\left(q\right)}\right)}^{q}=\sum_{a\in A}|{\text{d}}_{ij}{\left(a\right)|}^{q}=\sum_{a\in A}{\left|X_{ia}-X_{ja}\right|}^{q}=\sum_{a\in A}Z_{a}^{q}\dot{∼}N(\mu_{z_{a}^{q}}p,\sigma_{z_{a}^{q}}^{2}p). \end{array}$$ (7)

Consider the smooth function *g*(*z*) = *z*^1/*q*^, which is continuously differentiable for *z* > 0. Assuming that $\mu_{z_{a}^{q}}>0$, the Delta Method [18] can be applied to show that

$$\begin{array}{c} \begin{array}{cc} g({\left(D_{ij}^{\left(q\right)}\right)}^{q}) & =g(\sum_{a\in A}^{p}Z_{a}^{q})\\ & ={\left(\sum_{a\in A}{\left|X_{ia}-X_{ja}\right|}^{q}\right)}^{1/q}\\ & =D_{ij}^{\left(q\right)}\dot{∼}N(g(\mu_{z^{q}}p),{\left[g^{\prime }(\mu_{z_{a}^{q}}p)\right]}^{2}\sigma_{z_{a}^{q}}^{2}p)\\ \Rightarrow & D_{ij}^{\left(q\right)}\dot{∼}N({\left(\mu_{z_{a}^{q}}p\right)}^{1/q},\frac{\sigma_{z_{a}^{q}}^{2}p}{q^{2}{\left(\mu_{z_{a}^{q}}p\right)}^{2(1-\frac{1}{q})}}). \end{array} \end{array}$$ (8)

Therefore, the distance between two fixed, distinct instances *i* and *j* (Eq 1) is asymptotically normal. In particular, when *q* = 2, the distribution of $D_{ij}^{\left(2\right)}$ asymptotically approaches $N(\sqrt{\mu_{z_{a}^{2}}p},\frac{\sigma_{z_{a}^{2}}^{2}}{4\mu_{z_{a}^{2}}})$. A unique characteristic inherent to the *q* = 2 case is the fact that we get not only an asymptotic estimate for the average second raw moment of the *L*_*q*_ metric (Eq 8, *q* = 2), but also the variance of the second raw moment. This leads to the following higher order estimate of the sample mean in the case of *q* = 2

$$\begin{array}{c} \begin{array}{cc} \text{E}(D_{ij}^{\left(2\right)}) & =\sqrt{\text{E}[{\left(D_{ij}^{\left(2\right)}\right)}^{2}]-\text{Var}(D_{ij}^{\left(2\right)})}\\ & =\sqrt{\mu_{z_{a}^{2}}p-\frac{\sigma_{z_{a}^{2}}^{2}}{4\mu_{z_{a}^{2}}}}. \end{array} \end{array}$$ (9)

**Fig 1:**
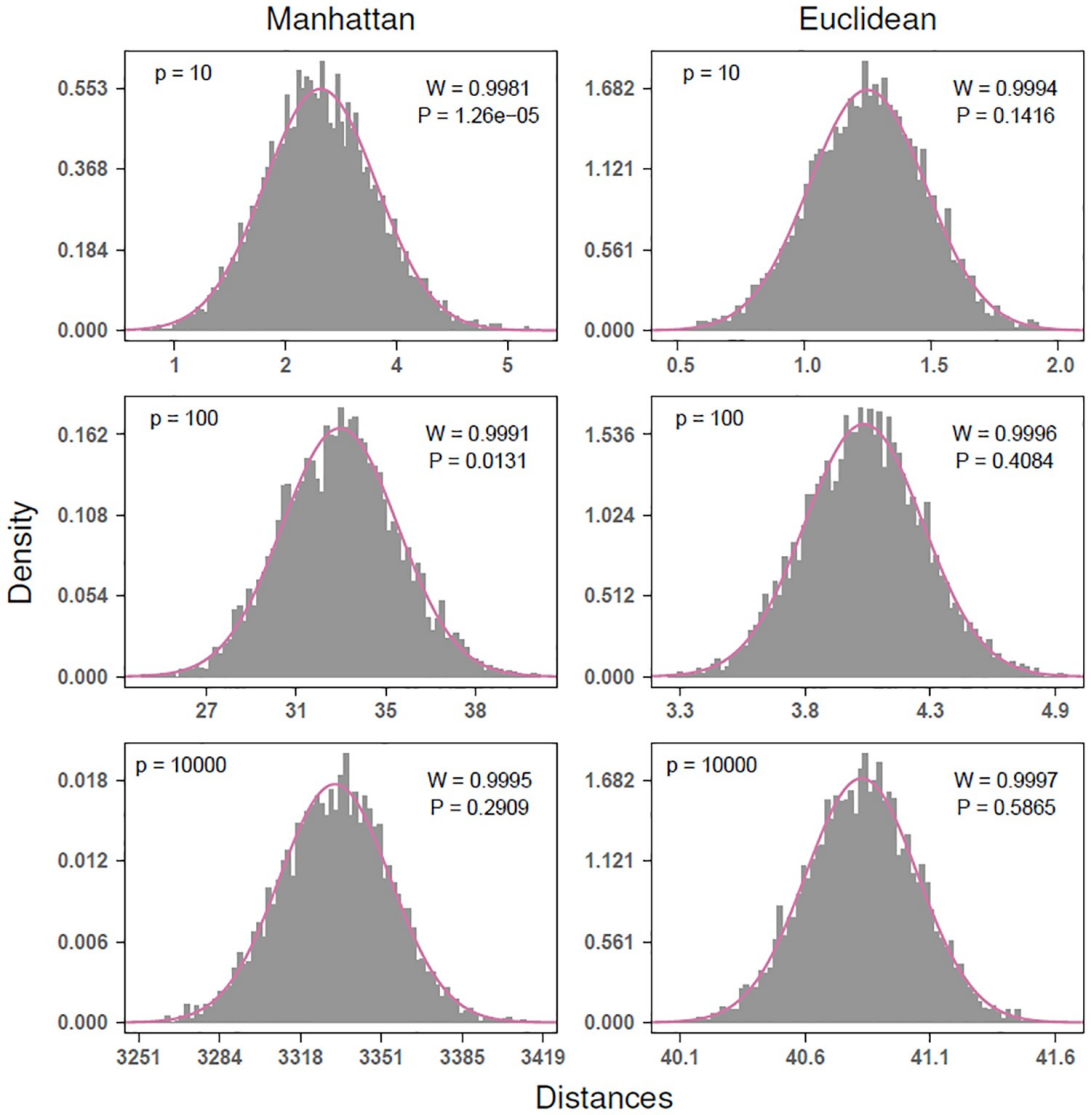
Convergence to Gaussian for Manhattan and Euclidean distances for simulated standard uniform data with *m* = 100 instances and *p* = 10, 100, and 10000 attributes. Convergence to Gaussian occurs rapidly with increasing *p*, and Gaussian is a good approximation for *p* as low as 10 attributes. The number of attributes in bioinformatics data is typically much larger, at least on the order of 10^3^. The Euclidean metric has stronger convergence to normal than Manhattan. P values from Shapiro-Wilk test, where the null hypothesis is a Gaussian distribution.

The distribution of pairwise distances convergences quickly to a Gaussian for Euclidean (*q* = 2) and Manhattan (*q* = 1) metrics as the number of attributes *p* increases (Fig 1). We compute the distance between all pairs of instances in simulated datasets of uniformly distributed random data. We simulate data with fixed *m* = 100 instances, and, by varying the number of attributes (*p* = 10, 100, 10000), we observe rapid convergence to Gaussian. For *p* as low as 10 attributes, Gaussian is a good approximation. The number of attributes in bioinformatics data is typically quite large, at least on the order of 10^3^. The Shapiro-Wilk statistic approaches 1 more rapidly for the Euclidean than Manhattan, which may indicate more rapid convergence in the case of Euclidean. This may be partly due to Euclidean’s use of the square root, which is a common transformation of data in statistics.

To show asymptotic normality of distances, we did not specify whether the data distribution $F_{X}$ was discrete or continuous. This is because asymptotic normality is a general phenomenon in high attribute dimension *p* for any data distribution $F_{X}$ satisfying the assumptions we have made. Therefore, the simulated distances we have shown (Fig 1) have an analogous representation for discrete data, as well as all other continuous data distributions. In addition to showing Gaussian convergence for Manhattan and Euclidean distances on standard uniform data, we show a similar result for standard normal data (S2 Fig in S1 File).

For distance based learning methods, all pairwise distances are used to determine relative importances for attributes. The collection of all distances above the diagonal in an *m* × *m* distance matrix does not satisfy the independence assumption used in the previous derivations. This is because of the redundancy that is inherent to the distance matrix calculation. However, this collection is still asymptotically normal with mean and variance approximately equal to those we have previously given (Eq 8). In the next section, we assume actual data distributions in order to define more specific general formulas for standard *L*_*q*_ and max-min normalized *L*_*q*_ metrics. We also derive asymptotic moments for a new discrete metric in GWAS data and a new metric for time series correlation-based data, such as, resting-state fMRI.

## 3 *L*_*q*_ metric moments for continuous data distributions

In this section, we derive general formulas for asymptotic means and variances of the *L*_*q*_ distance (Eq 1) for standard normal and standard uniform data. With our general formulas for continuous data, we compute moments associated with Manhattan (*L*_1_) and Euclidean (*L*_2_) metrics. In the subsequent section, we combine the asymptotic analysis of this section with extreme value theory (EVT) to derive mean and variance formulas for the more complicated max-min normalized version of the *L*_*q*_ distance, where the magnitude difference (Eq 2) is divided by the range of each attribute *a*.

### 3.1 Distribution of |d_*ij*_(*a*)|^*q*^ = |*X*_*ia*_ − *X*_*ja*_|^*q*^

Suppose that $X_{ia},X_{ja}\overset{iid}{∼}F_{X}\left(\mu_{x},\sigma_{x}^{2}\right)$ and define $Z_{a}^{q}=|{\text{d}}_{ij}{\left(a\right)|}^{q}={\left|X_{ia}-X_{ja}\right|}^{q}$, where a∈A and |A|=p. In order to find the distribution of $Z_{a}^{q}$, we will use the following theorem given in [19].

**Theorem 3.1** *Let f(x) be the value of the probability density of the continuous random variable X at x. If the function given by y = u(x) is differentiable and either increasing or decreasing for all values within the range of X for which f(x)≠0, then, for these values of x, the equation y = u(x) can be uniquely solved for x to give x = w(y), and for the corresponding values of y the probability density of Y = u(X) is given by*

$$\begin{array}{c} g\left(y\right)=f\left[w\left(y\right)\right]\cdot |w^{\prime }\left(y\right)|\;\;\text{provided}\;u^{\prime }\left(x\right)\neq 0 \end{array}$$

*Elsewhere, g*(*y*) = 0.

We have the following cases that result from solving for *X*_*ja*_ in the equation given by $Z_{a}^{q}={\left|X_{ia}-X_{ja}\right|}^{q}$:

1. Suppose that $X_{ja}=X_{ia}-{\left(Z_{a}^{q}\right)}^{1/q}$. Based on the iid assumption for *X*_*ia*_ and *X*_*ja*_, it follows from Thm. 3.1 that the joint density function *g*^(1)^ of *X*_*ia*_ and $Z_{a}^{q}$ is given by
  $$\begin{array}{c} \begin{array}{cc} g^{\left(1\right)}\left(x_{ia},z_{a}\right) & =f_{X}\left(x_{ia},x_{ja}\right)|\frac{\partial x_{ja}}{\partial z_{a}}|\\ & =f_{X}\left(x_{ia}\right)f_{X}\left(x_{ja}\right)|\frac{-1}{q}{\left(z_{a}^{q}\right)}^{\frac{1}{q}-1}|\\ & =\frac{1}{q{\left(z_{a}^{q}\right)}^{1-\frac{1}{q}}}f_{X}\left(x_{ia}\right)f_{X}\left(x_{ia}-{\left(z_{a}^{q}\right)}^{1/q}\right),\;z_{a}>0. \end{array} \end{array}$$ (10)
  The density function $f_{Z_{a}^{q}}^{\left(1\right)}$ of $Z_{a}^{q}$ is then defined as
  $$\begin{array}{c} \begin{array}{cc} f_{Z_{a}^{q}}^{\left(1\right)}\left(z_{a}^{q}\right) & =\int_{-\infty }^{\infty }g^{\left(1\right)}\left(x_{ia},z_{a}^{q}\right)\text{d}x_{ia}\\ & =\frac{1}{q{\left(z_{a}^{q}\right)}^{1-\frac{1}{q}}}\int_{-\infty }^{\infty }f_{X}\left(x_{ia}\right)f_{X}\left(x_{ia}-{\left(z_{a}^{q}\right)}^{1/q}\right)\text{d}x_{ia},\;z_{a}>0. \end{array} \end{array}$$ (11)
2. Suppose that $X_{ja}=X_{ia}+{\left(Z_{a}^{q}\right)}^{1/q}$. Based on the iid assumption for *X*_*ia*_ and *X*_*ja*_, it follows from Thm. 3.1 that the joint density function *g*^(2)^ of *X*_*ia*_ and *Z*_*a*_ is given by
  $$\begin{array}{c} \begin{array}{cc} g^{\left(2\right)}\left(x_{ia},z_{a}\right) & =f_{X}\left(x_{ia},x_{ja}\right)|\frac{\partial x_{ja}}{\partial z_{a}}|\\ & =f_{X}\left(x_{ia}\right)f_{X}\left(x_{ja}\right)|\frac{1}{q}{\left(z_{a}^{q}\right)}^{\frac{1}{q}-1}|\\ & =\frac{1}{q{\left(z_{a}^{q}\right)}^{1-\frac{1}{q}}}f_{X}\left(x_{ia}\right)f_{X}\left(x_{ia}-{\left(z_{a}^{q}\right)}^{1/q}\right),\;z_{a}>0. \end{array} \end{array}$$ (12)
  The density function $f_{Z_{a}^{q}}^{\left(2\right)}$ of $Z_{a}^{q}$ is then defined as
  $$\begin{array}{c} \begin{array}{cc} f_{Z_{a}^{q}}^{\left(2\right)}\left(z_{a}^{q}\right) & =\int_{-\infty }^{\infty }g^{\left(2\right)}\left(x_{ia},z_{a}^{q}\right)\text{d}x_{ia}\\ & =\frac{1}{q{\left(z_{a}^{q}\right)}^{1-\frac{1}{q}}}\int_{-\infty }^{\infty }f_{X}\left(x_{ia}\right)f_{X}\left(x_{ia}+{\left(z_{a}^{q}\right)}^{1/q}\right)\text{d}x_{ia},\;z_{a}>0. \end{array} \end{array}$$ (13)

Let $F_{Z_{a}^{q}}$ denote the distribution function of the random variable $Z_{a}^{q}$. Furthermore, we define the events *E*^(1)^ and *E*^(2)^ as

$$\begin{array}{c} E^{\left(1\right)}=\{|X_{ia}-X_{ja}{|}^{q}\leq z_{a}^{q}:X_{ja}=X_{ia}-{\left(Z_{a}^{q}\right)}^{1/q}\} \end{array}$$ (14)

and

$$\begin{array}{c} E^{\left(2\right)}=\{|X_{ia}-X_{ja}{|}^{q}\leq z_{a}^{q}:X_{ja}=X_{ia}+{\left(Z_{a}^{q}\right)}^{1/q}\}. \end{array}$$ (15)

Then it follows from fundamental rules of probability that

$$\begin{array}{c} \begin{array}{cc} F_{Z_{a}^{q}}\left(z_{a}^{q}\right) & =\text{P}[Z_{a}^{q}\leq z_{a}^{q}]\\ & =\text{P}[|X_{ia}-X_{ja}{|}^{q}\leq z_{a}^{q}]\\ & =\text{P}[E^{\left(1\right)}\cup E^{\left(2\right)}]\\ & =\text{P}[E^{\left(1\right)}]+\text{P}[E^{\left(2\right)}]-\text{P}[E^{\left(1\right)}\cap E^{\left(2\right)}]\\ & =\text{P}[E^{\left(1\right)}]+\text{P}[E^{\left(2\right)}]\\ & =\int_{-\infty }^{z_{a}^{q}}f_{Z_{a}^{q}}^{\left(1\right)}\left(t\right)\text{d}t+\int_{-\infty }^{z_{a}^{q}}f_{Z_{a}^{q}}^{\left(2\right)}\left(t\right)\text{d}t\\ & =\int_{-\infty }^{z_{a}^{q}}\left(f_{Z_{a}^{q}}^{\left(1\right)}\left(t\right)+f_{Z_{a}^{q}}^{\left(2\right)}\left(t\right)\right)\text{d}t\\ & =\frac{1}{q{\left(z_{a}^{q}\right)}^{1-\frac{1}{q}}}\int_{-\infty }^{z_{a}^{q}}\left(\int_{-\infty }^{\infty }f_{X}\left(x_{ia}\right)[f_{X}\left(x_{ia}-t\right)+f_{X}\left(x_{ia}+t\right)]\text{d}x_{ia}\right)\text{d}t,\;z_{a}>0. \end{array} \end{array}$$ (16)

It follows directly from the previous result (Eq 16) that the density function of the random variable $Z_{a}^{q}$ is given by

$$\begin{array}{c} \begin{array}{cc} f_{Z_{a}^{q}}\left(z_{a}^{q}\right) & =\frac{\partial }{\partial z_{a}^{q}}F_{Z_{a}^{q}}\left(z_{a}^{q}\right)\\ & =\frac{1}{q{\left(z_{a}^{q}\right)}^{1-\frac{1}{q}}}\int_{-\infty }^{\infty }f_{X}\left(x_{ia}\right)[f_{X}\left(x_{ia}-{\left(z_{a}^{q}\right)}^{1/q}\right)+f_{X}\left(x_{ia}+{\left(z_{a}^{q}\right)}^{1/q}\right)]\text{d}x_{ia}, \end{array} \end{array}$$ (17)

where *z*_*a*_ > 0.

Using the previous result (Eq 17), we can compute the mean and variance of the random variable $Z_{a}^{q}$ as

$$\begin{array}{c} \mu_{z_{a}^{q}}=\int_{-\infty }^{\infty }z_{a}^{q}f_{Z_{a}^{q}}\left(z_{a}^{q}\right)\text{d}z_{a}^{q} \end{array}$$ (18)

and

$$\begin{array}{c} \sigma_{z_{a}^{q}}^{2}=\int_{-\infty }^{\infty }{\left(z_{a}^{q}\right)}^{2}f_{Z_{a}^{q}}\left(z_{a}^{q}\right)\text{d}z_{a}^{q}-\mu_{z_{a}^{q}}^{2}. \end{array}$$ (19)

It follows immediately from the mean (Eq 18) and variance (Eq 19) and the Classical Central Limit Theorem (CCLT) that

$$\begin{array}{c} {\left(D_{ij}^{\left(q\right)}\right)}^{q}=\sum_{a\in A}Z_{a}^{q}=\sum_{a\in A}{\left|X_{ia}-X_{ja}\right|}^{q}\dot{∼}N\left(\mu_{z_{a}^{q}}p,\sigma_{z_{a}^{q}}^{2}p\right). \end{array}$$ (20)

Applying the convergence result we derived previously (Eq 8), the distribution of $D_{ij}^{\left(q\right)}$ is given by

$$\begin{array}{c} D_{ij}^{\left(q\right)}\dot{∼}N\left({\left(\mu_{z_{a}^{q}}p\right)}^{1/q},\frac{\sigma_{z_{a}^{q}}^{2}p}{q^{2}{\left(\mu_{z_{a}^{q}}p\right)}^{2\left(1-\frac{1}{q}\right)}}\right),\;\mu_{z_{a}^{q}}>0, \end{array}$$ (21)

where we have an improved estimate of the mean for *q* = 2 (Eq 9).

#### 3.1.1 Standard normal data

If $X_{ia},X_{ja}\overset{iid}{∼}N\left(0,1\right)$, then the marginal density functions with respect to *X* for *X*_*ia*_, $X_{ia}-{\left(Z_{a}^{q}\right)}^{1/q}$, and $X_{ia}+{\left(Z_{a}^{q}\right)}^{1/q}$ are defined as

$$\begin{array}{c} f_{X}\left(x_{ia}\right)=\frac{1}{\sqrt{2\pi }}e^{-\frac{1}{2}x_{ia}^{2}}, \end{array}$$ (22)

$$\begin{array}{c} f_{X}\left(x_{ia}-{\left(z_{a}^{q}\right)}^{1/q}\right)=\frac{1}{\sqrt{2\pi }}e^{-\frac{1}{2}{\left(x_{ia}-{\left(z_{a}^{q}\right)}^{1/q}\right)}^{2}},\;z_{a}>0,\;\text{and} \end{array}$$ (23)

$$\begin{array}{c} f_{X}\left(x_{ia}+{\left(z_{a}^{q}\right)}^{1/q}\right)=\frac{1}{\sqrt{2\pi }}e^{-\frac{1}{2}{\left(x_{ia}+{\left(z_{a}^{q}\right)}^{1/q}\right)}^{2}},\;z_{a}>0. \end{array}$$ (24)

Substituting these marginal densities (Eqs 22–24) into the general density function for $Z_{a}^{q}$ (Eq 17) and completing the square on *x*_*ia*_ in the exponents, we have

$$\begin{array}{c} \begin{array}{cc} f_{Z_{a}^{q}}\left(z_{a}^{q}\right) & =\frac{1}{2q\pi {\left(z_{a}^{q}\right)}^{1-\frac{1}{q}}}e^{-\frac{1}{4}{\left(z_{a}^{q}\right)}^{2/q}}\int_{-\infty }^{\infty }(e^{-\frac{1}{2}{\left[\sqrt{2}x_{ia}-\frac{\sqrt{2}}{2}{\left(z_{a}^{q}\right)}^{1/q}\right]}^{2}}\\ & \;+e^{-\frac{1}{2}{\left[\sqrt{2}x_{ia}+\frac{\sqrt{2}}{2}{\left(z_{a}^{q}\right)}^{1/q}\right]}^{2}})\text{d}x_{ia}\\ & =\frac{1}{2q\sqrt{\pi }{\left(z_{a}^{q}\right)}^{1-\frac{1}{q}}}e^{-\frac{1}{4}{\left(z_{a}^{q}\right)}^{2/q}}\int_{-\infty }^{\infty }\frac{1}{\sqrt{2\pi }}(e^{-\frac{1}{2}u^{2}}+e^{-\frac{1}{2}u^{2}})\text{d}u\\ & =\frac{1}{2q\sqrt{\pi }{\left(z_{a}^{q}\right)}^{1-\frac{1}{q}}}e^{-\frac{1}{4}{\left(z_{a}^{q}\right)}^{2/q}}\left(1+1\right)\\ & =\frac{1}{q\sqrt{\pi }}{\left(z_{a}^{q}\right)}^{\frac{1}{q}-1}e^{-\frac{1}{4}{\left(z_{a}^{q}\right)}^{2/q}}\\ & =\frac{\frac{2}{q}}{{\left(2^{q}\right)}^{1/q}\Gamma (\frac{\frac{1}{q}}{\frac{2}{q}})}{\left(z_{a}^{q}\right)}^{\frac{1}{q}-1}e^{-{\left(\frac{z_{a}^{q}}{2^{q}}\right)}^{2/q}}. \end{array} \end{array}$$ (25)

The density function given previously (Eq 25) is a Generalized Gamma density with parameters $b=\frac{2}{q}$, *c* = 2^*q*^, and $d=\frac{1}{q}$. This distribution has mean and variance given by

$$\begin{array}{c} \begin{array}{cc} \mu_{z_{a}^{q}} & =\frac{c\Gamma \left(\frac{d+1}{b}\right)}{\Gamma \left(\frac{d}{b}\right)}\\ & =\frac{2^{q}\Gamma \left(\frac{q+1}{2}\right)}{\sqrt{\pi }} \end{array} \end{array}$$ (26)

and

$$\begin{array}{c} \begin{array}{cc} \sigma_{z_{a}^{q}}^{2} & =c^{2}[\frac{\Gamma \left(\frac{d+2}{b}\right)}{\Gamma \left(\frac{d}{b}\right)}-{\left(\frac{\Gamma \left(\frac{d+1}{b}\right)}{\Gamma \left(\frac{d}{b}\right)}\right)}^{2}]\\ & =4^{q}[\frac{\Gamma \left(q+\frac{1}{2}\right)}{\sqrt{\pi }}-\frac{\Gamma^{2}\left(\frac{1}{2}q+\frac{1}{2}\right)}{\pi }]. \end{array} \end{array}$$ (27)

By linearity of the expected value and variance operators under the iid assumption, the mean (Eq 26) and variance (Eq 27) of the random variable $Z_{a}^{q}$ allow the *p*- dimensional mean and variance of the ${\left(D_{ij}^{\left(q\right)}\right)}^{q}$ distribution to be computed directly as

$$\begin{array}{c} \mu_{{\left(D_{ij}^{\left(q\right)}\right)}^{q}}=\text{E}[{\left(D_{ij}^{\left(q\right)}\right)}^{q}]=\text{E}\left(\sum_{a\in A}Z_{a}^{q}\right)=\sum_{a\in A}\text{E}\left(Z_{a}^{q}\right)=\sum_{a\in A}\frac{2^{q}\Gamma \left(\frac{q+1}{2}\right)}{\sqrt{\pi }}=\frac{2^{q}\Gamma \left(\frac{q+1}{2}\right)}{\sqrt{\pi }}p \end{array}$$ (28)

and

$$\begin{array}{c} \begin{array}{cc} \sigma_{{\left(D_{ij}^{\left(q\right)}\right)}^{q}}^{2}=\text{Var}[{\left(D_{ij}^{\left(q\right)}\right)}^{q}] & =\text{Var}\left(\sum_{a\in A}Z_{a}^{q}\right)\\ & =\sum_{a\in A}\text{Var}\left(Z_{a}^{q}\right)\\ & =\sum_{a\in A}4^{q}[\frac{\Gamma \left(q+\frac{1}{2}\right)}{\sqrt{\pi }}-\frac{\Gamma^{2}\left(\frac{1}{2}q+\frac{1}{2}\right)}{\pi }]\\ & =4^{q}[\frac{\Gamma \left(q+\frac{1}{2}\right)}{\sqrt{\pi }}-\frac{\Gamma^{2}\left(\frac{1}{2}q+\frac{1}{2}\right)}{\pi }]p. \end{array} \end{array}$$ (29)

Therefore, the asymptotic distribution of $D_{ij}^{\left(q\right)}$ for standard normal data is

$$\begin{array}{c} N\left({\left(2^{q}\frac{\Gamma \left(\frac{q+1}{2}\right)}{\sqrt{\pi }}p\right)}^{1/q},\frac{4^{q}p}{q^{2}{\left(\frac{2^{q}\Gamma \left(\frac{1}{2}q+\frac{1}{2}\right)}{\sqrt{\pi }}p\right)}^{2\left(1-\frac{1}{q}\right)}}[\frac{\Gamma \left(q+\frac{1}{2}\right)}{\sqrt{\pi }}-\frac{\Gamma^{2}\left(\frac{1}{2}q+\frac{1}{2}\right)}{\pi }]\right). \end{array}$$ (30)

**Fig 2:**
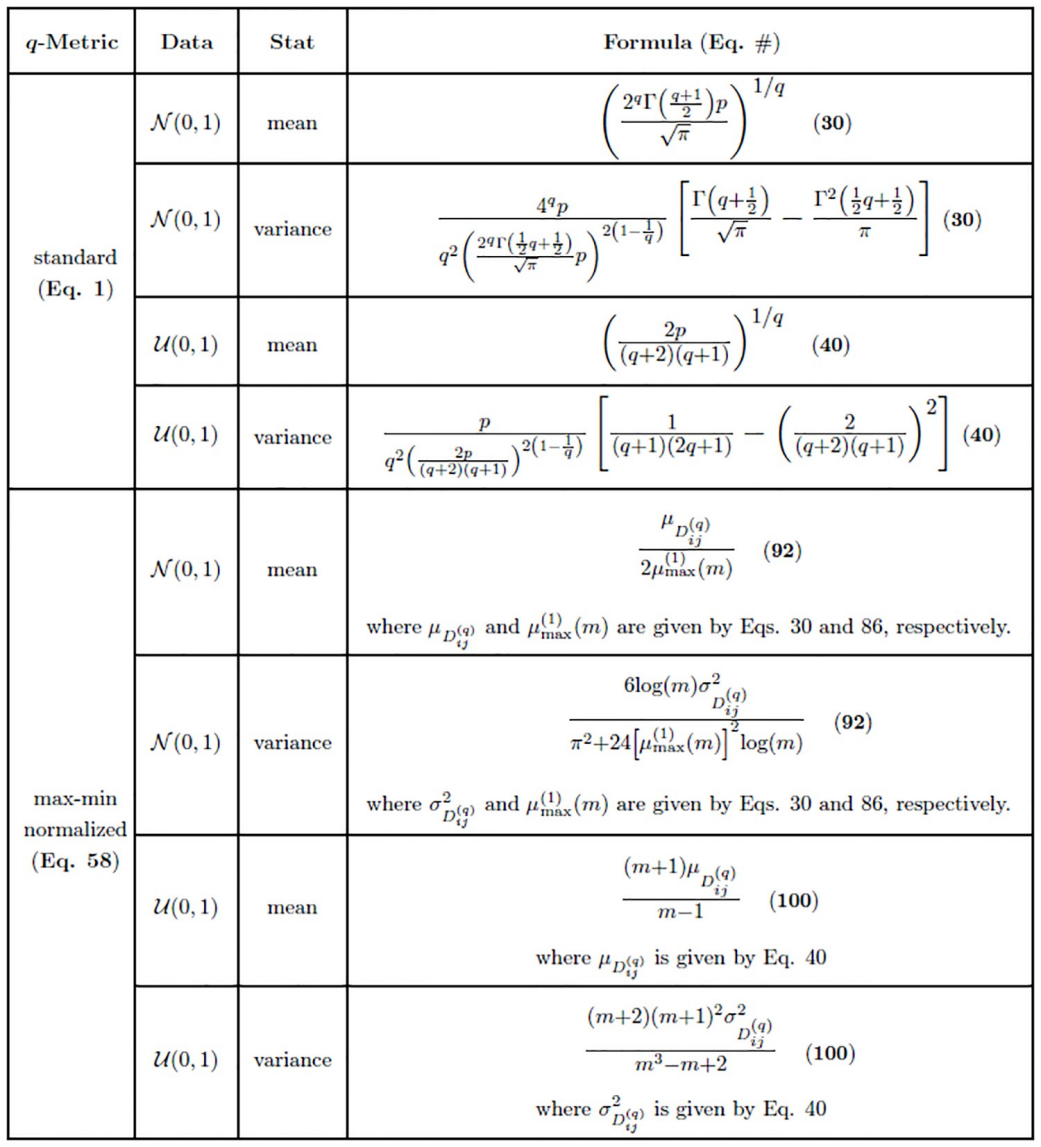
Summary of distance distribution derivations for standard normal (N(0,1)) and standard uniform (U(0,1)) data. Asymptotic estimates are given for both standard (Eq 1) and max-min normalized (Eq 58) q-metrics. These estimates are relevant for all q∈N and *p* ≫ 1 for which the normality assumption of distances holds.

As a useful reference, we tabulate the moment estimates (Eq 30) for the *L*_*q*_ metric on standard normal and uniform data (Fig 2). The derivations for standard uniform data are given in the next subsection. The table is organized by data type (normal or uniform), type of statistic (mean or variance), and corresponding asymptotic formula.

#### 3.1.2 Standard uniform data

If $X_{ia},X_{ja}\overset{iid}{∼}U\left(0,1\right)$, then the marginal density functions with respect to *X* for *X*_*ia*_, $X_{ia}-{\left(Z_{a}^{q}\right)}^{1/q}$, and $X_{ia}+{\left(Z_{a}^{q}\right)}^{1/q}$ are defined as

$$\begin{array}{c} f_{X}\left(x_{ia}\right)=1,\;0\leq x_{ia}\leq 1 \end{array}$$ (31)

$$\begin{array}{c} f_{X}\left(x_{ia}-{\left(z_{a}^{q}\right)}^{1/q}\right)=1,\;0\leq x_{ia}-{\left(z_{a}^{q}\right)}^{1/q}\leq 1,\;\text{and} \end{array}$$ (32)

$$\begin{array}{c} f_{X}\left(x_{ia}+{\left(z_{a}^{q}\right)}^{1/q}\right)=1,\;0\leq x_{ia}+{\left(z_{a}^{q}\right)}^{1/q}\leq 1. \end{array}$$ (33)

Substituting these marginal densities (Eqs 31–33) into the more general density function for $Z_{a}^{q}$ (Eq 17), we have

$$\begin{array}{c} \begin{array}{cc} f_{Z_{a}^{q}}\left(z_{a}^{q}\right) & =\frac{1}{q{\left(z_{a}^{q}\right)}^{1-\frac{1}{q}}}\int_{-\infty }^{\infty }f_{X}\left(x_{ia}\right)[f_{X}(x_{ia}-{\left(z_{a}^{q}\right)}^{1/q})+f_{X}(x_{ia}+{\left(z_{a}^{q}\right)}^{1/q})]\text{d}x_{ia},\\ & \;0<z_{a}\leq 1\\ & =\frac{1}{q{\left(z_{a}^{q}\right)}^{1-\frac{1}{q}}}\int_{0}^{1}[f_{X}(x_{ia}-(z_{a}^{q})+f_{X}(x_{ia}+{\left(z_{a}^{q}\right)}^{1/q})]\text{d}x_{ia},\;0<z_{a}\leq 1\\ & =\frac{1}{q{\left(z_{a}^{q}\right)}^{1-\frac{1}{q}}}\int_{\left(z_{a}^{q}\right)}^{1}1\text{d}x_{ia}+\int_{0}^{1-(z_{a}^{q})}1\text{d}x_{ia},\;0<z_{a}\leq 1\\ & =\frac{1}{q{\left(z_{a}^{q}\right)}^{1-\frac{1}{q}}}[(1-(z_{a}^{q}))+(1-(z_{a}^{q}))],\;0<z_{a}\leq 1\\ & =\frac{1}{q}\cdot 2{\left(z_{a}^{q}\right)}^{\frac{1}{q}-1}{\left[1-{\left(z_{a}^{q}\right)}^{1/q}\right]}^{2-1},\;0<z_{a}\leq 1. \end{array} \end{array}$$ (34)

The previous density (Eq 34) is a Kumaraswamy density with parameters $b=\frac{1}{q}$ and *c* = 2 with moment generating function (MGF) given by

$$\begin{array}{c} \begin{array}{cc} M_{n} & =\frac{c\Gamma \left(1+\frac{n}{b}\right)\Gamma \left(c\right)}{\Gamma \left(1+c+\frac{n}{b}\right)}\\ & =\frac{2}{\left(nq+2\right)\left(nq+1\right)}. \end{array} \end{array}$$ (35)

Using this MGF (Eq 35), the mean and variance of $Z_{a}^{q}$ are computed as

$$\begin{array}{c} \mu_{z_{a}^{q}}=\frac{2}{\left(q+2\right)\left(q+1\right)} \end{array}$$ (36)

and

$$\begin{array}{c} \sigma_{z_{a}^{q}}^{2}=\frac{1}{\left(q+1\right)\left(2q+1\right)}-{\left(\frac{2}{\left(q+2\right)\left(q+1\right)}\right)}^{2}. \end{array}$$ (37)

By linearity of the expected value and variance operators under the iid assumption, the mean (Eq 36) and variance (Eq 37) of the random variable $Z_{a}^{q}$ allow the *p*- dimensional mean and variance of the ${\left(D_{ij}^{\left(q\right)}\right)}^{q}$ distribution to be computed directly as

$$\begin{array}{c} \begin{array}{cc} \mu_{{\left(D_{ij}^{\left(q\right)}\right)}^{q}}=\text{E}[{\left(D_{ij}^{\left(q\right)}\right)}^{q}] & =\text{E}\left(\sum_{a\in A}Z_{a}^{q}\right)\\ & =\sum_{a\in A}\text{E}\left(Z_{a}^{q}\right)\\ & =\sum_{a\in A}\frac{2}{\left(q+2\right)\left(q+1\right)}\\ & =\frac{2p}{\left(q+2\right)\left(q+1\right)} \end{array} \end{array}$$ (38)

and

$$\begin{array}{c} \begin{array}{cc} \sigma_{{\left(D_{ij}^{\left(q\right)}\right)}^{q}}^{2}=\text{Var}[{\left(D_{ij}^{\left(q\right)}\right)}^{q}] & =\text{Var}\left(\sum_{a\in A}Z_{a}^{q}\right)\\ & =\sum_{a\in A}\text{Var}\left(Z_{a}^{q}\right)\\ & =\sum_{a\in A}[\frac{1}{\left(q+1\right)\left(2q+1\right)}-{\left(\frac{2}{\left(q+2\right)\left(q+1\right)}\right)}^{2}]\\ & =[\frac{1}{\left(q+1\right)\left(2q+1\right)}-{\left(\frac{2}{\left(q+2\right)\left(q+1\right)}\right)}^{2}]p. \end{array} \end{array}$$ (39)

Therefore, the asymptotic distribution of $D_{ij}^{\left(q\right)}$ for standard uniform data is

$$N({\left(\frac{2p}{\left(q+2)(q+1\right)}\right)}^{1/q},\frac{p}{q^{2}{\left(\frac{2p}{\left(q+2)(q+1\right)}\right)}^{2(1-\frac{1}{q})}}[\frac{1}{\left(q+1)(2q+1\right)}-{\left(\frac{2}{\left(q+2)(q+1\right)}\right)}^{2}]).$$ (40)

As previously noted, we tabulate the moment estimates (Eq 40) for the *L*_*q*_ metric on standard uniform data along with standard normal data (Fig 2). The summary is organized by data type (normal or uniform), type of statistic (mean or variance), and corresponding asymptotic formula. In the next subsections, we show the asymptotic moments of the distance distribution for standard normal and standard uniform data for the special case of Manhattan (*q* = 1) and Euclidean (*q* = 2) metrics. These are the most commonly applied metrics in the context of nearest-neighbor feature selection, so they are of particular interest.

### 3.2 Manhattan (*L*_1_)

With our general formulas for the asymptotic mean and variance (Eqs 30 and 40) for any value of q∈N, we can simply substitute a particular value of *q* in order to determine the asymptotic distribution of the corresponding distance *L*_*q*_ metric. We demonstrate this with the example of the Manhattan metric (*L*_1_) for standard normal and standard uniform data (Eq 1, *q* = 1).

#### 3.2.1 Standard normal data

Substituting *q* = 1 into the asymptotic formula for the mean *L*_*q*_ distance (Eq 30), we have the following for expected *L*_1_ distance between two independently sample instances i,j∈I in standard normal data

$$\begin{array}{c} \begin{array}{cc} \text{E}\left(D_{ij}^{\left(1\right)}\right) & ={\left(2\frac{\Gamma \left(\frac{1+1}{2}\right)}{\sqrt{\pi }}p\right)}^{1/1}\\ & =\frac{2p}{\sqrt{\pi }}. \end{array} \end{array}$$ (41)

We see in the formula for the expected Manhattan distance (Eq 41) that $D_{ij}^{\left(1\right)}∼p$ in the limit, which implies that this distance is unbounded as feature dimension *p* increases.

Substituting *q* = 1 into the formula for the asymptotic variance of $D_{ij}^{\left(1\right)}$ (Eq 30) leads to the following

$$\begin{array}{c} \begin{array}{cc} \text{Var}\left(D_{ij}^{\left(1\right)}\right) & =\frac{4^{1}p}{1^{2}{\left(\frac{2^{1}\Gamma \left(\frac{1}{2}\left(1\right)+\frac{1}{2}\right)}{\sqrt{\pi }}p\right)}^{2\left(1-\frac{1}{1}\right)}}[\frac{\Gamma \left(1+\frac{1}{2}\right)}{\sqrt{\pi }}-\frac{\Gamma^{2}\left(\frac{1}{2}\left(1\right)+\frac{1}{2}\right)}{\pi }]\\ & =\frac{2\left(\pi -2\right)p}{\pi }. \end{array} \end{array}$$ (42)

**Fig 3:**
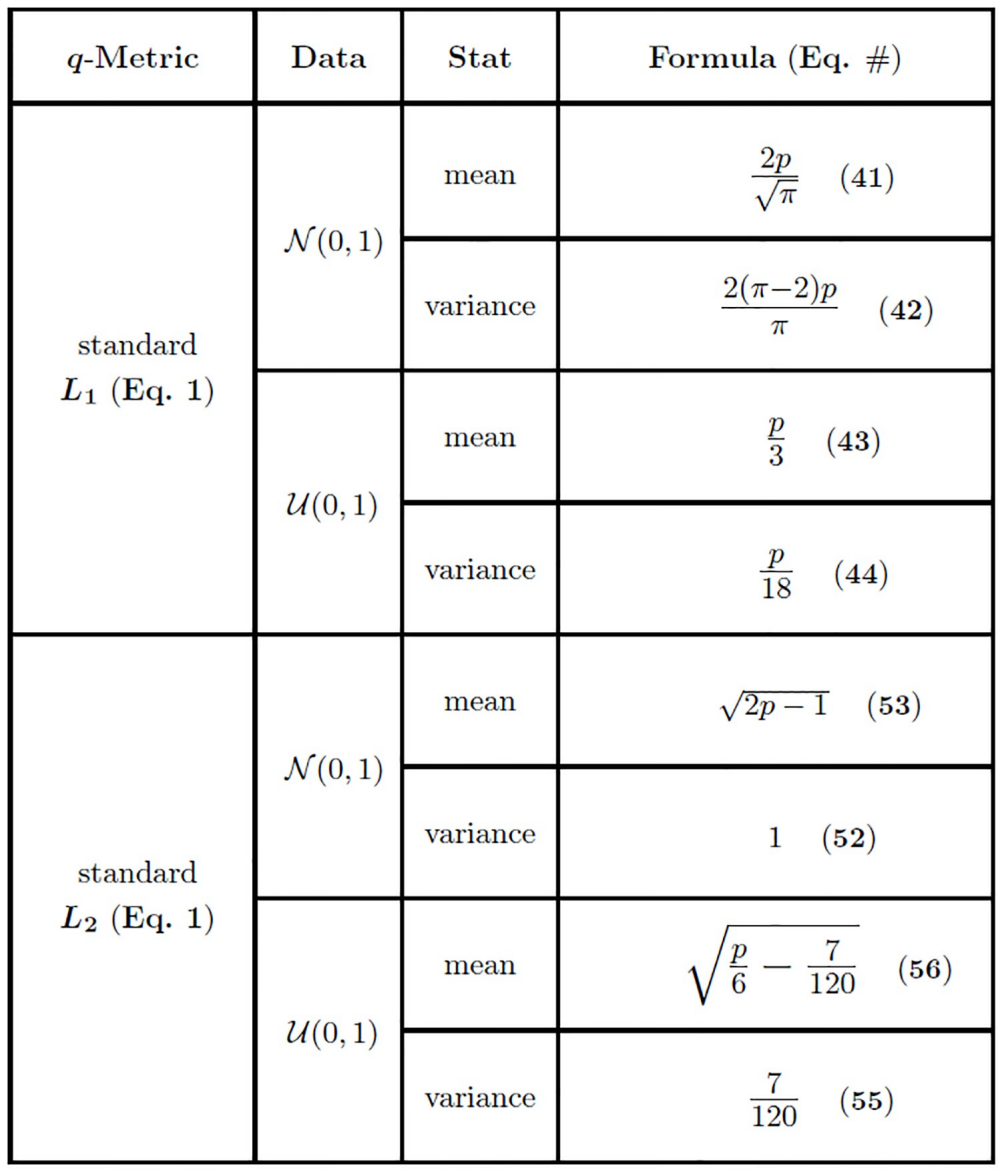
Asymptotic estimates of means and variances for the standard *L*_1_ and *L*_2_ (*q* = 1 and *q* = 2 in Fig 2) distance distributions. Estimates for both standard normal (N(0,1)) and standard uniform (U(0,1)) data are given.

Similar to the mean (Eq 41), the limiting variance of $D_{ij}^{\left(1\right)}$ (Eq 42) grows on the order of feature dimension *p*, which implies that points become more dispersed as the dimension increases. The summary of moment estimates given in this section (Eqs 41 and 42) is organized by metric, data type, statistic (mean or variance), and asymptotic formula (Fig 3).

#### 3.2.2 Standard uniform data

Substituting *q* = 1 into the asymptotic formula of the mean (Eq 40), we have the following for the expected *L*_1_ distance between two independently sampled instances i,j∈I in standard uniform data

$$\begin{array}{c} \begin{array}{cc} \text{E}\left(D_{ij}^{\left(1\right)}\right) & ={\left(\frac{2p}{\left(1+2\right)\left(1+1\right)}\right)}^{1/1}\\ & =\frac{p}{3}. \end{array} \end{array}$$ (43)

Once again, we see that the mean of $D_{ij}^{\left(1\right)}$ (Eq 43) grows on the order of *p* just as in the case of standard normal data.

Substituting *q* = 1 into the formula of the asymptotic variance of $D_{ij}^{\left(1\right)}$ (Eq 40) leads to the following

$$\begin{array}{c} \begin{array}{cc} \text{Var}\left(D_{ij}^{\left(1\right)}\right) & =\frac{p}{1^{2}{\left(\frac{2p}{\left(1+2\right)\left(1+1\right)}\right)}^{2\left(1-\frac{1}{1}\right)}}[\frac{1}{\left(1+1\right)\left(2\left(1\right)+1\right)}-{\left(\frac{2}{\left(1+2\right)\left(1+1\right)}\right)}^{2}]\\ & =\frac{p}{18}. \end{array} \end{array}$$ (44)

As in the case of the *L*_1_ metric on standard normal data, we have a variance (Eq 44) that grows on the order of *p*. The distances between points in high-dimensional uniform data become more widely dispersed with this metric. The summary of moment estimates given in this section (Eqs 43 and 44) is organized by metric, data type, statistic (mean or variance), and asymptotic formula (Fig 3).

#### 3.2.3 Distribution of one-dimensional projection of pairwise distance onto an attribute

In nearest-neighbor distance-based feature selection like NPDR and Relief-based algorithms, the one-dimensional projection of the pairwise distance onto an attribute (Eq 2) is particularly fundamental to feature quality for association with an outcome. For instance, this distance projection is the predictor used to determine beta coefficients in NPDR. In particular, understanding distributional properties of the projected distances is necessary for defining pseudo P values for NPDR. In this section, we summarize the exact distribution of the one-dimensional projected distance onto an attribute a∈A. These results apply to continuous data, such as gene expression.

In previous sections, we derived the exact density function (Eq 17) and moments (Eqs 18 and 19) for the distribution of $Z_{a}^{q}={\left|X_{ia}-X_{ja}\right|}^{q}$. We then derived the exact density (Eq 25) and moments (Eqs 26 and 27) for standard normal data. Analogously, we formulated the exact density (Eq 34) and moments (Eqs 36 and 37) for standard uniform data. From these exact densities and moments, we simply substitute *q* = 1 to define the distribution of the one-dimensional projected distance onto an attribute a∈A.

Assuming data is standard normal, we substitute *q* = 1 into the density function of $Z_{a}^{q}$ (Eq 25) to arrive at the following density function

$$\begin{array}{c} \begin{array}{cc} f_{Z_{a}^{1}}\left(z_{a}^{1}\right) & =\frac{\frac{2}{1}}{{\left(2^{1}\right)}^{1/1}\Gamma \left(\frac{\frac{1}{1}}{\frac{2}{1}}\right)}{\left(z_{a}^{1}\right)}^{1/1-1}e^{-{\left(\frac{z_{a}^{1}}{2^{1}}\right)}^{2/1}},\;z_{a}>0\\ & =\frac{1}{\sqrt{\pi }}z_{a}e^{-\frac{1}{4}z_{a}^{2}},\;z_{a}>0. \end{array} \end{array}$$ (45)

The mean corresponding to this Generalized Gamma density is computed by substituting *q* = 1 into the formula for the mean of $Z_{a}^{q}$ (Eq 26). This result is given by

$$\begin{array}{c} \begin{array}{cc} \mu_{Z_{a}^{1}} & =\frac{2^{1}\Gamma \left(\frac{1+1}{2}\right)}{\sqrt{\pi }}\\ & =\frac{2}{\sqrt{\pi }}. \end{array} \end{array}$$ (46)

Substituting *q* = 1 into Eq 27 for the variance, we have the following

$$\begin{array}{c} \begin{array}{cc} \sigma_{Z_{a}^{1}}^{2} & =4^{1}[\frac{\Gamma \left(1+\frac{1}{2}\right)}{\sqrt{\pi }}-\frac{\Gamma^{2}\left(\frac{1}{2}\cdot 1+\frac{1}{2}\right)}{\pi }]\\ & =\frac{2\left(\pi -2\right)}{\pi }. \end{array} \end{array}$$ (47)

These last few results (Eqs 45–47) provide us with the distribution for NPDR predictors when the data is from the standard normal distribution. We show density curves for *q* = 1, 2, …, 5 for the one-dimensional projection for standard normal data (S22 A Fig in S1 File).

If we have standard uniform data, we substitute *q* = 1 into the density function of $Z_{a}^{q}$ (Eq 34) to obtain the following density function

$$\begin{array}{c} \begin{array}{cc} f_{Z_{a}^{1}} & =\frac{1}{1}\cdot 2{\left(z_{a}^{1}\right)}^{1/1-1}{\left[1-{\left(z_{a}^{1}\right)}^{1/1}\right]}^{2-1},\;0<z_{a}\leq 1\\ & =2z_{a}\left(1-z_{a}\right),\;0<z_{a}\leq 1. \end{array} \end{array}$$ (48)

The mean corresponding to this Kumaraswamy density is computed by substituting *q* = 1 into the formula for the mean of $Z_{a}^{q}$ (Eq 36). After substitution, we have the following result

$$\begin{array}{c} \begin{array}{cc} \mu_{Z_{a}^{1}} & =\frac{2}{\left(1+2\right)\left(1+1\right)}\\ & =\frac{1}{3}. \end{array} \end{array}$$ (49)

Substituting *q* = 1 into the formula for the variance of $Z_{a}^{q}$ (Eq 37), we have the following

$$\begin{array}{c} \begin{array}{cc} \sigma_{Z_{a}^{1}}^{2} & =\frac{1}{\left(1+1\right)\left(2\cdot 1+1\right)}-{\left(\frac{2}{\left(1+2\right)\left(1+1\right)}\right)}^{2}\\ & =\frac{1}{18}. \end{array} \end{array}$$ (50)

In the event that the data distribution is standard uniform, the density function (Eq 48), the mean (Eq 49), and the variance (Eq 50) sufficiently define the distribution for NPDR predictors. As in the case of NPDR predictors for standard normal data, we show density curves for *q* = 1, 2, …, 5 for the NPDR predictor distribution for standard uniform data (S22 B Fig in S1 File).

The means (Eqs 46 and 49) and variances (Eqs.47 and 50) come from the exact distribution of pairwise distances with respect to a single attribute a∈A. This is the distribution of the so-called “projection” of the pairwise distance onto a single attribute to which we have been referring, which is a direct implication from our more general derivations. In a similar manner, one can substitute any value of *q* ≥ 2 into the general densities of $Z_{a}^{q}$ for standard normal (Eq 25) and standard uniform (Eq 34) to derive the associated density of $Z_{a}^{q}={\left|X_{ia}-X_{ja}\right|}^{q}$ for the given data type.

### 3.3 Euclidean (*L*_2_)

Moment estimates for the Euclidean metric are obtained by substituting *q* = 2 into the asymptotic moment formulas for standard normal data (Eq 30) and standard uniform data (Eq 40). As in the case of the Manhattan metric in the previous sections, we initially proceed by deriving Euclidean distance moments in standard normal data.

#### 3.3.1 Standard normal data

Substituting *q* = 2 into the asymptotic formula of the mean (Eq 30), we have the following for expected *L*_2_ distance between two independently sampled instances i,j∈I in standard normal data

$$\begin{array}{c} \begin{array}{cc} \text{E}\left(D_{ij}^{\left(2\right)}\right) & ={\left(2\frac{\Gamma \left(\frac{2+1}{2}\right)}{\sqrt{\pi }}p\right)}^{1/2}\\ & =\sqrt{2p}. \end{array} \end{array}$$ (51)

In the case of *L*_2_ on standard normal data, we see that the mean of $D_{ij}^{\left(2\right)}$ (Eq 51) grows on the order of $\sqrt{p}$. Hence, the Euclidean distance does not increase as quickly as the Manhattan distance on standard normal data.

Substituting *q* = 2 into the formula for the asymptotic variance of $D_{ij}^{\left(2\right)}$ (Eq 30) leads to the following

$$\begin{array}{c} \begin{array}{cc} \text{Var}\left(D_{ij}^{\left(2\right)}\right) & =\frac{4^{2}p}{2^{2}{\left(\frac{2^{2}\Gamma \left(\frac{1}{2}\left(2\right)+\frac{1}{2}\right)}{\sqrt{\pi }}p\right)}^{2\left(1-\frac{1}{2}\right)}}[\frac{\Gamma \left(2+\frac{1}{2}\right)}{\sqrt{\pi }}-\frac{\Gamma^{2}\left(\frac{1}{2}\left(2\right)+\frac{1}{2}\right)}{\pi }]\\ & =1. \end{array} \end{array}$$ (52)

Surprisingly, the asymptotic variance (Eq 52) is just 1. Regardless of data dimensions *m* and *p*, the variance of Euclidean distances on standard normal data tends to 1. Therefore, most instances are contained within a ball of radius 1 about the mean in high feature dimension *p*. This means that the Euclidean distance distribution on standard normal data is simply a horizontal shift to the right of the standard normal distribution.

For the case in which the number of attributes *p* is small, we have an improved estimate of the mean (Eq 9). The lower dimensional estimate of the mean is given by

$$\begin{array}{c} \begin{array}{cc} \text{E}\left(D_{ij}^{\left(2\right)}\right) & ={\left(2\frac{\Gamma \left(\frac{2+1}{2}\right)}{\sqrt{\pi }}p-1\right)}^{1/2}\\ & =\sqrt{2p-1}. \end{array} \end{array}$$ (53)

For high dimensional data sets like gene expression [20, 21], which typically contain thousands of genes (or features), it is clear that the magnitude of *p* will be sufficient to use the standard asymptotic estimate (Eq 51) since $\sqrt{2p}\approx \sqrt{2p-1}$ in that case. The summary of moment estimates given in this section (Eqs 52 and 53) is organized by metric, data type, statistic (mean or variance), and asymptotic formula (Fig 3).

#### 3.3.2 Standard uniform data

Substituting *q* = 2 into the asymptotic formula of the mean (Eq 40), we have the following for expected *L*_2_ distance between two independently sampled instances i,j∈I in standard uniform data

$$\begin{array}{c} \begin{array}{cc} \text{E}\left(D_{ij}^{\left(2\right)}\right) & ={\left(\frac{2p}{\left(2+2\right)\left(2+1\right)}\right)}^{1/2}\\ & =\sqrt{\frac{p}{6}}. \end{array} \end{array}$$ (54)

As in the case of standard normal data, the expected value of $D_{ij}^{\left(2\right)}$ (Eq 54) grows on the order of $\sqrt{p}$.

Substituting *q* = 2 into the formula for the asymptotic variance of $D_{ij}^{\left(2\right)}$ (Eq 40) leads to the following

$$\begin{array}{c} \begin{array}{cc} \text{Var}\left(D_{ij}^{\left(2\right)}\right) & =\frac{p}{2^{2}{\left(\frac{2p}{\left(2+2\right)\left(2+1\right)}\right)}^{2\left(1-\frac{1}{2}\right)}}[\frac{1}{\left(2+1\right)\left(2\left(2\right)+1\right)}-{\left(\frac{2}{\left(2+2\right)\left(2+1\right)}\right)}^{2}]\\ & =\frac{7}{120}. \end{array} \end{array}$$ (55)

Once again, the variance of Euclidean distance surprisingly approaches a constant.

For the case in which the number of attributes *p* is small, we have an improved estimate of the mean (Eq 9). The lower dimensional estimate of the mean is given by

$$\begin{array}{c} \begin{array}{cc} \text{E}\left(D_{ij}^{\left(2\right)}\right) & ={\left(\frac{2p}{\left(2+2\right)\left(2+1\right)}-\frac{7}{120}\right)}^{1/2}\\ & =\sqrt{\frac{p}{6}-\frac{7}{120}}. \end{array} \end{array}$$ (56)

We summarize the moment estimates given in this section for standard *L*_*q*_ metrics (Eqs 55 and 56) organized by metric, data type, statistic (mean or variance), and asymptotic formula (Fig 3). In the next section, we extend these results for the standard *L*_*q*_ metric to derive asymptotics for the attribute range-normalized (max-min) *L*_*q*_ metric used frequently in Relief-based algorithms [1, 3] for scoring attributes. These derivations use extreme value theory to handle the maximum and minimum attributes for standard normal and standard uniform data.

## 4 Moments for max-min normalized *L*_*q*_ metric

In this section, we derive formulas for asymptopic means and variances of a special *L*_*q*_ metric that is used in Relief-based feature selection methods. In this metric, the difference between pairs of subjects for a given attribute is normalized by the difference between the maximum and minimum of the attribute. For Relief-based methods [1, 3], the standard numeric difference metric (diff) is given by

$$\begin{array}{c} {\text{d}}_{ij}^{\text{num}}\left(a\right)=\text{diff}\left(a,\left(i,j\right)\right)=\frac{|X_{ia}-X_{ja}|}{\text{max}\left(a\right)-\text{min}\left(a\right)}, \end{array}$$ (57)

where $\text{max}\left(a\right)=\max_{k\in I}\left\{X_{ka}\right\}$, $\text{min}\left(a\right)=\min_{k\in I}\left\{X_{ka}\right\}$, and I={1,2,…,m}. The pairwise distance using this max-min normalized diff metric is then computed as

$$\begin{array}{c} \begin{array}{cc} D_{ij}^{\left(q*\right)} & ={\left(\sum_{a\in A}{\left|{\text{d}}_{ij}\left(a\right)\right|}^{q}\right)}^{1/q}\\ & ={\left(\sum_{a\in A}{\left(\frac{|X_{ia}-X_{ja}|}{\text{max}\left(a\right)-\text{min}\left(a\right)}\right)}^{q}\right)}^{1/q}. \end{array} \end{array}$$ (58)

This normalization leads to Relief attribute scores that are constrained to the interval [−1, 1]. The derivations in this section will invoke extreme value theory (EVT) because of the use of attribute extrema in the metric.

### 4.1 Distribution of max-min normalized *L*_*q*_ metric

We observe empirically that Gaussian convergence applies to the max-min normalized *L*_*q*_ metric in the case of continuous data. We show this behavior for the special cases of standard uniform (S1 Fig in S1 File) and standard normal (S3 Fig in S1 File). In order to determine moments of asymptotic max-min normalized distance (Eq 57) distributions, we will first derive the asymptotic extreme value distributions of the attribute maximum and minimum. Although the exact distribution of the maximum or minimum requires an assumption about the data distribution, the Fisher-Tippett-Gnedenko Theorem is an important result that allows one to generally categorize the extreme value distribution for a collection of independent and identically distributed random variables into one of three distributional families. This theorem does not, however, tell us the exact distribution of the maximum that we require in order to determine asymptotic results for the max-min normalized distance (Eq 58). We mention this theorem simply to provide some background on convergence of extreme values. Before stating the theorem, we first need the following definition

**Definition 4.1** *A distribution* $F_{X}$ *is said to be **degenerate** if its density function f*_*X*_ *is the Dirac delta δ*(*x* − *c*_0_) *centered at a constant* $c_{0}\in R$, *with corresponding distribution function F*_*X*_ *defined as*

$$F_{X}\left(x\right)=\{\begin{array}{cc} 1, & x\geq c_{0},\\ 0, & x<c_{0}. \end{array}$$

**Theorem 4.1 (Fisher-Tippett-Gnedenko)** *Let* $X_{1a},X_{2a},\ldots ,X_{ma}\overset{iid}{∼}F_{X}\left(\mu_{x},\sigma_{x}^{2}\right)$ *and let* $X_{a}^{\text{max}}=\max_{k\in I}\left\{X_{ka}\right\}$. *If there exists two non-random sequences b*_*m*_ > 0 *and c*_*m*_ *such that*

$$\lim_{m\to \infty }\text{P}\left(\frac{X_{a}^{\text{max}}-c_{m}}{b_{m}}\leq x\right)=G_{X}\left(x\right),$$

*where G*_*X*_ *is a non-degenerate distribution function, then the limiting distribution* $G_{X}$ *is in the Gumbel, Fréchet, or Wiebull family*.

The three distribution families given in Theorem 4.1 are actually special cases of the Generalized Extreme Value Distribution. In the context of extreme values, Theorem 4.1 is analogous to the Central Limit Theorem for the distribution of sample mean. Although we will not explicitly invoke this theorem, it does tell us something very important about the asymptotic behavior of sample extremes under certain necessary conditions. For illustration of this general phenomenon of sample extremes, we derive the distribution of the maximum for standard normal data to show that the limiting distribution is in the Gumbel family, which is a known result. In the case of standard uniform data, we will derive the distribution of the maximum and minimum directly. Regardless of data type, the distribution of the sample maximum can be derived as follows

$$\begin{array}{c} \begin{array}{cc} \text{P}[X_{a}^{\text{max}}\leq x] & =\text{P}[\max_{k\in I}\left\{X_{ka}\right\}\leq x]\\ & =\text{P}[X_{1a}\leq x,X_{2a}\leq x,\ldots ,X_{ma}\leq x]\\ & =\prod_{k=1}^{m}\text{P}\left[X_{ka}\leq x\right]\\ & =\prod_{k=1}F_{X}\left(x\right)\\ & ={\left[F_{X}\left(x\right)\right]}^{m}. \end{array} \end{array}$$ (59)

Using more precise notation, the distribution function of the sample maximum in standard normal data is

$$\begin{array}{c} F_{\text{max}}\left(x\right)={\left[F_{X}\left(x\right)\right]}^{m}, \end{array}$$ (60)

where *m* is the size of the sample from which the maximum is derived and *F*_*X*_ is the distribution function corresponding to the data sample. This means that the distribution of the sample maximum relies only on the distribution function of the data from which extremes are drawn *F*_*X*_ and the size of the sample *m*.

Differentiating the distribution function (Eq 60) gives us the following density function for the distribution of the maximum

$$\begin{array}{c} \begin{array}{cc} f_{\text{max}}\left(x\right) & =\frac{\text{d}}{\text{d}x}F_{\text{max}}\left(x\right)\\ & =\frac{\text{d}}{\text{d}x}{\left[F_{X}\left(x\right)\right]}^{m}\\ & =m{\left[F_{X}\left(x\right)\right]}^{m-1}f_{X}\left(x\right), \end{array} \end{array}$$ (61)

where *m* is the size of the sample from which the maximum is derived, *F*_*X*_ is the distribution function corresponding to the data sample, and *f*_*X*_ is the density function corresponding to the data sample. Similar to the distribution function for the sample maximum (Eq 60), the density function (Eq 61) relies only on the distribution and density function of the data from which extremes are derived.

The distribution of the sample minimum, $X_{a}^{\text{min}}$, can be derived as follows

$$\begin{array}{c} \begin{array}{cc} \text{P}[X_{a}^{\text{min}}\leq x] & =1-\text{P}[X_{a}^{\text{min}}\geq x]\\ & =1-\text{P}[\min_{k\in I}\left\{X_{ka}\right\}\geq x]\\ & =1-\text{P}[X_{1a}\geq x,X_{2a}\geq x,\ldots ,X_{ma}\geq x]\\ & =1-\prod_{k=1}^{m}\text{P}\left[X_{ka}\geq x\right]\\ & =1-{\left[\text{P}[X_{1a}\geq x]\right]}^{m}\\ & =1-{\left[1-\text{P}[X_{1a}\leq x]\right]}^{m}\\ & =1-{\left[1-F_{X}\left(x\right)\right]}^{m}, \end{array} \end{array}$$ (62)

where *m* is the size of the sample from which the maximum is derived and *F*_*X*_ is the distribution function corresponding to the data sample. Therefore, the distribution of sample minimum also relies only on the distribution function of the data from which extremes are derived.

With more precise notation, we have the following expression for the distribution function of the minimum

$$\begin{array}{c} F_{\text{min}}\left(x\right)=1-{\left[1-F_{X}\left(x\right)\right]}^{m}. \end{array}$$ (63)

where *m* is the size of the sample from which the minimum is derived and *F*_*X*_ is the distribution function corresponding to the data sample.

Differentiating the distribution function (Eq 63) gives us the following density function for the distribution of sample minimum

$$\begin{array}{c} \begin{array}{cc} f_{\text{min}}\left(x\right) & =\frac{\text{d}}{\text{d}x}F_{\text{min}}\left(x\right)\\ & =\frac{\text{d}}{\text{d}x}\left(1-{\left[1-F_{X}\left(x\right)\right]}^{m}\right)\\ & =m{\left[1-F_{X}\left(x\right)\right]}^{m-1}f_{X}\left(x\right), \end{array} \end{array}$$ (64)

where *m* is the size of the sample from which the minimum is derived, *F*_*X*_ is the distribution function corresponding to the data sample, and *f*_*X*_ is the density function corresponding to the data sample. As in the case of the density function for sample maximum (Eq 61), the density function for sample minimum relies only on the distribution *F*_*X*_ and density *f*_*X*_ functions of the data from which extremes are derived and the sample size *m*.

Given the densities of the distribution of sample maximum and minimum, we can easily compute the raw moments and variance. The first moment about the origin of the distribution of sample maximum is given by the following

$$\begin{array}{c} \begin{array}{cc} \mu_{\text{max}}^{\left(1\right)}\left(m\right)=\text{E}\left(X_{a}^{\text{max}}\right) & =\int_{-\infty }^{\infty }xf_{\text{max}}\left(x\right)\text{d}x\\ & =\int_{-\infty }^{\infty }x\left(m{\left[F_{X}\left(x\right)\right]}^{m-1}f_{X}\left(x\right)\right)\text{d}x\\ & =m\int_{-\infty }^{\infty }xf_{X}\left(x\right){\left[F_{X}\left(x\right)\right]}^{m-1}\text{d}x, \end{array} \end{array}$$ (65)

where *m* is the sample size, *F*_*X*_ is the distribution function, and *f*_*X*_ is the density function of the data from which the maximum is derived.

The second raw moment of the distribution of sample maximum is derived similarly as follows

$$\begin{array}{c} \begin{array}{cc} \mu_{\text{max}}^{\left(2\right)}\left(m\right)=\text{E}\left[{\left(X_{a}^{\text{max}}\right)}^{2}\right] & =\int_{-\infty }^{\infty }x^{2}f_{\text{max}}\left(x\right)\text{d}x\\ & =\int_{-\infty }^{\infty }x^{2}\left(m{\left[F_{X}\left(x\right)\right]}^{m-1}f_{X}\left(x\right)\right)\text{d}x\\ & =m\int_{-\infty }^{\infty }x^{2}f_{X}\left(x\right){\left[F_{X}\left(x\right)\right]}^{m-1}\text{d}x \end{array} \end{array}$$ (66)

where *m* is the sample size, *F*_*X*_ is the distribution function, and *f*_*X*_ is the density function of the data from which the maximum is derived.

Using the first (Eq 65) and second (Eq 66) raw moments of the distribution of sample maximum, the variance is given by

$$\begin{array}{c} \sigma_{\text{max}}^{2}\left(m\right)=\mu_{\text{max}}^{\left(2\right)}\left(m\right)-{\left[\mu_{\text{max}}^{\left(1\right)}\left(m\right)\right]}^{2}, \end{array}$$ (67)

where *m* is the sample size of the data from which the maximum is derived and $\mu_{\text{max}}^{\left(1\right)}\left(m\right)$ and $\mu_{\text{max}}^{\left(2\right)}$ are the first and second raw moments, respectively, of the distribution of sample maximum.

Moving on to the distribution of sample minimum, the first raw moment is given by the following

$$\begin{array}{c} \begin{array}{cc} \mu_{\text{min}}^{\left(1\right)}\left(m\right)=\text{E}\left(X_{a}^{\text{min}}\right) & =\int_{-\infty }^{\infty }xf_{\text{min}}\left(x\right)\text{d}x\\ & =\int_{-\infty }^{\infty }x\left(m{\left[1-F_{X}\left(x\right)\right]}^{m-1}f_{X}\left(x\right)\right)\text{d}x\\ & =m\int_{-\infty }^{\infty }xf_{X}\left(x\right){\left[1-F_{X}\left(x\right)\right]}^{m-1}\text{d}x, \end{array} \end{array}$$ (68)

where *m* is the sample size, *F*_*X*_ is the distribution function, and *f*_*X*_ is the density function of the data from which the minimum is derived.

Similarly, the second raw moment of the distribution of sample minimum is given by the following

$$\begin{array}{c} \begin{array}{cc} \mu_{\text{min}}^{\left(2\right)}\left(m\right)=\text{E}\left[{\left(X_{a}^{\text{min}}\right)}^{2}\right] & =\int_{-\infty }^{\infty }x^{2}f_{\text{min}}\left(x\right)\text{d}x\\ & =\int_{-\infty }^{\infty }x^{2}\left(m{\left[1-F_{X}\left(x\right)\right]}^{m-1}f_{X}\left(x\right)\right)\text{d}x\\ & =m\int_{-\infty }^{\infty }x^{2}f_{X}\left(x\right){\left[1-F_{X}\left(x\right)\right]}^{m-1}\text{d}x, \end{array} \end{array}$$ (69)

where *m* is the sample size, *F*_*X*_ is the distribution function, and *f*_*X*_ is the density function of the data from which the minimum is derived.

Using the first (Eq 68) and second (Eq 69) raw moments of the distribution of sample minimum, the variance is given by

$$\begin{array}{c} \sigma_{\text{min}}^{2}\left(m\right)=\mu_{\text{min}}^{\left(2\right)}\left(m\right)-{\left[\mu_{\text{min}}^{\left(1\right)}\left(m\right)\right]}^{2}, \end{array}$$ (70)

where *m* is the sample size of the data from which the maximum is derived and $\mu_{\text{min}}^{\left(1\right)}\left(m\right)$ and $\mu_{\text{min}}^{\left(2\right)}$ are the first and second raw moments, respectively, of the distribution of sample maximum.

Using the expected attribute maximum (Eq 65) and minimum (Eq 68) for sample size *m*, the following expected attribute range results from linearity of the expectation operator

$$\begin{array}{c} \begin{array}{cc} \text{E}\left(X_{a}^{\text{max}}-X_{a}^{\text{min}}\right) & =\text{E}\left(X_{a}^{\text{max}}\right)-\text{E}\left(X_{a}^{\text{min}}\right)\\ & =\mu_{\text{max}}^{\left(1\right)}\left(m\right)-\mu_{\text{min}}^{\left(1\right)}\left(m\right). \end{array} \end{array}$$ (71)

where $\mu_{\text{max}}^{\left(1\right)}\left(m\right)$ is the expected sample maximum (Eq 65) and $\mu_{\text{min}}^{\left(1\right)}\left(m\right)$ is the expected sample minimum.

For a data distribution whose density is an even function, the expected attribute range (Eq 71) can be simplified to the following expression

$$\begin{array}{c} \text{E}\left(X_{a}^{\text{max}}-X_{a}^{\text{min}}\right)=2\mu_{\text{max}}^{\left(1\right)}\left(m\right), \end{array}$$ (72)

where *m* is the size of the sample from which the maximum is derived. Hence, the expected attribute range is simply twice the expected attribute maximum (Eq 65). This result naturally applies to standard normal data, which is symmetric about its mean at 0 and without any skewness.

For large samples (*m* ≫ 1) from an exponential type distribution that has infinite support and all moments, the covariance between the sample maximum and minimum is approximately zero [22]. In this case, the variance of the attribute range of a sample of size *m* is given by the following

$$\begin{array}{c} \begin{array}{cc} \text{Var}\left(X_{a}^{\text{max}}-X_{a}^{\text{min}}\right) & \approx \text{Var}\left(X_{a}^{\text{max}}\right)+\text{Var}\left(X_{a}^{\text{min}}\right)\\ & =\sigma_{\text{max}}^{2}\left(m\right)+\sigma_{\text{min}}^{2}\left(m\right). \end{array} \end{array}$$ (73)

Under the assumption of zero skewness, infinite support and even density function, sufficiently large sample size *m*, and distribution of an exponential type for all moments, the variance of attribute range (Eq 73) simplifies to the following

$$\begin{array}{c} \begin{array}{cc} \text{Var}\left(X_{a}^{\text{max}}-X_{a}^{\text{min}}\right) & =2\text{Var}\left(X_{a}^{\text{max}}\right)\\ & =2\sigma_{\text{max}}^{2}. \end{array} \end{array}$$ (74)

Let $\mu_{D_{ij}^{\left(q\right)}}$ and $\sigma_{D_{ij}^{\left(q\right)}}^{2}$ (Eq 21) denote the mean and variance of the standard *L*_*q*_ distance metric (Eq 1). Then the expected value of the max-min normalized distance (Eq 58) distribution is given by the following

$$\begin{array}{c} \begin{array}{cc} \mu_{D_{ij}^{\left(q*\right)}} & =\text{E}[{\left(\sum_{a\in A}{\left(\frac{|X_{ia}-X_{ja}|}{X_{a}^{\text{max}}-X_{a}^{\text{min}}}\right)}^{q}\right)}^{1/q}]\\ & \approx \frac{1}{\text{E}\left(X_{a}^{\text{max}}-X_{a}^{\text{min}}\right)}\text{E}[{\left(\sum_{a\in A}{\left|X_{ia}-X_{ja}\right|}^{q}\right)}^{1/q}]\\ & =\frac{\mu_{D_{ij}^{\left(q\right)}}}{\text{E}\left(X_{a}^{\text{max}}\right)-\text{E}\left(X_{a}^{\text{min}}\right)}\\ & =\frac{\mu_{D_{ij}^{\left(q\right)}}}{\mu_{\text{max}}^{\left(1\right)}\left(m\right)-\mu_{\text{min}}^{\left(1\right)}\left(m\right)}, \end{array} \end{array}$$ (75)

where *m* is the size of the sample from which extremes are derived, $\mu_{\text{max}}^{\left(1\right)}\left(m\right)$ is the expected value of the sample maximum (Eq 65), and $\mu_{\text{min}}^{\left(1\right)}$ is the expected value of the sample minimum.

The variance of the max-min normalized distance (Eq 58) distribution is given by the following

$$\begin{array}{c} \begin{array}{cc} \sigma_{D_{ij}^{\left(q*\right)}}^{2} & =\text{Var}[{\left(\sum_{a\in A}{\left(\frac{|X_{ia}-X_{ja}|}{X_{a}^{\text{max}}-X_{a}^{\text{min}}}\right)}^{q}\right)}^{1/q}]\\ & =\text{E}[{\left(\sum_{a\in A}{\left(\frac{|X_{ia}-X_{ja}|}{X_{a}^{\text{max}}-X_{a}^{\text{min}}}\right)}^{q}\right)}^{2/q}]-{\left(\text{E}[{\left(\sum_{a\in A}{\left(\frac{|X_{ia}-X_{ja}|}{X_{a}^{\text{max}}-X_{a}^{\text{min}}}\right)}^{q}\right)}^{1/q}]\right)}^{2}\\ & \approx \frac{\text{E}[{\left(\sum_{a\in A}{\left|X_{ia}-X_{ja}\right|}^{q}\right)}^{2/q}]}{\text{E}[{\left(X_{a}^{\text{max}}-X_{a}^{\text{min}}\right)}^{2}]}-\frac{{\left(\text{E}[{\left(\sum_{a\in A}{\left|X_{ia}-X_{ja}\right|}^{q}\right)}^{1/q}]\right)}^{2}}{\text{E}[{\left(X_{a}^{\text{max}}-X_{a}^{\text{min}}\right)}^{2}]}\\ & =\frac{\sigma_{D_{ij}^{\left(q\right)}}^{2}+\mu_{D_{ij}^{\left(q\right)}}^{2}}{\text{E}[{\left(X_{a}^{\text{max}}-X_{a}^{\text{min}}\right)}^{2}]}-\frac{\mu_{D_{ij}^{\left(q\right)}}^{2}}{\text{E}[{\left(X_{a}^{\text{max}}-X_{a}^{\text{min}}\right)}^{2}]}\\ & =\frac{\sigma_{D_{ij}^{\left(q\right)}}^{2}}{\text{E}[{\left(X_{a}^{\text{max}}-X_{a}^{\text{min}}\right)}^{2}]}\\ & =\frac{\sigma_{D_{ij}^{\left(q\right)}}^{2}}{\text{E}\left[{\left(X_{a}^{\text{max}}\right)}^{2}\right]-2\text{E}\left(X_{a}^{\text{max}}\right)\text{E}\left(X_{a}^{\text{min}}\right)+\text{E}\left(X_{a}^{\text{min}}\right)}\\ & =\frac{\sigma_{D_{ij}^{\left(q\right)}}^{2}}{\mu_{\text{max}}^{\left(2\right)}\left(m\right)-2\mu_{\text{max}}^{\left(1\right)}\left(m\right)\mu_{\text{min}}^{\left(1\right)}\left(m\right)+\mu_{\text{min}}^{\left(2\right)}\left(m\right)}, \end{array} \end{array}$$ (76)

where *m* is the size of the sample from which extremes are derived, $\mu_{\text{max}}^{\left(1\right)}\left(m\right)$ is the expected value of the sample maximum (Eq 65), and $\mu_{\text{min}}^{\left(1\right)}$ is the expected value of the sample minimum.

With the mean (Eq 75) and variance (Eq 76) of the max-min normalized distance (Eq 58), we have the following generalized estimate for the asymptotic distribution of the max-min normalized distance distribution

$$\begin{array}{c} D_{ij}^{\left(q*\right)}\dot{∼}N\left(\frac{\mu_{D_{ij}^{\left(q\right)}}}{\mu_{\text{max}}^{\left(1\right)}\left(m\right)-\mu_{\text{min}}^{\left(1\right)}\left(m\right)},\frac{\sigma_{D_{ij}^{\left(q\right)}}^{2}}{\mu_{\text{max}}^{\left(2\right)}\left(m\right)-2\mu_{\text{max}}^{\left(1\right)}\left(m\right)\mu_{\text{min}}^{\left(1\right)}\left(m\right)+\mu_{\text{min}}^{\left(2\right)}\left(m\right)}\right), \end{array}$$ (77)

where *m* is the size of the sample from which extremes are derived, $\mu_{\text{max}}^{\left(1\right)}\left(m\right)$ is the expected value of the sample maximum (Eq 65), and $\mu_{\text{min}}^{\left(1\right)}$ is the expected value of the sample minimum.

For data with zero skewness, infinite support, and even density function, the expected sample maximum is the additive inverse of the expected sample minimum. This allows us to express the expected max-min normalized pairwise distance (Eq 75) exclusively in terms of the expected sample maximum. This result is given by the following

$$\begin{array}{c} \mu_{D_{ij}^{\left(q*\right)}}\approx \frac{\mu_{D_{ij}^{\left(q\right)}}}{2\mu_{\text{max}}^{\left(1\right)}\left(m\right)}, \end{array}$$ (78)

where *m* is the size of the sample from which the maximum is derived and $\mu_{\text{max}}^{\left(1\right)}\left(m\right)$ is the expected value of the sample maximum (Eq 65).

A similar substitution gives us the following expression for the variance of the max-min normalized distance distribution

$$\begin{array}{c} \begin{array}{cc} \sigma_{D_{ij}^{\left(q*\right)}}^{2} & \approx \frac{\sigma_{D_{ij}^{\left(q\right)}}^{2}}{2\mu_{\text{max}}^{\left(2\right)}\left(m\right)+2{\left[\mu_{\text{max}}^{\left(1\right)}\left(m\right)\right]}^{2}}\\ & =\frac{\sigma_{D_{ij}^{\left(q\right)}}^{2}}{2\left(\sigma_{\text{max}}^{2}\left(m\right)+{\left[\mu_{\text{max}}^{\left(1\right)}\left(m\right)\right]}^{2}\right)}, \end{array} \end{array}$$ (79)

where *m* is the size of the sample from which extremes are derived, $\mu_{\text{max}}^{\left(1\right)}\left(m\right)$ is the expected value of the sample maximum (Eq 65), and $\sigma_{\text{max}}^{2}\left(m\right)$ is the variance of the sample maximum (Eq 67).

Therefore, the asymptotic distribution of the max-min normalized distance distribution (Eq 77) becomes

$$\begin{array}{c} D_{ij}^{\left(q*\right)}\dot{∼}N\left(\frac{\mu_{D_{ij}^{\left(q\right)}}}{2\mu_{\text{max}}^{\left(1\right)}\left(m\right)},\frac{\sigma_{D_{ij}^{\left(q\right)}}^{2}}{2\left(\sigma_{\text{max}}^{2}\left(m\right)+{\left[\mu_{\text{max}}^{\left(1\right)}\left(m\right)\right]}^{2}\right)}\right), \end{array}$$ (80)

where *m* is the size of the sample from which extremes are derived, $\mu_{\text{max}}^{\left(1\right)}\left(m\right)$ is the expected value of the sample maximum (Eq 65), and $\sigma_{\text{max}}^{2}\left(m\right)$ is the variance of the sample maximum (Eq 67).

We have now derived asymptotic estimates of the moments of the max-min normalized *L*_*q*_ distance metric (Eq 58) for any continuous data distribution. In the next two sections, we examine the max-min normalized *L*_*q*_ distance on standard normal and standard uniform data. As in previous sections in which we analyzed the standard *L*_*q*_ metric (Eq 1), we will use the more general results for the max-min *L*_*q*_ metric to derive asymptotic estimates for normalized Manhattan (*q* = 1) and Euclidean (*q* = 2).

#### 4.1.1 Standard normal data

The standard normal distribution has zero skewness, even density function, infinite support, and all moments. This implies that the corresponding mean and variance of the distribution of sample range can be expressed exclusively in terms of the sample maximum. Given the nature of the density function of the sample maximum for sample size *m*, the integration required to determine the moments (Eqs 65 and 66) is not possible. These moments can either be approximated numerically or we can use extreme value theory to determine the form of the asymptotic distribution of the sample maximum. Using the latter method, we will show that the asymptotic distribution of the sample maximum for standard normal data is in the Gumbel family. Let $c_{m}=-\Phi^{-1}\left(\frac{1}{m}\right)$ and $b_{m}=\frac{1}{c_{m}}$, where Φ is the standard normal cumulative distribution function. Using Taylor’s Theorem, we have the following expansion

$$\begin{array}{c} \begin{array}{cc} \text{log}\Phi \left(-c_{m}-b_{m}x\right) & =\text{log}\Phi \left(-c_{m}\right)-b_{m}x\frac{\phi \left(-c_{m}\right)}{\Phi \left(-c_{m}\right)}+O\left(b_{m}^{2}x^{2}\right)\\ & =\text{log}\left(\frac{1}{m}\right)-x\frac{\phi \left(-c_{m}\right)}{c_{m}\Phi \left(-c_{m}\right)}+O\left(b_{m}^{2}x^{2}\right), \end{array} \end{array}$$ (81)

where *m* is the size of the sample from which the maximum is derived.

In order to simplify the right-hand side of this expansion (Eq 81), we will use the Mills Ratio Bounds [23] given by the following

$$\begin{array}{c} 1\leq \frac{\phi \left(x\right)}{x\Phi \left(-x\right)}\leq 1+\frac{1}{x^{2}}\;,x>0, \end{array}$$ (82)

where Φ and *ϕ* once again represent the cumulative distribution function and density function, respectively, of the standard normal distribution.

The inequalities given above (Eq 82) show that

$$\frac{\phi \left(x\right)}{x\Phi \left(-x\right)}\to 1\;\text{as}\;x\to \infty .$$

This further implies that

$$\frac{\phi \left(c_{m}\right)}{c_{m}\Phi \left(-c_{m}\right)}\to 1\;\text{as}\;m\to \infty$$

since

$$c_{m}=-\Phi^{-1}\left(\frac{1}{m}\right)\to \infty \;\text{as}\;m\to \infty .$$

This gives us the following approximation of the right-hand side of the expansion (Eq 81) given previously

$$\begin{array}{c} \begin{array}{cc} \text{log}\Phi \left(-c_{m}-b_{m}x\right) & \approx \text{log}\left(\frac{1}{m}\right)-x+O\left(b_{m}^{2}x^{2}\right)\\ \Rightarrow \Phi \left(-c_{m}-b_{m}x\right) & \approx \frac{1}{m}e^{-x+O\left(b_{m}^{2}x^{2}\right)}\\ \Rightarrow \Phi \left(c_{m}+b_{m}x\right) & \approx 1-\frac{1}{m}e^{-x+O\left(b_{m}^{2}x^{2}\right)}, \end{array} \end{array}$$ (83)

where *m* is the size of the sample from which the maximum is derived.

Using the approximation of expansion given previously (Eq 83), we now derive the limit distribution for the sample maximum in standard normal data as

$$\begin{array}{c} \begin{array}{cc} \text{P}\left(\frac{X_{a}^{\text{max}}-c_{m}}{b_{m}}\leq x\right) & =\text{P}\left(X_{a}^{\text{max}}\leq c_{m}+b_{m}x\right)\\ & =\Phi^{m}\left(c_{m}+b_{m}x\right)\\ & \approx {\left(1-\frac{1}{m}e^{-x+O\left(b_{m}^{2}x^{2}\right)}\right)}^{m}\\ & ={\left(1-\frac{1}{m}e^{-x+O\left(\frac{1}{c_{m}^{2}}x^{2}\right)}\right)}^{m}\\ & \approx {\left(1-\frac{1}{m}e^{-x}\right)}^{m}\\ \Rightarrow \lim_{m\to \infty }\text{P}\left(\frac{X_{a}^{\text{max}}-c_{m}}{b_{m}}\leq x\right) & =\lim_{m\to \infty }{\left(1-\frac{1}{m}e^{-x}\right)}^{m}\\ & =e^{-e^{-x}}, \end{array} \end{array}$$ (84)

which is the cumulative distribution function of the standard Gumbel distribution. The mean of this distribution is given by the following

$$\begin{array}{c} \text{E}\left(X_{a}^{\text{max}}\right)=\mu_{\text{max}}^{\left(1\right)}=-\Phi^{-1}\left(\frac{1}{m}\right)-\frac{\gamma }{\Phi^{-1}\left(\frac{1}{m}\right)}, \end{array}$$ (85)

where *m* is the size of the sample from which the maximum is derived and *γ* is the Euler-Mascheroni constant. This constant has many equivalent definitions, one of which is given by

$$\gamma =\lim_{m\to \infty }\left(-\text{log}\left(m\right)+\sum_{k=1}^{m}\frac{1}{k}\right).$$

Perhaps a more convenient definition of the Euler-Mascheroni constant is simply

$$\gamma =-\Gamma^{\prime }\left(1\right)=\frac{\text{d}}{\text{d}t}\left(\int_{0}^{\infty }z^{t-1}e^{-z}\text{d}z\right){|}_{t=1},$$

which is just the additive inverse of the first derivative of the gamma function evaluated at 1.

The median of the distribution of the maximum for standard normal data is given by

$$\begin{array}{c} {\tilde{\mu }}_{\text{max}}=\frac{\text{log}\left(\text{log}\left(2\right)\right)}{\Phi^{-1}\left(\frac{1}{m}\right)}-\Phi^{-1}\left(\frac{1}{m}\right), \end{array}$$ (86)

where *m* is the size of the sample from which the maximum is derived.

Finally, the variance of the asymptotic distribution of the sample maximum is given by

$$\begin{array}{c} \text{Var}\left(X_{a}^{\text{max}}\right)=\frac{\pi^{2}}{6}{\left(\frac{1}{-\Phi^{-1}\left(\frac{1}{m}\right)}\right)}^{2}, \end{array}$$ (87)

where *m* is the size of the sample from which the maximum is derived.

For typical sample sizes *m* in high-dimensional spaces, the variance estimate (Eq 87) exceeds the variance of the sample maximum significantly. Using the fact that

$$-\Phi^{-1}\left(\frac{1}{m}\right)\dot{∼}\sqrt{2\text{log}\left(m\right)}$$

[24] and

$$\frac{1}{2\text{log}\left(m\right)}\leq {\left(\frac{1}{-\Phi^{-1}\left(\frac{1}{m}\right)}\right)}^{2},\;m\geq 2,$$

we can get a more accurate approximation of the variance with the following

$$\begin{array}{c} \begin{array}{cc} \sigma_{\text{max}}^{2}\left(m\right)=\text{Var}\left(X_{a}^{\text{max}}\right) & \approx \frac{\pi^{2}}{6}{\left(\frac{1}{\sqrt{2\text{log}\left(m\right)}}\right)}^{2}\\ & =\frac{\pi^{2}}{12\text{log}\left(m\right)}. \end{array} \end{array}$$ (88)

Therefore, the mean of the range of *m* iid standard normal random variables is given by

$$\begin{array}{c} \text{E}\left(X_{a}^{\text{max}}-X_{a}^{\text{min}}\right)=2\mu_{\text{max}}^{\left(1\right)}\left(m\right)=2[-\Phi^{-1}\left(\frac{1}{m}\right)-\frac{\gamma }{\Phi^{-1}\left(\frac{1}{m}\right)}], \end{array}$$ (89)

where *γ* is the Euler-Mascheroni constant.

It is well known that the sample extremes from the standard normal distribution are approximately uncorrelated for large sample size *m* [22]. This implies that we can approximate the variance of the range of *m* iid standard normal random variables with the following result

$$\begin{array}{c} \begin{array}{cc} \text{Var}\left(X_{a}^{\text{max}}-X_{a}^{\text{min}}\right) & \approx \text{Var}\left(X_{a}^{\text{max}}\right)+\text{Var}\left(X_{a}^{\text{min}}\right)\\ & =\sigma_{\text{max}}^{2}\left(m\right)+\sigma_{\text{min}}^{2}\left(m\right)\\ & =2\sigma_{\text{max}}^{2}\left(m\right)\\ & \approx 2\left(\frac{\pi^{2}}{12\text{log}\left(m\right)}\right)\\ & =\frac{\pi^{2}}{6\text{log}\left(m\right)}. \end{array} \end{array}$$ (90)

For the purpose of approximating the mean and variance of the max-min normalized distance distribution, we observe empirically that the formula for the median of the distribution of the attribute maximum (Eq 86) yields more accurate results. More precisely, the approximation of the expected maximum (Eq 85) overestimates the sample maximum slightly. The formula for the median of the sample maximum (Eq 86) provides a more accurate estimate of this sample extreme. Therefore, the following estimate for the mean of the attribute range will be used instead

$$\begin{array}{c} \text{E}\left(X_{a}^{\text{max}}-X_{a}^{\text{min}}\right)=2\mu_{\text{max}}^{\left(1\right)}\left(m\right)\approx 2[\frac{\text{log}\left(\text{log}\left(2\right)\right)}{\Phi^{-1}\left(\frac{1}{m}\right)}-\Phi^{-1}\left(\frac{1}{m}\right)], \end{array}$$ (91)

where *m* is the size of the sample from which extremes are derived.

**Fig 4:**
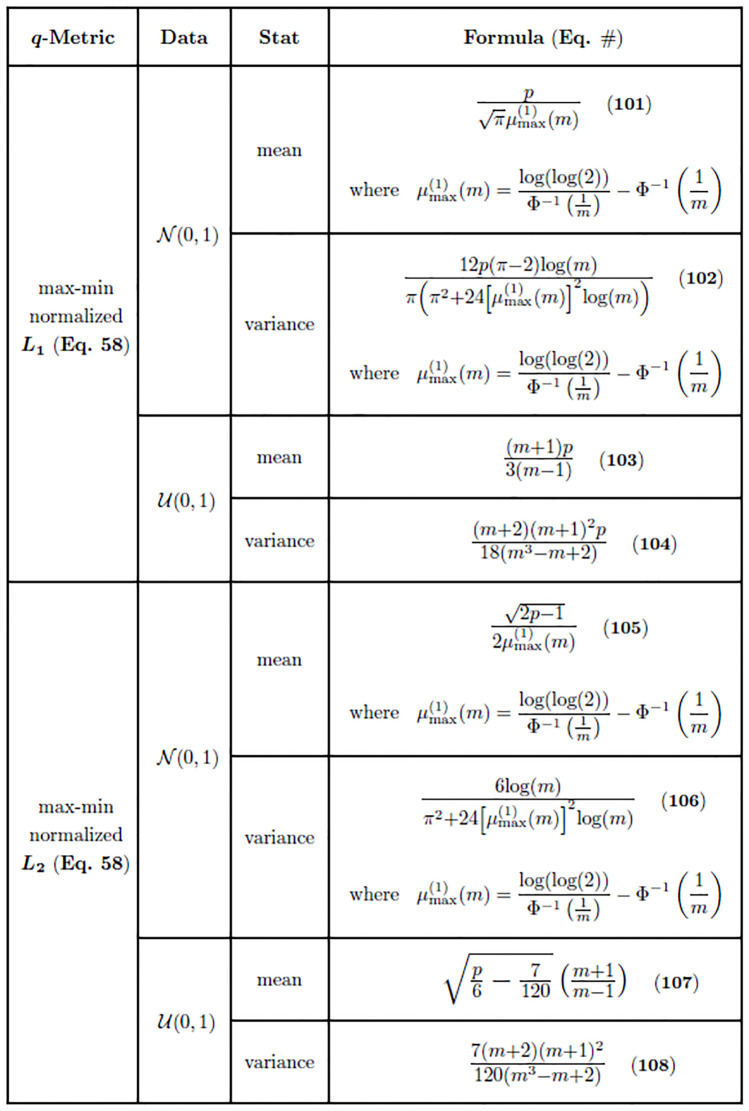
Asymptotic estimates of means and variances for the max-min normalized *L*_1_ and *L*_2_ distance distributions commonly used in Relief-based algorithms. Estimates for both standard normal (N(0,1)) and standard uniform (U(0,1)) data are given. The cumulative distribution function of the standard normal distribution is represented by Φ. Furthermore, $\mu_{\text{max}}^{\left(1\right)}\left(m\right)$ (Eq 86) is the asymptotic median of the sample maximum from *m* standard normal random samples.

We have already determined the mean and variance (Eq 30) for the *L*_*q*_ metric (Eq 1) on standard normal data. Using the expected value of the sample maximum (Eq 91), the variance of the sample maximum (Eq 90), and the general formulas for the mean and variance of the max-min normalized distance distribution (Eq 80), this leads us to the following asymptotic estimate for the distribution of the max-min normalized distances for standard normal data

$$\begin{array}{c} D_{ij}^{\left(q*\right)}\dot{∼}N\left(\frac{\mu_{D_{ij}^{\left(q\right)}}}{2\mu_{\text{max}}^{\left(1\right)}\left(m\right)},\frac{6\text{log}\left(m\right)\sigma_{D_{ij}^{\left(q\right)}}^{2}}{\pi^{2}+24{\left[\mu_{\text{max}}^{\left(1\right)}\left(m\right)\right]}^{2}\text{log}\left(m\right)}\right). \end{array}$$ (92)

where *m* is the size of the sample from which the maximum is derived, $\mu_{\text{max}}^{\left(1\right)}$ is the median of the sample maximum (Eq 86), $\mu_{D_{ij}^{\left(q\right)}}$ is the expected *L*_*q*_ pairwise distance (Eq 28), and $\sigma_{D_{ij}^{\left(q\right)}}^{2}$ is the variance of the *L*_*q*_ pairwise distance (Eq 29). The summary of moments of the max-min normalized *L*_*q*_ distance metric in standard normal data (Eq 92) is organized by metric, data type, statistic (mean or variance), and asymptotic formula (Fig 4).

#### 4.1.2 Standard uniform data

Standard uniform data does not have an even density function. Due to the simplicity of the density function, however, we can derive the distribution of the maximum and minimum of a sample of size *m* explicitly. Using the general forms of the distribution functions of the maximum (Eq 60) and minimum (Eq 63), we have the following distribution functions for standard uniform data

$$\begin{array}{c} F_{\text{max}}\left(x\right)=x^{m} \end{array}$$ (93)

and

$$\begin{array}{c} F_{\text{min}}\left(x\right)=1-{\left(1-x\right)}^{m}, \end{array}$$ (94)

where *m* is the size of the sample from which extremes are derived.

Using the general forms of the density functions of the maximum (Eq 61) and minimum (Eq 64), we have the following density functions for standard uniform data

$$\begin{array}{c} f_{\text{max}}\left(x\right)=mx^{m-1} \end{array}$$ (95)

and

$$\begin{array}{c} f_{\text{min}}\left(x\right)=m{\left(1-x\right)}^{m-1}, \end{array}$$ (96)

where *m* is the size of the sample from which extremes are derived.

Then the expected maximum and minimum are computed through straightforward integration as follows

$$\begin{array}{c} \begin{array}{cc} \text{E}\left(X_{a}^{\text{max}}\right)=\mu_{\text{max}}^{\left(1\right)}\left(m\right) & =\int_{0}^{1}xf_{\text{max}}\left(x\right)\text{d}x\\ & =\int_{0}^{1}x\left[mx^{m-1}\right]\text{d}x\\ & =\frac{m}{m+1} \end{array} \end{array}$$ (97)

and

$$\begin{array}{c} \begin{array}{cc} \text{E}\left(X_{a}^{\text{min}}\right)=\mu_{\text{min}}^{\left(1\right)}\left(m\right) & =\int_{0}^{1}xf_{\text{min}}\left(x\right)\text{d}x\\ & =\int_{0}^{1}x\left[m{\left(1-x\right)}^{m-1}\right]\text{d}x\\ & =\frac{1}{m+1}, \end{array} \end{array}$$ (98)

where *m* is the size of the sample from which extremes are derived.

We can compute the second moment about the origin of the sample range as follows

$$\begin{array}{c} \begin{array}{cc} \text{E}[{\left(X_{a}^{\text{max}}-X_{a}^{\text{min}}\right)}^{2}] & =\text{E}[{\left(X_{a}^{\text{max}}\right)}^{2}-2X_{a}^{\text{max}}X_{a}^{\text{min}}+{\left(X_{a}^{\text{min}}\right)}^{2}]\\ & =\text{E}\left[{\left(X_{a}^{\text{max}}\right)}^{2}\right]-2\text{E}\left(X_{a}^{\text{max}}\right)\text{E}\left(X_{a}^{\text{min}}\right)+\text{E}\left[{\left(X_{a}^{\text{min}}\right)}^{2}\right]\\ & =\mu_{\text{max}}^{\left(2\right)}\left(m\right)-2\mu_{\text{max}}^{\left(1\right)}\left(m\right)\mu_{\text{min}}^{\left(1\right)}\left(m\right)+\mu_{\text{min}}^{\left(2\right)}\left(m\right)\\ & =\int_{0}^{1}x^{2}\left[mx^{m-1}\right]\text{d}x-2\left(\frac{m}{m+1}\right)\left(\frac{1}{m+1}\right)\\ & \;+\int_{0}^{1}x^{2}\left[m{\left(1-x\right)}^{m-1}\right]\text{d}x\\ & =\frac{m}{m+2}-\frac{2m}{{\left(m+1\right)}^{2}}+\frac{2}{\left(m+1\right)\left(m+2\right)}\\ & =\frac{m^{3}-m+2}{\left(m+2\right){\left(m+1\right)}^{2}}, \end{array} \end{array}$$ (99)

where *m* is the size of the sample from which extremes are derived.

Using the general asymptotic distribution of max-min normalized distances for any data type (Eq 77) and the mean and variance (Eq 40) of the standard *L*_*q*_ distance metric (Eq 1), we have the following asymptotic estimate for the max-min normalized distance distribution for standard uniform data

$$\begin{array}{c} D_{ij}^{\left(q*\right)}\dot{∼}N\left(\frac{\left(m+1\right)\mu_{D_{ij}^{\left(q\right)}}}{m-1},\frac{\left(m+2\right){\left(m+1\right)}^{2}\sigma_{D_{ij}^{\left(q\right)}}^{2}}{m^{3}-m+2}\right), \end{array}$$ (100)

where *m* is the size of the sample from which extremes are derived, $\mu_{D_{ij}^{\left(q\right)}}$ is the expected value (Eq 38) of the *L*_*q*_ metric (Eq 1) in standard uniform data, and $\sigma_{D_{ij}^{\left(q\right)}}^{2}$ is the variance (Eq 39) of the *L*_*q*_ metric (Eq 1) in standard uniform data. The summary of moments of the max-min normalized *L*_*q*_ distance metric in standard uniform data (Eq 92) is organized by metric, data type, statistic (mean or variance), and asymptotic formula (Fig 4).

### 4.2 Range-Normalized Manhattan (*q* = 1)

Using the general asymptotic results for mean and variance of max-min normalized distances in standard normal and standard uniform data (Eqs 92 and 100) for any value of q∈N, we can substitute a particular value of *q* in order to determine a more specified distribution for the normalized distance (*D*^(q*)^, Eq 58). The following results are for the max-min normalized Manhattan (*q* = 1), *D*^(1*)^, metric for both standard normal and standard uniform data.

#### 4.2.1 Standard normal data

Substituting *q* = 1 into the asymptotic formula for the expected max-min normalized distance (Eq 92), we derive the expected normalized Manhattan distance in standard normal data as follows

$$\begin{array}{c} \begin{array}{cc} \text{E}\left(D_{ij}^{\left(1*\right)}\right) & =\frac{\mu_{D_{ij}^{\left(1\right)}}}{2\mu_{\text{max}}^{\left(1\right)}\left(m\right)}\\ & =\frac{p}{\sqrt{\pi }\mu_{\text{max}}^{\left(1\right)}\left(m\right)}, \end{array} \end{array}$$ (101)

where $\mu_{\text{max}}^{\left(1\right)}\left(m\right)$ is the expected attribute maximum (Eq 86), *m* is the size of the sample from which the maximum is derived, and *p* is the total number of attributes.

Similarly, the variance of $D_{ij}^{\left(1*\right)}$ is given by

$$\begin{array}{c} \begin{array}{cc} \text{Var}\left(D_{ij}^{\left(1*\right)}\right) & =\frac{6\text{log}\left(m\right)\sigma_{D_{ij}^{\left(1\right)}}^{2}}{\pi^{2}+24{\left[\mu_{\text{max}}^{\left(1\right)}\right]}^{2}\text{log}\left(m\right)}\\ & =\frac{12p\left(\pi -2\right)\text{log}\left(m\right)}{\pi \left(\pi^{2}+24{\left[\mu_{\text{max}}^{\left(1\right)}\right]}^{2}\text{log}\left(m\right)\right)}, \end{array} \end{array}$$ (102)

where $\mu_{\text{max}}^{\left(1\right)}\left(m\right)$ is the expected attribute maximum (Eq 86), *m* is the size of the sample from which the maximum is derived, and *p* is the total number of attributes. Similar to the variance of the standard Manhattan distance, the variance of the max-min normalized Manhattan distance is on the order of *p* for fixed instance dimension *m*. For fixed *p*, the variance (Eq 102) vanishes as *m* grows without bound. If we fix *m*, the same variance increases monotonically with increasing *p*. The summary of moments derived in this section (Eqs 101 and 102) is organized by metric, data type, statistic (mean or variance), and asymptotic formula (Fig 4).

#### 4.2.2 Standard uniform data

Substituting *q* = 1 into the asymptotic formula for the expected max-min pairwise distance (Eq 100), we derive the expected normalized Manhattan distance in standard uniform data as

$$\begin{array}{c} \begin{array}{cc} \text{E}\left(D_{ij}^{\left(1*\right)}\right) & =\frac{\left(m+1\right)\mu_{D_{ij}^{\left(1\right)}}}{m-1}\\ & =\frac{\left(m+1\right)p}{3\left(m-1\right)}, \end{array} \end{array}$$ (103)

where *m* is the size of the sample from which extremes are derived and *p* is the total number attributes.

Similarly, the variance of $D_{ij}^{\left(1*\right)}$ is given by

$$\begin{array}{c} \begin{array}{cc} \text{Var}\left(D_{ij}^{\left(1*\right)}\right) & =\frac{\left(m+2\right){\left(m+1\right)}^{2}\sigma_{D_{ij}^{\left(1\right)}}^{2}}{m^{3}-m+2}\\ & =\frac{\left(m+2\right){\left(m+1\right)}^{2}p}{18\left(m^{3}-m+2\right)}, \end{array} \end{array}$$ (104)

where *m* is the size of the sample from which extremes are derived and *p* is the total number of attributes. Interestingly, the variance of the max-min normalized Manhattan distance in standard uniform data approaches *p*/18 as *m* increases without bound for a fixed number of attributes *p*. This is the same asymptotic value to which the variance of the standard Manhattan distance (Eq 43) converges. Therefore, large sample sizes make the variance of the normalized Manhattan distance approach the variance of the standard Manhattan distance in standard uniform data. The summary of moments derived in this section (Eqs 103 and 104) is organized by metric, data type, statistic (mean or variance), and asymptotic formula (Fig 4).

### 4.3 Range-Normalized Euclidean (*q* = 2)

Analogous to the previous section, we use the asymptotic moment estimates for the max-min normalized metric (*D*^(q*)^, Eq 58) for standard normal (Eq 92) and standard uniform (Eq 100) data but specific to a range-normalized Euclidean metric (*q* = 2).

#### 4.3.1 Standard normal data

Substituting *q* = 2 into the asymptotic formula for the expected max-min normalized pairwise distance (Eq 92), we derive the expected normalized Euclidean distance in standard normal data as

$$\begin{array}{c} \begin{array}{cc} \text{E}\left(D_{ij}^{\left(2*\right)}\right) & =\frac{\mu^{D_{ij}^{\left(2\right)}}}{2\mu_{\text{max}}^{\left(1\right)}\left(m\right)}\\ & =\frac{\sqrt{2p-1}}{2\mu_{\text{max}}^{\left(1\right)}\left(m\right)}, \end{array} \end{array}$$ (105)

where $\mu_{\text{max}}^{\left(1\right)}\left(m\right)$ is the expected attribute maximum (Eq 86), *m* is the size of the sample from which the maximum is derived, and *p* is the total number of attributes.

Similarly, the variance of $D_{ij}^{\left(2*\right)}$ is given by

$$\begin{array}{c} \begin{array}{cc} \text{Var}\left(D_{ij}^{\left(2*\right)}\right) & =\frac{6\text{log}\left(m\right)\sigma_{D_{ij}^{\left(2\right)}}^{2}}{\pi^{2}+24{\left[\mu_{\text{max}}^{\left(1\right)}\left(m\right)\right]}^{2}\text{log}\left(m\right)}\\ & =\frac{6\text{log}\left(m\right)}{\pi^{2}+24{\left[\mu_{\text{max}}^{\left(1\right)}\left(m\right)\right]}^{2}\text{log}\left(m\right)}, \end{array} \end{array}$$ (106)

where $\mu_{\text{max}}^{\left(1\right)}\left(m\right)$ is the expected attribute maximum (Eq 86) and *m* is the size of the sample from which the maximum is derived. It is interesting to note that the variance (Eq 106) vanishes as the sample size *m* increases without bound, which means that all distances will be tightly clustered about the mean (Eq 105). This is different than the variance of the standard *L*_2_ metric (Eq 52), which is asymptotically equal to 1. This could imply that any two pairwise distances computed with the max-min normalized Euclidean metric in a large sample space *m* may be indistinguishable, which is another curse of dimensionality. The summary of moments derived in this section (Eqs 105 and 106) is organized by metric, data type, statistic (mean or variance), and asymptotic formula (Fig 4).

#### 4.3.2 Standard uniform data

Substituting *q* = 2 into the asymptotic formula for the expected max-min normalized pairwise distance (Eq 100), we derive the expected normalized Euclidean distance in standard uniform data as

$$\begin{array}{c} \begin{array}{cc} \text{E}\left(D_{ij}^{\left(2*\right)}\right) & =\frac{\left(m+1\right)\mu_{D_{ij}^{\left(2\right)}}}{m-1}\\ & =\sqrt{\frac{p}{6}-\frac{7}{120}}\left(\frac{m+1}{m-1}\right). \end{array} \end{array}$$ (107)

where *m* is the size of the sample from which extremes are derived and *p* is the total number of attributes.

Similarly, the variance of $D_{ij}^{\left(2*\right)}$ is given by

$$\begin{array}{c} \begin{array}{cc} \text{Var}\left(D_{ij}^{\left(2*\right)}\right) & =\frac{\left(m+2\right){\left(m+1\right)}^{2}\sigma_{D_{ij}^{\left(2\right)}}^{2}}{m^{3}-m+2}\\ & =\frac{7\left(m+2\right){\left(m+1\right)}^{2}}{120\left(m^{3}-m+2\right)}. \end{array} \end{array}$$ (108)

where *m* is the size of the sample from which extremes are derived. Similar to the variance of max-min normalized Manhattan distances in standard uniform data (Eq 104), the variance of normalized Euclidean distances approaches the variance of the standard Euclidean distances in uniform data (Eq 55) as *m* increases without bound. That is, the variance of the max-min normalized Euclidean distance (Eq 108) approaches 7/120 as *m* grows larger. The summary of moments derived in this section (Eqs 107 and 108) is organized by metric, data type, statistic (mean or variance), and asymptotic formula (Fig 4).

We summarize moment estimates in figures (Figs 2–4) that contain all of our asymptotic results for both standard and max-min normalized *L*_*q*_ metrics in each data type we have considered. This includes our most general results for any combination of sample size *m*, number of attributes *p*, type of metric *L*_*q*_, and data type (Fig 2). From these more general derivations, we show the results of the standard *L*_1_ and *L*_2_ metrics for any combination of sample size *m*, number of attributes *p*, and data type (Fig 3). Our last set of summarized results show asymptotics for the max-min normalized *L*_1_ and *L*_2_ metrics for any combination of sample size *m*, number of attributes *p*, and data type (Fig 4). For both standard and max-min normalized *L*_2_ metrics (Figs 3 and 4), the low-dimensional improved estimates of sample means (Eqs 53 and 56) are used because they perform well at both low and high attribute dimension *p*.

In the next section, we make a transition into discrete GWAS data. We will discuss some commonly known metrics and then a relatively new metric, which will lead us into novel asymptotic results for this data type.

## 5 GWAS distance distributions

Genome-wide association study (GWAS) data consists of single nucleotide polymorphisms (SNPs), which are inherited nucleotide changes at loci along the DNA. Each SNP has two possible nucleotide alleles: the minor allele, which is the less frequent nucleotide in the population, and the common allele. The attribute/feature corresponding to each SNP is typically represented as a three-state genotype: homozygous for the minor allele, heterozygous or homozygous for the common allele. Feature selection in GWAS is typically concerned with finding main effect or interacting SNPs that are associated with disease susceptibility [25]. The similarity or distance between individuals in the SNP space is routinely calculated in GWAS for principal component analysis but is also calculated for nearest-neighbor feature selection.

For our asymptotic analysis formalism, consider a GWAS data set with the following encoding based on minor allele frequency

$$\begin{array}{c} X_{ia}=\{\begin{array}{cc} 0 & \;\text{if}\;\text{there}\;\text{are}\;\text{no}\;\text{minor}\;\text{alleles}\;\text{at}\;\text{locus}\;a,\\ 1 & \;\text{if}\;\text{there}\;\text{is}\;\text{1}\;\text{minor}\;\text{allele}\;\text{at}\;\text{locus}\;a,\\ 2 & \;\text{if}\;\text{there}\;\text{are}\;\text{2}\;\text{minor}\;\text{alleles}\;\text{at}\;\text{locus}\;a. \end{array} \end{array}$$ (109)

For random GWAS data sets, we can think *X*_*ia*_ as the number of successes in two Bernoulli trials. That is, $X_{ia}∼B\left(2,f_{a}\right)$ where *f*_*a*_ is the probability of success. The success probability *f*_*a*_ is the probability of a minor allele occurring at *a*. Furthermore, the minor allele probabilities are assumed to be independent and identically distributed according to U(l,u), where *l* and *u* are the lower and upper bounds, respectively, of the sampling distribution’s support.

Two commonly known types of distance metrics for GWAS data are the Genotype Mismatch (GM) and Allele Mismatch (AM) metrics. The GM and AM metrics are defined by

$$\begin{array}{c} {\text{d}}_{ij}^{\text{GM}}\left(a\right)=\{\begin{array}{cc} 0 & \;\text{if}\;X_{ia}\neq X_{ja},\\ 1 & \;\text{otherwise} \end{array} \end{array}$$ (110)

and

$$\begin{array}{c} {\text{d}}_{ij}^{\text{AM}}\left(a\right)=\frac{1}{2}|X_{ia}-X_{ja}|. \end{array}$$ (111)

**Fig 5:**
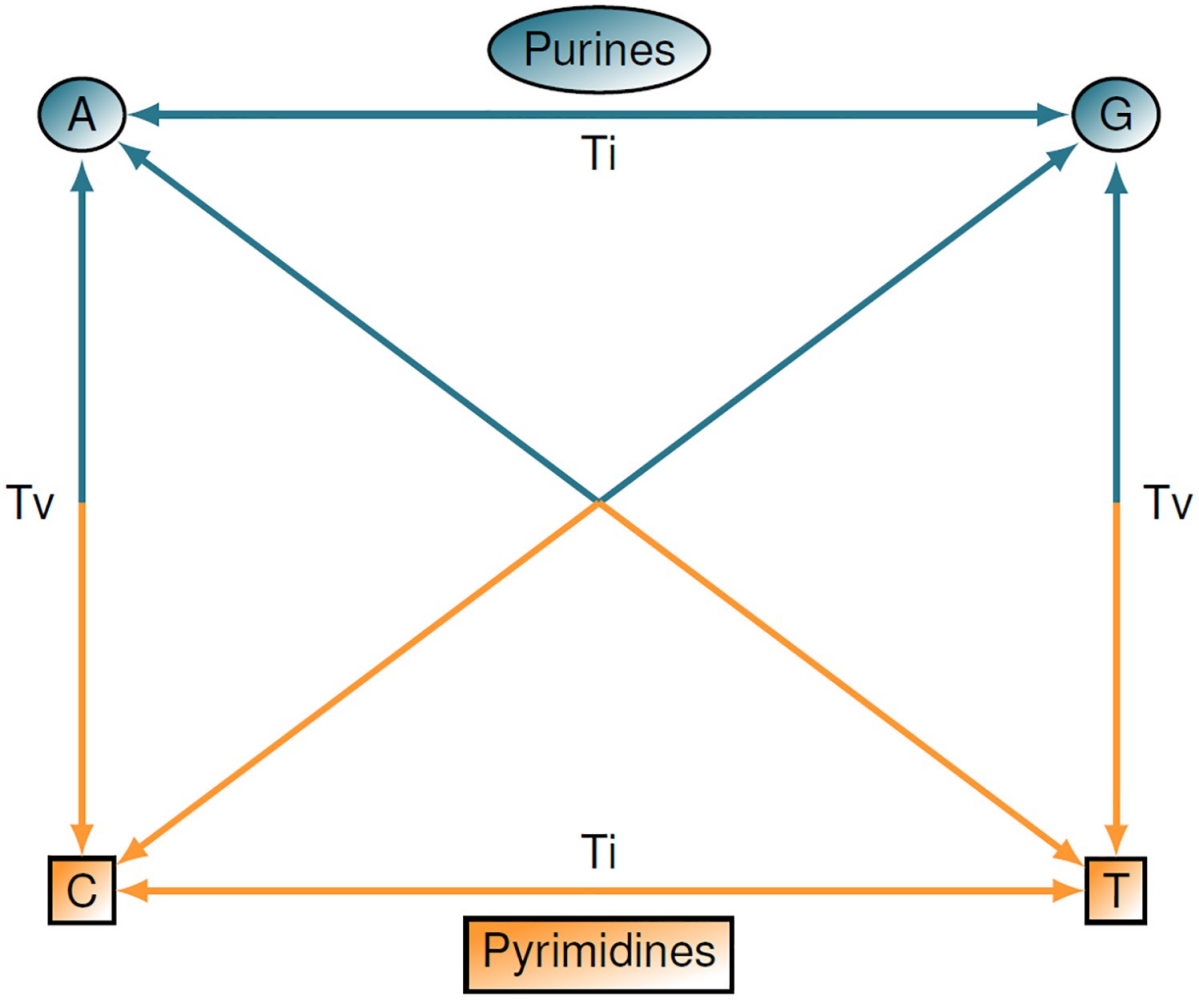
Purines (A and G) and pyrimidines (C and T) are shown. Transitions occur when a mutation involves purine-to-purine or pyrimidine-to-pyrimidine insertion. Transversions occur when a purine-to-pyrimidine or pyrimidine-to-purine insertion happens, which is a more extreme case. There are visibly more possibilities for transversions to occur than there are transitions, but there are about twice as many transitions in real data.

More informative metrics may include differences at the nucleotide level for each allele by considering differences in the rates of transition and transversion mutations (Fig 5). One such discrete metric that accounts for transitions (Ti) and transversions (Tv) was introduced in [7] and can be written as

$$\begin{array}{c} {\text{d}}_{ij}^{\text{TiTv}}\left(a\right)=\{\begin{array}{cc} 0 & \;\text{if}\;X_{ia}=X_{ja}\;\text{and}\;\text{Ti/Tv},\\ 1/4 & \;\text{if}\;|X_{ia}-X_{ja}|=1\;\text{and}\;\text{Ti},\\ 1/2 & \;\text{if}\;|X_{ia}-X_{ja}|=1\;\text{and}\;\text{Tv},\\ 3/4 & \;\text{if}\;|X_{ia}-X_{ja}|=2\;\text{and}\;\text{Ti},\\ 1 & \;\text{if}\;|X_{ia}-X_{ja}|=2\;\text{and}\;\text{Tv}. \end{array} \end{array}$$ (112)

With these GWAS distance metrics, we then compute the pairwise distance between two instances i,j∈I with

$$\begin{array}{c} D_{ij}^{\text{GM}}\left(a\right)=\sum_{a\in A}{\text{d}}_{ij}^{\text{GM}}\left(a\right), \end{array}$$ (113)

$$\begin{array}{c} D_{ij}^{\text{AM}}\left(a\right)=\sum_{a\in A}{\text{d}}_{ij}^{\text{AM}}\left(a\right),\;\text{or} \end{array}$$ (114)

$$\begin{array}{c} D_{ij}^{\text{TiTv}}\left(a\right)=\sum_{a\in A}{\text{d}}_{ij}^{\text{TiTv}}\left(a\right). \end{array}$$ (115)

Assuming that all data entries *X*_*ia*_ are independent and identically distributed, we have already shown that the distribution of pairwise distances is asymptotically normal regardless of data distribution and value of *q*. Therefore, it follows that the distance distributions induced by each of the GWAS metrics (Eqs 110–112) are asymptotically normal. We illustrate Gaussian convergence in the case of GM (S4 Fig in S1 File), AM (S5 Fig in S1 File), and TiTv (S6 Fig in S1 File). With this Gaussian limiting behavior, we will proceed by deriving the mean and variance for each distance distribution induced by these three GWAS metrics.

### 5.1 GM distance distribution

The simplest distance metric in nearest-neighbor feature selection in GWAS data is the genotype-mismatch (GM) distance metric (Eq 113). The GM attribute diff (Eq 110) indicates only whether two genotypes are the same or not. There are many ways two genotypes could differ, but this metric does not record this information. We will now derive the moments for the GM distance (Eq 113), which are sufficient for defining its corresponding asymptotic distribution.

The expected value of the GM attribute diff metric (Eq 110) is given by the following

$$\begin{array}{c} \begin{array}{cc} \text{E}[{\text{d}}_{ij}^{\text{GM}}\left(a\right)] & =\sum_{k=0}^{1}k\cdot \text{P}[{\text{d}}_{ij}^{\text{GM}}\left(a\right)=k]\\ & =0\cdot \text{P}[{\text{d}}_{ij}^{\text{GM}}\left(a\right)=0]+1\cdot \text{P}[{\text{d}}_{ij}^{\text{GM}}\left(a\right)=1]\\ & =\text{P}[{\text{d}}_{ij}^{\text{GM}}\left(a\right)=1]\\ & =2\text{P}\left[X_{ia}=0,X_{ja}=1\right]+2\text{P}\left[X_{ia}=1,X_{ja}=2\right]+2\text{P}\left[X_{ia}=0,X_{ja}=2\right]\\ & =4{\left(1-f_{a}\right)}^{3}f_{a}+4\left(1-f_{a}\right)f_{a}^{3}+2{\left(1-f_{a}\right)}^{2}f_{a}^{2}\\ & =2[2{\left(1-f_{a}\right)}^{3}f_{a}+2\left(1-f_{a}\right)f_{a}^{3}+{\left(1-f_{a}\right)}^{2}f_{a}^{2}]\\ & =2F^{\text{GM}}\left(a\right), \end{array} \end{array}$$ (116)

where $F^{\text{GM}}\left(a\right)=2{\left(1-f_{a}\right)}^{3}f_{a}+2\left(1-f_{a}\right)f_{a}^{3}+{\left(1-f_{a}\right)}^{2}f_{a}^{2}$ and *f*_*a*_ is the probability of a minor allele occurring at locus *a*.

Then the expected pairwise GM distance between instances i,j∈I is given by

$$\begin{array}{c} \begin{array}{cc} \text{E}\left(D_{ij}^{\text{GM}}\right) & =\text{E}\left(\sum_{a\in A}{\text{d}}_{ij}^{\text{GM}}\left(a\right)\right)\\ & =\sum_{a\in A}\text{E}[{\text{d}}_{ij}^{\text{GM}}\left(a\right)]\\ & =2\sum_{a\in A}F^{\text{GM}}\left(a\right), \end{array} \end{array}$$ (117)

where $F^{\text{GM}}\left(a\right)=2{\left(1-f_{a}\right)}^{3}f_{a}+2\left(1-f_{a}\right)f_{a}^{3}+{\left(1-f_{a}\right)}^{2}f_{a}^{2}$ and *f*_*a*_ is the probability of a minor allele occurring at locus *a*. We see that the expected GM pairwise distance (Eq 117) relies only on the minor allele probabilities *f*_*a*_ for all a∈A. In real data, we can easily determine these probabilities by dividing the total number of minor alleles at locus *a* by the twice the number of instances *m*. To be more explicit, this is just

$$f_{a}=\frac{1}{2m}\sum_{i\in I}X_{ia},\;\;\text{for}\;\text{all}\;a\in A,$$

where *m* is the number of instances (or sample size). This is because each instance has two alleles, the minor and major alleles, at each locus. Therefore, the total number of alleles at locus *a* is 2*m*.

The second moment about the origin for the GM distance is computed as follows

$$\begin{array}{c} \begin{array}{cc} \text{E}[{\left(D_{ij}^{\text{GM}}\right)}^{2}] & =\text{E}[{\left(\sum_{a\in A}{\text{d}}_{ij}^{\text{GM}}\left(a\right)\right)}^{2}]\\ & =\text{E}[\sum_{a\in A}{\left({\text{d}}_{ij}^{\text{GM}}\left(a\right)\right)}^{2}]+2\text{E}[\sum_{r\in A}\sum_{s\leq r-1}{\text{d}}_{ij}^{\text{GM}}\left(r\right)\cdot {\text{d}}_{ij}^{\text{GM}}\left(s\right)]\\ & =\sum_{a\in A}\left(\sum_{k=0}^{1}k^{2}\cdot \text{P}[{\text{d}}_{ij}^{\text{GM}}\left(a\right)=k]\right)\\ & +2\sum_{a\in A}\sum_{s\leq r-1}\left(\sum_{k=0}^{1}k\cdot \text{P}[{\text{d}}_{ij}^{\text{GM}}\left(r\right)=k]\right)\cdot \left(\sum_{k=0}^{1}k\cdot \text{P}[{\text{d}}_{ij}^{\text{GM}}\left(s\right)=k]\right)\\ & =2\sum_{a\in A}F^{\text{GM}}\left(a\right)+8\sum_{r\in A}\sum_{s\leq r-1}\prod_{\lambda \in \{r,s\}}F^{\text{GM}}\left(\lambda \right), \end{array} \end{array}$$ (118)

where $F^{\text{GM}}\left(a\right)=2{\left(1-f_{a}\right)}^{3}f_{a}+2\left(1-f_{a}\right)f_{a}^{3}+{\left(1-f_{a}\right)}^{2}f_{a}^{2}$ and *f*_*a*_ is the probability of a minor allele occurring at locus *a*.

Using the first (Eq 117) and second (Eq 118) raw moments of the GM distance, the variance is given by

$$\begin{array}{c} \begin{array}{cc} \text{Var}\left(D_{ij}^{\text{GM}}\right) & =\text{E}[{\left(D_{ij}^{\text{GM}}\right)}^{2}]-{\left[\text{E}\left(D_{ij}^{\text{GM}}\right)\right]}^{2}\\ & =2\sum_{a\in A}F^{\text{GM}}\left(a\right)+8\sum_{r\in A}\sum_{s\leq r-1}\prod_{\lambda \in \{r,s\}}F^{\text{GM}}\left(\lambda \right)-4{\left(\sum_{a\in A}F^{\text{GM}}\left(a\right)\right)}^{2}\\ & =2\sum_{a\in A}F^{\text{GM}}\left(a\right)-4\sum_{a\in A}{\left[F^{\text{GM}}\left(a\right)\right]}^{2}\\ & =2\sum_{a\in A}F^{\text{GM}}\left(a\right)\left[1-2F^{\text{GM}}\left(a\right)\right], \end{array} \end{array}$$ (119)

where $F^{\text{GM}}\left(a\right)=2{\left(1-f_{a}\right)}^{3}f_{a}+2\left(1-f_{a}\right)f_{a}^{3}+{\left(1-f_{a}\right)}^{2}f_{a}^{2}$ and *f*_*a*_ is the probability of a minor allele occurring at locus *a*. Hence, the variance of the asymptotic GM distance distribution also just depends on the minor allele probabilities *f*_*a*_ for all a∈A. This implies that the limiting GM distance distribution is fully determined by the minor allele probabilities, which are known in real data.

With the mean and variance estimates (Eqs 117 and 119), the asymptotic GM distance distribution is given by the following

$$\begin{array}{c} D_{ij}^{\text{GM}}\dot{∼}N\left(2\sum_{a\in A}F^{\text{GM}}\left(a\right),2\sum_{a\in A}F^{\text{GM}}\left(a\right)\left[1-2F^{\text{GM}}\left(a\right)\right]\right), \end{array}$$ (120)

where $F^{\text{GM}}\left(a\right)=2{\left(1-f_{a}\right)}^{3}f_{a}+2\left(1-f_{a}\right)f_{a}^{3}+{\left(1-f_{a}\right)}^{2}f_{a}^{2}$ and *f*_*a*_ is the probability of a minor allele occurring at locus *a*. This GM distribution holds for random independent GWAS data with minor allele probabilities *f*_*a*_ and binomial samples $X_{ia}∼B\left(2,f_{a}\right)$ for all a∈A. Next we consider the distance distribution for an AM metric, which incorporates differences at the allele level and contains more information than genotype differences.

### 5.2 AM distance distribution

As we have mentioned previously, the AM attribute diff metric (Eq 111) is slightly more dynamic than the GM metric because the AM metric accounts for differences between the alleles of two genotypes. In this section, we derive moments of the AM distance metric (Eq 114) that adequately define its corresponding asymptotic distribution.

The expected value of the AM attribute diff metric (Eq 111) is given by the following

$$\begin{array}{c} \begin{array}{cc} \text{E}[{\text{d}}_{ij}^{\text{AM}}\left(a\right)] & =\sum_{k\in D}k\cdot \text{P}[{\text{d}}_{ij}^{\text{AM}}\left(a\right)=k]\\ & =0\cdot \text{P}[{\text{d}}_{ij}^{\text{AM}}\left(a\right)=0]+\frac{1}{2}\cdot \text{P}[{\text{d}}_{ij}^{\text{AM}}\left(a\right)=\frac{1}{2}]+1\cdot \text{P}[{\text{d}}_{ij}^{\text{AM}}\left(a\right)=1]\\ & =\frac{1}{2}\left(2\text{P}[X_{ia}=0,X_{ja}=1]+2\text{P}[X_{ia}=1,X_{ja}=2]\right)\\ & +2\text{P}[X_{ia}=0,X_{ja}=2]\\ & =\text{P}[X_{ia}=0,X_{ja}=1]+\text{P}[X_{ia}=1,X_{ja}=2]+2\text{P}[X_{ia}=0,X_{ja}=2]\\ & =2{\left(1-f_{a}\right)}^{3}f_{a}+2\left(1-f_{a}\right)f_{a}^{3}+2{\left(1-f_{a}\right)}^{2}f_{a}^{2}\\ & =2[{\left(1-f_{a}\right)}^{3}f_{a}+\left(1-f_{a}\right)f_{a}^{3}+{\left(1-f_{a}\right)}^{2}f_{a}^{2}]\\ & =2F^{\text{AM}}\left(a\right), \end{array} \end{array}$$ (121)

where $F^{\text{AM}}\left(a\right)={\left(1-f_{a}\right)}^{3}f_{a}+\left(1-f_{a}\right)f_{a}^{3}+{\left(1-f_{a}\right)}^{2}f_{a}^{2}$, D={0,1/2,1}, and *f*_*a*_ is the probability of a minor allele occurring at locus *a*.

Using the expected AM attribute diff (Eq 121), the expected pairwise AM distance (Eq 114) between instances i,j∈I is given by

$$\begin{array}{c} \begin{array}{cc} \text{E}\left(D_{ij}^{\text{AM}}\right) & =\text{E}\left(\sum_{a\in A}{\text{d}}_{ij}^{\text{AM}}\left(a\right)\right)\\ & =\sum_{a\in A}\text{E}[{\text{d}}_{ij}^{\text{AM}}\left(a\right)]\\ & =2\sum_{a\in A}F^{\text{AM}}\left(a\right). \end{array} \end{array}$$ (122)

where $F^{\text{AM}}\left(a\right)={\left(1-f_{a}\right)}^{3}f_{a}+\left(1-f_{a}\right)f_{a}^{3}+{\left(1-f_{a}\right)}^{2}f_{a}^{2}$ and *f*_*a*_ is the probability of a minor allele occurring at locus *a*. Similar to GM distances, the expected AM distance (Eq 122) depends only on the minor allele probabilities *f*_*a*_ for all a∈A. This is to be expected because, although the AM metric is more informative, it still only accounts for simple differences between nucleotides of two instances i,j∈I at some locus *a*.

The second moment about the origin for the AM distance is computed as follows

$$\begin{array}{c} \begin{array}{cc} \text{E}[{\left(D_{ij}^{\text{AM}}\right)}^{2}] & =\text{E}[{\left(\sum_{a\in A}{\text{d}}_{ij}^{\text{AM}}\left(a\right)\right)}^{2}]\\ & =\text{E}[\sum_{a\in A}{\left({\text{d}}_{ij}^{\text{AM}}\left(a\right)\right)}^{2}]+2\text{E}[\sum_{r\in A}\sum_{s\leq r-1}{\text{d}}_{ij}^{\text{AM}}\left(r\right)\cdot {\text{d}}_{ij}^{\text{AM}}\left(s\right)]\\ & =\sum_{a\in A}\left(\sum_{k\in D}k^{2}\cdot \text{P}[{\text{d}}_{ij}^{\text{AM}}\left(a\right)=k]\right)\\ & +2\sum_{a\in A}\sum_{s\leq r-1}\left(\sum_{k\in D}k\cdot \text{P}[{\text{d}}_{ij}^{\text{AM}}\left(r\right)=k]\right)\cdot \left(\sum_{k\in D}k\cdot \text{P}[{\text{d}}_{ij}^{\text{AM}}\left(s\right)=k]\right)\\ & =\sum_{a\in A}G^{\text{AM}}\left(a\right)+8\sum_{r\in A}\sum_{s\leq r-1}\prod_{\lambda \in \{r,s\}}F^{\text{AM}}\left(\lambda \right), \end{array} \end{array}$$ (123)

where $G^{\text{AM}}\left(a\right)={\left(1-f_{a}\right)}^{3}f_{a}+f_{a}^{3}\left(1-f_{a}\right)+2{\left(1-f_{a}\right)}^{2}f_{a}^{2}$, $F^{\text{AM}}\left(\lambda \right)={\left(1-f_{\lambda }\right)}^{3}f_{\lambda }+f_{\lambda }^{3}\left(1-f_{\lambda }\right)+{\left(1-f_{\lambda }\right)}^{2}f_{\lambda }^{2}$, and *f*_*a*_ is the probability of a minor allele occurring at locus *a*.

Using the first (Eq 122) and second (Eq 123) raw moments of the asymptotic AM distance distribution, the variance is given by

$$\begin{array}{c} \begin{array}{cc} \text{Var}\left(D_{ij}^{\text{AM}}\right) & =\text{E}[{\left(D_{ij}^{\text{AM}}\right)}^{2}]-{\left[\text{E}\left(D_{ij}^{\text{AM}}\right)\right]}^{2}\\ & =\sum_{a\in A}G^{\text{AM}}\left(a\right)+8\sum_{r\in A}\sum_{s\leq r-1}\prod_{\lambda \in \{r,s\}}F^{\text{AM}}\left(\lambda \right)-4{\left(\sum_{a\in A}F^{\text{AM}}\left(a\right)\right)}^{2}\\ & =\sum_{a\in A}G^{\text{AM}}\left(a\right)-4\sum_{a\in A}{\left[F^{\text{AM}}\left(a\right)\right]}^{2}\\ & =\sum_{a\in A}\left(G^{\text{AM}}\left(a\right)-4{\left[F^{\text{AM}}\left(a\right)\right]}^{2}\right), \end{array} \end{array}$$ (124)

where $G^{\text{AM}}\left(a\right)={\left(1-f_{a}\right)}^{3}f_{a}+f_{a}^{3}\left(1-f_{a}\right)+2{\left(1-f_{a}\right)}^{2}f_{a}^{2}$, $F^{\text{AM}}\left(a\right)={\left(1-f_{a}\right)}^{3}f_{a}+f_{a}^{3}\left(1-f_{a}\right)+{\left(1-f_{a}\right)}^{2}f_{a}^{2}$, and *f*_*a*_ is the probability of a minor allele occurring at locus *a*. Similar to the mean (Eq 122), the variance just depends on minor allele probabilities *f*_*a*_ for all a∈A.

With the mean (Eq 122) and variance (Eq 124) estimates of AM distances, the asymptotic AM distance distribution is given by the following

$$\begin{array}{c} D_{ij}^{\text{AM}}\dot{∼}N\left(2\sum_{a\in A}F^{\text{AM}}\left(a\right),\sum_{a\in A}\left(G^{\text{AM}}\left(a\right)-4{\left[F^{\text{AM}}\left(a\right)\right]}^{2}\right)\right), \end{array}$$ (125)

where $G^{\text{AM}}\left(a\right)={\left(1-f_{a}\right)}^{3}f_{a}+f_{a}^{3}\left(1-f_{a}\right)+2{\left(1-f_{a}\right)}^{2}f_{a}^{2}$, $F\left(a\right)={\left(1-f_{a}\right)}^{3}f_{a}+f_{a}^{3}\left(1-f_{a}\right)+{\left(1-f_{a}\right)}^{2}f_{a}^{2}$, and *f*_*a*_ is the probability of a minor allele occurring at locus *a*.

This concludes our analysis of the AM metric in GWAS data when the independence assumption holds for minor allele probabilities *f*_*a*_ and binomial samples $B\left(2,f_{a}\right)$ for all a∈A. In the next section, we derive more complex asymptotic results for the TiTv distance metric (Eq 115).

### 5.3 TiTv distance distribution

The TiTv metric allows for one to account for both genotype mismatch, allele mismatch, transition, and transversion. However, this added dimension of information requires knowledge of the nucleotide makeup at a particular locus. A sufficient condition to compute the TiTv metric between instances i,j∈I is that we know whether the nucleotides associated with a particular locus *a* are both purines (PuPu), purine and pyrimidine (PuPy), or both pyrimidines (PyPy). We illustrate all possibilities for transitions and transversions in a diagram (Fig 5). Purines (A and G) and pyrimidines (C and T) are shown at the top and bottom, respectively. Transitions occur in the cases of PuPu and PyPy, while transversion occurs only with PuPy encoding.

This additional encoding is always given in a particular GWAS data set, which leads us to consider the probabilities of PuPu, PuPy, and PyPy. These will be necessary to determine asymptotics for the TiTv distance metric. Let *γ*_0_, *γ*_1_, and *γ*_2_ denote the probabilities of PuPu, PuPy, and PyPy, respectively, for the *p* loci of data matrix *X*. In real data, there are approximately twice as many transitions as there are transversions. That is, the probability of a transition P(Ti) is approximately twice the probability of transversion P(Tv). It is likely that any particular data set will not satisfy this criterion exactly. In this general case, we have P(Ti) being equal to some multiple *η* times P(Tv). In order to enforce this general constraint in simulated data, we define the following set of equalities

$$\gamma_{0}+\gamma_{1}+\gamma_{2}=1,$$ (126)

P(Ti)-ηP(Tv)=0. (127)

The sum-to-one constraint (Eq 126) is natural in this context because there are only three possible genotype encodings at a particular locus, which are PuPu, PuPy, and PyPy. Solving the Ti/Tv ratio constraint (Eq 127) for *η* gives

$$\eta =\frac{\text{P}\left(\text{Ti}\right)}{\text{P}\left(\text{Tv}\right)},$$

which is easily computed in a real data set by dividing the fraction of Ti out of the total *p* loci by the fraction of Tv out of the total *p* loci. We will use the simplified notation *η* = Ti/Tv to represent this factor for the remainder of this work.

Using this PuPu, PuPy, and PyPy encoding, the probability of a transversion occurring at any fixed locus *a* is given by the following

$$\begin{array}{c} \begin{array}{cc} \text{P}\left(\text{Tv}\right) & =\gamma_{1}. \end{array} \end{array}$$ (128)

Using the sum-to-one constraint (Eqs 126) and the probability of transversion (Eq 127), the probability of a transition occuring at locus *a* is computed as follows

$$\begin{array}{c} \begin{array}{cc} \text{P}\left(\text{Ti}\right) & =\gamma_{0}+\gamma_{2}. \end{array} \end{array}$$ (129)

Also using the sum-to-one constraint (Eq 126) and the Ti/Tv ratio constraint (Eq 127), it is clear that we have $\text{P}\left(\text{Tv}\right)=\frac{1}{\eta +1}$ and $\text{P}\left(\text{Ti}\right)=\frac{\eta }{\eta +1}$. Without loss of generality, we then sample

$$\begin{array}{c} \gamma_{0}∼U\left(\varepsilon ,\frac{\eta }{\eta +1}-\varepsilon \right), \end{array}$$ (130)

where *ε* is some small positive real number.

Then it immediately follows that we have

$$\begin{array}{c} \gamma_{2}=\frac{\eta }{\eta +1}-\gamma_{0}. \end{array}$$ (131)

However, we can derive the mean and variance of the distance distribution induced by the TiTv metric without specifying any relationship between *γ*_0_, *γ*_1_, and *γ*_2_. We proceed by computing $\text{P}[{\text{d}}_{ij}^{\text{TiTv}}\left(a\right)=k]$ for each $k\in D=\{0,\frac{1}{4},\frac{1}{2},\frac{3}{4},1\}$. Let *y* represent a random sample of size *p* from {0, 1, 2}, where

$$\begin{array}{c} y_{a}=\{\begin{array}{cc} 0 & \text{if}\;\text{locus}\;a\;\text{is}\;\text{PuPu},\\ 1 & \text{if}\;\text{locus}\;a\;\text{is}\;\text{PuPy},\\ 2 & \text{if}\;\text{locus}\;a\;\text{is}\;\text{PyPy}. \end{array} \end{array}$$ (132)

We derive $\text{P}[{\text{d}}_{ij}^{\text{TiTv}}\left(a\right)=0]$ as follows

$$\begin{array}{c} \begin{array}{cc} \text{P}[{\text{d}}_{ij}^{\text{TiTv}}\left(a\right)=0] & =\text{P}[y_{a}=0,X_{ia}=X_{ja}]\\ & +\text{P}[y_{a}=1,X_{ia}=X_{ja}]\\ & +\text{P}[y_{a}=2,X_{ia}=X_{ja}]\\ & =\gamma_{0}[{\left(1-f_{a}\right)}^{2}+4f_{a}\left(1-f_{a}\right)+f_{a}^{2}]\\ & +\gamma_{1}[{\left(1-f_{a}\right)}^{2}+4f_{a}\left(1-f_{a}\right)+f_{a}^{2}]\\ & +\gamma_{2}[{\left(1-f_{a}\right)}^{2}+4f_{a}\left(1-f_{a}\right)+f_{a}^{2}]\\ & =\left(\gamma_{0}+\gamma_{1}+\gamma_{2}\right)[{\left(1-f_{a}\right)}^{2}+4f_{a}\left(1-f_{a}\right)+f_{a}^{2}]\\ & ={\left(1-f_{a}\right)}^{2}+4f_{a}\left(1-f_{a}\right)+f_{a}^{2}, \end{array} \end{array}$$ (133)

where *f*_*a*_ is the probability of a minor allele occurring at locus *a*.

We derive $\text{P}[{\text{d}}_{ij}^{\text{TiTv}}\left(a\right)=\frac{1}{4}]$ as follows

$$\begin{array}{c} \begin{array}{cc} \text{P}[{\text{d}}_{ij}^{\text{TiTv}}\left(a\right)=\frac{1}{4}] & =2\text{P}[y_{a}=0,X_{ia}=0,X_{ja}=1]\\ & +2\text{P}[y_{a}=0,X_{ia}=1,X_{ja}=2]\\ & +2\text{P}[y_{a}=2,X_{ia}=0,X_{ja}=1]\\ & +2\text{P}[y_{a}=2,X_{ia}=1,X_{ja}=2]\\ & =4\gamma_{0}{\left(1-f_{a}\right)}^{3}f_{a}+4\gamma_{0}f_{a}^{3}\left(1-f_{a}\right)+4\gamma_{2}{\left(1-f_{a}\right)}^{3}f_{a}\\ & +4\gamma_{2}f_{a}^{3}\left(1-f_{a}\right)\\ & =4\gamma_{0}[{\left(1-f_{a}\right)}^{3}f_{a}+f_{a}^{3}\left(1-f_{a}\right)]\\ & +4\gamma_{2}[{\left(1-f_{a}\right)}^{3}f_{a}+f_{a}^{3}\left(1-f_{a}\right)]\\ & =4\left(\gamma_{0}+\gamma_{2}\right)[{\left(1-f_{a}\right)}^{3}f_{a}+f_{a}^{3}\left(1-f_{a}\right)], \end{array} \end{array}$$ (134)

where *f*_*a*_ is the probability of a minor allele occurring at locus *a*, *γ*_0_ is the probability of PuPu occurring at any locus *a*, and *γ*_2_ is the probability of PyPy occurring at any locus *a*.

We derive $\text{P}[{\text{d}}_{ij}^{\text{TiTv}}\left(a\right)=\frac{1}{2}]$ as follows

$$\begin{array}{c} \begin{array}{cc} \text{P}[{\text{d}}_{ij}^{\text{TiTv}}\left(a\right)=\frac{1}{2}] & =2\text{P}[y_{a}=1,X_{ia}=0,X_{ja}=1]\\ & +2\text{P}[y_{a}=1,X_{ia}=1,X_{ja}=2]\\ & =4\gamma_{1}{\left(1-f_{a}\right)}^{3}f_{a}+4\gamma_{1}f_{a}^{3}\left(1-f_{a}\right)\\ & =4\gamma_{1}[{\left(1-f_{a}\right)}^{3}f_{a}+f_{a}^{3}\left(1-f_{a}\right)], \end{array} \end{array}$$ (135)

where *f*_*a*_ is the probability of a minor allele occurring at locus *a* and *γ*_1_ is the probability of PuPy occurring at any locus *a*.

We derive $\text{P}[{\text{d}}_{ij}^{\text{TiTv}}\left(a\right)=\frac{3}{4}]$ as follows

$$\begin{array}{c} \begin{array}{cc} \text{P}[{\text{d}}_{ij}^{\text{TiTv}}\left(a\right)=\frac{3}{4}] & =2\text{P}[y_{a}=0,X_{ia}=0,X_{ja}=2]\\ & +2\text{P}[y_{a}=2,X_{ia}=0,X_{ja}=2]\\ & =2\gamma_{0}{\left(1-f_{a}\right)}^{2}f_{a}^{2}+2\gamma_{2}{\left(1-f_{a}\right)}^{2}f_{a}^{2}\\ & =2\left(\gamma_{0}+\gamma_{2}\right){\left(1-f_{a}\right)}^{2}f_{a}^{2}, \end{array} \end{array}$$ (136)

where *f*_*a*_ is the probability of a minor allele occurring at locus *a*, *γ*_0_ is the probability of PuPu occurring at any locus *a*, and *γ*_2_ is the probability of PyPy occurring at any locus *a*.

We derive $\text{P}[{\text{d}}_{ij}^{\text{TiTv}}\left(a\right)=1]$ as follows

$$\begin{array}{c} \begin{array}{cc} \text{P}[{\text{d}}_{ij}^{\text{TiTv}}\left(a\right)=1] & =2\text{P}[y_{a}=1,X_{ia}=0,X_{ja}=2]\\ & =2\gamma_{1}{\left(1-f_{a}\right)}^{2}f_{a}^{2}, \end{array} \end{array}$$ (137)

where *f*_*a*_ is the probability of a minor allele occurring at locus *a* and *γ*_1_ is the probability of PuPy occurring at any locus *a*.

Using the TiTv diff probabilities (Eqs 133–137), we compute the expected TiTv distance between instances i,j∈I as follows

$$\begin{array}{c} \begin{array}{cc} \text{E}\left(D_{ij}^{\text{TiTv}}\right) & =\sum_{a\in A}\left(\sum_{k\in D}k\cdot \text{P}[{\text{d}}_{ij}^{\text{TiTv}}\left(a\right)=k]\right)\\ & =\left(\gamma_{0}+\gamma_{2}+2\gamma_{1}\right)\sum_{a\in A}[{\left(1-f_{a}\right)}^{3}f_{a}+f_{a}^{3}\left(1-f_{a}\right)]\\ & +[\frac{3}{2}\left(\gamma_{0}+\gamma_{2}\right)+2\gamma_{1}]\sum_{a\in A}{\left(1-f_{a}\right)}^{2}f_{a}^{2}\\ & =\left(\gamma_{0}+\gamma_{2}+2\gamma_{1}\right)\sum_{a\in A}F^{\text{TiTv}}\left(a\right)+[\frac{3}{2}\left(\gamma_{0}+\gamma_{2}\right)+2\gamma_{1}]\sum_{a\in A}G^{\text{TiTv}}\left(a\right), \end{array} \end{array}$$ (138)

where $F^{\text{TiTv}}\left(a\right)={\left(1-f_{a}\right)}^{3}f_{a}+f_{a}^{3}\left(1-f_{a}\right)$, $G^{\text{TiTv}}\left(a\right)={\left(1-f_{a}\right)}^{2}f_{a}^{2}$, *f*_*a*_ is the probability of a minor allele occurring at locus *a*, *γ*_0_ is the probability of PuPu occurring at any locus *a*, *γ*_1_ is the probability of PuPy occurring at any locus *a*, and *γ*_2_ is the probability of PyPy occurring at any locus *a*. In contrast to the expected GM and AM distances (Eqs 117 and 122), the expected TiTv distance (Eq 138) depends on minor allele probabilities *f*_*a*_ for all a∈A and the genotype encoding probabilities *γ*_0_, *γ*_1_, and *γ*_2_.

The second moment about the origin for the TiTv distance is computed as follows

$$\begin{array}{c} \begin{array}{c} \begin{array}{cc} \text{E}[{\left(D_{ij}^{\text{TiTv}}\right)}^{2}] & =\text{E}[{\left(\sum_{a\in A}{\text{d}}_{ij}^{\text{TiTv}}\left(a\right)\right)}^{2}]\\ & =\text{E}[\sum_{a\in A}{\left({\text{d}}_{ij}^{\text{TiTv}}\left(a\right)\right)}^{2}]+2\text{E}[\sum_{r\in A}\sum_{s\leq r-1}{\text{d}}_{ij}^{\text{TiTv}}\left(r\right)\cdot {\text{d}}_{ij}^{\text{TiTv}}\left(s\right)]\\ & =\sum_{a\in A}\left(\sum_{k\in D}k^{2}\cdot \text{P}[{\text{d}}_{ij}^{\text{TiTv}}\left(a\right)=k]\right)\\ & +2\sum_{a\in A}\sum_{s\leq r-1}\left(\sum_{k\in D}k\cdot \text{P}[{\text{d}}_{ij}^{\text{TiTv}}\left(r\right)=k]\right)\cdot \left(\sum_{k\in D}k\cdot \text{P}[{\text{d}}_{ij}^{\text{TiTv}}\left(s\right)=k]\right)\\ & =[\frac{1}{4}\left(\gamma_{0}+\gamma_{2}\right)+\gamma_{1}]\sum_{a\in A}F^{\text{TiTv}}\left(a\right)+[\frac{9}{8}\left(\gamma_{0}+\gamma_{2}\right)+2\gamma_{1}]\sum_{a\in A}G^{\text{TiTv}}\left(a\right) \end{array}\\ +2\sum_{r\in A}\sum_{s\leq r-1}\prod_{\lambda \in \{r,s\}}\left(\left[\gamma_{0}+\gamma_{2}+2\gamma_{1}\right]F^{\text{TiTv}}\left(\lambda \right)+[\frac{3}{2}\left(\gamma_{0}+\gamma_{2}\right)+2\gamma_{1}]G^{\text{TiTv}}\left(\lambda \right)\right), \end{array} \end{array}$$ (139)

where $F^{\text{TiTv}}\left(a\right)={\left(1-f_{a}\right)}^{3}f_{a}+f_{a}^{3}\left(1-f_{a}\right)$, $G^{\text{TiTv}}\left(a\right)={\left(1-f_{a}\right)}^{2}f_{a}^{2}$, *f*_*a*_ is the probability of a minor allele occurring at locus *a*, *γ*_0_ is the probability of PuPu occurring at any locus *a*, *γ*_1_ is the probability of PuPy occurring at any locus *a*, and *γ*_2_ is the probability of PyPy occurring at any locus *a*.

Using the first (Eq 138) and second (Eq 139) raw moments of the TiTv distance, the variance is given by

$$\begin{array}{c} \begin{array}{c} \begin{array}{cc} \text{Var}\left(D_{ij}^{\text{TiTv}}\right) & =\text{E}[{\left(D_{ij}^{\text{TiTv}}\right)}^{2}]-{\left[\text{E}\left(D_{ij}^{\text{TiTv}}\right)\right]}^{2}\\ & =[\frac{1}{4}\left(\gamma_{0}+\gamma_{2}\right)+\gamma_{1}]\sum_{a\in A}F^{\text{TiTv}}\left(a\right)+[\frac{9}{8}\left(\gamma_{0}+\gamma_{2}\right)+2\gamma_{1}]\sum_{a\in A}G^{\text{TiTv}}\left(a\right) \end{array}\\ +2\sum_{r\in A}\sum_{s\leq r-1}\prod_{\lambda \in \{r,s\}}\left(\left[\gamma_{0}+\gamma_{2}+2\gamma_{1}\right]F^{\text{TiTv}}\left(\lambda \right)+[\frac{3}{2}\left(\gamma_{0}+\gamma_{2}\right)+2\gamma_{1}]G^{\text{TiTv}}\left(\lambda \right)\right)\\ \begin{array}{cc} & -{\left(\left[\gamma_{0}+\gamma_{2}+2\gamma_{1}\right]\sum_{a\in A}F^{\text{TiTv}}\left(a\right)+[\frac{3}{2}\left(\gamma_{0}+\gamma_{2}\right)+2\gamma_{1}]\sum_{a\in A}G^{\text{TiTv}}\left(a\right)\right)}^{2}\\ & =[\frac{1}{4}\left(\gamma_{0}+\gamma_{2}\right)+\gamma_{1}]\sum_{a\in A}F^{\text{TiTv}}\left(a\right)+[\frac{9}{8}\left(\gamma_{0}+\gamma_{2}\right)+2\gamma_{1}]\sum_{a\in A}G^{\text{TiTv}}\left(a\right)\\ & -\sum_{a\in A}{\left(\left[\gamma_{0}+\gamma_{2}+2\gamma_{1}\right]F^{\text{TiTv}}\left(a\right)+[\frac{3}{2}\left(\gamma_{0}+\gamma_{2}\right)+2\gamma_{1}]G^{\text{TiTv}}\left(a\right)\right)}^{2}, \end{array} \end{array} \end{array}$$ (140)

where $F^{\text{TiTv}}\left(a\right)={\left(1-f_{a}\right)}^{3}f_{a}+f_{a}^{3}\left(1-f_{a}\right)$, $G^{\text{TiTv}}\left(a\right)={\left(1-f_{a}\right)}^{2}f_{a}^{2}$, *f*_*a*_ is the probability of a minor allele occurring at locus *a*, *γ*_0_ is the probability of PuPu occurring at any locus *a*, *γ*_1_ is the probability of PuPy occurring at any locus *a*, and *γ*_2_ is the probability of PyPy occurring at any locus *a*.

With the mean (Eq 138) and variance (Eq 140) estimates, the asymptotic TiTv distance distribution is given by the following

$$\begin{array}{c} D_{ij}^{\text{TiTv}}\dot{∼}N(\left(\gamma_{0}+\gamma_{2}+2\gamma_{1}\right)\sum_{a\in A}F^{\text{TiTv}}\left(a\right)+[\frac{3}{2}\left(\gamma_{0}+\gamma_{2}\right)+2\gamma_{1}]\sum_{a\in A}G^{\text{TiTv}}\left(a\right),\\ {}[\frac{1}{4}\left(\gamma_{0}+\gamma_{2}\right)+\gamma_{1}]\sum_{a\in A}F^{\text{TiTv}}\left(a\right)+[\frac{9}{8}\left(\gamma_{0}+\gamma_{2}\right)+2\gamma_{1}]\sum_{a\in A}G^{\text{TiTv}}\left(a\right)\\ -\sum_{a\in A}{\left(\left[\gamma_{0}+\gamma_{2}+2\gamma_{1}\right]F^{\text{TiTv}}\left(a\right)+[\frac{3}{2}\left(\gamma_{0}+\gamma_{2}\right)+2\gamma_{1}]G^{\text{TiTv}}\left(a\right)\right)}^{2}), \end{array}$$ (141)

where $F^{\text{TiTv}}\left(a\right)={\left(1-f_{a}\right)}^{3}f_{a}+f_{a}^{3}\left(1-f_{a}\right)$, $G^{\text{TiTv}}\left(a\right)={\left(1-f_{a}\right)}^{2}f_{a}^{2}$, *f*_*a*_ is the probability of a minor allele occurring at locus *a*, *γ*_0_ is the probability of PuPu occurring at any locus *a*, *γ*_1_ is the probability of PuPy occurring at any locus *a*, and *γ*_2_ is the probability of PyPy occurring at any locus *a*.

Given upper and lower bounds *l* and *u*, respectively, of the success probability sampling interval, the average success probability (or average MAF) is computed as follows

$$\begin{array}{c} {\bar{f}}_{a}=\frac{1}{2}\left(l+u\right). \end{array}$$ (142)

**Fig 6:**
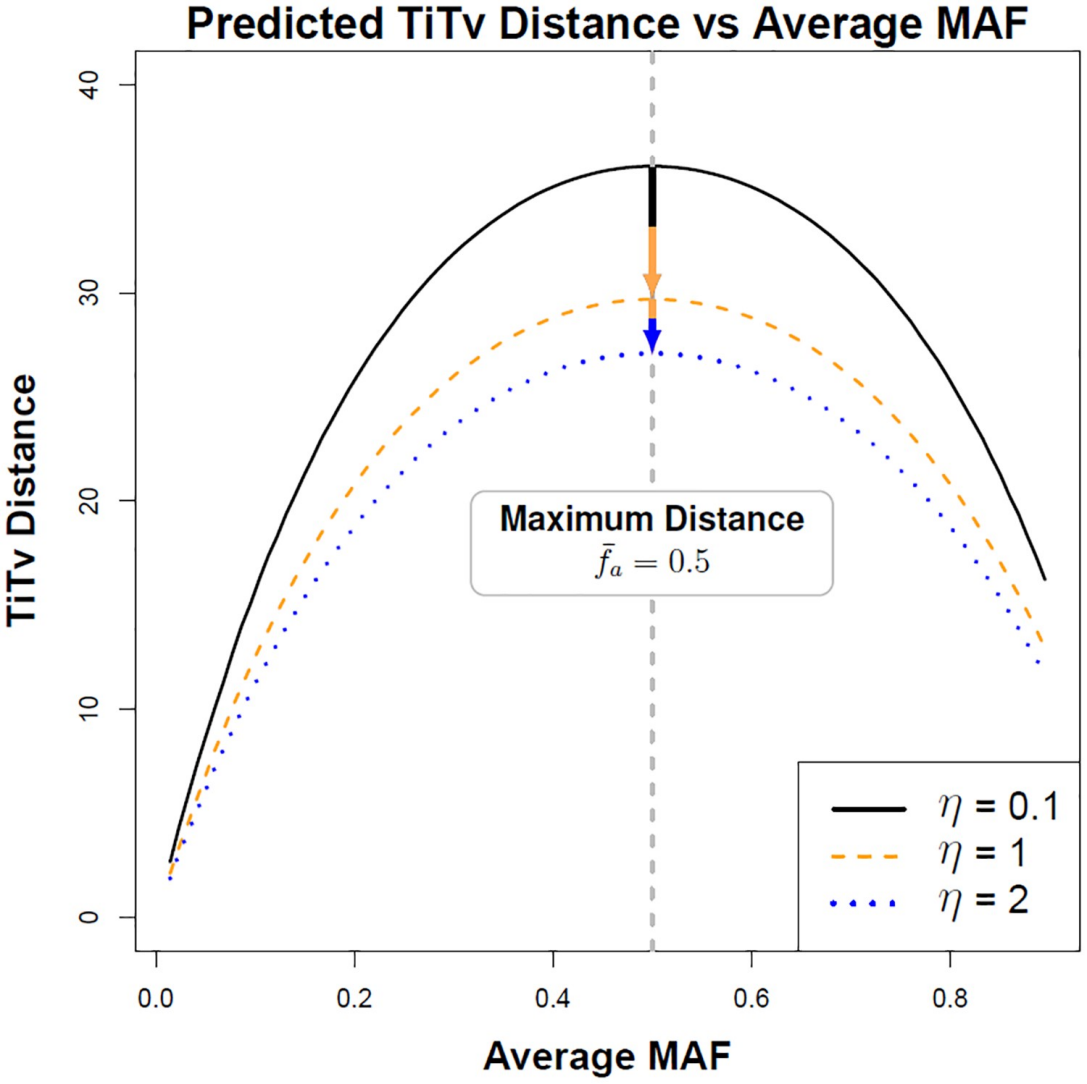
Predicted average TiTv distance as a function of average minor allele frequency ${\bar{f}}_{a}$ (see Eq 142). Success probabilities *f*_*a*_ are drawn from a sliding window interval from 0.01 to 0.9 in increments of about 0.009 and *m* = *p* = 100. For *η* = 0.1, where *η* is the Ti/Tv ratio given by Eq 126, Tv is ten times more likely than Ti and results in larger distance. Increasing to *η* = 1, Tv and Ti are equally likely and the distance is lower. In line with real data for *η* = 2, Tv is half as likely as Ti so the distances are relatively small.

**Fig 7:**
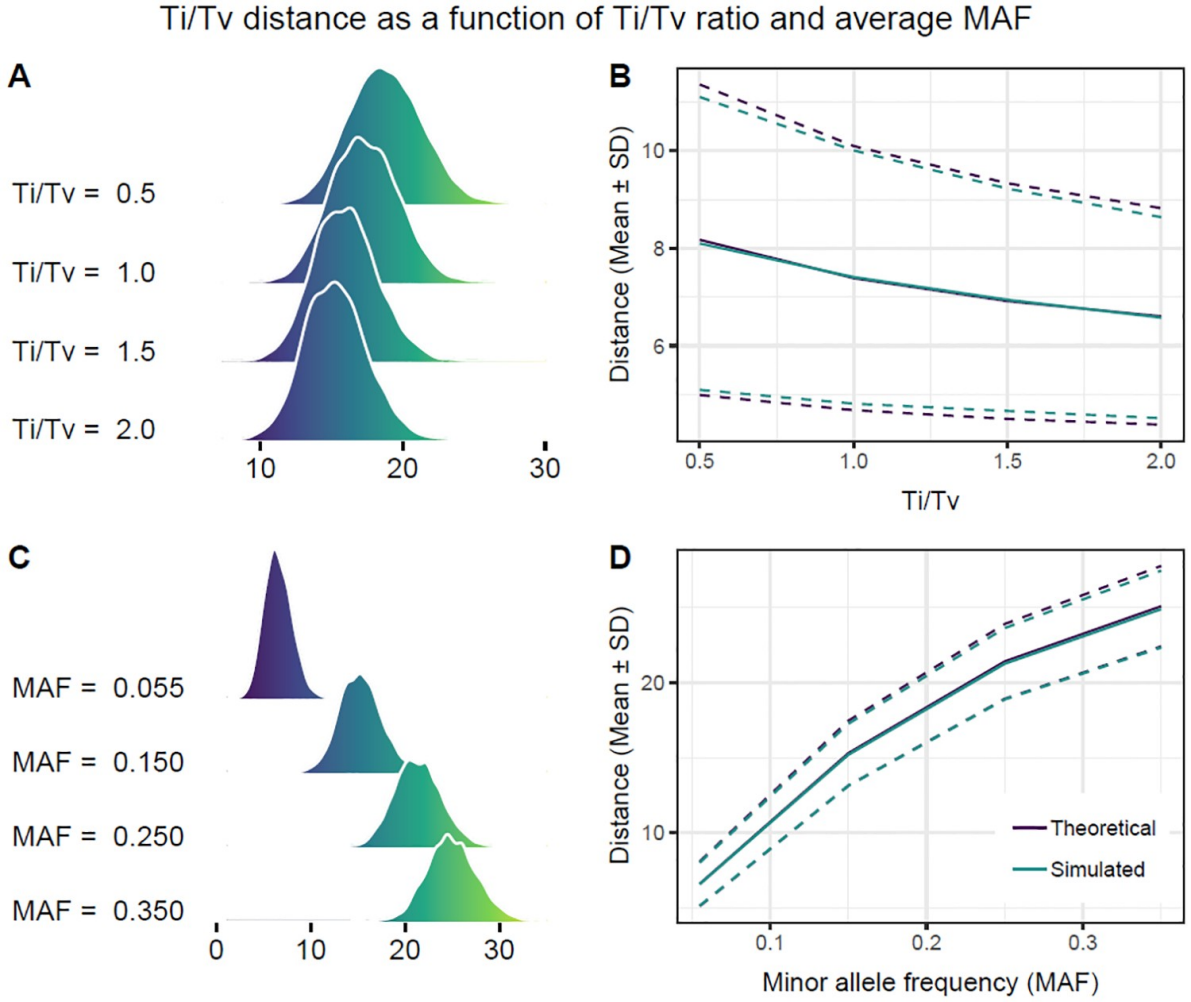
Density curves and moments of TiTv distance as a function of average MAF ${\bar{f}}_{a}$, given by Eq 142, and Ti/Tv ratio *η*, given by Eq 127. We fix *m* = *p* = 100 for all simulated TiTv distances. (**A**) For fixed ${\bar{f}}_{a}=0.055$, TiTv distance density is plotted as a function of increasing *η*. TiTv distance decreases as *η* increases. For *η* = Ti/Tv = 0.5, there are twice as many transversions as there are transitions. On the other hand, *η* = Ti/Tv = 2 indicates that there are half as many transversions as transitions. Since transversions encode a larger magnitude distance than transitions, this behavior is expected. (**B**) Simulated and predicted mean ± SD are shown as a function of increasing Ti/Tv ratio *η*. Distance decreases as Ti/Tv increases. Theoretical and simulated moments are approximately the same. (**C**) For fixed *η* = 2, TiTv distance density is plotted as a function of increasing ${\bar{f}}_{a}$. TiTv distance increases as ${\bar{f}}_{a}$ approaches maximum of 0.5, which means that there is about the same frequency of minor alleles as major alleles. (**D**) Simulated and predicted mean ± SD as a function of increasing average MAF ${\bar{f}}_{a}$. Distance increases as the number of minor alleles increases. Theoretical and simulated moments are approximately the same.

The maximum TiTv distance occurs at ${\bar{f}}_{a}=0.5$ for any fixed Ti/Tv ratio *η* (Eq 127), which is the inflection point about which the minor allele changes at locus *a* (Fig 6). If few minor alleles are present (${\bar{f}}_{a}\to 0$), the predicted TiTv distance approaches 0. The same is true after the minor allele switches (${\bar{f}}_{a}\to 1$). To explore how TiTv distance changes with increased minor allele frequency, we fixed the Ti/Tv ratio *η* and generated simulated TiTv distances for ${\bar{f}}_{a}=0.055,0.150,0.250,\;\text{and}\;0.350$ (Fig 7A). For fixed *η*, TiTv distance increases significantly with increased ${\bar{f}}_{a}$. We similarly fixed the average minor allele frequency ${\bar{f}}_{a}$ and generated simulated TiTv distances for *η* = Ti/Tv = 0.5, 1, 1.5, and 2 (Fig 7C). The TiTv distance decreases slightly with increased *η* = Ti/Tv. As *η* → 0^+^, the data is approaching all Tv and no Ti, which means the TiTv distance is larger by definition. On the other hand, the TiTv distance decreases as *η* → 2^−^ because the data is approaching approximately twice as many Ti as there are Tv, which is typical for GWAS data in humans.

We also compared theoretical and sample moments as a function of *η* = Ti/Tv and ${\bar{f}}_{a}$ for the TiTv distance metric (Fig 7B and 7D). We fixed ${\bar{f}}_{a}$ and computed the theoretical and simulated moments as a function of *η* (Fig 7B). Theoretical average TiTv distance (Eq 138) and simulated TiTv average distance are approximately equal as *η* increases. Theoretical standard deviation (Eq 140) and simulated TiTv standard deviation differ slightly. We also fixed *η* and computed theoretical and sample moments as a function of ${\bar{f}}_{a}$ (Fig 7D). In this case, there is approximate agreement with simulated and theoretical moments as ${\bar{f}}_{a}$ increases.

**Fig 8:**
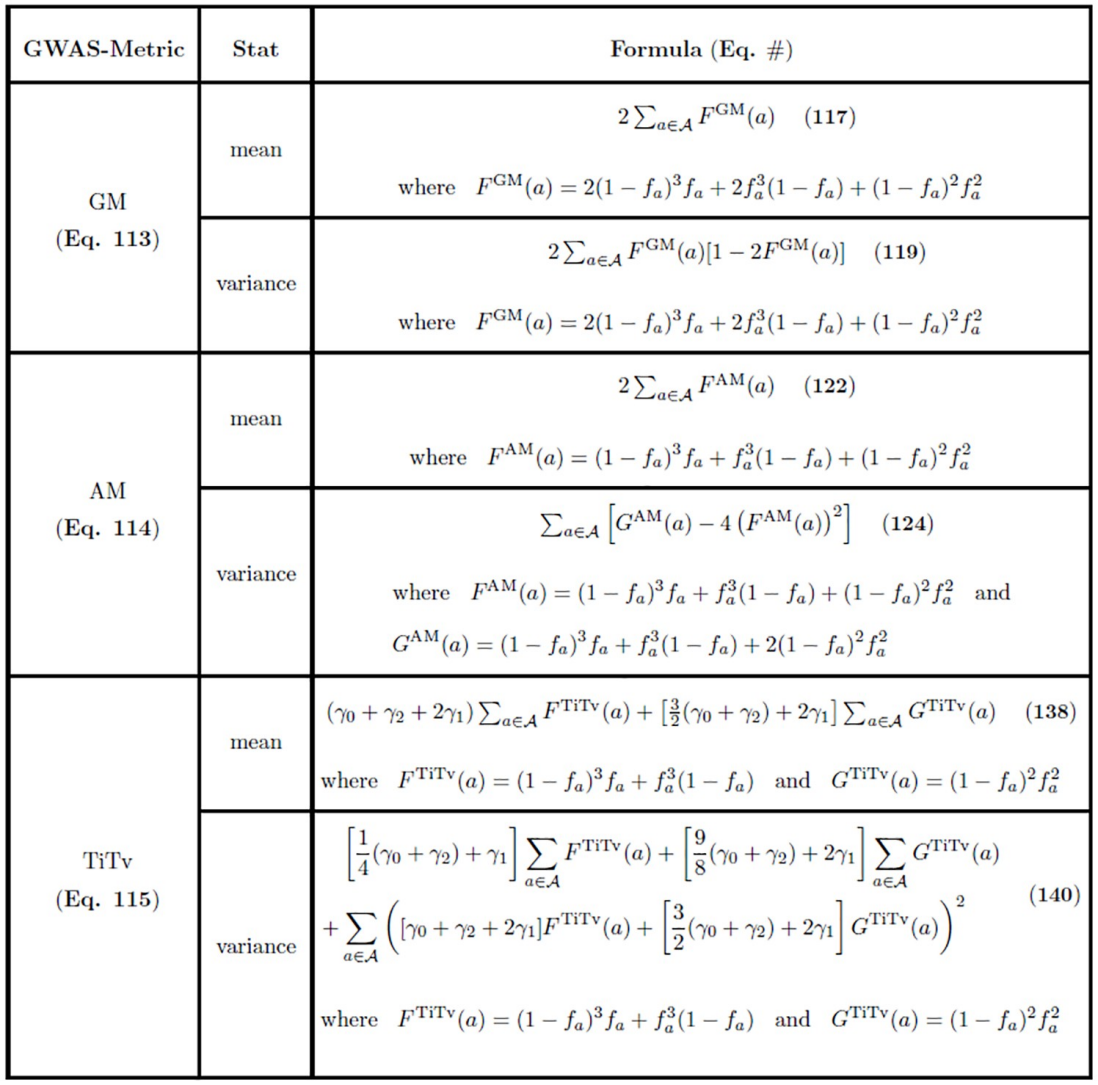
Asymptotic estimates of means and variances of genotype mismatch (GM) (Eq 113), allele mismatch (AM) (Eq 114), and transition-transversion (TiTv) (Eq 115) distance metrics in GWAS data (*p* ≫ 1). GWAS data $X_{ia}∼B\left(2,f_{a}\right)$, where *f*_*a*_ for all a∈A are the probabilities of a minor allele occurring at locus *a*. For the TiTv distance metric, we have the additional encoding that uses *γ*_0_ = P(PuPu), *γ*_1_ = P(PuPy), and *γ*_2_ = P(PyPy).

We summarize our moment estimates for GWAS distance metrics (Eqs 113–115) (Fig 8) organized by metric, statistic (mean or variance), and asymptotic formula. Next we consider the important case of distributions of GWAS distances projected onto a single attribute (Eqs 110–112).

### 5.4 Distribution of one-dimensional projection of GWAS distance onto a SNP

We previously derived the exact distribution of the one-dimensional projected distance onto an attribute in continuous data (Section 3.2.3), which is used as the predictor in NPDR to calculate relative attribute importance in the form of standardized beta coefficients. GWAS data and the metrics we have considered are discrete. Therefore, we derive the density function for each diff metric (Eqs 110–112), which also serves as the probability distribution for each metric, respectively.

The support of the GM metric (Eq 110) is simply {0, 1}, so we derive the probability, $\text{P}[{\text{d}}_{ij}^{\text{GM}}\left(a\right)=k]$, of this diff taking on each of these two possible values. First, the probability that the GM diff is equal to zero is given by

$$\begin{array}{c} \begin{array}{cc} f_{\text{GM}}\left(0;f_{a}\right)=\text{P}[{\text{d}}_{ij}^{\text{GM}}\left(a\right)=0] & =\text{P}\left(X_{ia}=0,X_{ja}=0\right)+\text{P}\left(X_{ia}=1,X_{ja}=1\right)\\ & \;+\text{P}\left(X_{ia}=2,X_{ja}=2\right)\\ & ={\left(1-f_{a}\right)}^{4}+4f_{a}^{2}{\left(1-f_{a}\right)}^{2}+f_{a}^{4}, \end{array} \end{array}$$ (143)

where *f*_*a*_ is the probability of a minor allele occurring at locus *a*.

Similarly, the probability that the GM diff is equal to 1 is derived as follows

$$\begin{array}{c} \begin{array}{cc} f_{\text{GM}}\left(1;f_{a}\right)=\text{P}[{\text{d}}_{ij}^{\text{GM}}\left(a\right)=1] & =2\text{P}\left(X_{ia}=0,X_{ja}=1\right)+2\text{P}\left(X_{ia}=1,X_{ia}=2\right)\\ & \;+2\text{P}\left(X_{ia}=0,X_{ja}=2\right)\\ & =4{\left(1-f_{a}\right)}^{3}f_{a}+4f_{a}^{3}\left(1-f_{a}\right)+2f_{a}^{2}{\left(1-f_{a}\right)}^{2}, \end{array} \end{array}$$ (144)

where *f*_*a*_ is the probability of a minor allele occurring at locus *a*.

This leads us to the probability distribution of the GM diff metric, which is the distribution of the one-dimensional GM distance projected onto a single SNP. This distribution is given by

$$\begin{array}{c} f_{\text{GM}}\left(d;f_{a}\right)=\{\begin{array}{cc} {\left(1-f_{a}\right)}^{4}+4f_{a}^{2}{\left(1-f_{a}\right)}^{2}+f_{a}^{4} & d=0,\\ 4{\left(1-f_{a}\right)}^{3}f_{a}+4f_{a}^{3}\left(1-f_{a}\right)+2f_{a}^{2}{\left(1-f_{a}\right)}^{2} & d=1, \end{array} \end{array}$$ (145)

where *f*_*a*_ is the probability of a minor allele occurring at locus *a*.

The mean and variance of this GM diff distribution can easily be derived using this newly determined density function (Eq 145). The average GM diff is given by the following

$$\begin{array}{c} \text{E}[{\text{d}}_{ij}^{\text{GM}}\left(a\right)]=2F^{\text{GM}}\left(a\right), \end{array}$$ (146)

where $F^{\text{GM}}=2{\left(1-f_{a}\right)}^{3}f_{a}+2f_{a}^{3}\left(1-f_{a}\right)+f_{a}^{2}{\left(1-f_{a}\right)}^{2}$ and *f*_*a*_ is the probability of a minor allele occurring at locus *a*.

The variance of the GM diff metric is given by

$$\begin{array}{c} \text{Var}[{\text{d}}_{ij}^{\text{GM}}\left(a\right)]=2F^{\text{GM}}\left(a\right)[1-2F^{\text{GM}}\left(a\right)], \end{array}$$ (147)

where $F^{\text{GM}}=2{\left(1-f_{a}\right)}^{3}f_{a}+2f_{a}^{3}\left(1-f_{a}\right)+f_{a}^{2}{\left(1-f_{a}\right)}^{2}$ and *f*_*a*_ is the probability of a minor allele occurring at locus *a*.

The support of the AM metric (Eq 111) is {0, 1/2, 1}. Beginning with the probability of the AM diff being equal to 0, we have the following probability

$$\begin{array}{c} \begin{array}{cc} f_{\text{AM}}\left(0;f_{a}\right)=\text{P}[{\text{d}}_{ij}^{\text{AM}}\left(a\right)=0] & =\text{P}\left(X_{ia}=0,X_{ja}=0\right)+\text{P}\left(X_{ia}=1,X_{ja}=1\right)\\ & \;+\text{P}\left(X_{ia}=2,X_{ja}=2\right)\\ & ={\left(1-f_{a}\right)}^{4}+4f_{a}^{2}{\left(1-f_{a}\right)}^{2}+f_{a}^{4}, \end{array} \end{array}$$ (148)

where *f*_*a*_ is the probability of a minor allele occurring at locus *a*.

The probability of the AM diff metric being equal to 1/2 is computed similarly as follows

$$\begin{array}{c} \begin{array}{cc} f_{\text{AM}}\left(1/2;f_{a}\right)=\text{P}[{\text{d}}_{ij}^{\text{AM}}\left(a\right)=1/2] & =2\text{P}\left(X_{ia}=0,X_{ja}=1\right)+2\text{P}\left(X_{ia}=1,X_{ia}=2\right)\\ & =4{\left(1-f_{a}\right)}^{3}f_{a}+4f_{a}^{3}\left(1-f_{a}\right), \end{array} \end{array}$$ (149)

where *f*_*a*_ the probability of a minor allele occurring at locus *a*.

Finally, the probability of the AM diff metric being equal to 1 is given by the following

$$\begin{array}{c} \begin{array}{cc} f_{\text{AM}}\left(1;f_{a}\right)=\text{P}[{\text{d}}_{ij}^{\text{AM}}\left(a\right)=1] & =2\text{P}\left(X_{ia}=0,X_{ja}=2\right)\\ & =2f_{a}^{2}{\left(1-f_{a}\right)}^{2}, \end{array} \end{array}$$ (150)

where *f*_*a*_ is the probability of a minor allele occurring at locus *a*.

As in the case of the GM diff metric, we now have the probability distribution of the AM diff metric. This also serves as the distribution of the one-dimensional AM distance projected onto a single SNP, and is given by the following

$$\begin{array}{c} f_{\text{AM}}\left(d;f_{a}\right)=\{\begin{array}{cc} {\left(1-f_{a}\right)}^{4}+4f_{a}^{2}{\left(1-f_{a}\right)}^{2}+f_{a}^{4} & d=0,\\ 4{\left(1-f_{a}\right)}^{3}f_{a}+4f_{a}^{3}\left(1-f_{a}\right) & d=1/2,\\ 2f_{a}^{2}{\left(1-f_{a}\right)}^{2} & d=1, \end{array} \end{array}$$ (151)

where *f*_*a*_ is the probability of a minor allele occurring at locus *a*.

The mean and variance of this AM diff distribution is derived using the corresponding density function (Eq 151). The average AM diff is given by

$$\begin{array}{c} \text{E}[{\text{d}}_{ij}^{\text{AM}}\left(a\right)]=2F^{\text{AM}}\left(a\right), \end{array}$$ (152)

where $F^{\text{AM}}\left(a\right)={\left(1-f_{a}\right)}^{3}f_{a}+f_{a}^{3}\left(1-f_{a}\right)+f_{a}^{2}{\left(1-f_{a}\right)}^{2}$ and *f*_*a*_ is the probability of a minor allele occurring at locus *a*.

The variance of the AM diff metric is given by

$$\begin{array}{c} \text{Var}[{\text{d}}_{ij}^{\text{AM}}\left(a\right)]=G^{\text{AM}}\left(a\right)-4{\left[F^{\text{AM}}\left(a\right)\right]}^{2}, \end{array}$$ (153)

where $G^{\text{AM}}\left(a\right)={\left(1-f_{a}\right)}^{3}f_{a}+f_{a}^{3}\left(1-f_{a}\right)+2{\left(1-f_{a}\right)}^{2}f_{a}^{2}$, $F^{\text{AM}}\left(a\right)={\left(1-f_{a}\right)}^{3}f_{a}+f_{a}^{3}\left(1-f_{a}\right)+f_{a}^{2}\left(1-f_{a}\right)$, *f*_*a*_ is the probability of a minor allele occurring at locus *a*.

For the TiTv diff metric (Eq 112), the support is {0, 1/4, 1/2, 3/4, 1}. We have already derived the probability that the TiTv diff assumes each of the values of its support (Eqs 133–137). Therefore, we have the following distribution of the TiTv diff metric

$$\begin{array}{c} f_{\text{TiTv}}\left(d;f_{a},\gamma_{0},\gamma_{1},\gamma_{2},\eta \right)=\{\begin{array}{cc} {\left(1-f_{a}\right)}^{4}+4f_{a}^{2}{\left(1-f_{a}\right)}^{2}+f_{a}^{4} & d=0,\\ 4\left(\gamma_{0}+\gamma_{2}\right)[{\left(1-f_{a}\right)}^{3}f_{a}+f_{a}^{3}\left(1-f_{a}\right)] & d=1/4,\\ 4\gamma_{1}[{\left(1-f_{a}\right)}^{3}f_{a}+f_{a}^{3}\left(1-f_{a}\right)] & d=1/2,\\ 2\left(\gamma_{0}+\gamma_{2}\right){\left(1-f_{a}\right)}^{2}f_{a}^{2} & d=3/4,\\ 2\gamma_{1}{\left(1-f_{a}\right)}^{2}f_{a}^{2} & d=1, \end{array} \end{array}$$ (154)

where *f*_*a*_ is the probability of a minor allele occurring at locus *a*, *γ*_0_ is the probability of PuPu at locus *a*, *γ*_1_ is the probability of PuPy at locus *a*, *γ*_2_ is the probability of PyPy at locus *a*, and *η* is the Ti/Tv ratio (Eq 127).

The mean and variance of this TiTv diff distribution is derived using the corresponding density function (Eq 154). The average TiTv diff is given by

$$\begin{array}{c} \begin{array}{cc} \text{E}[{\text{d}}_{ij}^{\text{TiTv}}\left(a\right)] & =\left(\gamma_{0}+\gamma_{2}+2\gamma_{1}\right)F^{\text{TiTv}}\left(a\right)+[\frac{3}{2}\left(\gamma_{0}+\gamma_{2}\right)+2\gamma_{1}]G^{\text{TiTv}}\left(a\right), \end{array} \end{array}$$ (155)

where $F^{\text{TiTv}}\left(a\right)={\left(1-f_{a}\right)}^{3}f_{a}+f_{a}^{3}\left(1-f_{a}\right)$, $G^{\text{TiTv}}\left(a\right)=f_{a}^{2}{\left(1-f_{a}\right)}^{2}$, *f*_*a*_ is the probability of a minor allele occurring at locus *a*, *γ*_0_ is the probability of PuPu at locus *a*, *γ*_1_ is the probability of PuPy at locus *a*, and *γ*_2_ is the probability of PyPy at locus *a*.

The variance of the TiTv diff metric is given by

$$\begin{array}{c} \begin{array}{cc} \text{Var}[{\text{d}}_{ij}^{\text{TiTv}}\left(a\right)] & =[\frac{1}{4}\left(\gamma_{0}+\gamma_{2}\right)+\gamma_{1}]F^{\text{TiTv}}\left(a\right)+[\frac{9}{8}\left(\gamma_{0}+\gamma_{2}\right)+2\gamma_{1}]G^{\text{TiTv}}\left(a\right)\\ & -{\left(\left(\gamma_{0}+\gamma_{2}+2\gamma_{1}\right)F^{\text{TiTv}}\left(a\right)+[\frac{3}{2}\left(\gamma_{0}+\gamma_{2}\right)+2\gamma_{1}]G^{\text{TiTv}}\left(a\right)\right)}^{2}, \end{array} \end{array}$$ (156)

where $F^{\text{TiTv}}\left(a\right)={\left(1-f_{a}\right)}^{3}f_{a}+f_{a}^{3}\left(1-f_{a}\right)$, $G^{\text{TiTv}}\left(a\right)=f_{a}^{2}{\left(1-f_{a}\right)}^{2}$, *f*_*a*_ is the probability of a minor allele occurring at locus *a*, *γ*_0_ is the probability of PuPu at locus *a*, *γ*_1_ is the probability of PuPy at locus *a*, and *γ*_2_ is the probability of PyPy at locus *a*.

These novel distribution results for the projection of pairwise GWAS distances onto a single genetic variant, as well as results for the full space of *p* variants, can inform NPDR and other nearest-neighbor distance-based feature selection algorithms. We show density curves for GM (S23 Fig in S1 File), AM (S24 Fig in S1 File), and TiTv (S25 Fig in S1 File) for each possible support value. Next we introduce our new diff metric and distribution results for time-series derived correlation-based data, with a particular application to resting-state fMRI.

## 6 Time series correlation-based distance distribution

In this section, we introduce a new metric and projected distance for correlation data, and we derive its asymptotic properties. For this type of data, each of the *m* subjects has a correlation matrix *A*^(*p*×*p*)^ between pairs of attributes from the set A (p=|A|). The application we have in mind is resting-state fMRI (rs-fMRI) data, where correlations are calculated from the time-series activity between brain regions. However, the methods that follow are relevant to all correlation-based data. The |A| attributes in rs-fMRI are known as Regions of Interest (ROIs), which are collections of spatially proximal voxels [26]. Correlation in their time-series activity is calculated between voxels or ROIs based on a known brain atlas [27].

In rs-fMRI feature selection applications, a common approach is to use the correlation between ROIs as the attribute. However, our goal is to allow the individual ROIs to be the attributes of interest (*a*) even though the data is correlation. Thus, we propose the following attribute projection (diff)

$$\begin{array}{c} {\text{d}}_{ij}^{\text{ROI}}\left(a\right)=\sum_{k\neq a}|A_{ka}^{\left(i\right)}-A_{ka}^{\left(j\right)}|, \end{array}$$ (157)

where $A_{ak}^{\left(i\right)}$ and $A_{ak}^{\left(j\right)}$ are the correlations between ROI *a* and ROI *k* for instances i,j∈I, respectively. With this rs-fMRI diff, we define the pairwise distance between two instances i,j∈I as follows

$$\begin{array}{c} D_{ij}^{\text{fMRI}}=\sum_{a\in A}{\text{d}}_{ij}^{\text{ROI}}\left(a\right), \end{array}$$ (158)

which is based on Manhattan (*q* = 1). This metric may be expanded to general *q*, but we only consider *q* = 1.

**Fig 9:**
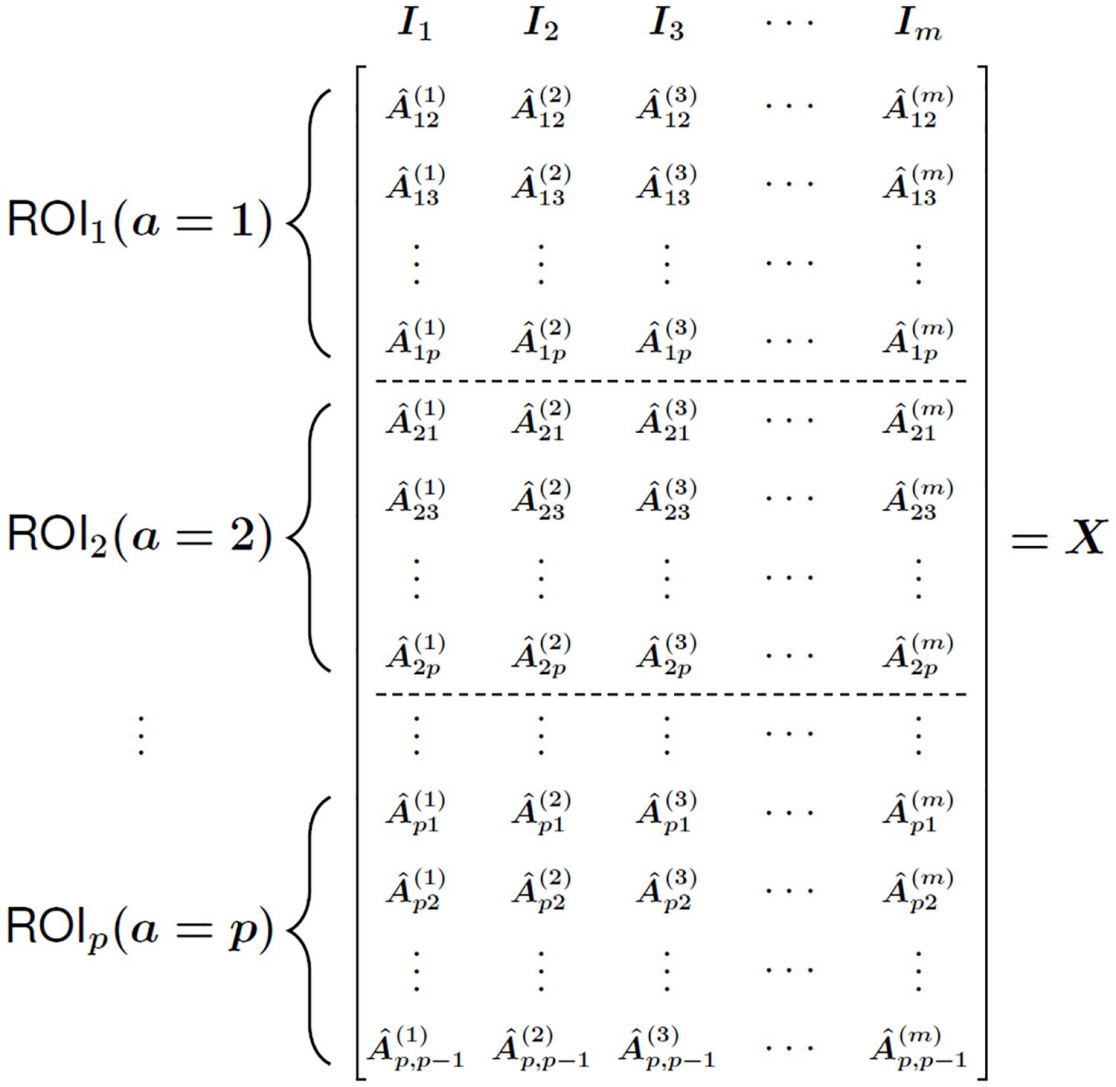
Organization based on brain regions of interest (ROIs) of resting-state fMRI correlation dataset consisting of transformed correlation matrices for *m* subjects. Each column corresponds to an instance (or subject) *I*_*j*_ and each subset of rows corresponds to the correlations for an ROI attribute (*p* sets). The notation ${\hat{A}}_{ak}^{\left(j\right)}$ represents the r-to-z transformed correlation between attributes (ROIs) *a* and *k* ≠ *a* for instance *j*.

In order for comparisons between different correlations to be possible, we first perform a Fisher r-to-z transform on the correlations. This transformation makes the data approximately normally distributed with stabilized variance across different samples. After this transformation, we then load all of the transformed correlations into a *p*(*p* − 1) × *m* matrix *X* (Fig 9). Each column of *X* represents a single instance (or subject) in rs-fMRI data. Contrary to a typical *p* × *m* data set, each row does not represent a single attribute. Rather, each attribute (or ROI) is represented by *p* − 1 consecutive rows. The first *p* − 1 rows represent ROI_1_, the next *p* − 1 rows represent ROI_2_, and so on until the last *p* − 1 rows that represent ROI_*p*_. For a given column of *X*, we exclude pairwise correlations between an ROI and itself. Therefore, the matrix does not contain ${\hat{A}}_{aa}^{\left(i\right)}$ for any i∈I or a∈A. Furthermore, symmetry of correlation matrices means that each column contains exactly two of each element of the upper triangle of an instance’s transformed correlation matrix. For example, ${\hat{A}}_{ka}^{\left(i\right)}={\hat{A}}_{ak}^{\left(i\right)}$ for *k* ≠ *a* and both will be contained in a given column of *X* for each a∈A. Based on our rs-fMRI diff (Eq 157), the organization of *X* makes computation of each value of the diff very simple. In order to compute each value of the rs-fMRI diff, we just need to know the starting and ending row indices for a given ROI. Starting indices are given by

$${\text{start}}_{k}=\left(k-1\right)\left(p-1\right)+1,\;\;\text{for}\;k=1,2,\ldots ,p$$

and ending indices are given by

$${\text{end}}_{k}=k\left(p-1\right),\;\;\text{for}\;k=1,2,\ldots ,p.$$

These indices allow us to extract just the rows necessary to compute the rs-fMRI diff for a fixed ROI.

We further transform the data matrix *X* by standardizing so that each of the *m* columns has zero mean and unit variance. Therefore, the data in matrix *X* are approximately standard normal. Since we assume independent samples, the standard rs-fMRI distance is asymptotically normal. Gaussian limiting behavior is illustrated in the form of histograms as shown previously (S7 Fig in S1 File). Recall that the mean (Eq 41) and variance (Eq 42) of the Manhattan (*L*_1_) distance distribution for standard normal data are $\frac{2p}{\sqrt{\pi }}$ and $\frac{2\left(\pi -2\right)p}{\pi }$, respectively. This allows us to easily derive the expected pairwise distance between instances i,j∈I in rs-fMRI data as follows

$$\begin{array}{c} \begin{array}{cc} \text{E}\left(D_{ij}^{\text{fMRI}}\right) & =\text{E}\left(\sum_{a\in A}{\text{d}}_{ij}^{\text{ROI}}\left(a\right)\right)\\ & =\text{E}\left(\sum_{a\in A}\sum_{k\neq a}|{\hat{A}}_{ak}^{\left(i\right)}-{\hat{A}}_{ak}^{\left(j\right)}|\right)\\ & =\sum_{a\in A}\sum_{k\neq a}\text{E}\left(|{\hat{A}}_{ak}^{\left(i\right)}-{\hat{A}}_{ak}^{\left(j\right)}|\right)\\ & =\sum_{a\in A}\sum_{k\neq a}\frac{2}{\sqrt{\pi }}\\ & =\frac{2p\left(p-1\right)}{\sqrt{\pi }}. \end{array} \end{array}$$ (159)

The expected pairwise rs-fMRI distance (Eq 159) grows on the order of *p*(*p* − 1), which is the total number of transformed pairwise correlations in each column of *X* (Fig 9). This is similar to the case of a typical *m* × *p* data matrix in which the data is standard normal and Manhattan distances are computed between instances.

We first derive the variance of the rs-fMRI distance by making an independence assumption with respect to the magnitude differences $|{\hat{A}}_{ak}^{\left(i\right)}-{\hat{A}}_{ak}^{\left(j\right)}|$ for all k≠a∈A. We observe empirically that this assumption gives a reasonable estimate of the actual variance of rs-fMRI distances in simulated data, but there is a consistent discrepancy between predicted and simulated variances. We begin our derivation of the variance of rs-fMRI distances by assuming that cross-covariances between the diffs of different pairs of ROIs are negligible. This allows us to determine the relationship between the predicted variance under the independence assumption and the simulated variance. We proceed by applying the variance operator linearly as follows

$$\begin{array}{c} \begin{array}{cc} \text{Var}\left(D_{ij}^{\text{fMRI}}\right) & =\text{Var}\left(\sum_{a\in A}{\text{d}}_{ij}^{\text{ROI}}\left(a\right)\right)\\ & =\text{Var}\left(\sum_{a\in A}\sum_{k\neq a}|{\hat{A}}_{ak}^{\left(i\right)}-{\hat{A}}_{ak}^{\left(j\right)}|\right)\\ & =\sum_{a\in A}\sum_{k\neq a}\text{Var}\left(|{\hat{A}}_{ak}^{\left(i\right)}-{\hat{A}}_{ak}^{\left(j\right)}|\right)\\ & =\sum_{a\in A}\sum_{k\neq a}\frac{2\left(\pi -2\right)}{\pi }\\ & =\frac{2\left(\pi -2\right)\left(p-1\right)p}{\pi }. \end{array} \end{array}$$ (160)

Similar to the case of an *m* × *p* data matrix containing standard normal data, we have an rs-fMRI distance variance that grows on the order of *p*(*p* − 1), which is the total number of pairwise associations in a column of data matrix *X* (Fig 9). Therefore, the expected rs-fMRI distance (Eq 159) and the variance of the rs-fMRI distance (Eq 160) increase on the same order.

The independence assumption used to derive the variance of our rs-fMRI distance metric (Eq 160) is not satisfied because a single value of the diff (Eq 157) includes the same fixed ROI, *a*, for each term in the sum for all *k* ≠ *a*. Therefore, the linear application of the variance operator we have previously employed does not account for the additional cross-covariance that exists. However, we have seen empirically that the theoretical variance of the distance we computed for the rs-fMRI distance metric (Eq 160) still reasonably approximates the sample variance, there is a slight discrepancy between our theoretical rs-fMRI distance metric variance (Eq 160) and the sample variance. More precisely, the formula we have given for the variance (Eq 160) consistently underestimates the sample variance of the rs-fMRI distance. To adjust for this discrepancy, we determine a corrected formula by assuming that there is dependence between the terms of the rs-fMRI diff and estimate the cross-covariance between rs-fMRI diffs of different pairs of ROIs.

We begin the derivation of our corrected formula by writing the variance as a two-part sum, where the first term in the sum involves the variance of the magnitude difference $|{\hat{A}}_{ak}^{\left(i\right)}-{\hat{A}}_{ak}^{\left(j\right)}|$ and then second term involves the cross-covariance of the rs-fMRI diff for distinct pairwise ROI-ROI associations. This formulation is implied in our previous derivation of the variance, but our independence assumption allowed us to assume that all terms in the second part of the two-part sum were zero. Our formulation of the variance is given by the following

$$\begin{array}{c} \begin{array}{cc} \text{Var}\left(D_{ij}^{\text{fMRI}}\right) & =\text{Var}\left(\sum_{a\in A}\sum_{k\neq a}|{\hat{A}}_{ak}^{\left(i\right)}-{\hat{A}}_{ak}^{\left(j\right)}|\right)\\ & =\sum_{a=1}^{p-1}\text{Var}\left(\sum_{k=a+1}^{p}2|{\hat{A}}_{ak}^{\left(i\right)}-{\hat{A}}_{ak}^{\left(j\right)}|\right)\\ & +2\sum_{a=1}^{p-1}\sum_{r=a+1}^{p-1}\text{Cov}\left(\sum_{k=a+1}^{p}2|{\hat{A}}_{ak}^{\left(i\right)}-{\hat{A}}_{ak}^{\left(j\right)}|,\sum_{s=r+1}^{p}2|{\hat{A}}_{rs}^{\left(i\right)}-{\hat{A}}_{rs}^{\left(j\right)}|\right)\\ & =\sum_{a=1}^{p-1}\sum_{k=a+1}^{p}\text{Var}\left(2|{\hat{A}}_{ak}^{\left(i\right)}-{\hat{A}}_{ak}^{\left(j\right)}|\right)\\ & +2\sum_{a=1}^{p-1}\sum_{r=a+1}^{p-1}\text{Cov}\left(\sum_{k=a+1}^{p}2|{\hat{A}}_{ak}^{\left(i\right)}-{\hat{A}}_{ak}^{\left(j\right)}|,\sum_{s=r+1}^{p}2|{\hat{A}}_{rs}^{\left(i\right)}-{\hat{A}}_{rs}^{\left(j\right)}|\right)\\ & =\sum_{a=1}^{p-1}\sum_{k=a+1}^{p-1}\frac{4\left(\pi -2\right)}{\pi }\\ & +2\sum_{a=1}^{p-1}\sum_{r=a+1}^{p-1}\text{Cov}\left(\sum_{k=a+1}^{p}2|{\hat{A}}_{ak}^{\left(i\right)}-{\hat{A}}_{ak}^{\left(j\right)}|,\sum_{s=r+1}^{p}2|{\hat{A}}_{rs}^{\left(i\right)}-{\hat{A}}_{rs}^{\left(j\right)}|\right)\\ & =\frac{2p\left(\pi -2\right)\left(p-1\right)}{\pi }\\ & +2\sum_{a=1}^{p-1}\sum_{r=a+1}^{p-1}\text{Cov}\left(\sum_{k=a+1}^{p}2|{\hat{A}}_{ak}^{\left(i\right)}-{\hat{A}}_{ak}^{\left(j\right)}|,\sum_{s=r+1}^{p}2|{\hat{A}}_{rs}^{\left(i\right)}-{\hat{A}}_{rs}^{\left(j\right)}|\right). \end{array} \end{array}$$ (161)

In order to have a formula in terms of the number of ROIs *p* only, we estimate the double sum on the right-hand side of the equation of rs-fMRI distance variance (Eq 161). Through simulation, it can be seen that the difference between the actual sample variance $S_{D_{ij}}^{2}$ and the corresponding variance under the independence assumption $\frac{2p\left(\pi -2\right)\left(p-1\right)}{\pi }$ has a quadratic relationship with *p*. More explicitly, we have the following relationship

$$\begin{array}{c} S_{D_{ij}^{\text{fMRI}}}^{2}-\frac{2p\left(\pi -2\right)\left(p-1\right)}{\pi }=\beta_{1}p^{2}+\beta_{0}p. \end{array}$$ (162)

where *β*_0_ and *β*_1_ are the coefficients we must estimate in order to approximate the cross-covariance term in the right-hand side of the rs-fMRI distance variance equation (Eq 161).

The coefficient estimates found through least squares fitting are *β*_1_ = −*β*_0_ ≈ 0.08. These estimates allow us to arrive at a functional form for the double sum in the right-hand side of the rs-fMRI distance variance equation (Eq 161) that is proportional to $\frac{2p\left(\pi -2\right)\left(p-1\right)}{\pi }$. That is, we have the following formula for approximating the double sum

$$\begin{array}{c} 2\sum_{a=1}^{p-1}\sum_{r=a+1}^{p-1}\text{Cov}\left(\sum_{k=a+1}^{p}2|{\hat{A}}_{ak}^{\left(i\right)}-{\hat{A}}_{ak}^{\left(j\right)}|,\sum_{s=r+1}^{p}2|{\hat{A}}_{rs}^{\left(i\right)}-{\hat{A}}_{rs}^{\left(j\right)}|\right)\approx \frac{p\left(\pi -2\right)\left(p-1\right)}{4\pi }. \end{array}$$ (163)

Therefore, the variance of the rs-fMRI distances is approximated well by the following

$$\begin{array}{c} \text{Var}\left(D_{ij}^{\text{fMRI}}\right)\approx \frac{9p\left(\pi -2\right)\left(p-1\right)}{4\pi }. \end{array}$$ (164)

With the mean (Eq 159) and variance (Eq 164) estimates, we have the following asymptotic distribution for rs-fMRI distances

$$\begin{array}{c} D_{ij}^{\text{fMRI}}\dot{∼}N\left(\frac{2p\left(p-1\right)}{\sqrt{\pi }},\frac{9p\left(\pi -2\right)\left(p-1\right)}{4\pi }\right). \end{array}$$ (165)

### 6.1 Max-min normalized time series correlation-based distance distribution

Previously (Section 4) we determined the asymptotic distribution of the sample maximum of size *m* from a standard normal distribution. We can naturally extend these results to our transformed rs-fMRI data because *X* (Fig 9) is approximately standard normal. Furthermore, we have previously mentioned that the max-min normalized *L*_*q*_ metric yields approximately normal distances with the iid assumption. We show a similar result for max-min normalized rs-fMRI distances (S8 Fig in S1 File). We proceed with the definition of the max-min normalized rs-fMRI pairwise distance.

Consider the max-min normalized rs-fMRI distance given by the following equation

$$\begin{array}{c} D_{ij}^{\text{fMRI}*}=\sum_{a\in A}\sum_{k\neq a}\frac{\left|A_{ak}^{\left(i\right)}-A_{ak}^{\left(j\right)}\right|}{\max\left(a\right)-\min\left(a\right)}. \end{array}$$ (166)

Assuming that the data *X* has been r-to-z transformed and standardized, we can easily compute the expected attribute range and variance of the attribute range. The expected maximum of a given attribute in data matrix *X* is estimated by the following

$$\begin{array}{c} \text{E}\left(X_{a}^{\text{max}}-X_{a}^{\text{min}}\right)=2\mu_{\text{max}}^{\left(1\right)}\left(m,p\right)=2[\frac{\text{log}\left(\text{log}\left(2\right)\right)}{\Phi^{-1}\left(\frac{1}{m\left(p-1\right)}\right)}-\Phi^{-1}\left(\frac{1}{m\left(p-1\right)}\right)]. \end{array}$$ (167)

The variance can be esimated with the following

$$\begin{array}{c} \text{Var}\left(X_{a}^{\text{max}}-X_{a}^{\text{min}}\right)=\frac{\pi^{2}}{6\text{log}[m\left(p-1\right)]}. \end{array}$$ (168)

Let $\mu_{D_{ij}^{\text{fMRI}}}$ and $\sigma_{D_{ij}^{\text{fMRI}}}^{2}$ denote the mean and variance of the rs-fMRI distance distribution given by Eqs 159 and 164. Using the formulas for the mean and variance of the max-min normalized distance distribution given in Eq 92, we have the following asymptotic distribution for the max-min normalized rs-fMRI distances

$$\begin{array}{c} D_{ij}^{\text{fMRI}*}\dot{∼}N\left(\frac{\mu_{D_{ij}^{\text{fMRI}}}}{2\mu_{\text{max}}^{\left(1\right)}\left(m,p\right)},\frac{6\sigma_{D_{ij}^{\text{fMRI}}}^{2}\text{log}\left[m\left(p-1\right)\right]}{\pi^{2}+24{\left[\mu_{\text{max}}^{\left(1\right)}\left(m,p\right)\right]}^{2}\text{log}\left[m\left(p-1\right)\right]}\right). \end{array}$$ (169)

### 6.2 One-dimensional projection of rs-fMRI distance onto a single ROI

Just as in previous sections (Sections. 3.2.3 and 5.4), we now derive the distribution of our rs-fMRI diff metric (Eq 157). Unlike what we have seen in previous sections, we do not derive the exact distribution for this diff metric. We have determined empirically that the rs-fMRI diff is approximately normal. Although the rs-fMRI diff is a sum of *p* − 1 magnitude differences, the Classical Central Limit Theorem does not apply because of the dependencies that exist between the terms of the sum. Examination of histograms and quantile-quantile plots of simulated values of the rs-fMRI diff easily indicate that the normality assumption is safe. Therefore, we derive the mean and variance of the approximately normal distribution of the rs-fMRI diff. As we have seen previously, this normality assumption is reasonable even for small values of *p*.

The mean of the rs-fMRI diff is derived by fixing a single ROI *a* and considering all pairwise associations with other ROIs k≠A. This is done as follows

$$\begin{array}{c} \begin{array}{cc} \text{E}[{\text{d}}_{ij}^{\text{ROI}}\left(a\right)] & =\text{E}\left(\sum_{k\neq a}|{\hat{A}}_{ak}^{\left(i\right)}-{\hat{A}}_{ak}^{\left(j\right)}|\right)\\ & =\sum_{k\neq a}\text{E}\left(|{\hat{A}}_{ak}^{\left(i\right)}-{\hat{A}}_{ak}^{\left(j\right)}|\right)\\ & =\sum_{k\neq a}\frac{2}{\sqrt{\pi }}\\ & =\frac{2\left(p-1\right)}{\sqrt{\pi }}, \end{array} \end{array}$$ (170)

where *a* is a single fixed ROI.

Considering the variance of the rs-fMRI diff metric, we have two estimates. The first estimate uses the variance operator in a linear fashion, while the second will simply be a direct implication of the corrected formula of the variance of rs-fMRI pairwise distances (Eq 164). Our first estimate is derived as follows

$$\begin{array}{c} \begin{array}{cc} \text{Var}[{\text{d}}_{ij}^{\text{ROI}}\left(a\right)] & =\text{Var}\left(\sum_{k\neq a}|{\hat{A}}_{ak}^{\left(i\right)}-{\hat{A}}_{ak}^{\left(j\right)}|\right)\\ & =\sum_{k\neq a}\text{Var}\left(|{\hat{A}}_{ak}^{\left(i\right)}-{\hat{A}}_{ak}^{\left(j\right)}|\right)\\ & =\sum_{k\neq a}\frac{2\left(\pi -2\right)}{\pi }\\ & =\frac{2\left(\pi -2\right)\left(p-1\right)}{\pi }, \end{array} \end{array}$$ (171)

where *a* is a single fixed ROI.

Using the corrected rs-fMRI distance variance formula (Eq 164), our second estimate of the rs-fMRI diff variance is given directly by the following

$$\begin{array}{c} \text{Var}[{\text{d}}_{ij}^{\text{ROI}}\left(a\right)]=\frac{9\left(\pi -2\right)\left(p-1\right)}{4\pi }, \end{array}$$ (172)

where *a* is a single fixed ROI.

Empirically, the first estimate (Eq 171) of the variance of our rs-fMRI diff is closer to the sample variance than the second estimate (Eq 172). This is due to fact that we are considering only a fixed ROI a∈A, so the cross-covariance between the magnitude differences $|{\hat{A}}_{ak}^{\left(i\right)}-{\hat{A}}_{ak}^{\left(j\right)}|$ for different pairs of ROIs (*a* and *k* ≠ *a*) is negligible here. When considering all ROIs a∈A, these cross-covariances are no longer negligible. Using the first variance estimate (Eq 171) and the estimate of the mean (Eq 170), we have the following asymptotic distribution of the rs-fMRI diff

$$\begin{array}{c} {\text{d}}_{ij}^{\text{ROI}}\left(a\right)\dot{∼}N\left(\frac{2\left(p-1\right)}{\sqrt{\pi }},\frac{2\left(\pi -2\right)\left(p-1\right)}{\pi }\right), \end{array}$$ (173)

where *a* is a single fixed ROI. We compare moment estimates for the rs-fMRI diff (Eqs 170 and 171) with sample moments from simulated data with *m* = 100 samples and *p* = 1000, 2000, …, 5000 attributes (S21 Fig in S1 File). Our estimates follow the sample moments from simulated data very closely.

### 6.3 Normalized Manhattan (*q* = 1) for rs-fMRI

Substituting the non-normalized mean (Eq 159) into the equation for the mean of the max-min normalized rs-fMRI metric (Eq 169), we have the following

$$\begin{array}{c} \begin{array}{cc} \text{E}\left(D_{ij}^{\text{fMRI}*}\right) & =\frac{\mu_{D_{ij}^{\text{fMRI}}}}{2\mu_{\text{max}}^{\left(1\right)}\left(m,p\right)}\\ & =\frac{p\left(p-1\right)}{\sqrt{\pi }\mu_{\text{max}}^{\left(1\right)}\left(m,p\right)}, \end{array} \end{array}$$ (174)

where $\mu_{\text{max}}^{\left(1\right)}\left(m,p\right)$ (Eq 167) is the expected maximum of a single ROI in a data set with *m* instances and *p* ROIs.

Similarly, the variance of $D_{ij}^{\text{fMRI}*}$ is given by

$$\begin{array}{c} \begin{array}{cc} \text{Var}\left(D_{ij}^{\text{fMRI}*}\right) & =\frac{6\sigma_{D_{ij}^{\text{fMRI}}}^{2}\text{log}\left[m\left(p-1\right)\right]}{\pi^{2}+24{\left[\mu_{\text{max}}^{\left(1\right)}\left(m,p\right)\right]}^{2}\text{log}\left[m\left(p-1\right)\right]}\\ & =\frac{27\left(\pi -2\right)\text{log}[m\left(p-1\right)]\left(p-1\right)p}{2\pi \left(\pi^{2}+24{\left[\mu_{\text{max}}^{\left(1\right)}\left(m,p\right)\right]}^{2}\text{log}\left[m\left(p-1\right)\right]\right)}, \end{array} \end{array}$$ (175)

where $\mu_{\text{max}}^{\left(1\right)}\left(m,p\right)$ (Eq 167) is the expected maximum of a single ROI in a data set with *m* instances and *p* ROIs.

**Fig 10:**
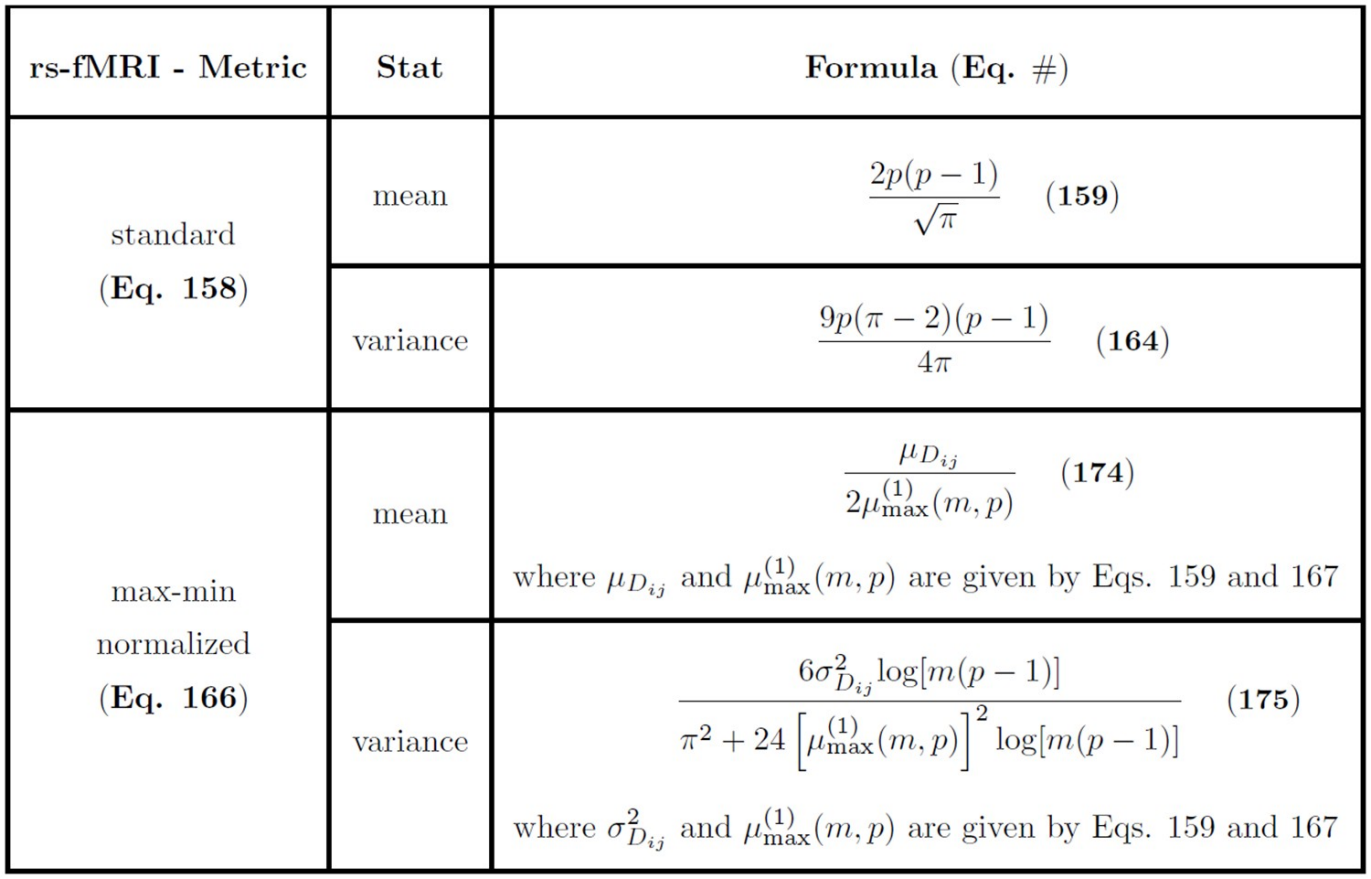
Aymptotic means and variances for the new standard (Eq 158) and max-min normalized (Eq 166) rs-fMRI distance metrics.

We summarize the moment estimates for the rs-fMRI metrics for correlation-based data derived from time series (Fig 10). We organize this summary by standard and attribute range-normalized rs-fMRI distance metric, statistic (mean or variance), and asymptotic formula.

## 7 Comparison of theoretical and sample moments

**Fig 11:**
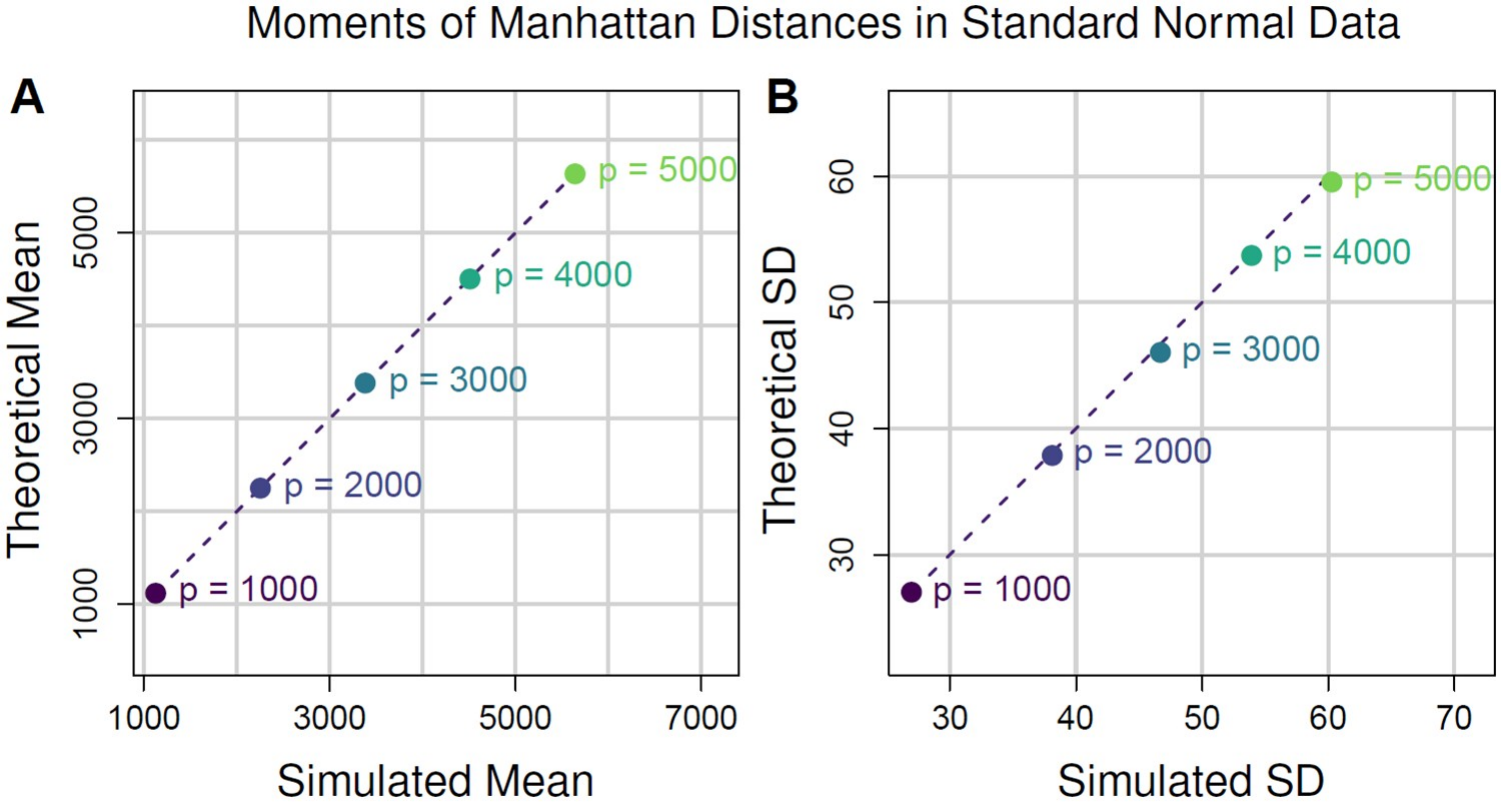
Comparison of theoretical and sample moments of Manhattan (Eq 1) distances in standard normal data. (A) Scatter plot of theoretical vs simulated mean Manhattan distance (Eq 41). Each point represents a different number of attributes *p*. For each value of *p* we fixed *m* = 100 and generated 20 distance matrices from standard normal data and computed the average simulated pairwise distance from the 20 iterations. The corresponding theoretical mean was then computed for each value of *p* for comparison. The dashed line represents the identity (or *y* = *x*) line for reference. (**B**) Scatter plot of theoretical vs simulated standard deviation of Manhattan (Eq 1) distance (Eq 42). These standard deviations come from the same random distance matrices for which mean distance was computed for **A**. Both theoretical mean and standard deviation approximate the simulated moments quite well.

We compare our analytical asymptotic estimates of sample moments for distributions of pairwise distances in high attribute dimension by generating random data for various dimensions *m* and *p* (Fig 11). We fix *m* = 100 samples and compute Manhattan (Eq 1) distance matrices from standard normal data for *p* = 1000, 2000, 3000, 4000, and 5000 attributes. For each value of *p*, we generate 20 random datasets and compute the mean and standard deviation of pairwise distances. We then average these 20 simulated means and standard deviations. For comparison, we compute the theoretical moments (Eqs 41 and 42) for each value of *p* and fixed *m* = 100 from the theoretical formulas. Scatter plots of theoretical versus simulated mean (Fig 11A) and theoretical versus simulated standard deviation (Fig 11B) indicate that our theoretical asymptotic formulas for sample moments are reliable for both large and relatively small numbers of attributes. For other combinations of data type, distance metric, sample size *m*, and number of attributes *p*, we find similar agreement between theoretical formulas and simulated moments (S9-S21 Figs in S1 File).

## 8 Effects of correlation on distances

All of the derivations presented in previous sections were for the cases where there is no correlation between instances or between attributes. We assumed that any pair (*X*_*ia*_, *X*_*ja*_) of data points for instances *i* and *j* and fixed attribute *a* were independent and identically distributed. This was assumed in order to determine asymptotic estimates in null data. That is, data with no main effects, interaction effects, or pairwise correlations between attributes. Within this simplified context, our asymptotic formulas for distributional moments are reliable. However, in real data are numerous statistical effects that impact distance distributional properties. That being said, we have shown that for Manhattan distances generated on real gene expression microarray data (S26-S124 Figs in S1 File) and distances generated with our new metric (Eq 158) on real rs-fMRI data (S125-S126 Figs in S1 File) that the normality assumption is approximately satisfied in many cases. In simulated data, we find that deviation from normality is caused primarily by large magnitude pairwise correlation between attributes. Pairwise attribute correlation can be the result of main effects, where attributes have different within-group means. On the other hand, there could be an underlying interaction network in which there are strong associations between attributes. If attributes are differentially correlated between phenotype groups, then interactions exist that change the distance distribution. In the following few sections, we consider particular cases of the *L*_*q*_ metric for continuous and discrete data under the effects of pairwise attribute correlation.

### 8.1 Continuous data

**Fig 12:**
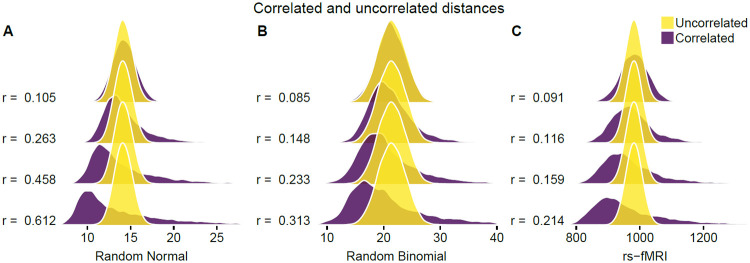
Distance densities from uncorrelated vs correlated bioinformatics data. (**A**) Euclidean distance densities for random normal data with and without correlation. Correlated data was created by multiplying random normal data by upper-triangular Cholesky factor from randomly generated correlation matrix. We created correlated data for average absolute pairwise correlation (Eq 176) ${\bar{r}}_{\text{abs}}=0.105,0.263,0.458,\;\text{and}\;0.612$. (**B**) TiTv distance densities for random binomial data with and without correlation. Correlated data was created by first generating correlated standard normal data using the Cholesky method from (A). Then we applied the standard normal CDF to create correlated uniformly distributed data, which was then transformed by the inverse binomial CDF with *n* = 2 trials and success probabilites $f_{a}\;\text{for}\;\text{all}\;a\in A$. (**C**) Time series correlation-based distance densities for random rs-fMRI data (Fig 9) with and without additional pairwise feature correlation. Correlation was added to the transformed rs-fMRI data matrix (Fig 9) using the Cholesky algorithm from (A).

Without loss of generality, suppose we have *X*^(*m*×*p*)^ where $X_{ia}∼N\left(0,1\right)$ for all *i* = 1, 2, …, *m* and *a* = 1, 2, …, *p*, and let *m* = *p* = 100. We consider only the *L*_2_ (Euclidean) metric (Eq 1, *q* = 2). We explore the effects of correlation on these distances by generating simulated data sets with increasing strength of pairwise attribute correlation and then plotting the density curve of the induced distances (Fig 12A). Deviation from normality in the distance distribution is directly related to the average absolute pairwise correlation that exists in the simulated data. This measure is given by

$$\begin{array}{c} {\bar{r}}_{\text{abs}}=\frac{2}{p\left(p-1\right)}\sum_{i=1}^{p-1}\sum_{j>i}r_{ij} \end{array}$$ (176)

where *r*_*ak*_ is the correlation between attributes a,k∈A across all instances *m*. Distances generated on data without correlation closely approximate a Gaussian. The mean (Eq 53) and variance (Eq 52) of the uncorrelated distance distribution are given by substituting *p* = 100 for the mean. As ${\bar{r}}_{\text{abs}}$ increases, positive skewness and increased variability in distances emerges. The predicted and sample means, however, are approximately the same between correlated and uncorrelated distances due to linearity of the expectation operator. Because of the dependencies between attributes, the predicted variance of 1 for *L*_2_ on standard normal data obviously no longer holds.

In order to introduce a controlled level of correlation between attributes, we created correlation matrices based on a random graph with specified connection probability, where attributes correspond to the vertices in each graph. We assigned high correlations to connected attributes from the random graph and low correlations to all non-connections. Using the upper-triangular Cholesky factor *U* for uncorrelated data matrix *X*, we computed the following product to create correlated data matrix *X*^corr^

$$\begin{array}{c} X^{\text{corr}}=XU^{\text{T}}. \end{array}$$ (177)

The new data matrix *X*^corr^ has approximately the same correlation structure as the randomly generated correlation matrix created from a random graph.

### 8.2 GWAS data

Analogous to the previous section, we explore the effects of pairwise attribute correlation in the context of GWAS data. Without loss of generality, we let *m* = *p* = 100 and consider only the TiTv metric (Eq 115). To create correlated GWAS data, we first generated standard normal data with random correlation structure, just as in the previous section. We then applied the standard normal cumulative distribution function (CDF) to this correlated data in order transform the correlated standard normal variates into uniform data with preserved correlation structure. We then subsequently applied the inverse binomial CDF to the correlated uniform data with random success probabilities $f_{a}\;\text{for}\;\text{all}\;a\in A$. Each attribute a∈A corresponds to an individual SNP in the data matrix. The resulting GWAS data set is binomial with *n* = 2 trials and has roughly the same correlation matrix as the original correlated standard normal data with which we started. Average absolute pairwise correlation ${\bar{r}}_{\text{abs}}$ induces positive skewness in GWAS data at lower levels than in correlated standard normal data (Fig 12B). This could have important implications in nearest neighborhoods in NPDR and similar methods.

### 8.3 Time-series derived correlation-based datasets

For our correlation data-based metric (Eq 158), we consider additional effects of correlation between features. Without loss of generality, we let *m* = 100 and *p* = 30. We show an illustration of the effects of correlated features in this context (Fig 12C). Based on the density estimates, it appears that correlation between features introduces positive skewness at low values of ${\bar{r}}_{\text{abs}}$. We introduced correlation to the transformed data matrix (Fig 9) with the cholesky method used previously.

## 9 Feature selection with distance distribution-informed nearest neighbors

Our derivation of asymptotic moments of distance distributions has been motivated by the need to improve performance of feature selection in nearest-neighbor algorithms. The choice of *k* or a neighborhood radius can have a large impact on selected features [5]. Historically, the general rule-of-thumb for fixed *k* was *k* = 10. However, this rule-of-thumb does not adapt to properties of the data, such as sample size *m* or number of features *p*. As we have shown for random data with uncorrelated attributes, mean distance or standard deviation of sample distances increases in direct proportion to some function of *p*. As a result, the rule-of-thumb can be out of step with the average distance between neighbors in a real data set. Parameterizing the neighborhood sizes by the expected moments of the distance distribution, under the assumption of independent data and uncorrelated features, can improve upon naive neighborhood approaches.

The adaptive radius method MultiSURF outperformed fixed *k* methods for detecting interaction effects in simulated data [1]. In another simulation study, it was shown that MultiSURF performed relatively well in detecting both interaction effects and main effects [5]. The MultiSURF approach gives each target instance *i* its own tailored neighborhood radius *R*_*i*_ (Eq 178) as a function of the average pairwise distance to the target instance *i* (${\bar{D}}_{ij\neq i}$) and the sample standard deviation of the same distances (*D*_*ij* ≠ *i*_).

$$\begin{array}{c} R_{i}={\bar{D}}_{ij\neq i}-\alpha \sigma_{D_{ij\neq i}}, \end{array}$$ (178)

where ${\bar{D}}_{ij\neq i}={\left(m-1\right)}^{-1}\sum_{j\neq i}D_{ij}$, $\sigma_{D_{ij\neq i}}=\sqrt{\text{Var}\left(D_{ij\neq i}\right)}$, and *α* = 0.5.

The study in Ref. [5] showed that fixed *k* = ⌊*m*/6⌋ = 16 empirically gave approximately the same neighborhood size as MultiSURF on average, but the fixed *k* = ⌊*m*/6⌋ = 16 method modestly improved the detection of main effects and performed approximately the same for interaction effects in 100 replicated simulations. Furthermore, in Ref. [4], the approximation of *k* = ⌊*m*/6⌋ to the average MultiSURF radius (Eq 178) neighborhood order, although very accurate for *m* = 100, was more precisely shown to be

$$\begin{array}{c} {\bar{k}}_{\alpha }=\lfloor \frac{m-1}{2}\left(1-\text{erf}\left(\frac{\alpha }{\sqrt{2}}\right)\right)\rfloor , \end{array}$$ (179)

where *α* = 0.5 for MultiSURF. This formula for ${\bar{k}}_{\alpha }$ (Eq 179) is simply the transformation ⌊⋅⌋ of the expected value of a binomial random variable with *n* = *m* − 1 trials and success probability $q_{\alpha }=0.5\left(1-\text{erf}\left(\alpha /\sqrt{2}\right)\right)$. The value of *q*_*α*_ is the probability of a random instance *j* ≠ *i* being in the neighborhood of target instance *i*, which is equivalent to satisfying *D*_*ij* ≠ *i*_ ≤ *R*_*i*_ (Eq 178). When we take *α* = 0.5, we find that ${\bar{k}}_{1/2}\approx \left\lfloor 0.154\left(m-1\right)\right\rfloor =15$, which differs from the empirically determined *k* = 16 by only a single neighbor.

**Fig 13:**
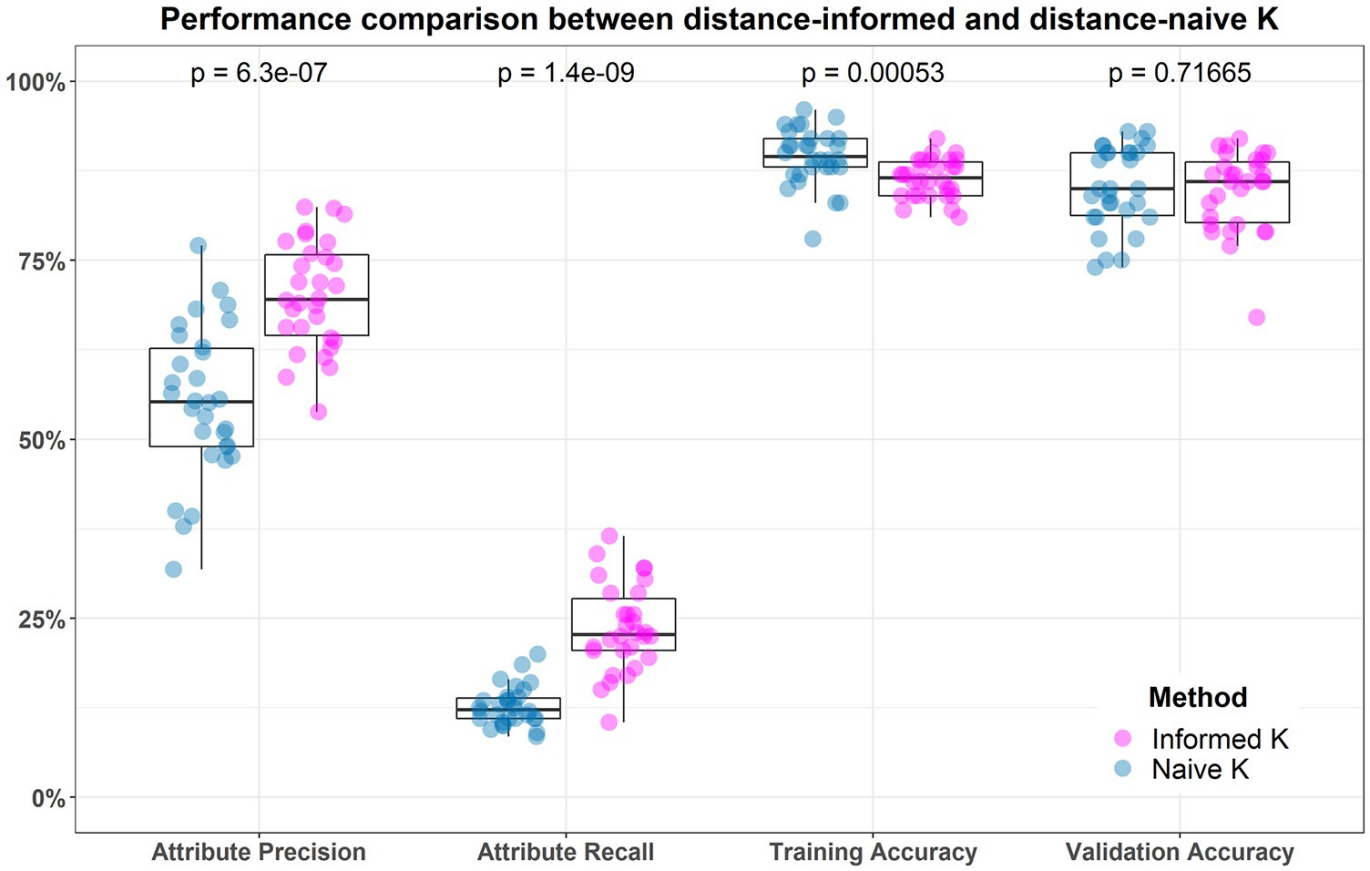
Simulation comparison between rule-of-thumb naive *k* = 10 and distance-distribution informed *k*_*α* = 1/2_. Precision and recall for the functional features are significantly improved using informed *k* versus naive *k* = 10. The training and validation classification accuracy are similar for the two values of *k* with slightly less overfitting for informed-k.

We compare the performance of Relief nearest-neighbor feature selection with data-informed *k*_*α* = 1/2_ and the rule-of-thumb *k* = 10 (Fig 13). We use consensus features nested cross-validation (cnCV) to perform feature and model selection while avoiding overfitting [28]. The cnCV approach has been shown to select fewer false positive features on average across all simulation replicates than standard nested cross-validation while simultaneously maintaining a low false negative rate for functional features. Our application of cnCV (cncv https://github.com/insilico/cncv) uses the Relief nearest-neighbor method for feature selection and random forest for classification, which was parameterized by ntree = 1000 trees and mtry = *p*_f_/3 randomly selected features at each node split. The value *p*_f_ is the total features in a given training fold.

For the comparison, we simulate data with an underlying interaction network, where interacting features have no main effects [29], and then we add main effect features. Each simulated data set has *p* = 1000 attributes, where 100 are functional, and *m* = 100 instances (50 cases and 50 controls). For statistical comparison, we create 30 replicate simulations, and each simulated data set is split into a training and a validation set for indepedent assessment.

The distance distribution informed-*k*_*α* = 1/2_ shows a statistically significantly advantage over naive *k* = 10 for feature selection performance (left two plots of Fig 13). The training and validation accuracy are very similar and very high for both types of *k*. The training accuracy is slightly higher for naive *k* = 10, but there is more of a drop in its validation accuracy, which suggests possible overfitting. The validation accuracy for informed *k*_*α* = 1/2_ is closer to its training accuracy, which suggests that its training accuracy is a better estimation of the true accuracy.

## 10 Discussion

Nearest-neighbor distance-based feature selection is a class of methods that are relatively simple to implement, and they perform well at detecting interaction effects in high dimensional data. Theoretical analysis of the limiting behavior of distance distributions for various data types and dimensions may lead to improved hyperparameter estimates of these feature selection methods. Furthermore, these theoretical results may help guide the choice of distance metric for a given dataset. Most often, distance-based feature selection methods use the *L*_*q*_ metric (Eq 1) with *q* = 1 or *q* = 2. However, these two realizations of the *L*_*q*_ metric have considerably different behavior for the mean and variance of their respective limiting distributions. For instance, the expected distance for *L*_1_ and *L*_2_ for standard normal data is proportional to *p* (Eq 41 and Fig 3) and $\sqrt{p}$ (Eq 51 and Fig 3), respectively. In addition, *L*_1_ and *L*_2_ on standard normal data have asymptotic variances on the order of *p* and 1, respectively (Eqs 42 and 52).

These results can inform the choice of *L*_1_ or *L*_2_ depending on context. For instance, distances become harder to distinguish from one another in high dimensions, which is one of the curses of dimensionality. In the case of *L*_2_, the asymptotic distribution ($N\left(\sqrt{2p-1},1\right)$) indicates that the limiting *L*_2_ distribution can be thought of simply as a positive translation of the standard normal distribution (N(0,1)). The *L*_2_ distribution also indicates that most neighbors are contained in a thin shell far from the instance in high dimension (*p* ≫ 1). On the other hand, the *L*_1_ distances become more dispersed due to the fact that the variance of the limiting distribution is proportional to the attribute dimension *p* (variance is 2(*π* − 2)*p*/*π* and mean is $2p/\sqrt{\pi }$). This variance for *L*_1_ could be more desirable when determining nearest neighbors because instances may be easier to distinguish with this metric. If using *L*_1_, then it may be best to use a fixed-k algorithm instead of fixed-radius because fixed-radius neighborhood size could vary quite a bit (variance proportional to attribute dimension *p*), which in turn could affect the quality of selected attributes. If *L*_2_ is being used, then either fixed-k or fixed-radius may perform equally well because most distances will be within 1 standard deviation away from the mean.

We derived distance asymptotics for some of the most commonly used metrics in nearest-neighbor distance-based feature selection, as well as two new metrics for GWAS (Eq 115) and a new metric for time-series correlation-based data (Eqs 158 and 166) like resting-state fMRI. These are novel results that show the behavior of distances in random data. We also extended the asymptotic results of the standard *L*_*q*_ metrics to derive new estimates of the mean and variance of the attribute range-normalized *L*_*q*_ (max-min) distance for standard normal (Eq 92) and standard uniform (Eq 100) data using extreme value theory. Our derivations provide an important reference for those using nearest-neighbor feature selection or classification methods in common bioinformatics data. In particular, the range-normalized asymptotic results apply directly to Relief-based algorithms that use the range of each attribute to constrain its score to be within [−1, 1].

We derived the asymptotic mean and variance of the recently developed transition-transversion (TiTv) metric (Eq 112) for nearest-neighbor feature selection in GWAS data [30]. Our novel asymptotic estimates for the TiTv metric, as well as for the GM (Eq 110) and AM (Eq 111) metrics, provide an important reference to aid in neighborhood parameter selection for GWAS. We also showed how the Ti/TV ratio *η* (Eq 127) and minor allele frequency (or success probability) *f*_*a*_ affect these discrete distances. For the GM and AM metrics, the distance is solely determined by the minor allele frequencies because the genotype encoding is not taken into account. We showed how both minor allele frequency and Ti/Tv ratio uniquely affects the TiTv distance (Fig 7A and 7C). Because transversions are more disruptive forms of mutation than transitions, this additional dimension of information is important to consider, which is why we have provided asymptotic results for this metric.

We developed a new nearest-neighbor metric for time-series correlation-based data, motivated in part by feature selection for resting-state fMRI studies. The new metric (Eq 157) allows us to use regions of interest (ROIs) as attributes. Previously Relief-based methods would only compute the importance of ROI-ROI pairs based on differential correlation, but this new metric allows one to compute the individual contribution of each ROI. Nearest-neighbor feature selection would be a useful tool for case-control studies to determine important ROIs due to interactions and to help elucidate the network structure of the brain as it relates to the phenotype of interest. With our new rs-fMRI metric (Eq 158), we can apply NPDR or any other nearest neighbor feature selection algorithm to determine the importance of individual ROIs in classifying important phenotypes (e.g., major depressive disorder versus healthy controls).

In addition to asymptotic *L*_*q*_ distance distributions, we also provided the exact distributions for the one-dimensional projection of the *L*_*q*_ distance onto individual attributes (Sections. 3.2.3, 5.4, and 6.2). These distributions are important for all nearest-neighbor distance-based feature selection algorithms, such as Relief or NPDR, because the *L*_*q*_ distance is a function of the one-dimensional attribute projection (diff). In particular, these projected distance distributions are important for improving inference for predictors in NPDR, which are one-dimensional attribute projections.

Deviations from Gaussian for the distribution of the pairwise distances could be an indication of interaction or other statistical effects in the data. We explored Gaussianity of Manhattan distances in real gene expression microarrays (S26-S124 Figs in S1 File) and rs-fMRI data (S125, S126 Figs in S1 File). In most of the cases, we found distances are approximately normally distributed after standardizing samples to be zero mean and unit variance. One implication of this is that we can roughly predict how many neighbors to expect within a fixed radius about a given target instance. In the cases where the distribution deviates from Gaussian, an important future goal is to understand how the expected moments are modified. This will help us identify fixed-k neighborhoods for NPDR feature selection that avoid the potentially high variability of radius-based neighborhood sizes and increase the power to detect important statistical effects. Another future direction is to apply the asymptotic techniques to derive means and variances for other new metrics such as set-theoretic distance measures [31, 32].

In addition to interaction effects, correlation between attributes and instances can cause significant deviations from the asymptotic variances derived in this work, which assumed independence between variables. To illustrate this deviation, we showed how strong correlations lead to positive skewness in the distance distribution of random normal, binomial, and rs-fMRI data (Fig 12A, 12B and 12C). Pairwise correlation between attributes causes very little change to the average distance, so our mean asymptotic results for uncorrelated data also are good approximations when attributes are not independent. In contrast, the sample variance of distances deviates from the uncorrelated case substantially as the average absolute pairwise attribute correlation increases (Eq 176). For fixed or adaptive-radius neighborhood methods, this deviation can increase the probability of including neighbors for a given instance and may reduce the power to detect interactions. A future goal is to derive formulas for the variance of metrics that adjust for correlation in the data. The increased variance for distances with correlated data may inform the choice of metric and optimization of neighborhoods in nearest-neighbor feature selection.

## Supporting information

S1 File (PDF)
