# Supplementary material for: Theoretical properties of distance distributions and novel metrics for nearest-neighbor feature selection
Source: PLoS One. 2021 Feb 8;16(2):e0246761. doi: 10.1371/journal.pone.0246761 (PMC7870093; doi:10.1371/journal.pone.0246761)
Supplement: S1 File — (PDF) [file pone.0246761.s001.pdf]

Theoretical properties of distance distributions and novel metrics for nearest-neighbor feature selection: Supplementary figures

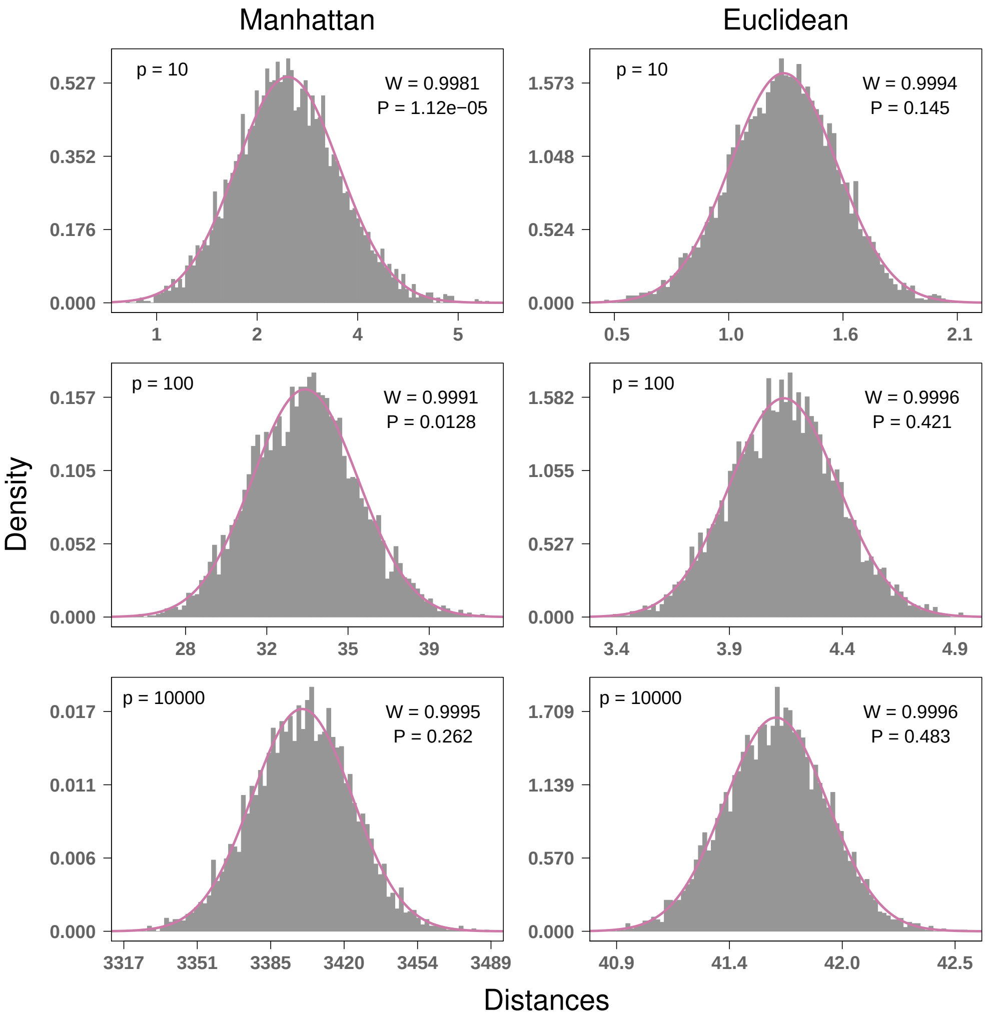

S1 Fig. Convergence to Gaussian for max-min normalized Manhattan and Euclidean distances for simulated standard uniform data with $m = 100$ instances and $p = 10, 100,$ and 10000 attributes.C onvergence to Gaussian occurs rapidly with increasing p, and Gaussian is a good approximation for p as low as 10 attributes. The number of attributes in bioinformatics data is typically much larger, at least on the order of 10^3. The Euclidean metric has stronger convergence to normal than Manhattan. P values from Shapiro-Wilk test, where the null hypothesis is a Gaussian distribution.

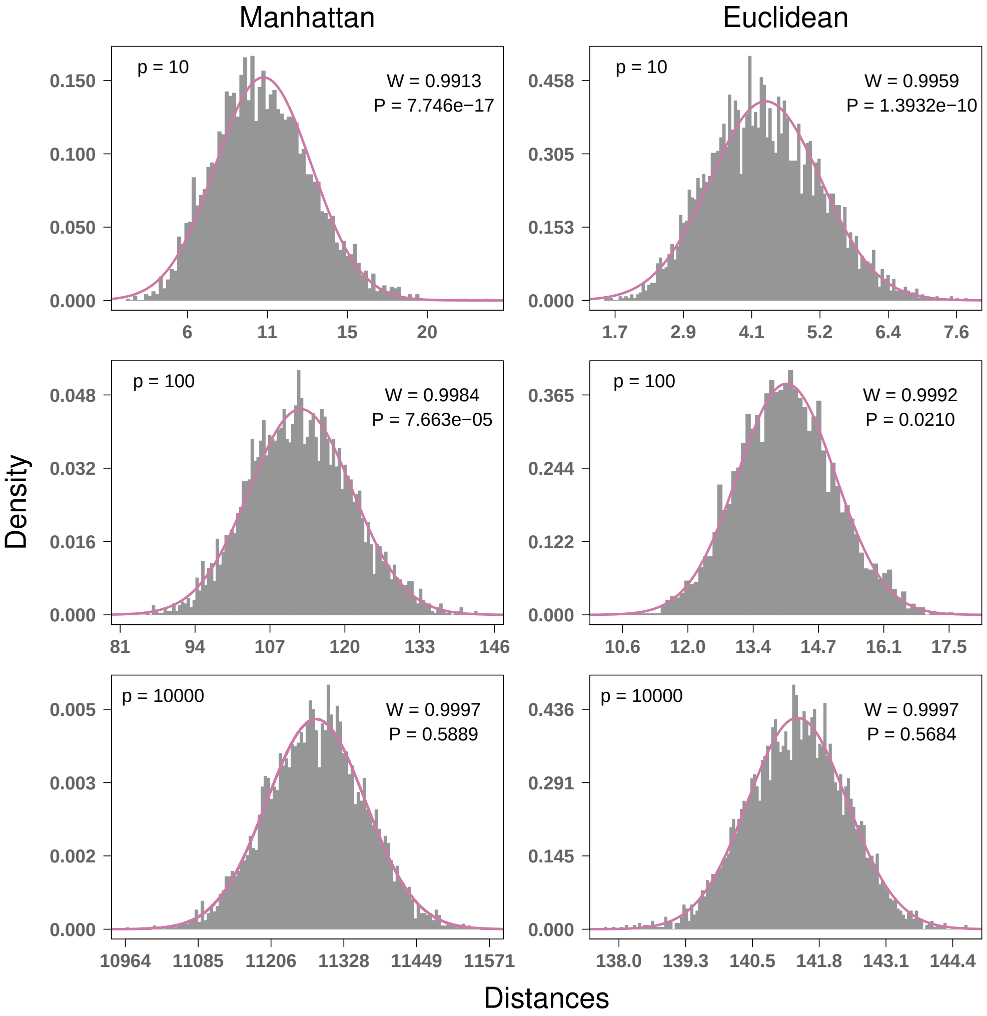

S2 Fig. Convergence to Gaussian for Manhattan and Euclidean distances for simulated standard normal data with $m = 100$ instances and $p = 10, 100$, and 10000 attributes. Convergence to Gaussian occurs rapidly with increasing p, and Gaussian is a good approximation for p as low as 10 attributes. The number of attributes in bioinformatics data is typically much larger, at least on the order of 10^3. The Euclidean metric has stronger convergence to normal than Manhattan. P values from Shapiro-Wilk test, where the null hypothesis is a Gaussian distribution.

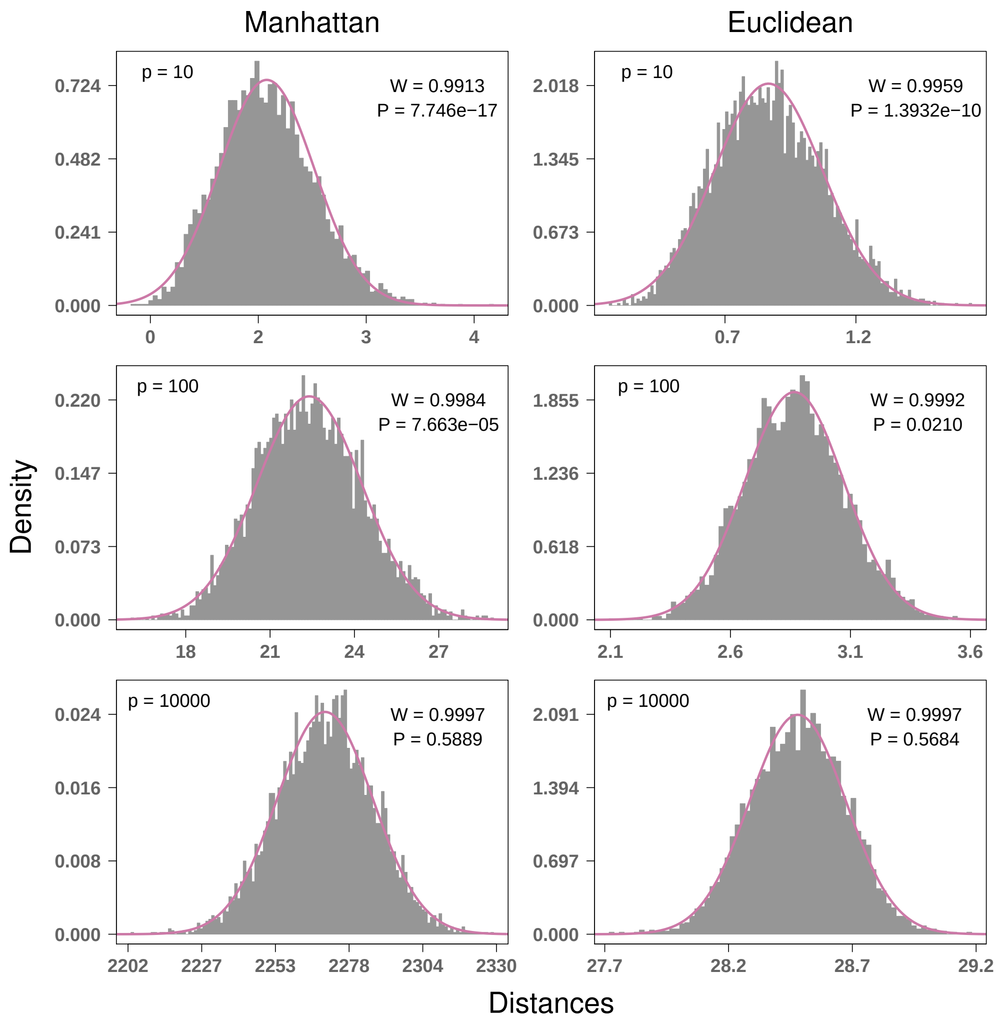

S3 Fig. Convergence to Gaussian for max-min normalized Manhattan and Euclidean distances for simulated standard normal data with $m = 100$ instances and $p = 10, 100$, and 10000 attributes. Convergence to Gaussian occurs rapidly with increasing p, and Gaussian is a good approximation for p as low as 10 attributes. The number of attributes in bioinformatics data is typically much larger, at least on the order of 10^3. The Euclidean metric has stronger convergence to normal than Manhattan. P values from Shapiro-Wilk test, where the null hypothesis is a Gaussian distribution.

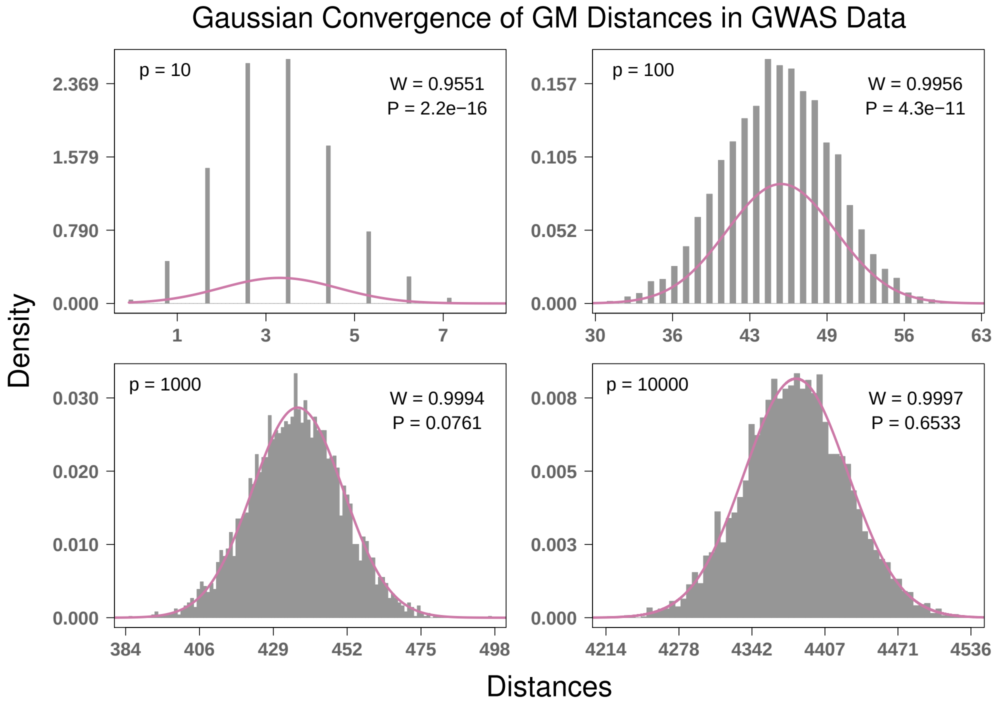

S4 Fig. Convergence to Gaussian for GM distances for simulated binomial GWAS data with $m = 100$ instances and $p = 10, 100, 1000$, and 10000 attributes. The average MAF was set to 0.205 for all simulations. Convergence to Gaussian occurs more gradually with increasing p than in continuous data. Significant convergence seems to occur when $p \geq 1000$, however, this is actually a relatively small number of features in the context of GWAS. Considering a realistic number of features for GWAS, the normality assumption of GM distances holds. This metric has the slowest convergence to Gaussian among all we have considered. P values from Shapiro-Wilk test, where the null hypothesis is a Gaussian distribution.

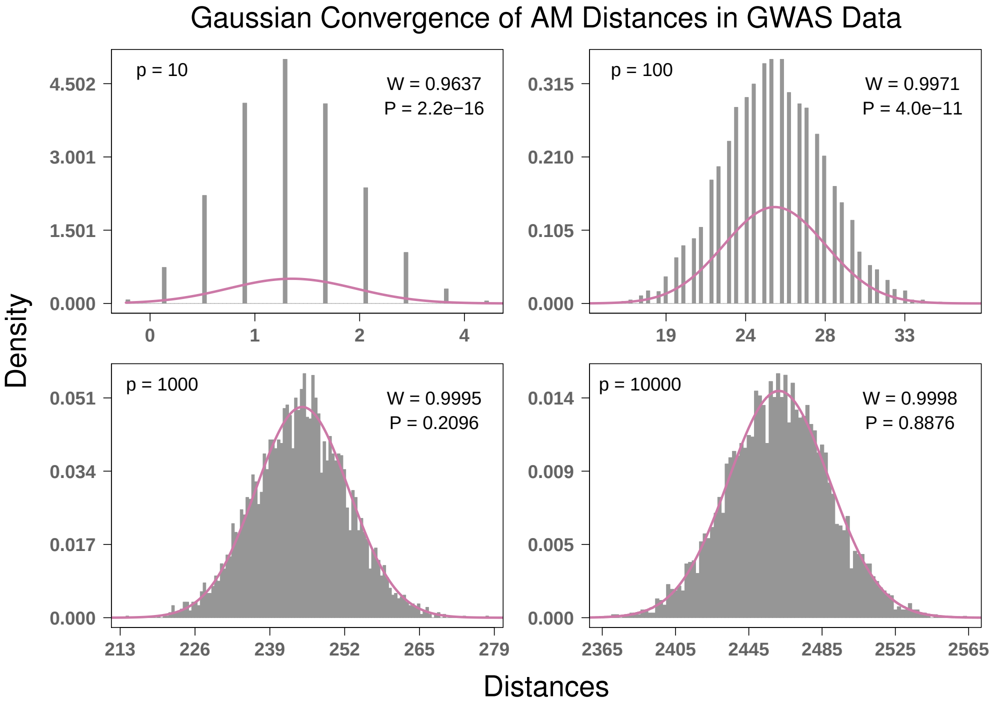

S5 Fig. Convergence to Gaussian for AM distances for simulated binomial GWAS data with $m = 100$ instances and $p = 10, 100, 1000$, and 10000 attributes. The average MAF was set to 0.205 for all simulations. Convergence to Gaussian occurs more gradually with increasing p than in continuous data. Significant convergence seems to occur when $p \geq 1000$, however, this is actually a relatively small number of features in the context of GWAS. Considering a realistic number of features for GWAS, the normality assumption of AM distances holds. This metric has the slightly faster convergence to Gaussian than the GM metric, which is probably due to the fact that the AM metric has one more value in its range (e.g., 1/2). P values from Shapiro-Wilk test, where the null hypothesis is a Gaussian distribution.

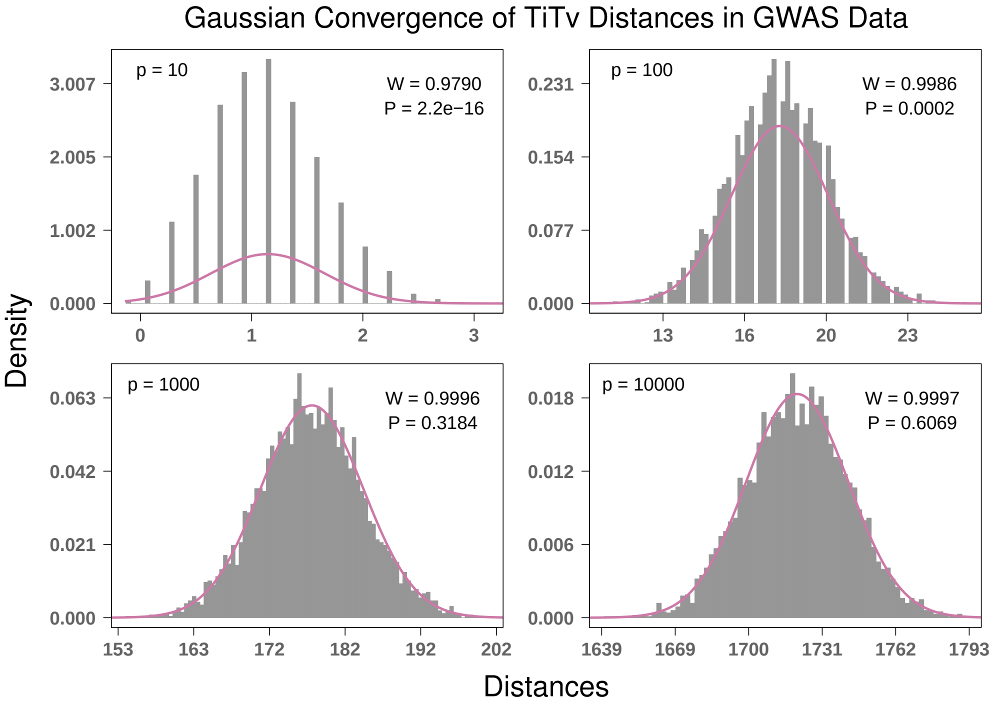

S6 Fig. Convergence to Gaussian for TiTv distances for simulated binomial GWAS data with $m = 100$ instances and $p = 10, 100, 1000$, and 10000 attributes. The average MAF was set to 0.205 for all simulations and the Ti/Tv ratio (η) was set to 2. Convergence to Gaussian occurs more gradually with increasing p than in continuous data. Significant convergence seems to occur when $p \geq 1000$, however, this is actually a relatively small number of features in the context of GWAS. Considering a realistic number of features for GWAS, the normality assumption of TiTv distances holds. This metric has the significantly faster convergence to Gaussian than the AM metric, which is probably due to the fact that the TiTv metric contains 2 more values in its range (e.g., 1/4 & 3/4). P values from Shapiro-Wilk test, where the null hypothesis is a Gaussian distribution.

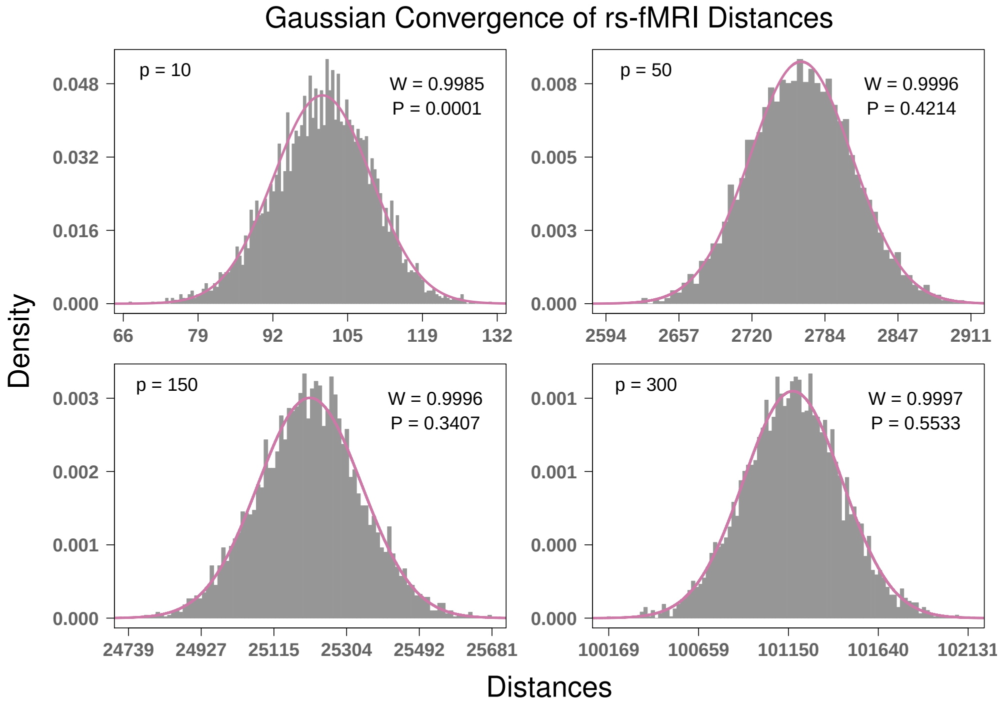

S7 Fig. Convergence to Gaussian for rs-fMRI distances for simulated correlation matrices with $m = 100$ instances and $p = 10, 50, 150$, and 300 attributes (or ROIs). Correlation matrices were generated for each instance from random normal $m \times p$ data matrices. Each correlation matrix was then stretched out into a long vector, Fisher r-to-z transformed, stored in a $p(p-1) \times m$ matrix, and standardized so that the m columns are mean 0 and unit variance. Convergence to Gaussian occurs very rapidly for this data because the dimensions are larger than a typical $m \times p$ data set. The large attribute dimension $p(p-1)$ means that there are significantly more terms in each sum to compute pairwise distances. Therefore, Classical Central Limit Theorem dictates that distances in this context will be closer to Gaussian. P values from Shapiro-Wilk test, where the null hypothesis is a Gaussian distribution.

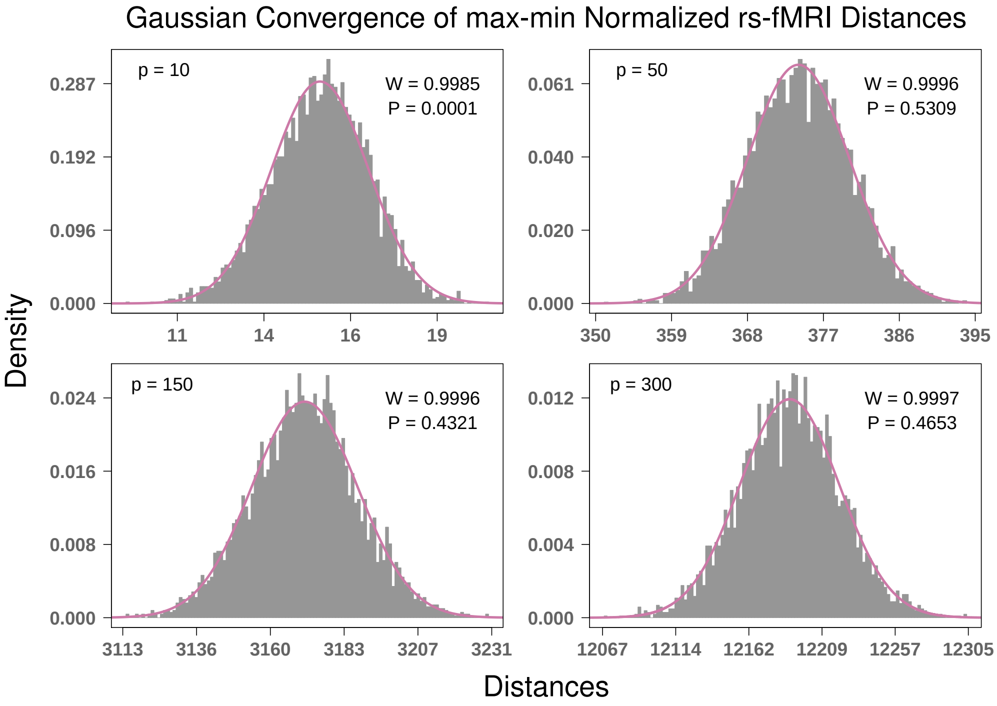

S8 Fig. Convergence to Gaussian for max-min normalized rs-fMRI distances for simulated correlation matrices with $m = 100$ instances and $p = 10, 50, 150$, and 300 attributes (or ROIs). Correlation matrices were generated for each instance from random normal $m \times p$ data matrices. Each correlation matrix was then stretched out into a long vector, Fisher r-to-z transformed, stored in a $p(p-1) \times m$ matrix, and standardized so that the m columns are mean 0 and unit variance. Convergence to Gaussian occurs approximately as rapidly as the standard rs-fMRI metric. Just as in the standard rs-fMRI metric, the large attribute dimension $p(p-1)$ means that there are significantly more terms in each sum to compute pairwise distances. Therefore, Classical Central Limit Theorem dictates that distances in this context will be closer to Gaussian. P values from Shapiro-Wilk test, where the null hypothesis is a Gaussian distribution.

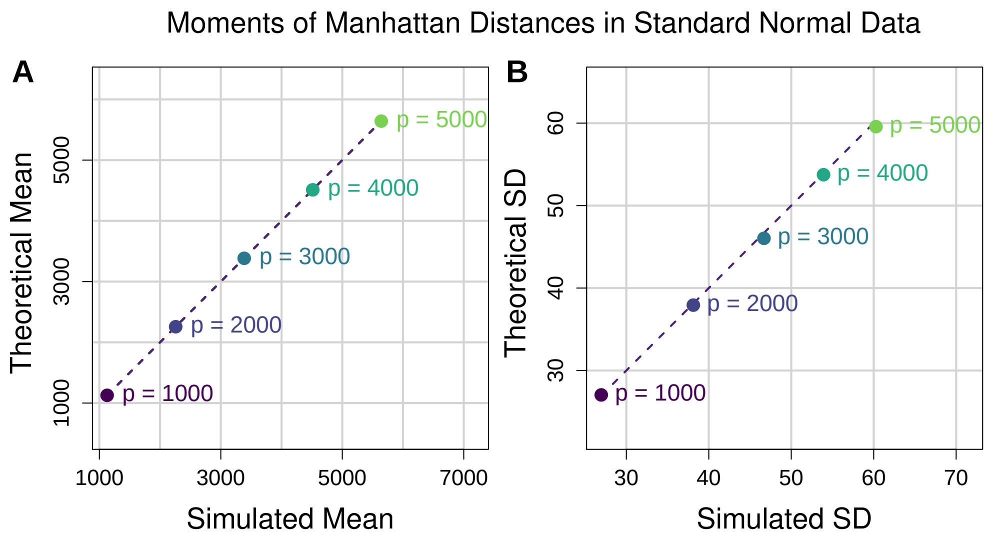

S9 Fig. Comparison of theoretical and simulated moments of Manhattan distances in standard normal data. (**A**) Scatter plot of theoretical vs simulated mean Manhattan distance. Each point represents a different number of attributes p. For each value of p we fixed $m = 100$ and generated 20 distance matrices from standard normal data and computed the average simulated pairwise distance from the 20 iterations. The corresponding theoretical mean was then computed for each value of p for comparison. The dashed line represents the identity (or $y = x$) line for reference. (**B**) Scatter plot of theoretical vs simulated standard deviation of Manhattan distance. These standard deviations come from the same random distance matrices for which mean distance was computed for **A**. Both theoretical mean and standard deviation approximate the simulated moments quite well.

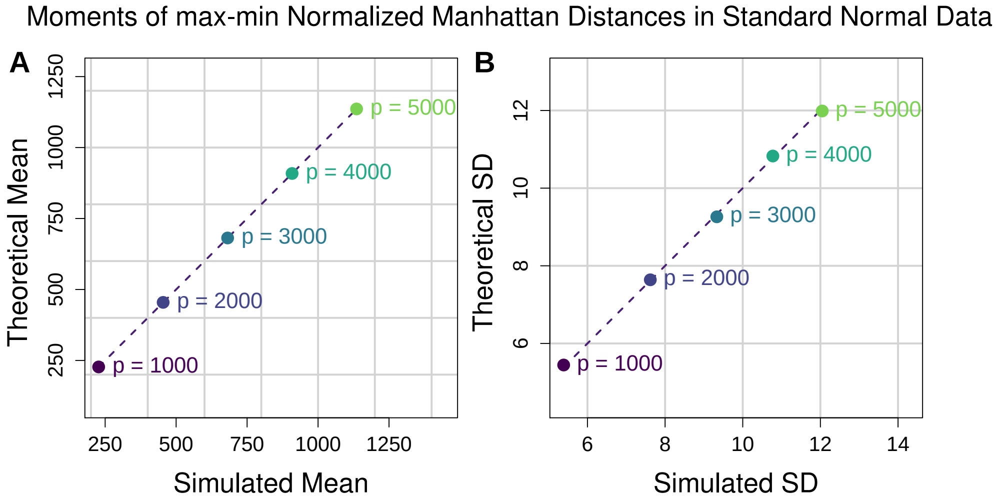

S10 Fig. Comparison of theoretical and simulated moments of max-min normalized Manhattan distances in standard normal data. (**A**) Scatter plot of theoretical vs simulated mean max-min normalized Manhattan distance. Each point represents a different number of attributes p. For each value of p we fixed $m = 100$ and generated 20 distance matrices from standard normal data and computed the average simulated pairwise distance from the 20 iterations. The corresponding theoretical mean was then computed for each value of p for comparison. The dashed line represents the identity (or $y = x$) line for reference. (**B**) Scatter plot of theoretical vs simulated standard deviation of max-min normalized Manhattan distance. These standard deviations come from the same random distance matrices for which mean distance was computed for **A**. Both theoretical mean and standard deviation approximate the simulated moments quite well.

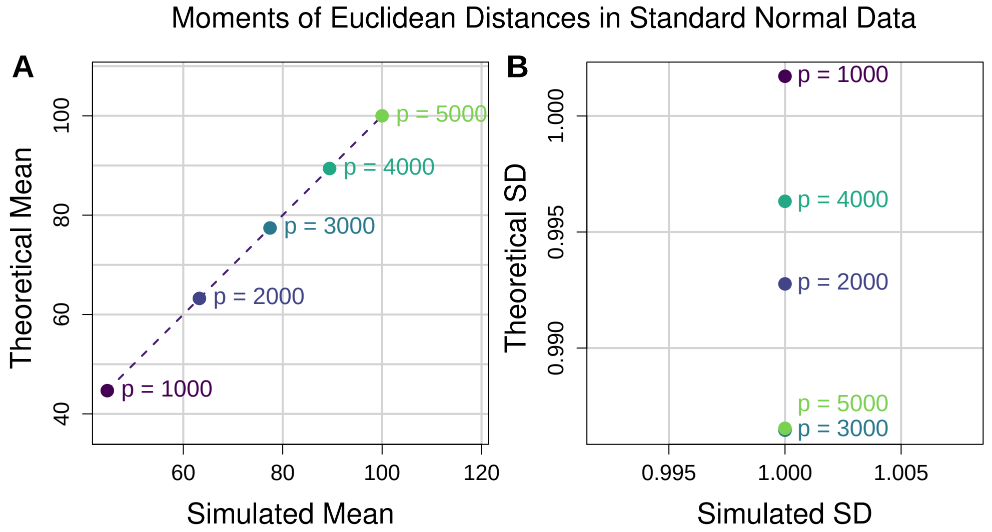

S11 Fig. Comparison of theoretical and simulated moments of Euclidean distances in standard normal data. (**A**) Scatter plot of theoretical vs simulated mean Euclidean distance. Each point represents a different number of attributes p. For each value of p we fixed $m = 100$ and generated 20 distance matrices from standard normal data and computed the average simulated pairwise distance from the 20 iterations. The corresponding theoretical mean was then computed for each value of p for comparison. The dashed line represents the identity (or $y = x$) line for reference. (**B**) Scatter plot of theoretical vs simulated standard deviation of Euclidean distance. These standard deviations come from the same random distance matrices for which mean distance was computed for **A**. Theoretical and simulated means lie approximately on the identity line because the mean is proportional to attribute dimension p. Theoretical standard deviation is constant, which is why each horizontal coordinate is the same for $p = 1000, 2000, 3000, 4000$, and 5000. The variation in sample standard deviation of Euclidean distance is quite small, so each simulated moment is clustered about 1.

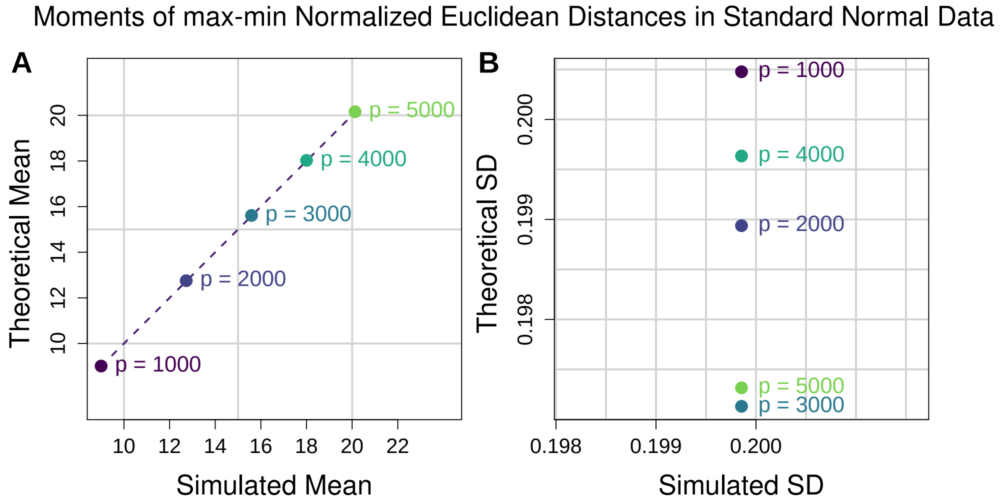

S12 Fig. Comparison of theoretical and simulated moments of max-min normalized Euclidean distances in standard normal data. (**A**) Scatter plot of theoretical vs simulated mean max-min normalized Euclidean distance. Each point represents a different number of attributes p. For each value of p we fixed $m = 100$ and generated 20 distance matrices from standard normal data and computed the average simulated pairwise distance from the 20 iterations. The corresponding theoretical mean was then computed for each value of p for comparison. The dashed line represents the identity (or $y = x$) line for reference. (**B**) Scatter plot of theoretical vs simulated standard deviation of max-min normalized Euclidean distance. These standard deviations come from the same random distance matrices for which mean distance was computed for **A**. Theoretical and simulated means lie approximately on the identity line because the mean is proportional to $\sqrt{p}$. Theoretical standard deviation is a function of the fixed attribute dimension m, which is why each horizontal coordinate is the same for $p = 1000, 2000, 3000, 4000$, and 5000. The variation in sample standard deviation of max-min normalized Euclidean distance is quite small, so each simulated moment is clustered about the theoretical value that depends on m.

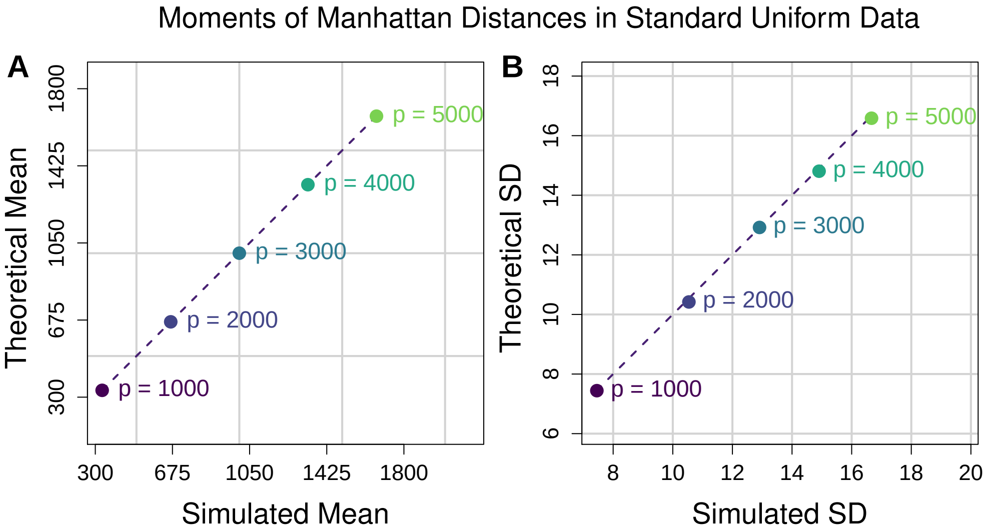

S13 Fig. Comparison of theoretical and simulated moments of Manhattan distances in standard uniform data. (**A**) Scatter plot of theoretical vs simulated mean Manhattan distance. Each point represents a different number of attributes p. For each value of p we fixed $m = 100$ and generated 20 distance matrices from standard uniform data and computed the average simulated pairwise distance from the 20 iterations. The corresponding theoretical mean was then computed for each value of p for comparison. The dashed line represents the identity (or $y = x$) line for reference. (**B**) Scatter plot of theoretical vs simulated standard deviation of Manhattan distance. These standard deviations come from the same random distance matrices for which mean distance was computed for **A**. Both theoretical mean and standard deviation approximate the simulated moments quite well.

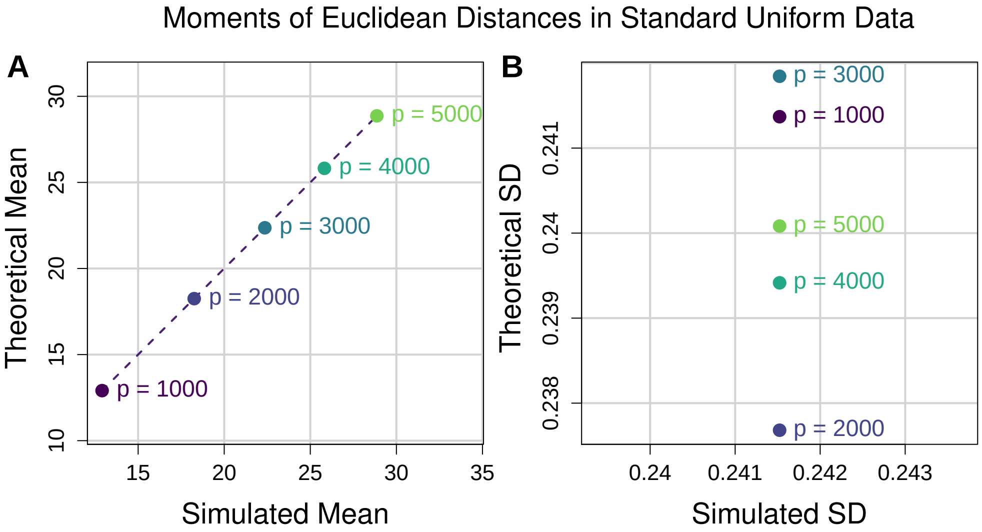

S14 Fig. Comparison of theoretical and simulated moments of Euclidean distances in standard uniform data. (**A**) Scatter plot of theoretical vs simulated mean Euclidean distance. Each point represents a different number of attributes p. For each value of p we fixed $m = 100$ and generated 20 distance matrices from standard uniform data and computed the average simulated pairwise distance from the 20 iterations. The corresponding theoretical mean was then computed for each value of p for comparison. The dashed line represents the identity (or $y = x$) line for reference. (**B**) Scatter plot of theoretical vs simulated standard deviation of Euclidean distance. These standard deviations come from the same random distance matrices for which mean distance was computed for **A**. Theoretical and simulated means lie approximately on the identity line because the mean is proportional to attribute dimension p. Theoretical standard deviation is constant, which is why each horizontal coordinate is the same for $p = 1000, 2000, 3000, 4000,$ and 5000. The variation in sample standard deviation of Euclidean distance is quite small, so each simulated moment is clustered about $7/120$.

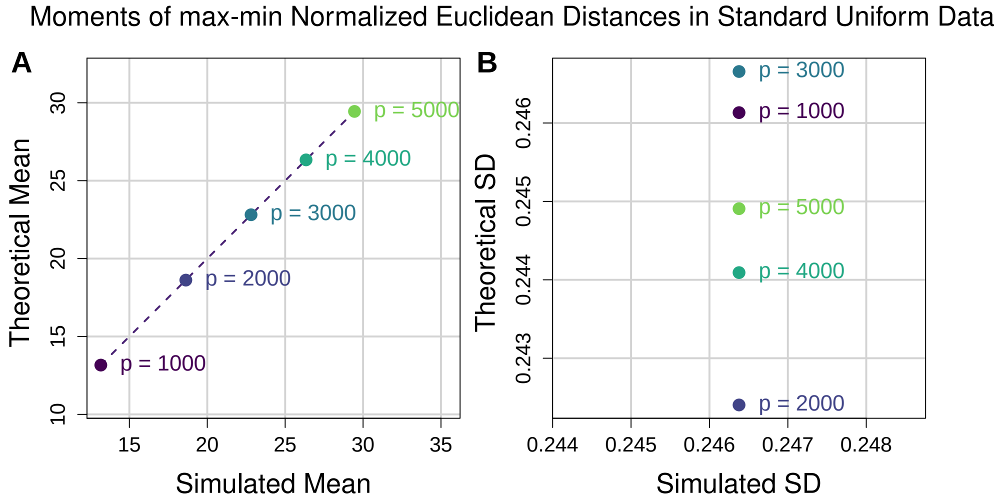

S15 Fig. Comparison of theoretical and simulated moments of max-min normalized Euclidean distances in standard uniform data. (**A**) Scatter plot of theoretical vs simulated mean max-min normalized Euclidean distance. Each point represents a different number of attributes p. For each value of p we fixed $m = 100$ and generated 20 distance matrices from standard uniform data and computed the average simulated pairwise distance from the 20 iterations. The corresponding theoretical mean was then computed for each value of p for comparison. The dashed line represents the identity (or $y = x$) line for reference. (**B**) Scatter plot of theoretical vs simulated standard deviation of max-min normalized Euclidean distance. These standard deviations come from the same random distance matrices for which mean distance was computed for **A**. Theoretical and simulated means lie approximately on the identity line because the mean is proportional to $\sqrt{p}$. Theoretical standard deviation is a function of the fixed attribute dimension m, which is why each horizontal coordinate is the same for $p = 1000, 2000, 3000, 4000,$ and 5000. The variation in sample standard deviation of max-min normalized Euclidean distance is quite small, so each simulated moment is clustered about the theoretical value that depends on m.

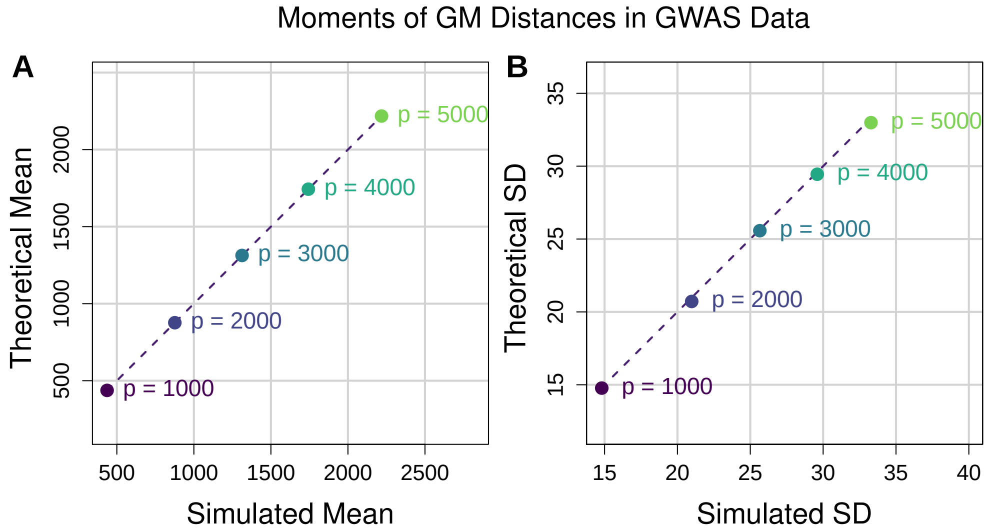

S16 Fig. Comparison of theoretical and simulated moments of GM distances in binomial GWAS data. (**A**) Scatter plot of theoretical vs simulated mean GM distance. Each point represents a different number of attributes p. For each value of p we fixed $m = 100$ and generated 20 distance matrices from binomial GWAS data and computed the average simulated pairwise distance from the 20 iterations. The corresponding theoretical mean was then computed for each value of p for comparison. The dashed line represents the identity (or $y = x$) line for reference. (**B**) Scatter plot of theoretical vs simulated standard deviation of GM distance. These standard deviations come from the same random distance matrices for which mean distance was computed for **A**. Both theoretical mean and standard deviation approximate the simulated moments quite well.

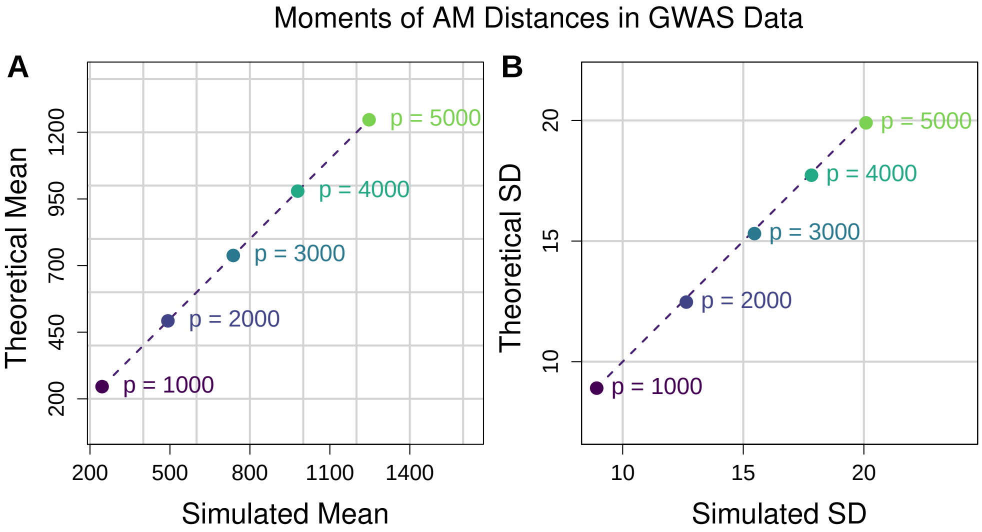

S17 Fig. Comparison of theoretical and simulated moments of AM distances in binomial GWAS data. (**A**) Scatter plot of theoretical vs simulated mean AM distance. Each point represents a different number of attributes p. For each value of p we fixed $m = 100$ and generated 20 distance matrices from binomial GWAS data and computed the average simulated pairwise distance from the 20 iterations. The corresponding theoretical mean was then computed for each value of p for comparison. The dashed line represents the identity (or $y = x$) line for reference. (**B**) Scatter plot of theoretical vs simulated standard deviation of AM distance. These standard deviations come from the same random distance matrices for which mean distance was computed for **A**. Both theoretical mean and standard deviation approximate the simulated moments quite well.

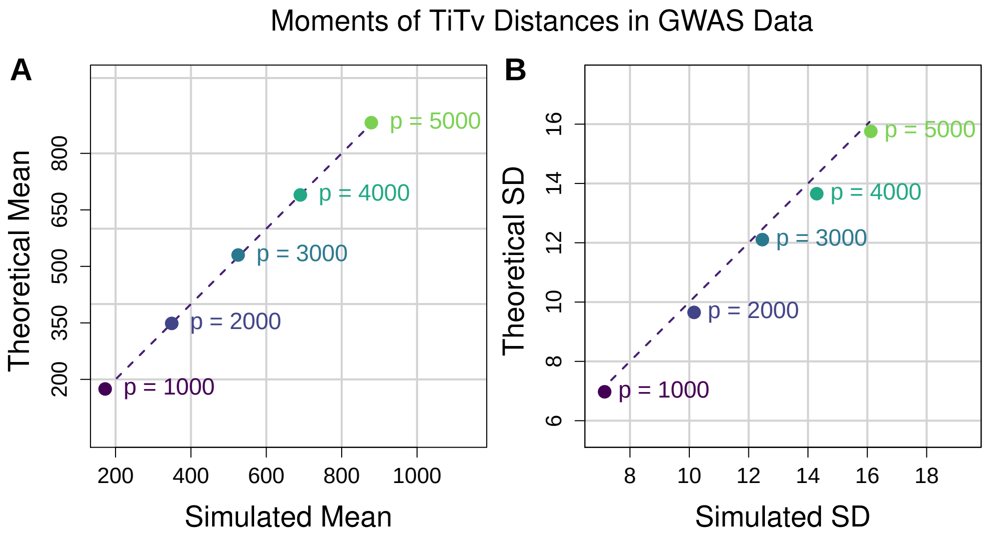

S18 Fig. Comparison of theoretical and simulated moments of TiTv distances in binomial GWAS data. For each simulated data set, the average MAF was set to be 0.205 and the Ti/Tv ratio (η) was fixed to be 2. (**A**) Scatter plot of theoretical vs simulated mean TiTv distance. Each point represents a different number of attributes p. For each value of p we fixed $m = 100$ and generated 20 distance matrices from binomial GWAS data and computed the average simulated pairwise distance from the 20 iterations. The corresponding theoretical mean was then computed for each value of p for comparison. The dashed line represents the identity (or $y = x$) line for reference. (**B**) Scatter plot of theoretical vs simulated standard deviation of TiTv distance. These standard deviations come from the same random distance matrices for which mean distance was computed for **A**. Both theoretical mean and standard deviation approximate the simulated moments quite well.

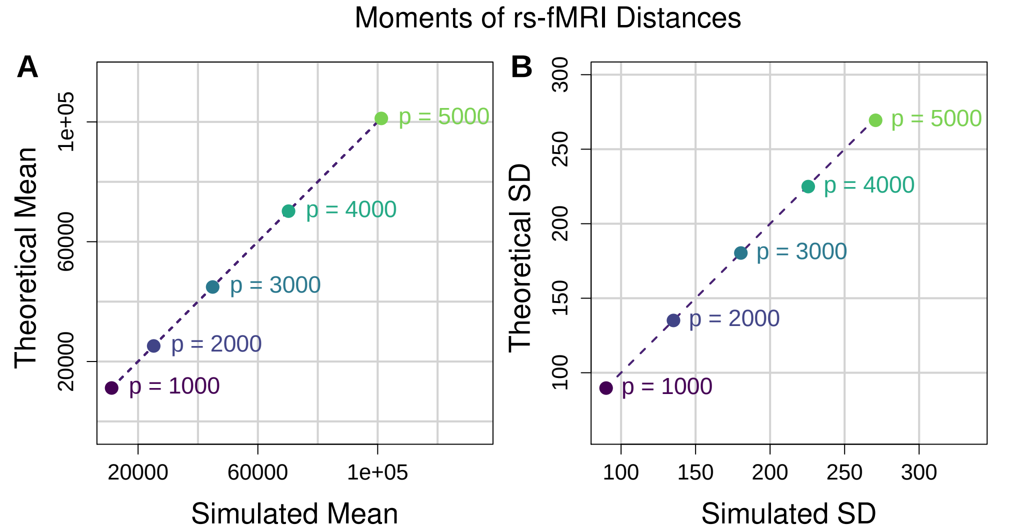

S19 Fig. Comparison of theoretical and simulated moments of rs-fMRI distances from random correlation matrices. For each instance, we generated a $p \times p$ correlation matrix from a random $m \times p$ standard normal data set. We then stretched out each correlation matrix into a long vector, Fisher r-to-z transformed the correlations, stored the vector in a column of a large $p(p-1) \times m$ matrix, and then standardized columns to be mean 0 and unit variance. (**A**) Scatter plot of theoretical vs simulated mean rs-fMRI distance. Each point represents a different number of attributes p. For each value of p we fixed $m = 100$ and generated 20 distance matrices from rs-fMRI data and computed the average simulated pairwise distance from the 20 iterations. The corresponding theoretical mean was then computed for each value of p for comparison. The dashed line represents the identity (or $y = x$) line for reference. (**B**) Scatter plot of theoretical vs simulated standard deviation of rs-fMRI distance. These standard deviations come from the same random distance matrices for which mean distance was computed for **A**. Both theoretical mean and standard deviation approximate the simulated moments quite well.

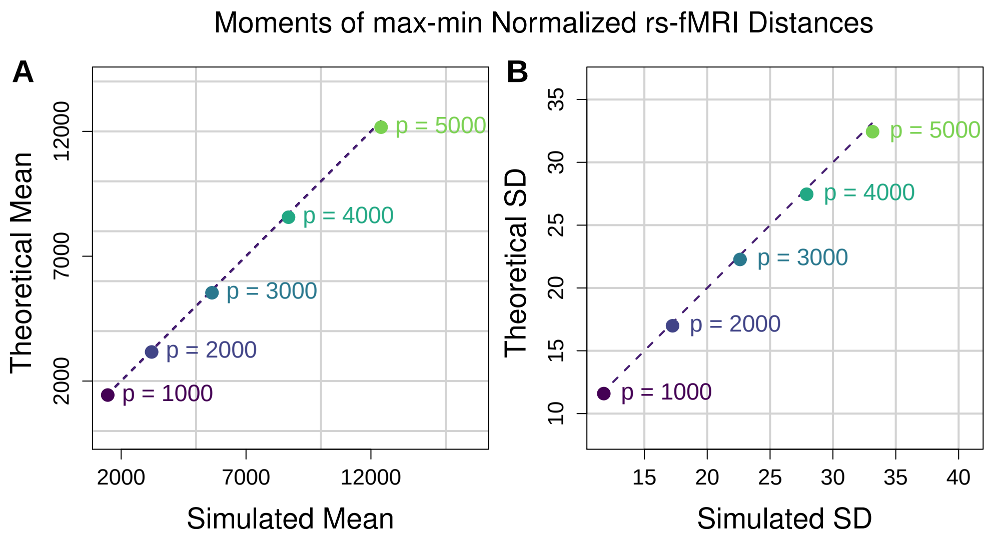

S20 Fig. Comparison of theoretical and simulated moments of max-min normalized rs-fMRI distances from random correlation matrices. For each instance, we generated a $p \times p$ correlation matrix from a random $m \times p$ standard normal data set. We then stretched out each correlation matrix into a long vector, Fisher r-to-z transformed the correlations, stored the vector in a column of a large $p(p-1) \times m$ matrix, and then standardized columns to be mean 0 and unit variance. (**A**) Scatter plot of theoretical vs simulated mean max-min normalized rs-fMRI distance. Each point represents a different number of attributes p. For each value of p we fixed $m = 100$ and generated 20 distance matrices from rs-fMRI data and computed the average simulated pairwise distance from the 20 iterations. The corresponding theoretical mean was then computed for each value of p for comparison. The dashed line represents the identity (or $y = x$) line for reference. (**B**) Scatter plot of theoretical vs simulated standard deviation of max-min normalized rs-fMRI distance. These standard deviations come from the same random distance matrices for which mean distance was computed for **A**. Both theoretical mean and standard deviation approximate the simulated moments quite well.

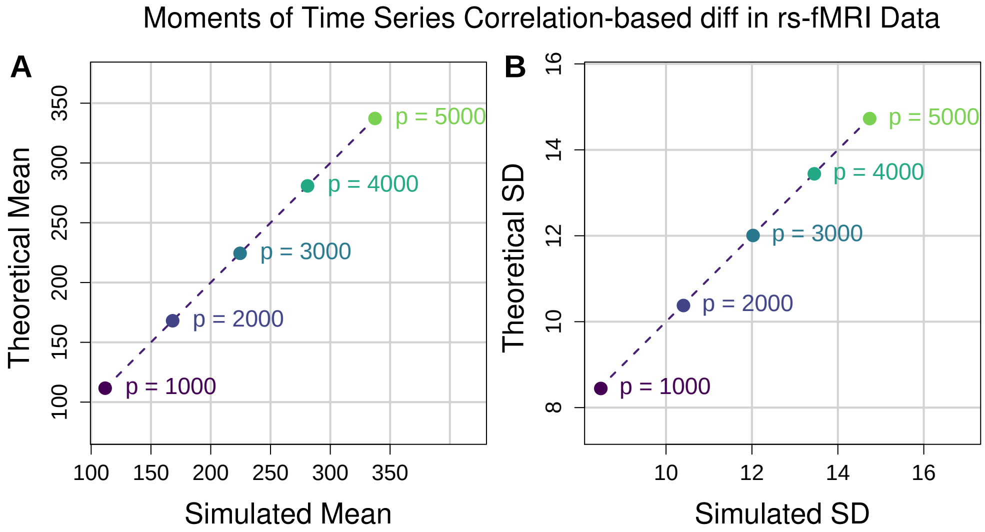

S21 Fig. Comparison of theoretical and simulated moments of rs-fMRI diff metric from random correlation matrices. For each instance, we generated a $p \times p$ correlation matrix from a random $m \times p$ standard normal data set. We then stretched out each correlation matrix into a long vector, Fisher r-to-z transformed the correlations, stored the vector in a column of a large $p(p-1) \times m$ matrix, and then standardized columns to be mean 0 and unit variance. (**A**) Scatter plot of theoretical vs simulated mean rs-fMRI diff. Each point represents a different number of attributes p. For each value of p we fixed $m = 100$ and generated 20 diff metric values from the rs-fMRI data and computed the average simulated diff from the 20 iterations. The corresponding theoretical mean was then computed for each value of p for comparison. The dashed line represents the identity (or $y = x$) line for reference. (**B**) Scatter plot of theoretical vs simulated standard deviation of rs-fMRI diff. These standard deviations come from the same random diff values from which mean diffs were computed for **A**. Both theoretical mean and standard deviation approximate the simulated moments quite well.

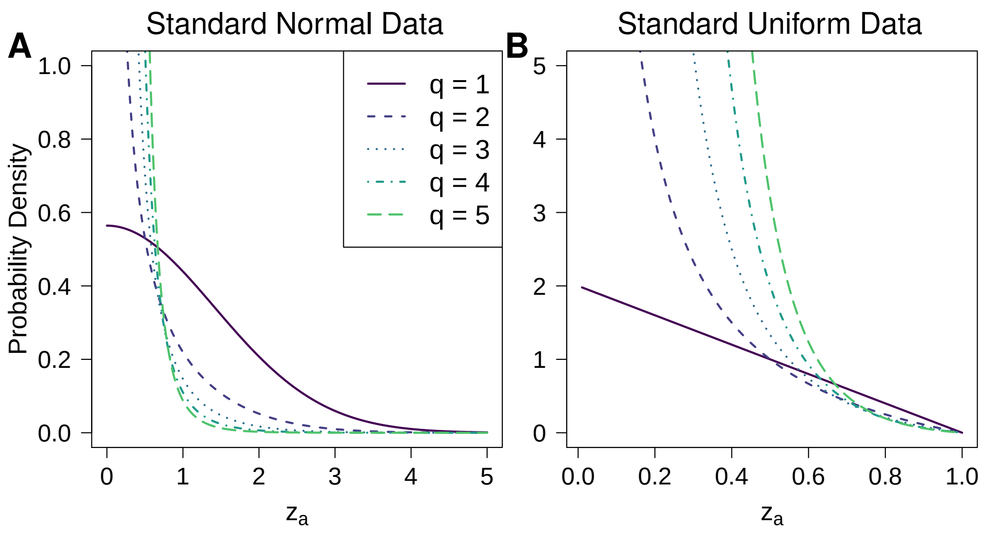

S22 Fig. Density curves for one-dimensional projected distances (diffs) onto a fixed attribute a for standard normal and standard uniform data. (**A**) Density curves for the distribution of attribute diff in standard normal data for $q = 1, 2, 3, 4$, and 5. This density is that of a Generalized Gamma distribution. For $q = 1$, this is also known as a half-normal distribution. (**B**) Density curves for the distribution of attribute diff in standard uniform data for $q = 1, 2, 3, 4$, and 5. This density is that of a Kumaraswamy distribution. For $q = 1$, this is also known as a triangular distribution.

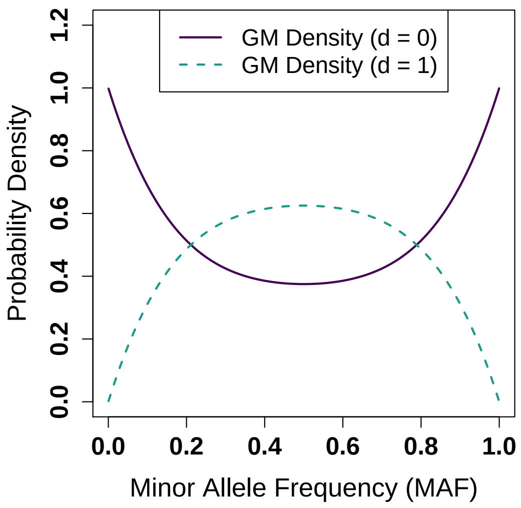

S23 Fig. One-dimensional projected GM distance (diff) onto an attribute vs minor allele frequency (MAF). For each possible value of the GM diff (d $= 0, 1$), the exact density of the GM diff is plotted for all possible values of MAF. The expected value of MAF at a particular locus a is f_a for all $a \in \mathcal{A}$, where f_a is the probability of a minor allele occurring at locus a. For each element X_{ia} of the data matrix for a fixed attribute a, we have $X_{ia} \sim \mathcal{B}(2, f_a)$. Depending on the MAF, the frequency of GM diff taking on a value of 0 or 1 changes. For small MAF, the GM diff will be 0 most often. As MAF increases beyond 0.5, the minor allele switches.

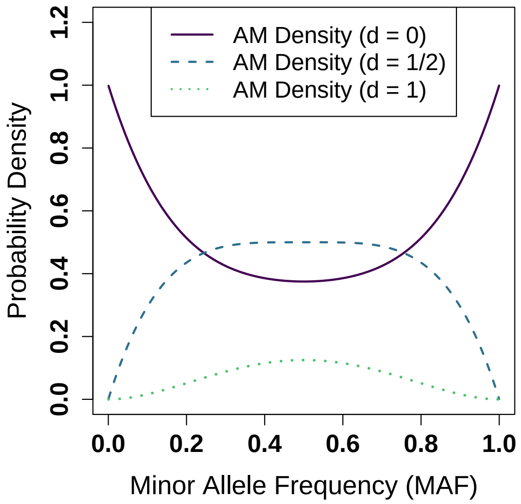

S24 Fig. One-dimensional projected AM distance (diff) onto an attribute vs minor allele frequency (MAF). For each possible value of the AM diff (d $= 0, 1/2, 1$), the exact density of the AM diff is plotted for all possible values of MAF. The expected value of MAF at a particular locus a is f_a for all $a \in \mathcal{A}$, where f_a is the probability of a minor allele occurring at locus a. For each element X_{ia} of the data matrix for a fixed attribute a, we have $X_{ia} \sim \mathcal{B}(2, f_a)$. Depending on the MAF, the frequency of AM diff taking on a value of 0, 1/2, or 1 changes. For small MAF, the AM diff will be 0 most often. For large MAF ($\approx$0.5), the AM diff will be mostly 1/2 with 1 being the second most common value. The least common value of the AM diff is 1. As MAF increases beyond 0.5, the minor allele switches.

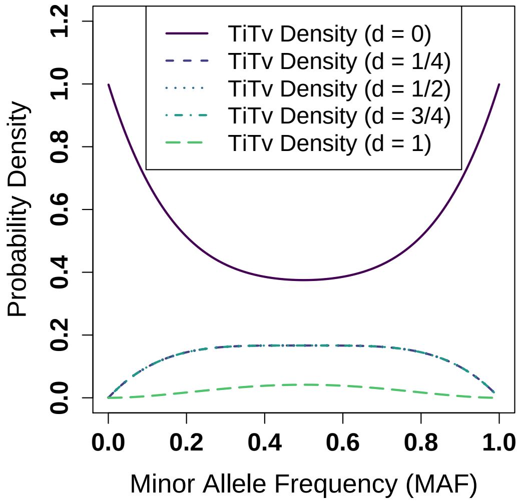

S25 Fig. One-dimensional projected TiTv distance (diff) onto an attribute vs minor allele frequency (MAF). For each possible value of the TiTv diff (d $= 0, 1/4, 1/2, 3/4, 1$), the exact density of the TiTv diff is plotted for all possible values of MAF. The Ti/Tv ratio η was fixed to be 2. The expected value of MAF at a particular locus a is f_a for all $a \in \mathcal{A}$, where f_a is the probability of a minor allele occurring at locus a. For each element X_{ia} of the data matrix for a fixed attribute a, we have $X_{ia} \sim \mathcal{B}(2, f_a)$. Depending on the MAF, the frequency of TiTv diff taking on a value of 0, 1/4, 1/2, 3/4, or 1 changes. The density of the TiTv diff for d $= 1/4, 1/2,$ and $3/4$ has the same resulting curve as a function of MAF. The most common value of TiTv diff at any MAF is 0, the second most common is 1/4, 1/2, or 3/4, and the least common is 1. As MAF increases beyond 0.5, the minor allele switches.

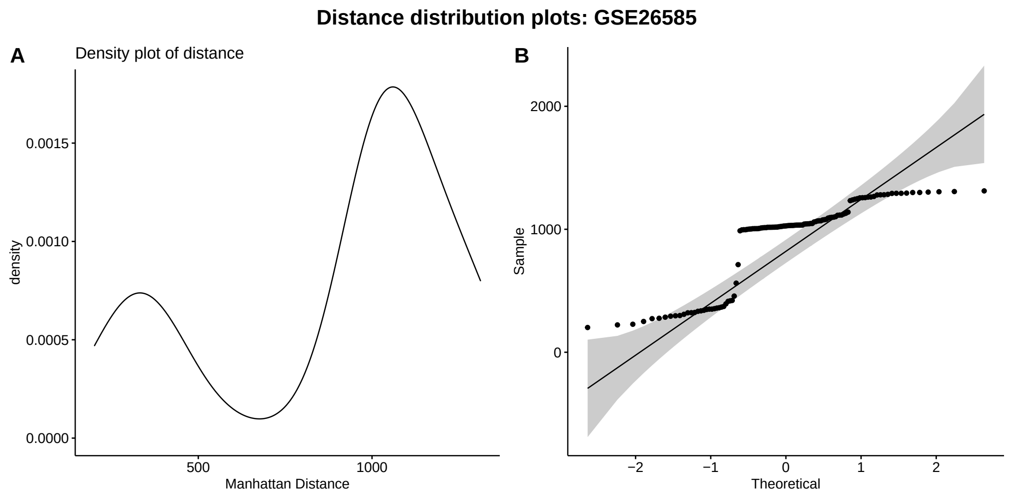

S26 Fig. Density and quantile-quantile plots for distances between samples in GSE26585. **A** Estimated density curve for distances. **B** Quantile-quantile plot between theoretical (standard normal) quantiles and sample distance quantiles.

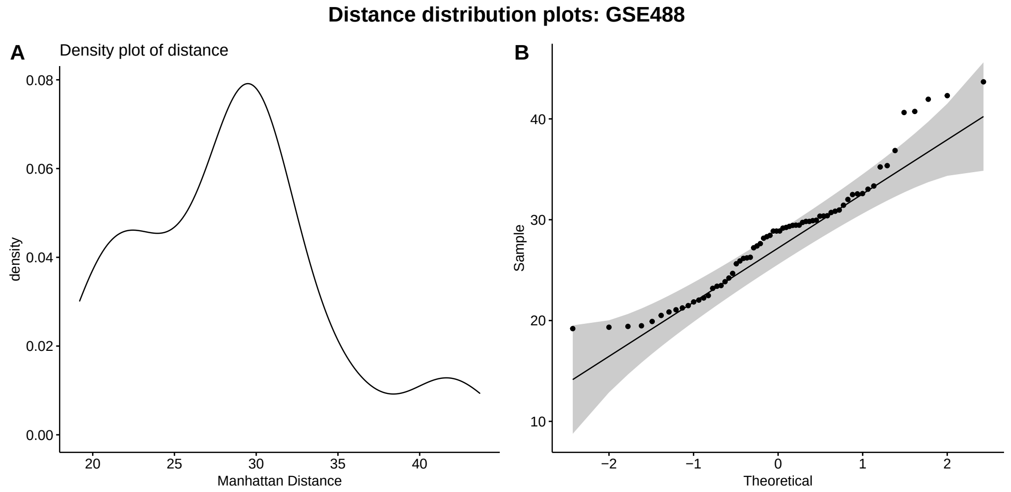

S27 Fig. Density and quantile-quantile plots for distances between samples in GSE488. **A** Estimated density curve for distances. **B** Quantile-quantile plot between theoretical (standard normal) quantiles and sample distance quantiles.

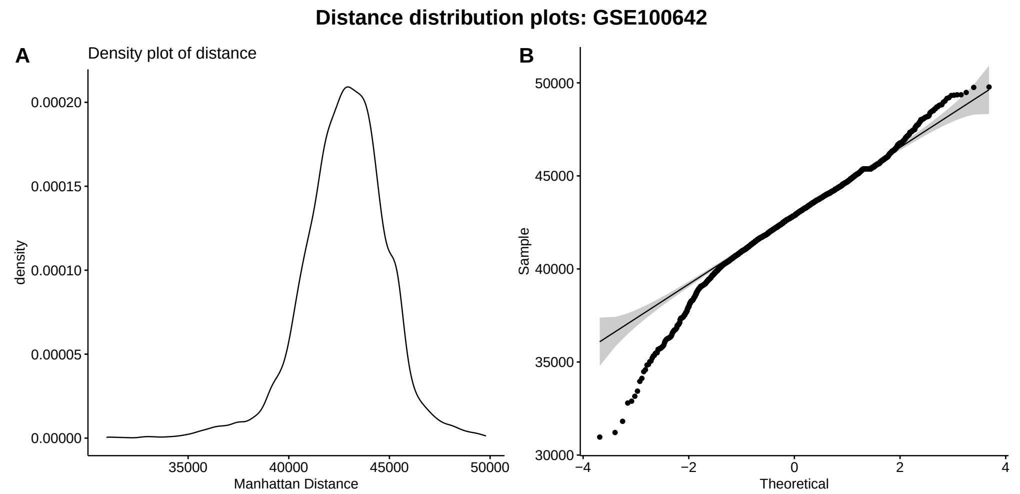

S28 Fig. Density and quantile-quantile plots for distances between samples in GSE100642. **A** Estimated density curve for distances. **B** Quantile-quantile plot between theoretical (standard normal) quantiles and sample distance quantiles.

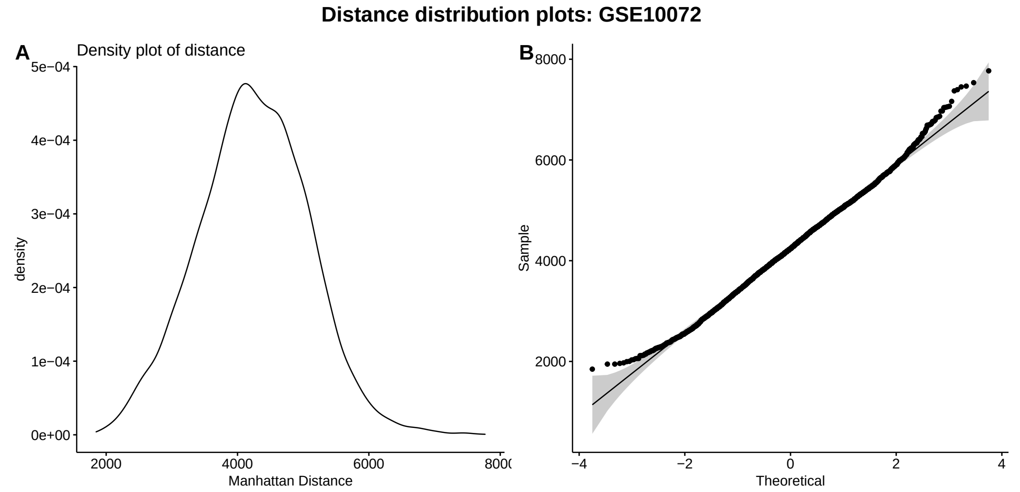

S29 Fig. Density and quantile-quantile plots for distances between samples in GSE10072. **A** Estimated density curve for distances. **B** Quantile-quantile plot between theoretical (standard normal) quantiles and sample distance quantiles.

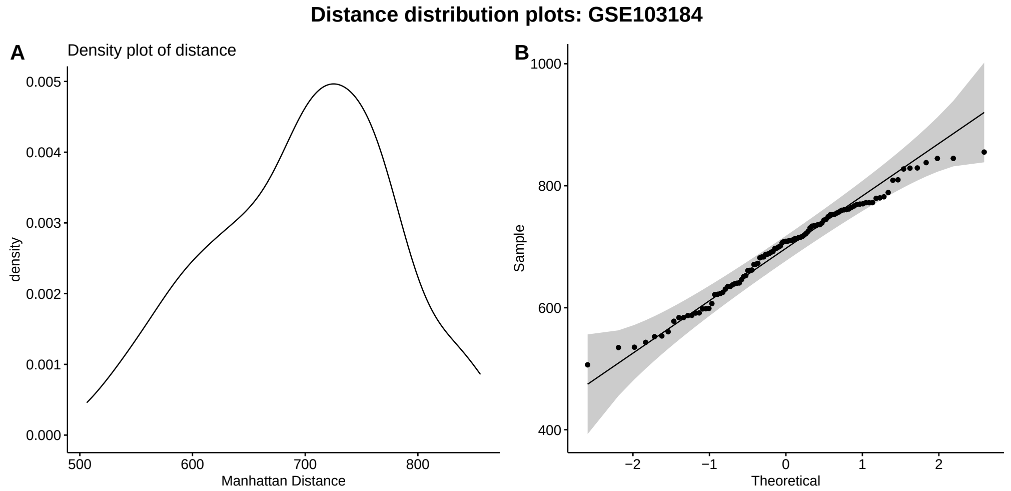

S30 Fig. Density and quantile-quantile plots for distances between samples in GSE103184. **A** Estimated density curve for distances. **B** Quantile-quantile plot between theoretical (standard normal) quantiles and sample distance quantiles.

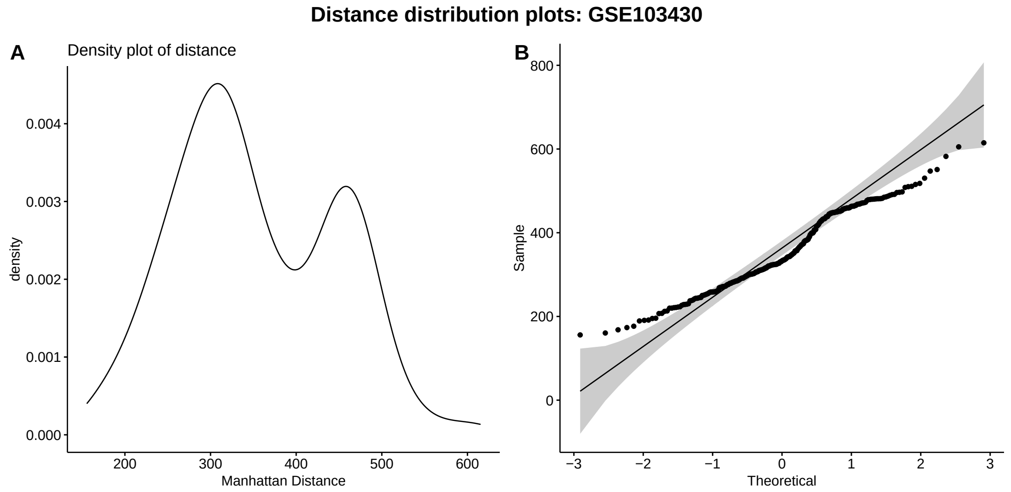

S31 Fig. Density and quantile-quantile plots for distances between samples in GSE103430. **A** Estimated density curve for distances. **B** Quantile-quantile plot between theoretical (standard normal) quantiles and sample distance quantiles.

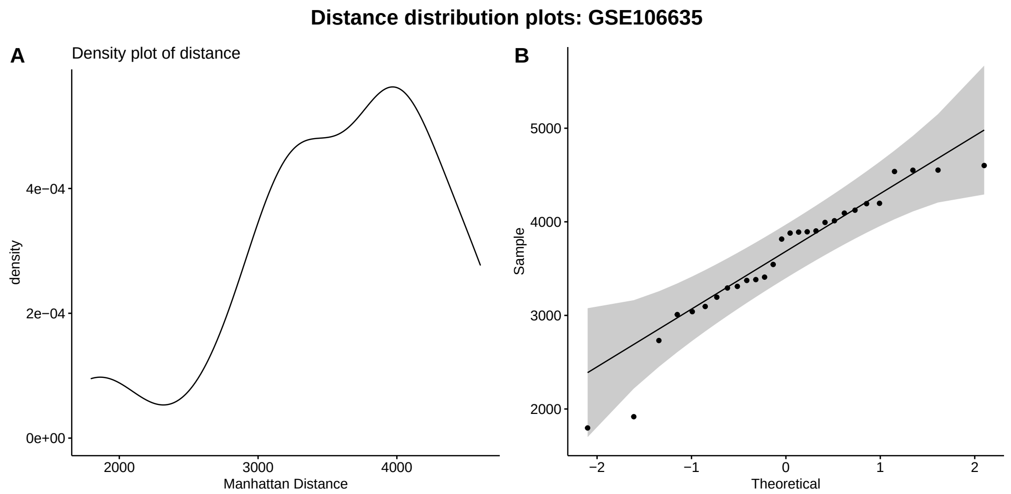

S32 Fig. Density and quantile-quantile plots for distances between samples in GSE106635. **A** Estimated density curve for distances. **B** Quantile-quantile plot between theoretical (standard normal) quantiles and sample distance quantiles.

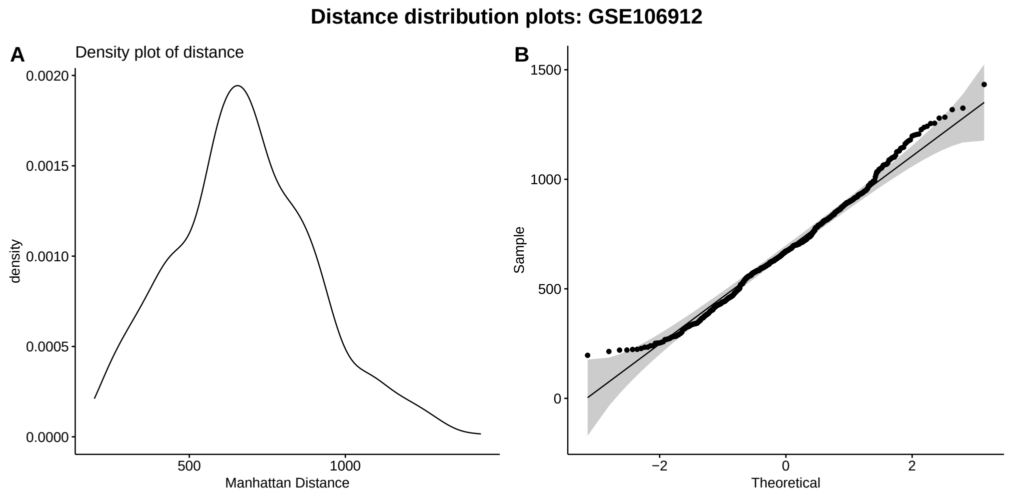

S33 Fig. Density and quantile-quantile plots for distances between samples in GSE106912. **A** Estimated density curve for distances. **B** Quantile-quantile plot between theoretical (standard normal) quantiles and sample distance quantiles.

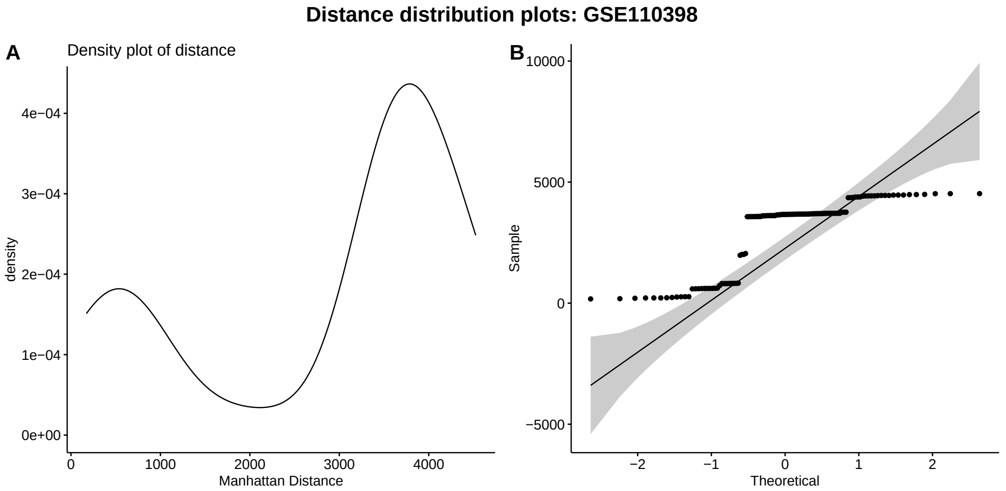

S34 Fig. Density and quantile-quantile plots for distances between samples in GSE110398. **A** Estimated density curve for distances. **B** Quantile-quantile plot between theoretical (standard normal) quantiles and sample distance quantiles.

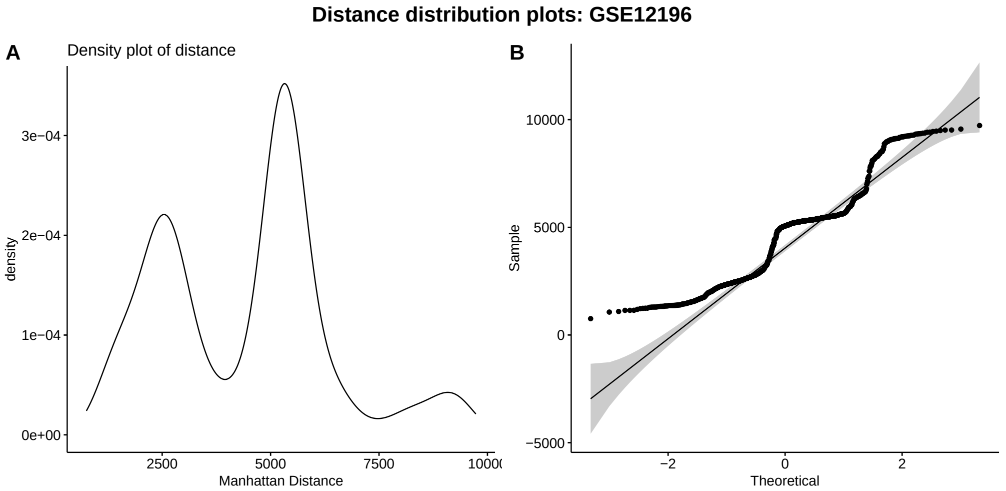

S35 Fig. Density and quantile-quantile plots for distances between samples in GSE12196. **A** Estimated density curve for distances. **B** Quantile-quantile plot between theoretical (standard normal) quantiles and sample distance quantiles.

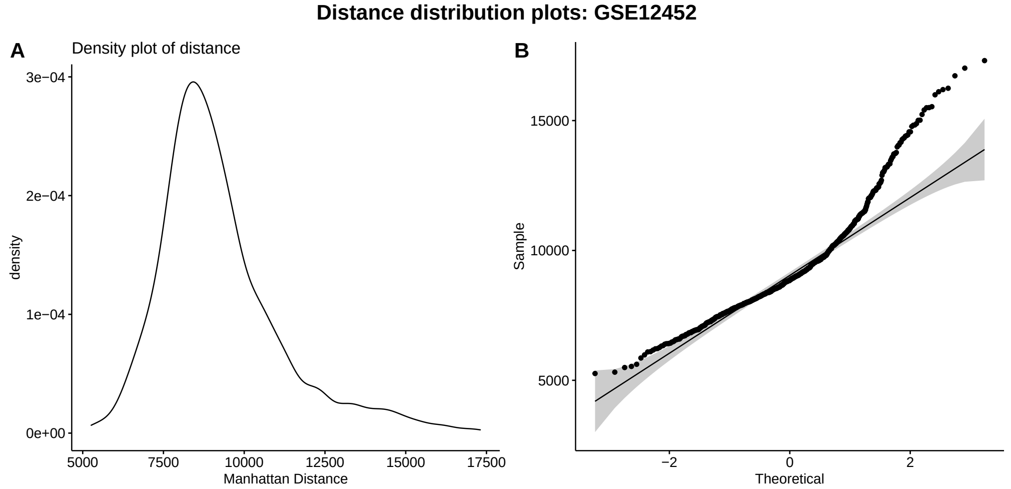

S36 Fig. Density and quantile-quantile plots for distances between samples in GSE12452. **A** Estimated density curve for distances. **B** Quantile-quantile plot between theoretical (standard normal) quantiles and sample distance quantiles.

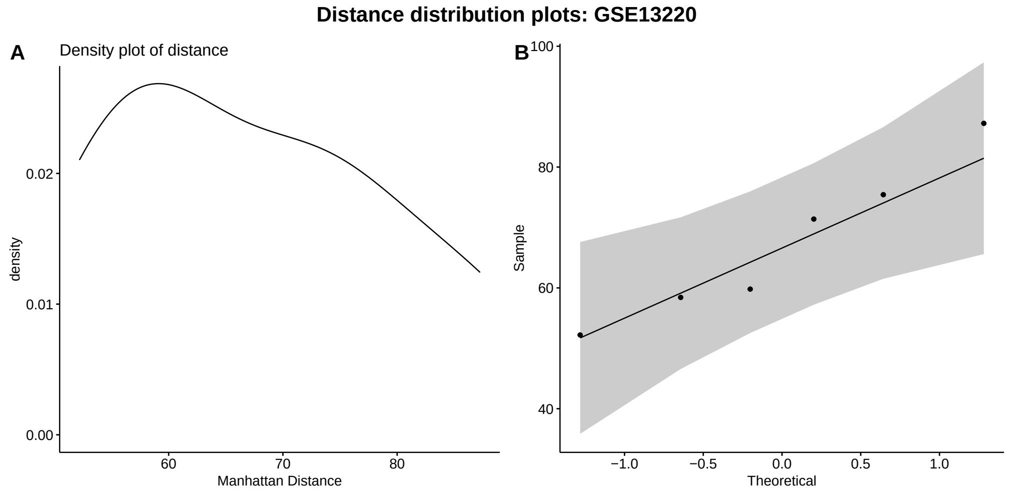

S37 Fig. Density and quantile-quantile plots for distances between samples in GSE13220. **A** Estimated density curve for distances. **B** Quantile-quantile plot between theoretical (standard normal) quantiles and sample distance quantiles.

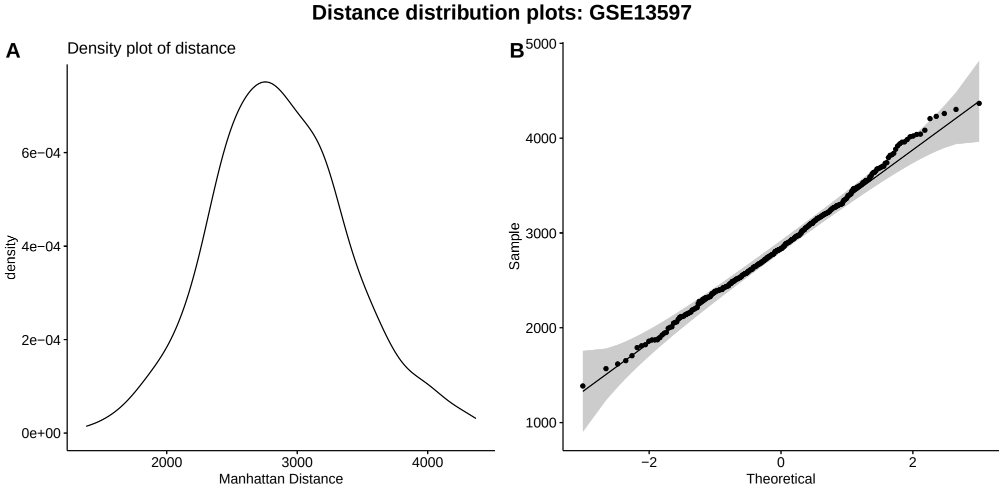

S38 Fig. Density and quantile-quantile plots for distances between samples in GSE13597. **A** Estimated density curve for distances. **B** Quantile-quantile plot between theoretical (standard normal) quantiles and sample distance quantiles.

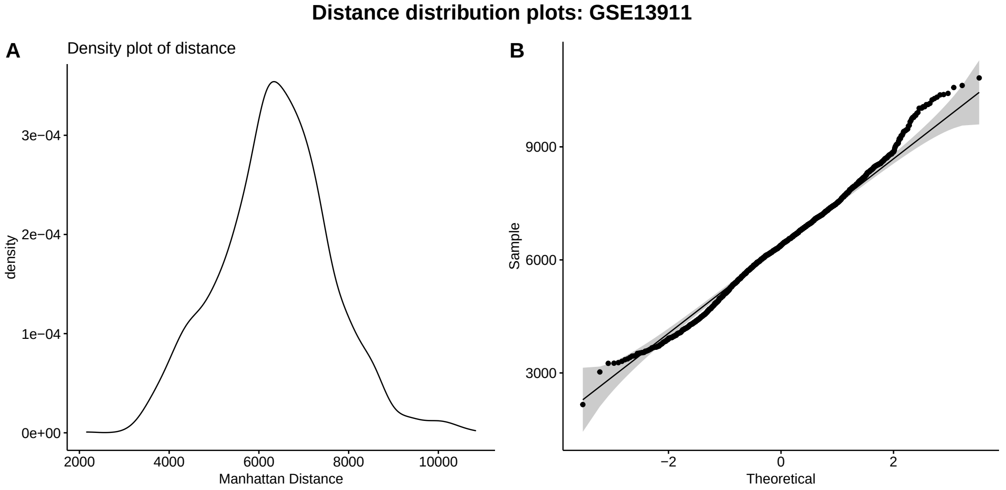

S39 Fig. Density and quantile-quantile plots for distances between samples in GSE13911. **A** Estimated density curve for distances. **B** Quantile-quantile plot between theoretical (standard normal) quantiles and sample distance quantiles.

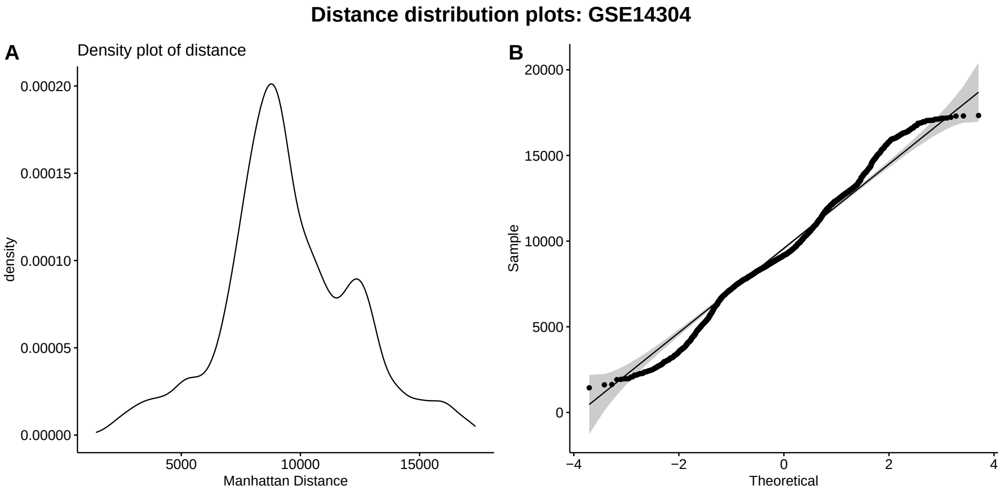

S40 Fig. Density and quantile-quantile plots for distances between samples in GSE14304. **A** Estimated density curve for distances. **B** Quantile-quantile plot between theoretical (standard normal) quantiles and sample distance quantiles.

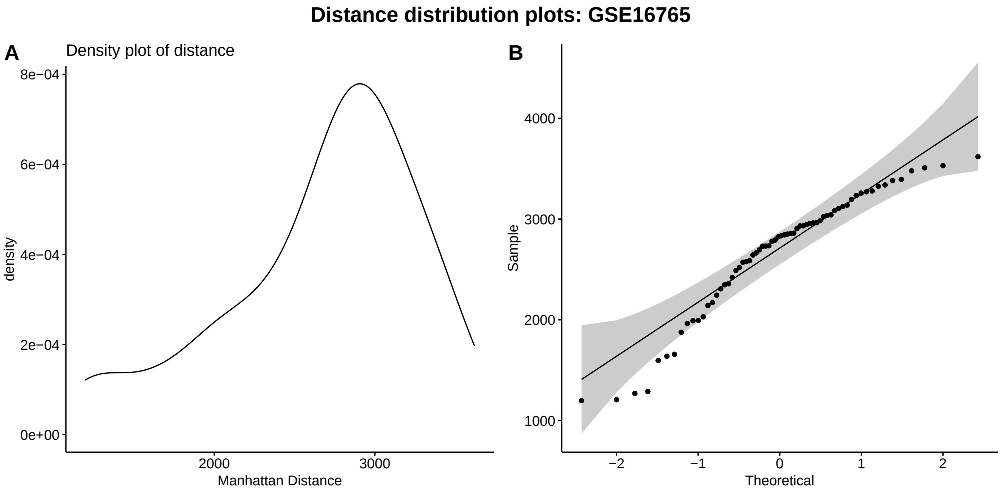

S41 Fig. Density and quantile-quantile plots for distances between samples in GSE16765. **A** Estimated density curve for distances. **B** Quantile-quantile plot between theoretical (standard normal) quantiles and sample distance quantiles.

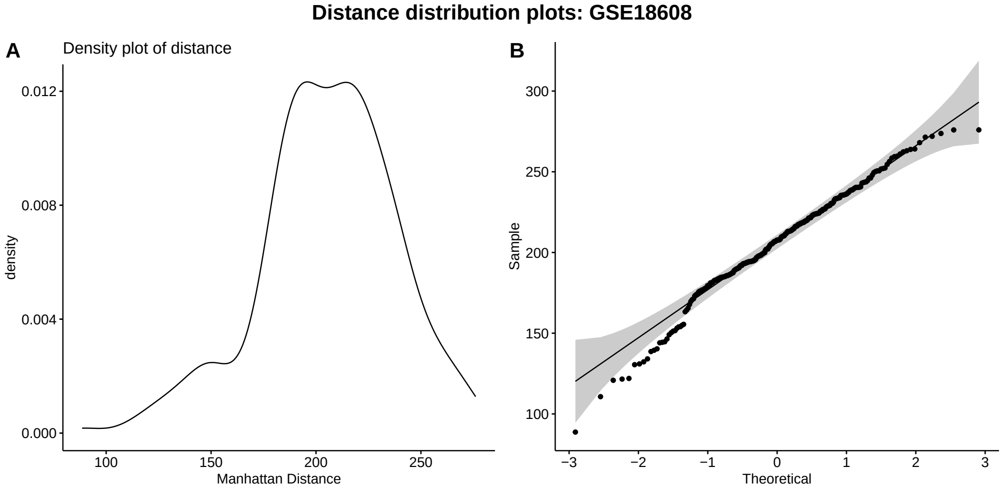

S42 Fig. Density and quantile-quantile plots for distances between samples in GSE18608. **A** Estimated density curve for distances. **B** Quantile-quantile plot between theoretical (standard normal) quantiles and sample distance quantiles.

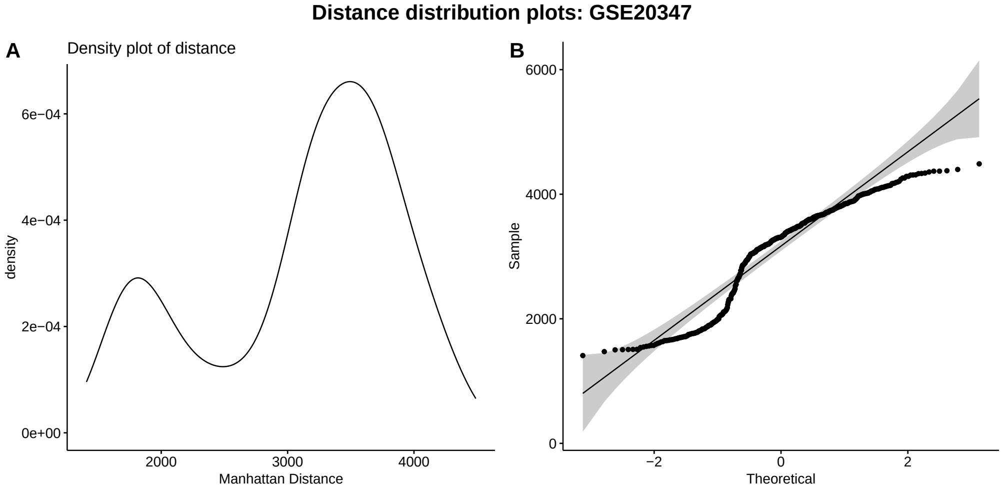

S43 Fig. Density and quantile-quantile plots for distances between samples in GSE20347. **A** Estimated density curve for distances. **B** Quantile-quantile plot between theoretical (standard normal) quantiles and sample distance quantiles.

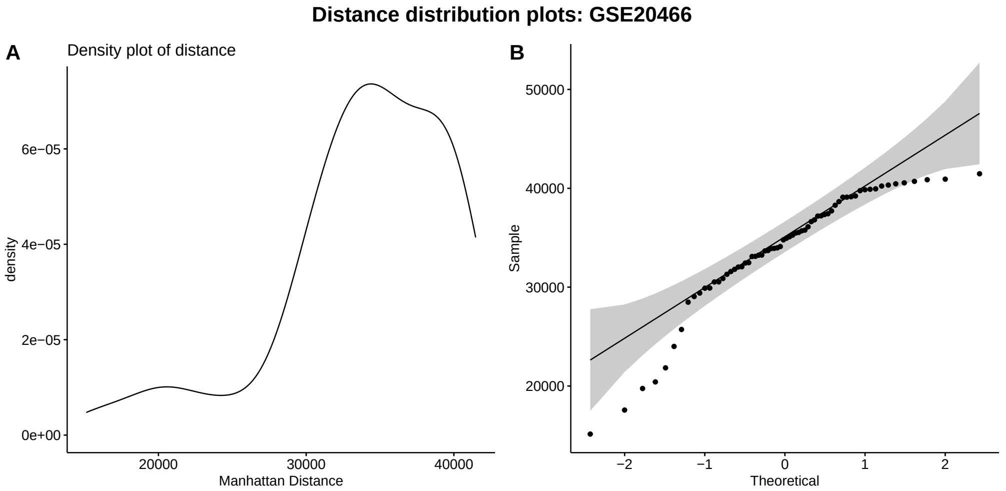

S44 Fig. Density and quantile-quantile plots for distances between samples in GSE20466. **A** Estimated density curve for distances. **B** Quantile-quantile plot between theoretical (standard normal) quantiles and sample distance quantiles.

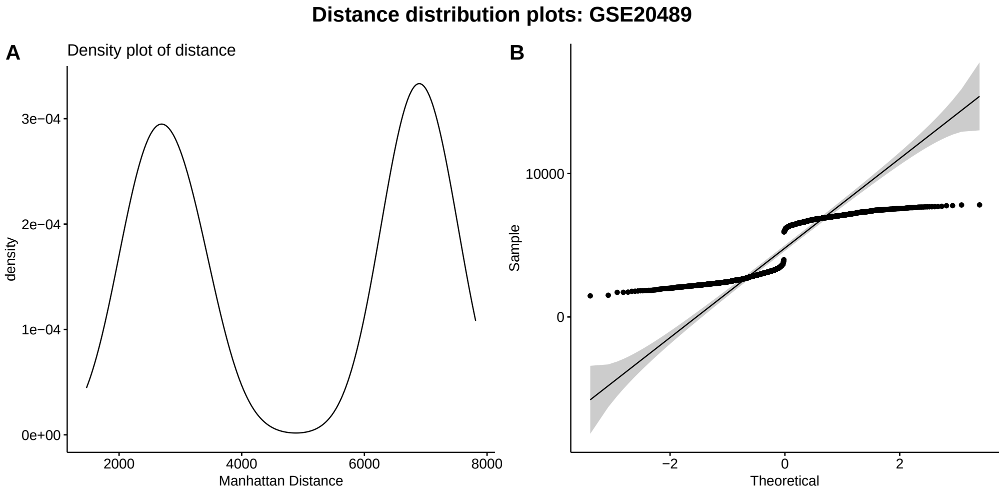

S45 Fig. Density and quantile-quantile plots for distances between samples in GSE20489. **A** Estimated density curve for distances. **B** Quantile-quantile plot between theoretical (standard normal) quantiles and sample distance quantiles.

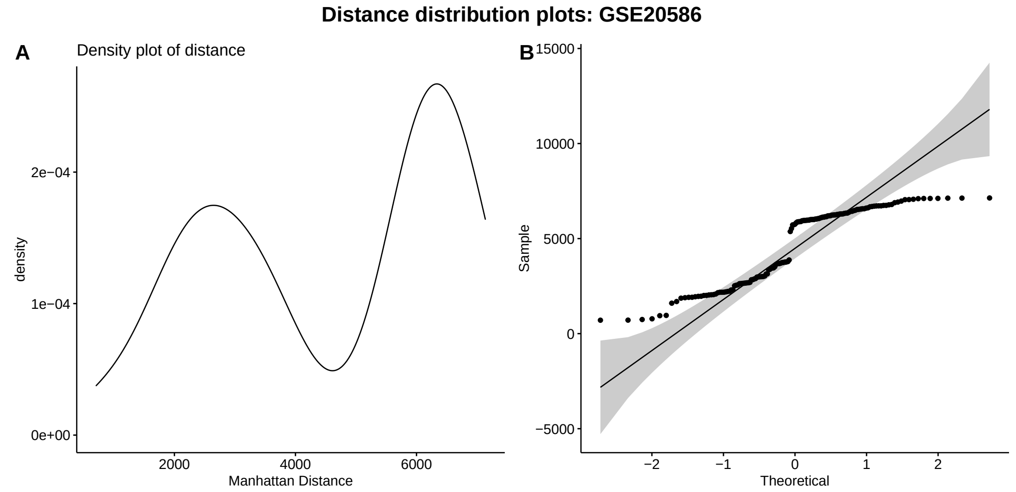

S46 Fig. Density and quantile-quantile plots for distances between samples in GSE20586. **A** Estimated density curve for distances. **B** Quantile-quantile plot between theoretical (standard normal) quantiles and sample distance quantiles.

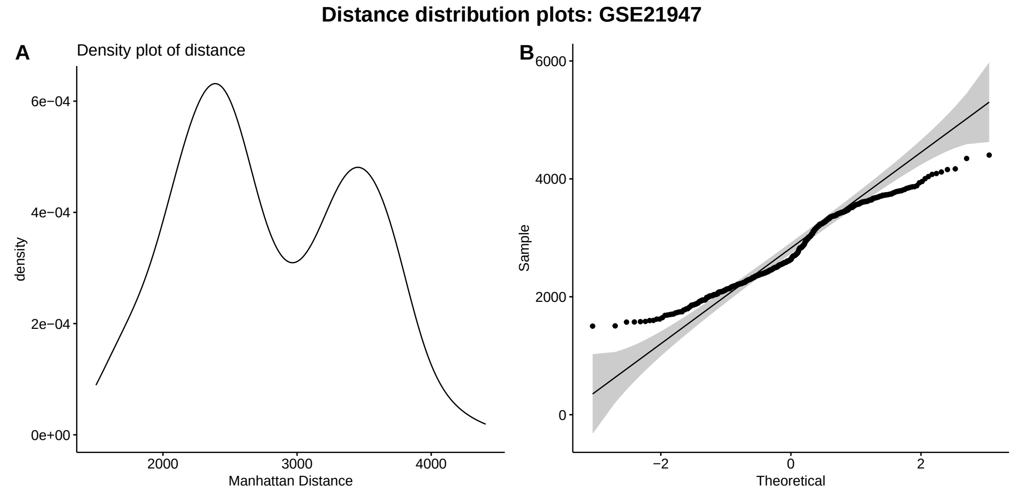

S47 Fig. Density and quantile-quantile plots for distances between samples in GSE21947. **A** Estimated density curve for distances. **B** Quantile-quantile plot between theoretical (standard normal) quantiles and sample distance quantiles.

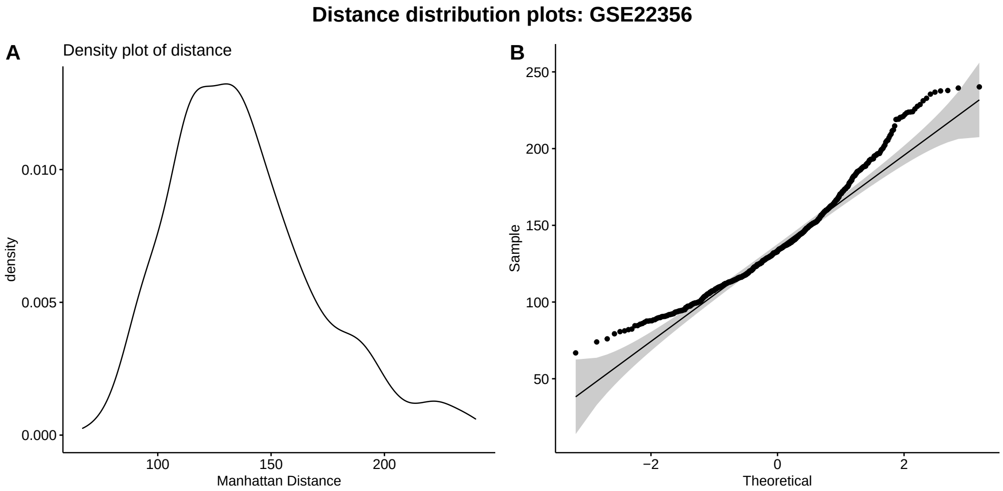

S48 Fig. Density and quantile-quantile plots for distances between samples in GSE22356. **A** Estimated density curve for distances. **B** Quantile-quantile plot between theoretical (standard normal) quantiles and sample distance quantiles.

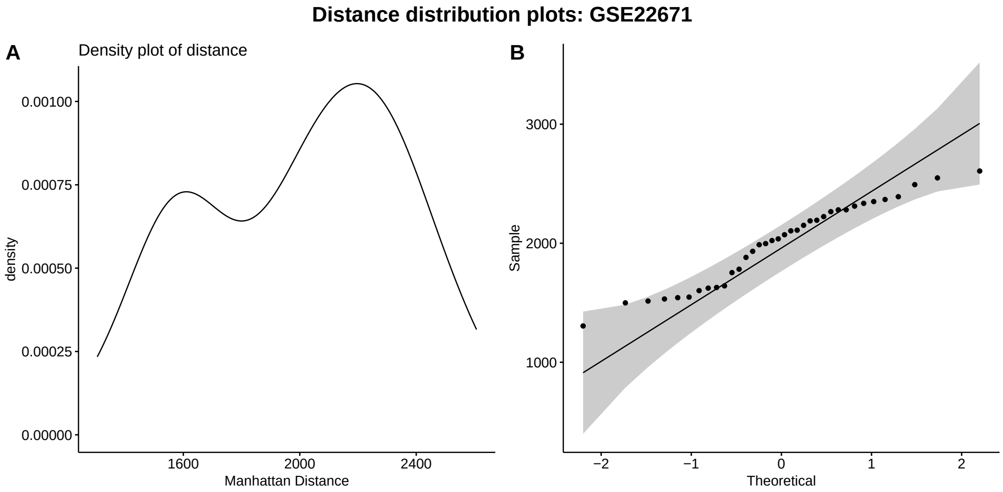

S49 Fig. Density and quantile-quantile plots for distances between samples in GSE22671. **A** Estimated density curve for distances. **B** Quantile-quantile plot between theoretical (standard normal) quantiles and sample distance quantiles.

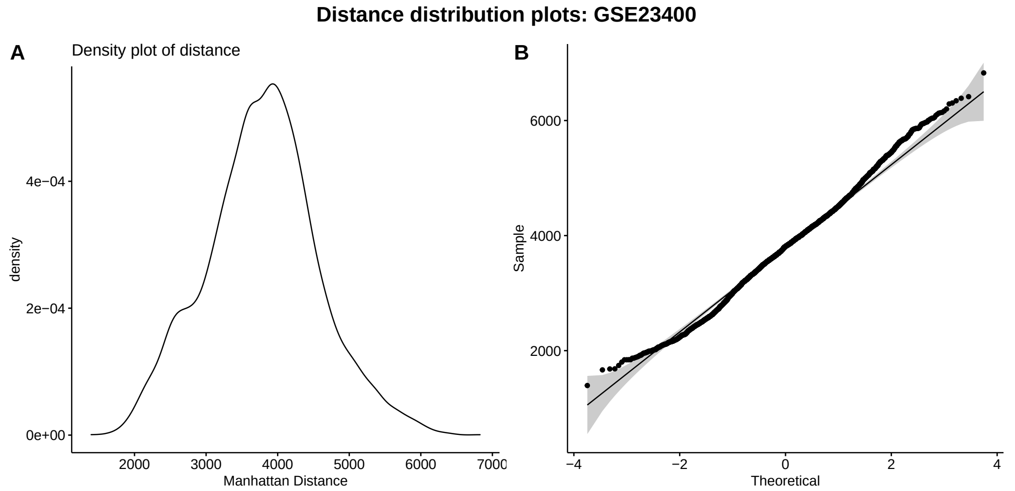

S50 Fig. Density and quantile-quantile plots for distances between samples in GSE23400. **A** Estimated density curve for distances. **B** Quantile-quantile plot between theoretical (standard normal) quantiles and sample distance quantiles.

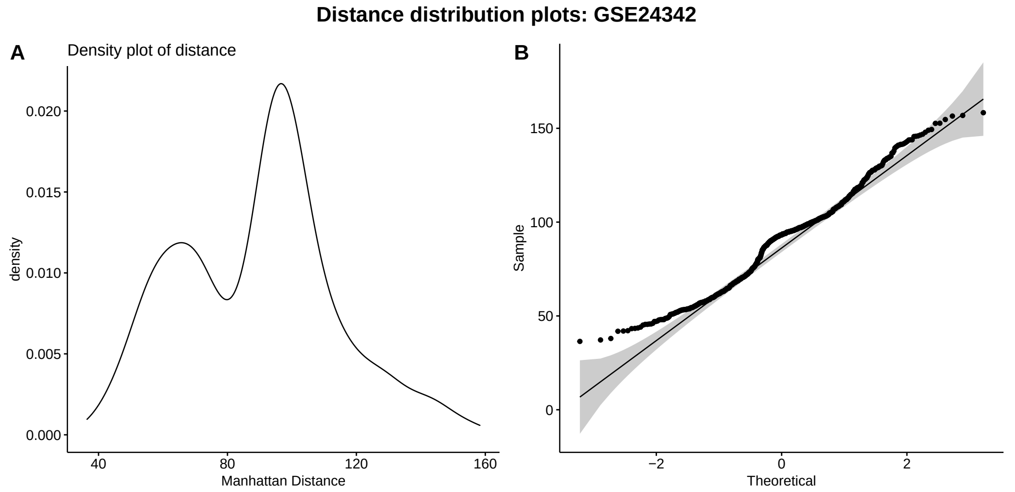

S51 Fig. Density and quantile-quantile plots for distances between samples in GSE24342. **A** Estimated density curve for distances. **B** Quantile-quantile plot between theoretical (standard normal) quantiles and sample distance quantiles.

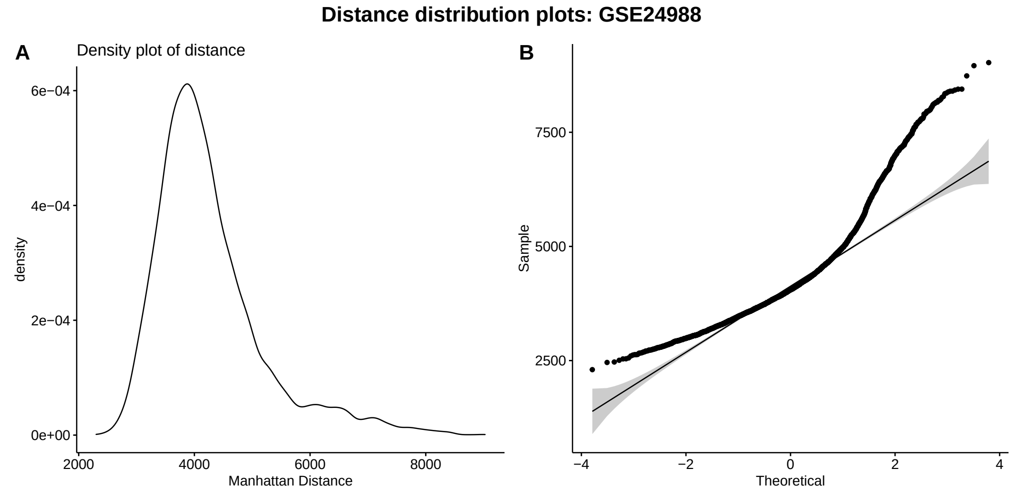

S52 Fig. Density and quantile-quantile plots for distances between samples in GSE24988. **A** Estimated density curve for distances. **B** Quantile-quantile plot between theoretical (standard normal) quantiles and sample distance quantiles.

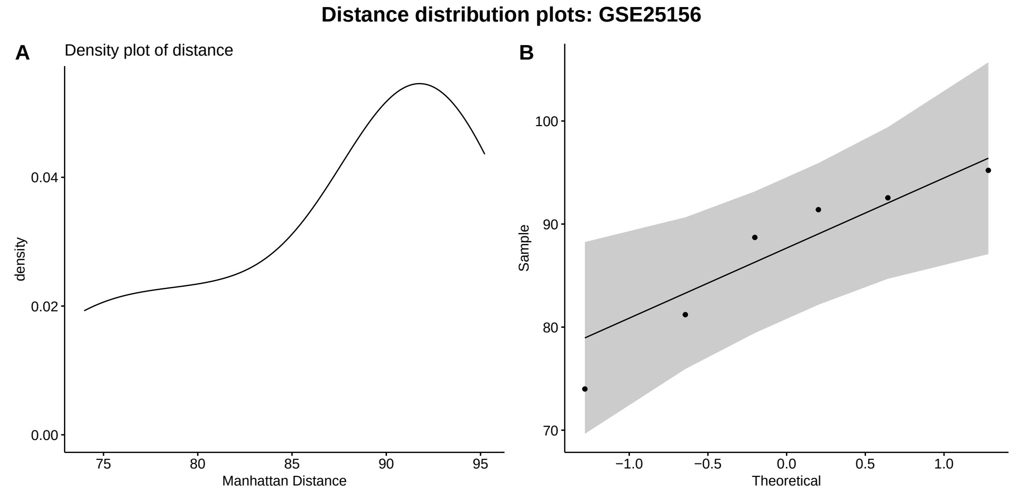

S53 Fig. Density and quantile-quantile plots for distances between samples in GSE25156. **A** Estimated density curve for distances. **B** Quantile-quantile plot between theoretical (standard normal) quantiles and sample distance quantiles.

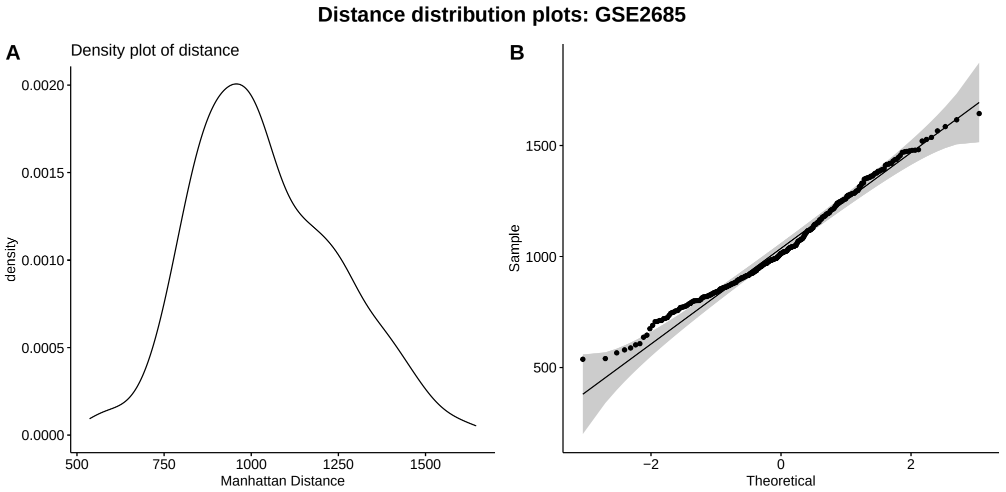

S54 Fig. Density and quantile-quantile plots for distances between samples in GSE2685. **A** Estimated density curve for distances. **B** Quantile-quantile plot between theoretical (standard normal) quantiles and sample distance quantiles.

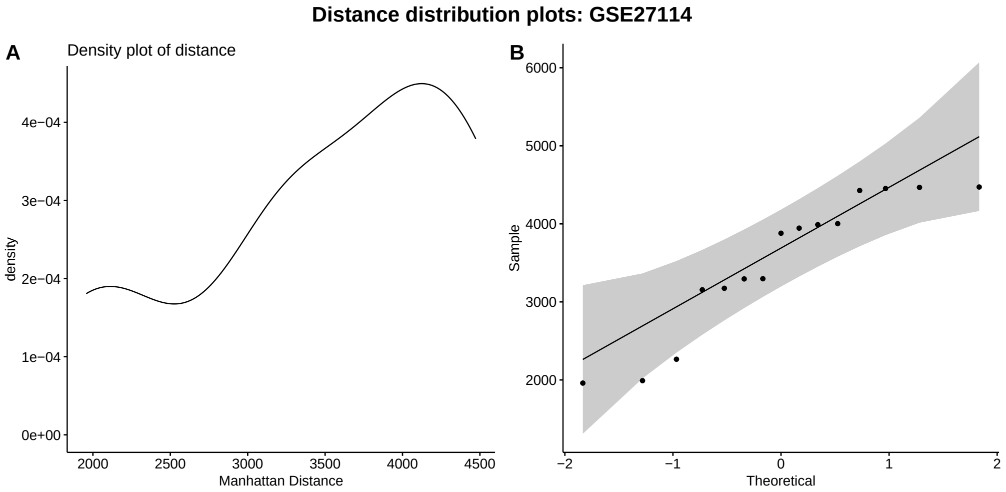

S55 Fig. Density and quantile-quantile plots for distances between samples in GSE27114. **A** Estimated density curve for distances. **B** Quantile-quantile plot between theoretical (standard normal) quantiles and sample distance quantiles.

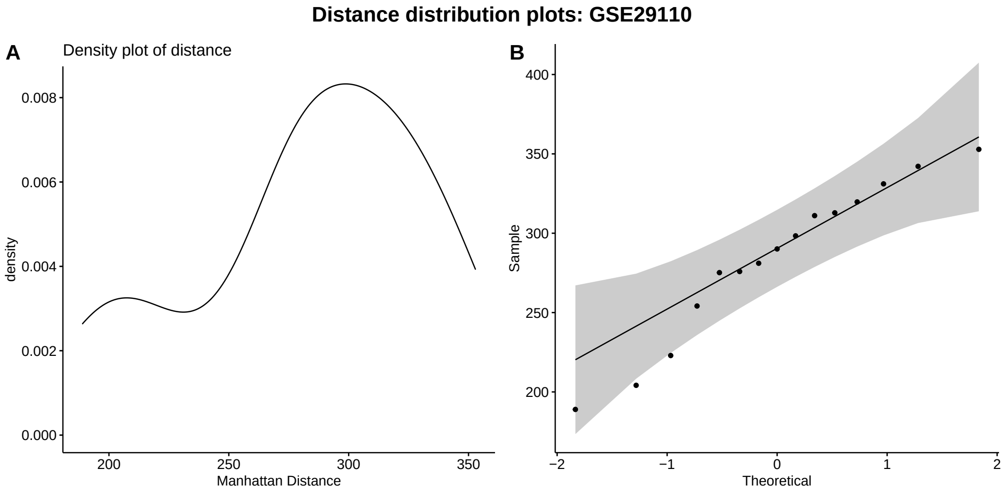

S56 Fig. Density and quantile-quantile plots for distances between samples in GSE29110. **A** Estimated density curve for distances. **B** Quantile-quantile plot between theoretical (standard normal) quantiles and sample distance quantiles.

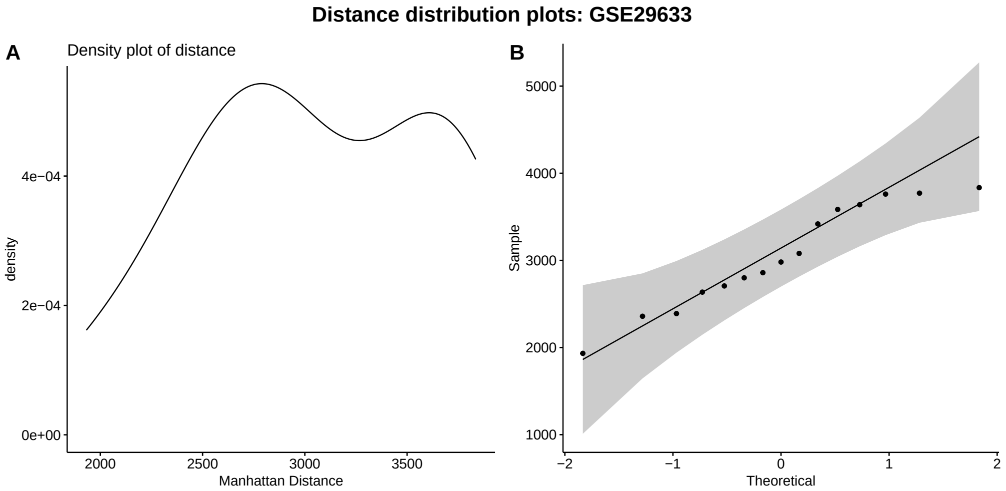

S57 Fig. Density and quantile-quantile plots for distances between samples in GSE29633. **A** Estimated density curve for distances. **B** Quantile-quantile plot between theoretical (standard normal) quantiles and sample distance quantiles.

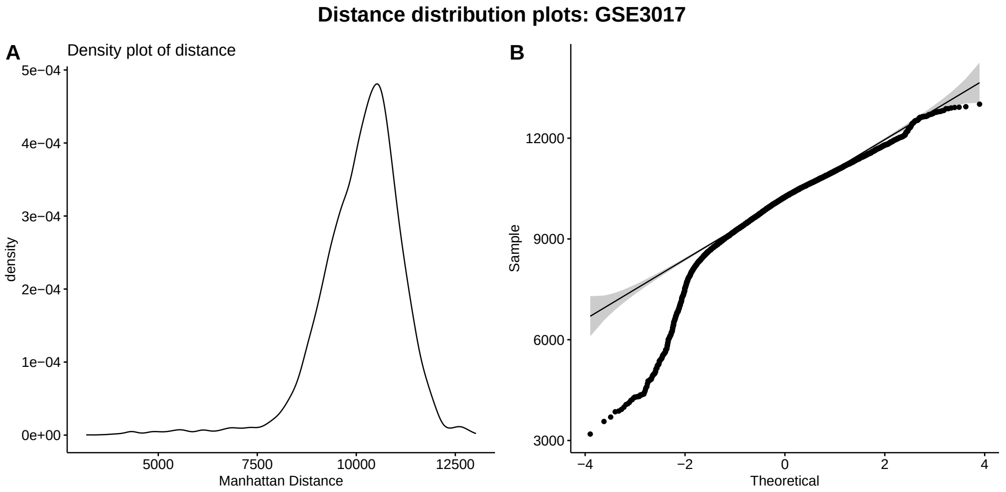

S58 Fig. Density and quantile-quantile plots for distances between samples in GSE3017. **A** Estimated density curve for distances. **B** Quantile-quantile plot between theoretical (standard normal) quantiles and sample distance quantiles.

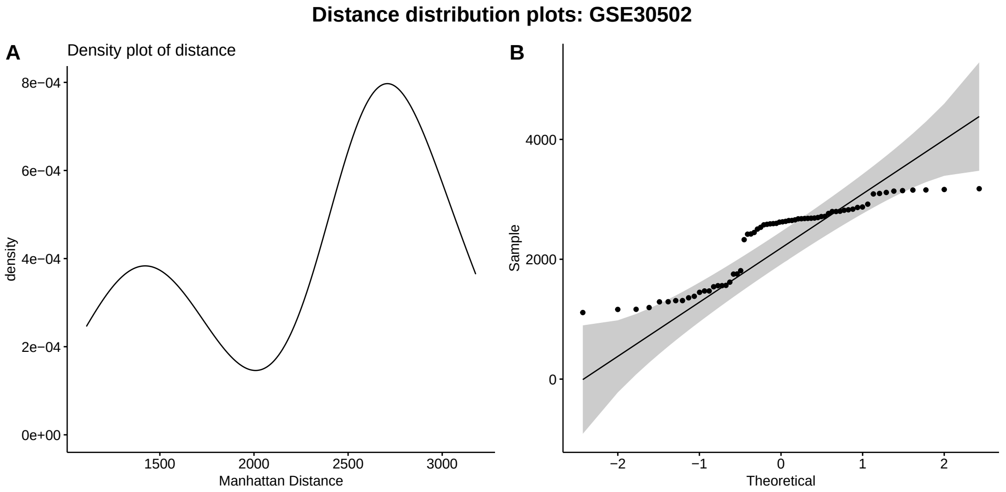

S59 Fig. Density and quantile-quantile plots for distances between samples in GSE30502. **A** Estimated density curve for distances. **B** Quantile-quantile plot between theoretical (standard normal) quantiles and sample distance quantiles.

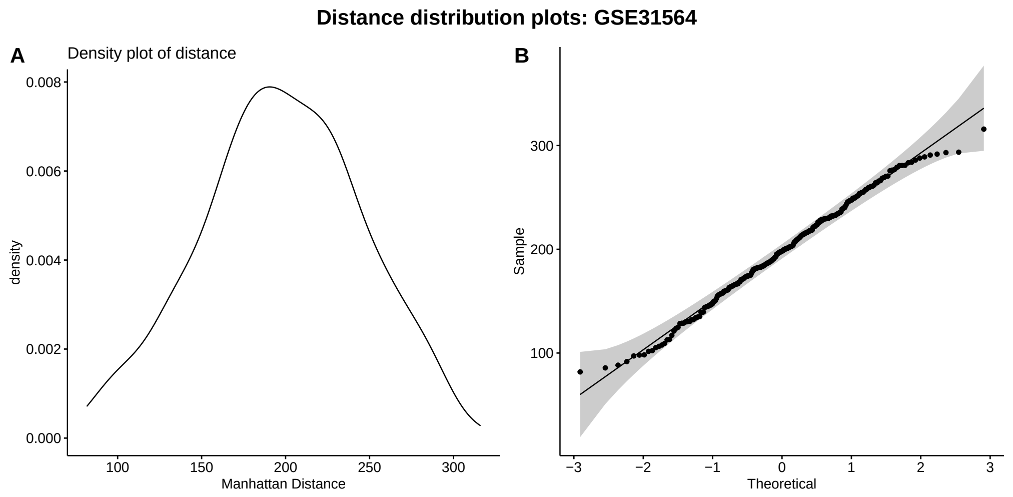

S60 Fig. Density and quantile-quantile plots for distances between samples in GSE31564. **A** Estimated density curve for distances. **B** Quantile-quantile plot between theoretical (standard normal) quantiles and sample distance quantiles.

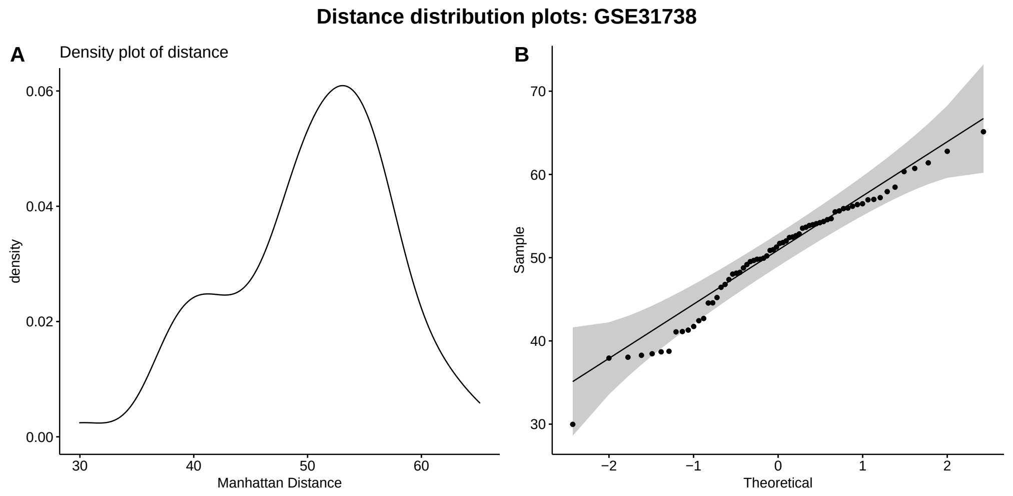

S61 Fig. Density and quantile-quantile plots for distances between samples in GSE31738. **A** Estimated density curve for distances. **B** Quantile-quantile plot between theoretical (standard normal) quantiles and sample distance quantiles.

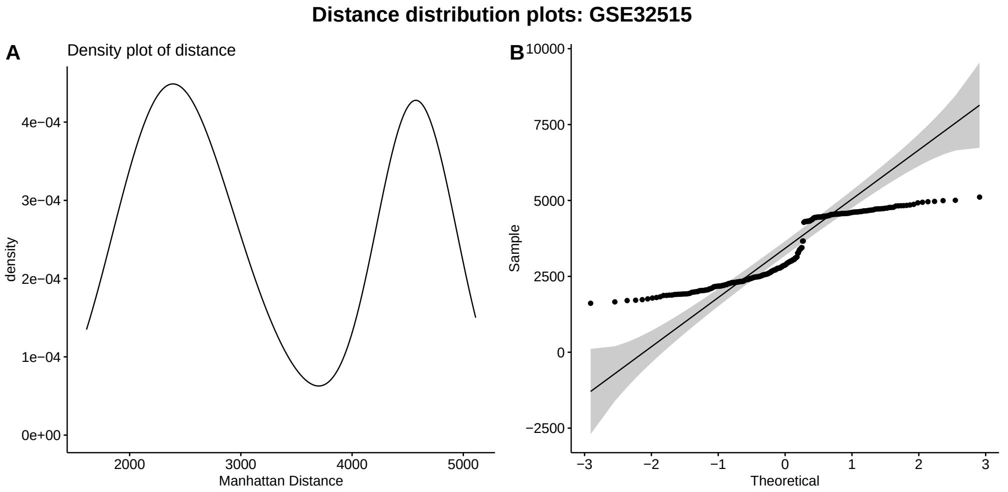

S62 Fig. Density and quantile-quantile plots for distances between samples in GSE32515. **A** Estimated density curve for distances. **B** Quantile-quantile plot between theoretical (standard normal) quantiles and sample distance quantiles.

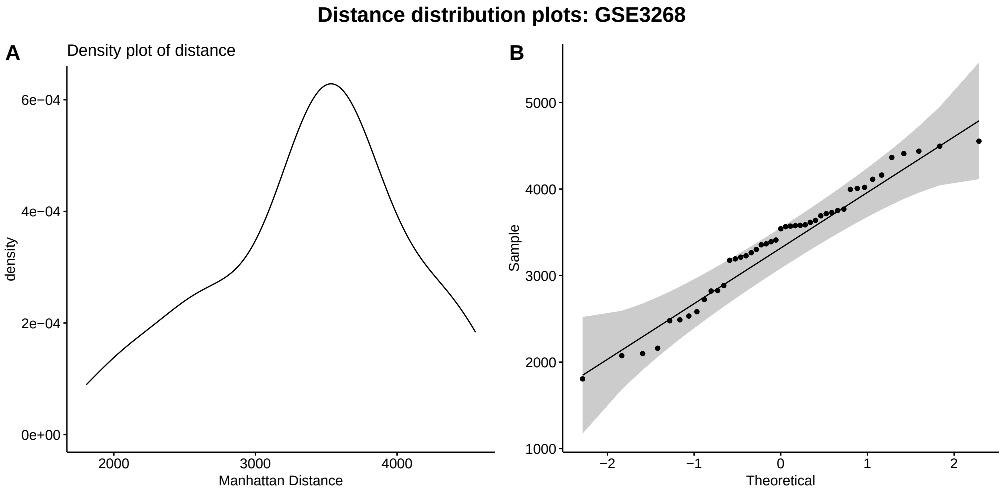

S63 Fig. Density and quantile-quantile plots for distances between samples in GSE3268. **A** Estimated density curve for distances. **B** Quantile-quantile plot between theoretical (standard normal) quantiles and sample distance quantiles.

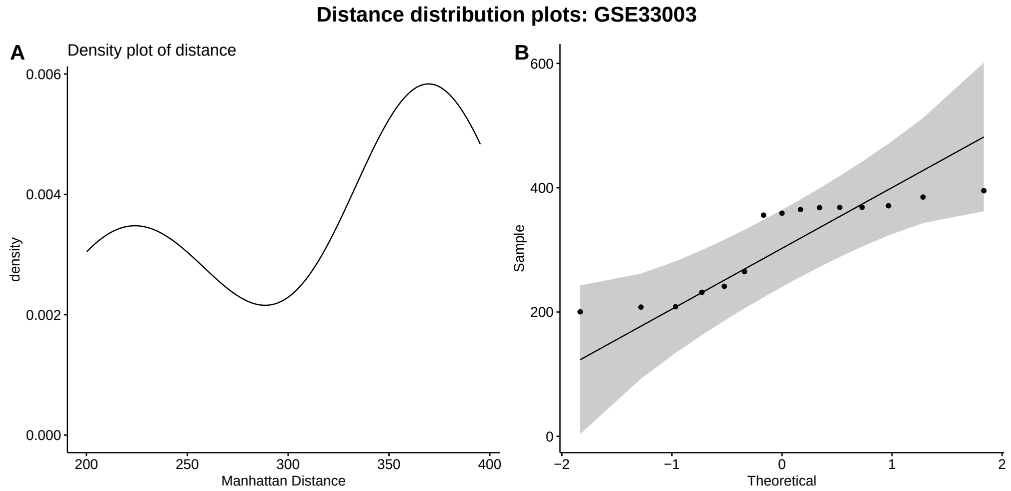

S64 Fig. Density and quantile-quantile plots for distances between samples in GSE33003. **A** Estimated density curve for distances. **B** Quantile-quantile plot between theoretical (standard normal) quantiles and sample distance quantiles.

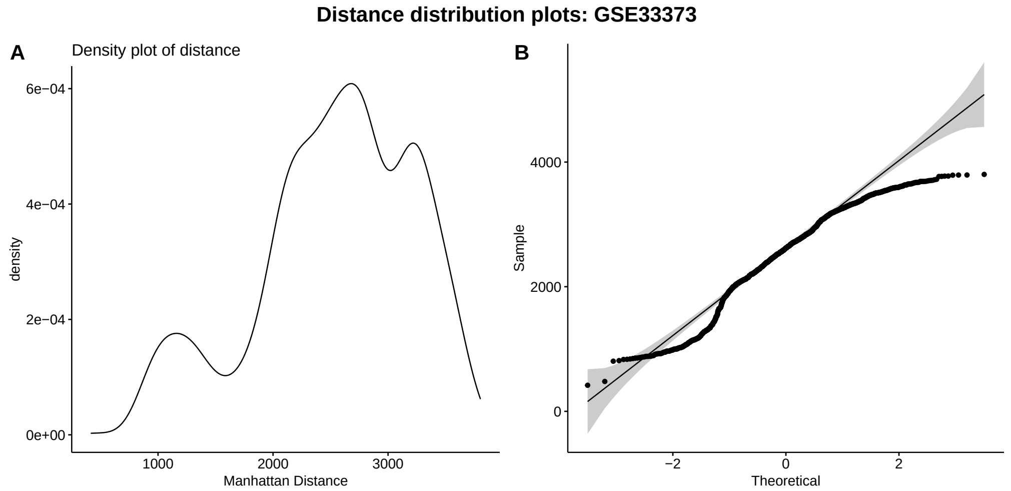

S65 Fig. Density and quantile-quantile plots for distances between samples in GSE33373. **A** Estimated density curve for distances. **B** Quantile-quantile plot between theoretical (standard normal) quantiles and sample distance quantiles.

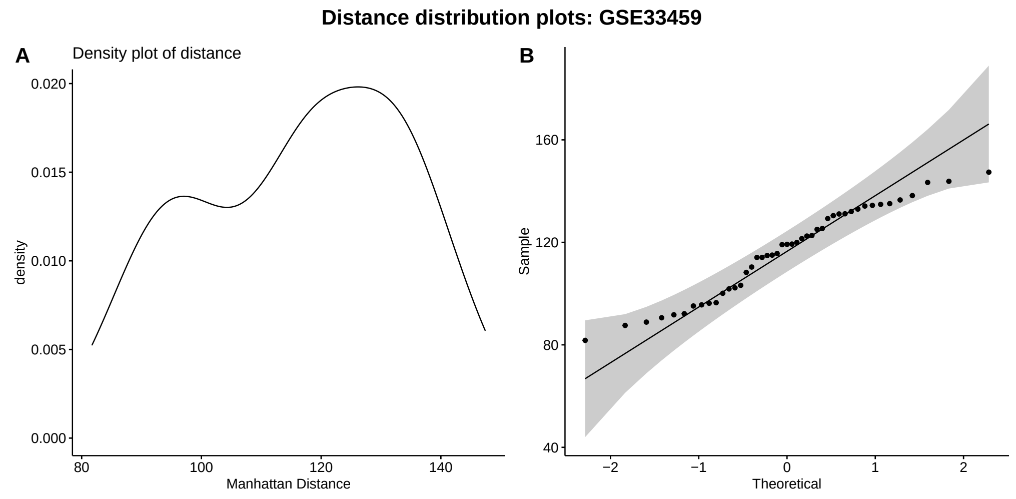

S66 Fig. Density and quantile-quantile plots for distances between samples in GSE33459. **A** Estimated density curve for distances. **B** Quantile-quantile plot between theoretical (standard normal) quantiles and sample distance quantiles.

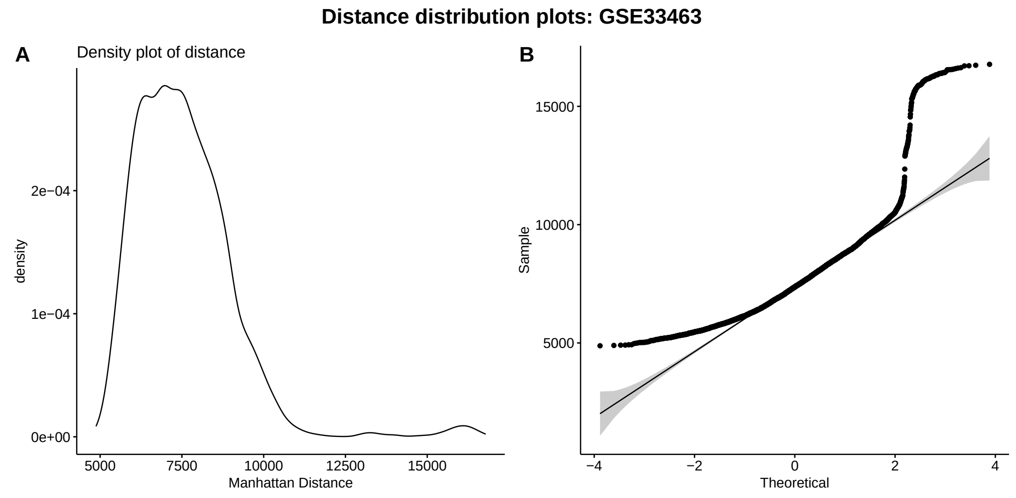

S67 Fig. Density and quantile-quantile plots for distances between samples in GSE33463. **A** Estimated density curve for distances. **B** Quantile-quantile plot between theoretical (standard normal) quantiles and sample distance quantiles.

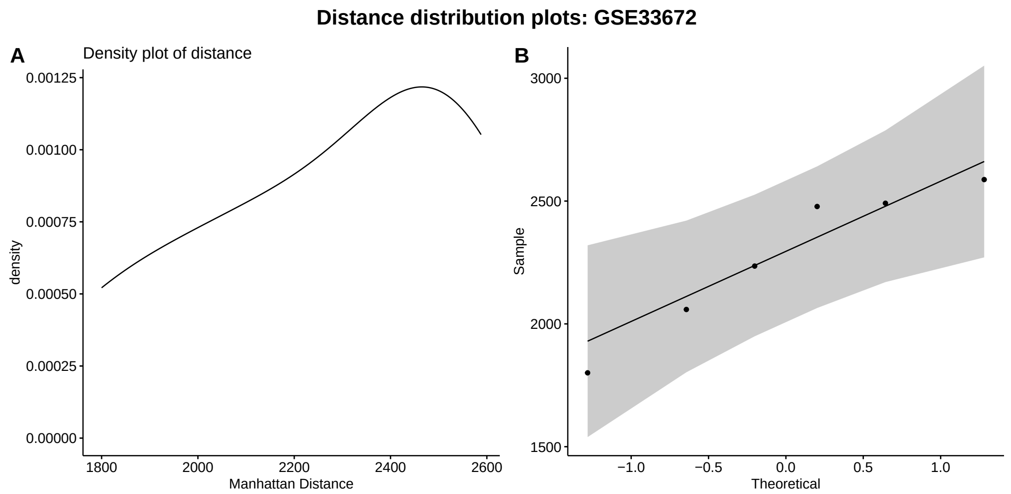

S68 Fig. Density and quantile-quantile plots for distances between samples in GSE33672. **A** Estimated density curve for distances. **B** Quantile-quantile plot between theoretical (standard normal) quantiles and sample distance quantiles.

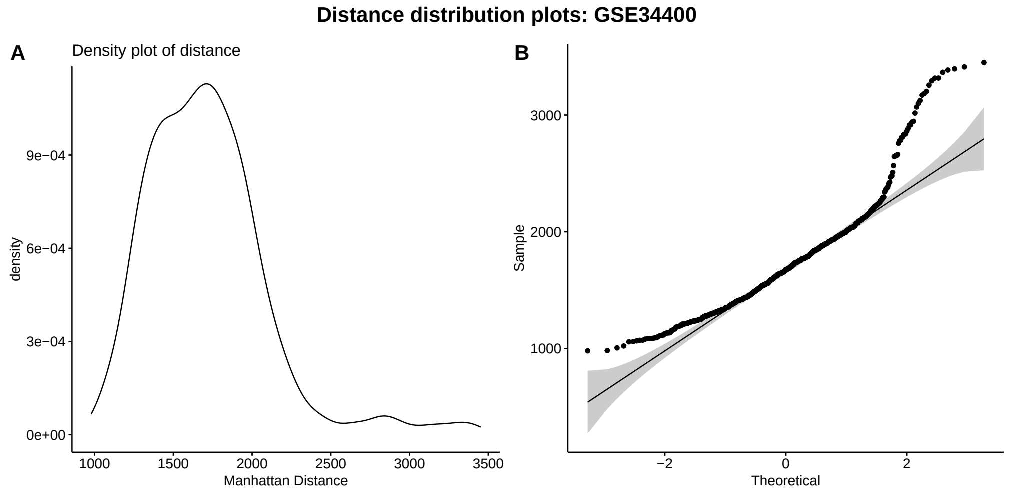

S69 Fig. Density and quantile-quantile plots for distances between samples in GSE34400. **A** Estimated density curve for distances. **B** Quantile-quantile plot between theoretical (standard normal) quantiles and sample distance quantiles.

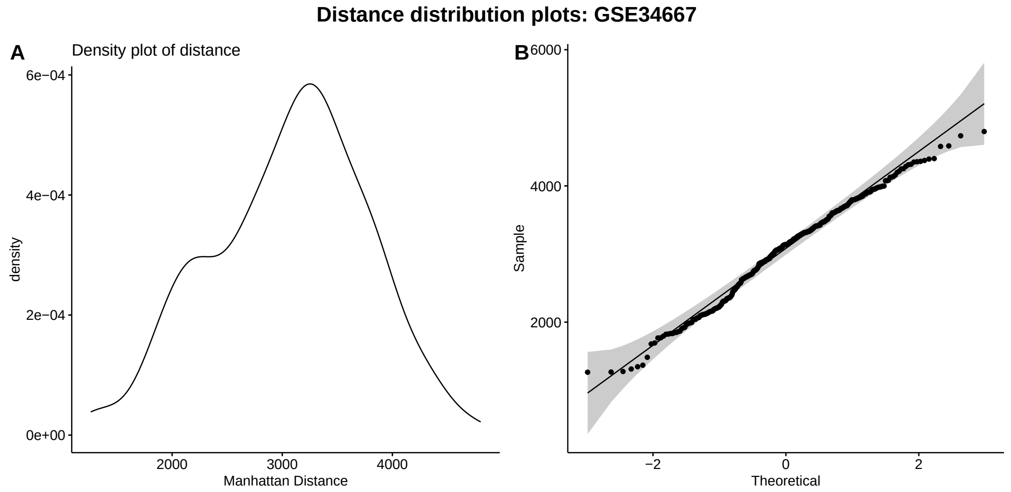

S70 Fig. Density and quantile-quantile plots for distances between samples in GSE34667. **A** Estimated density curve for distances. **B** Quantile-quantile plot between theoretical (standard normal) quantiles and sample distance quantiles.

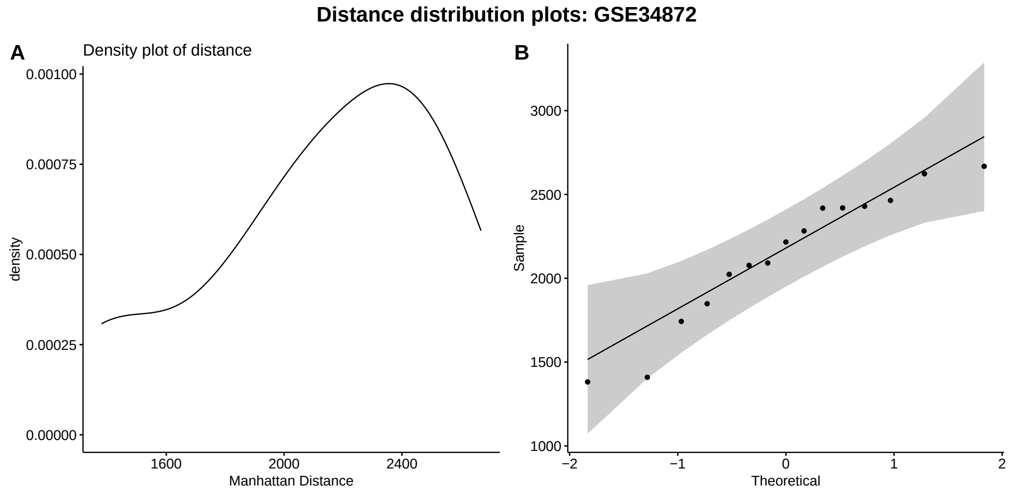

S71 Fig. Density and quantile-quantile plots for distances between samples in GSE34872. **A** Estimated density curve for distances. **B** Quantile-quantile plot between theoretical (standard normal) quantiles and sample distance quantiles.

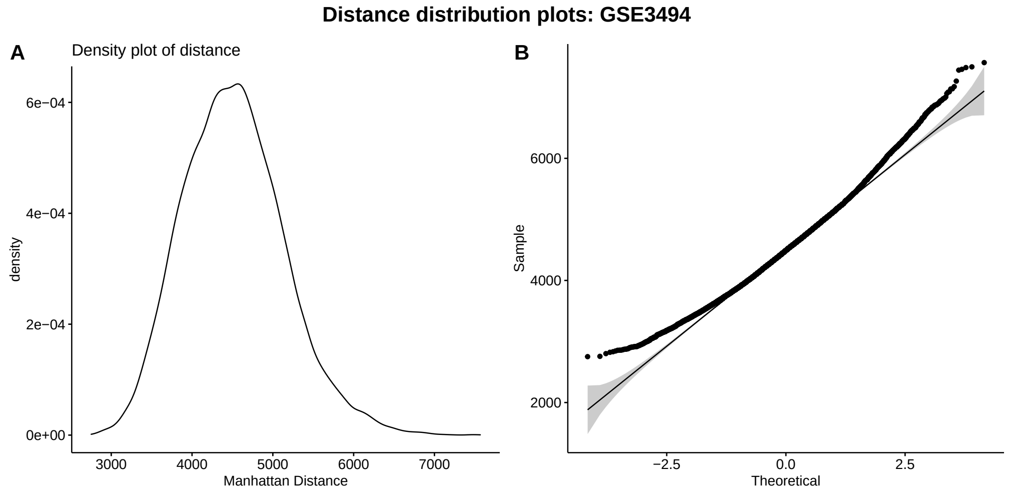

S72 Fig. Density and quantile-quantile plots for distances between samples in GSE3494. **A** Estimated density curve for distances. **B** Quantile-quantile plot between theoretical (standard normal) quantiles and sample distance quantiles.

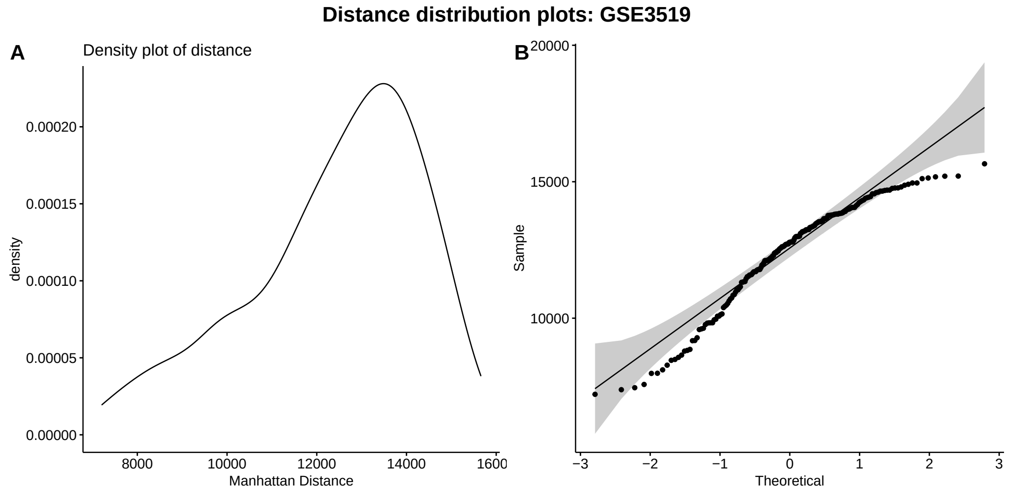

S73 Fig. Density and quantile-quantile plots for distances between samples in GSE3519. **A** Estimated density curve for distances. **B** Quantile-quantile plot between theoretical (standard normal) quantiles and sample distance quantiles.

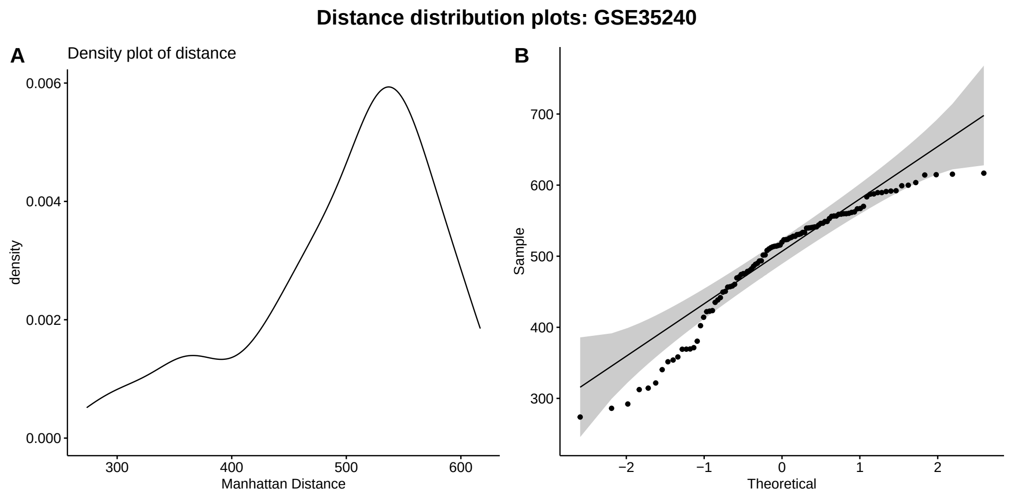

S74 Fig. Density and quantile-quantile plots for distances between samples in GSE35240. **A** Estimated density curve for distances. **B** Quantile-quantile plot between theoretical (standard normal) quantiles and sample distance quantiles.

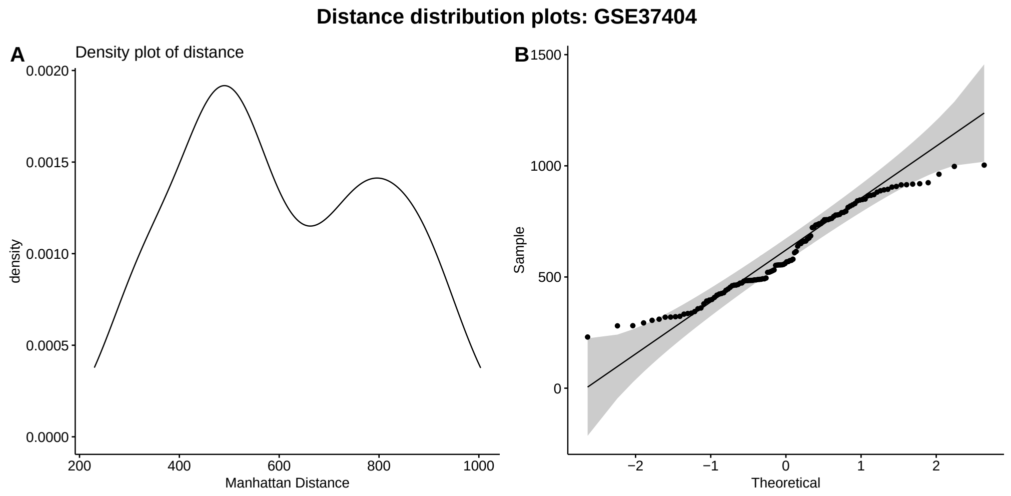

S75 Fig. Density and quantile-quantile plots for distances between samples in GSE37404. **A** Estimated density curve for distances. **B** Quantile-quantile plot between theoretical (standard normal) quantiles and sample distance quantiles.

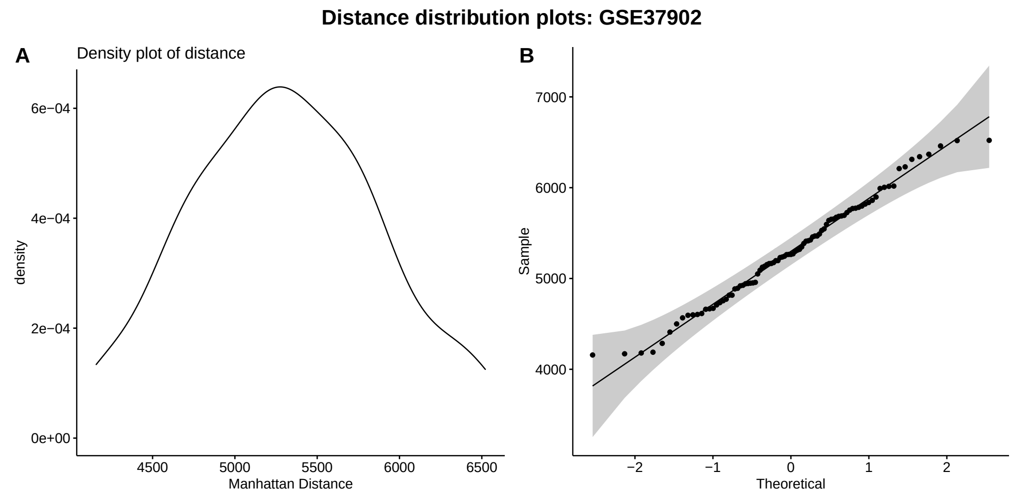

S76 Fig. Density and quantile-quantile plots for distances between samples in GSE37902. **A** Estimated density curve for distances. **B** Quantile-quantile plot between theoretical (standard normal) quantiles and sample distance quantiles.

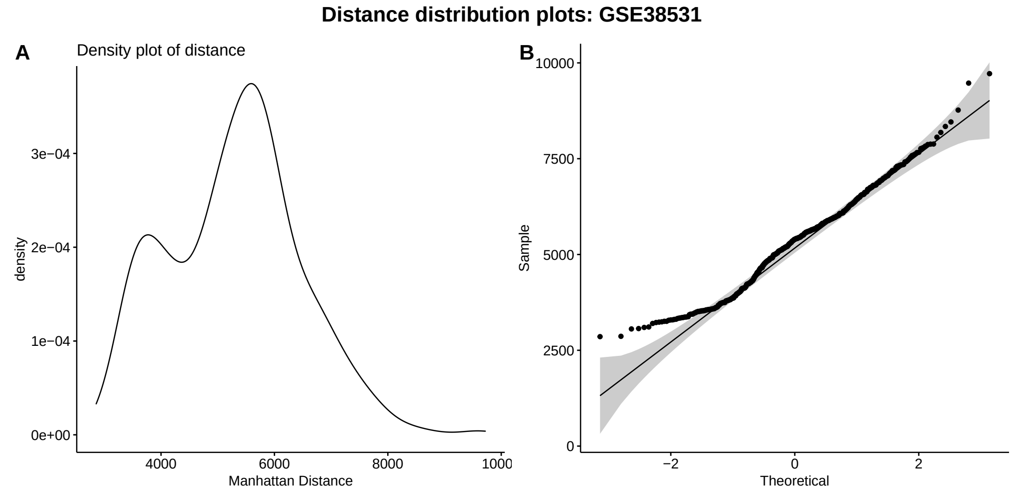

S77 Fig. Density and quantile-quantile plots for distances between samples in GSE38531. **A** Estimated density curve for distances. **B** Quantile-quantile plot between theoretical (standard normal) quantiles and sample distance quantiles.

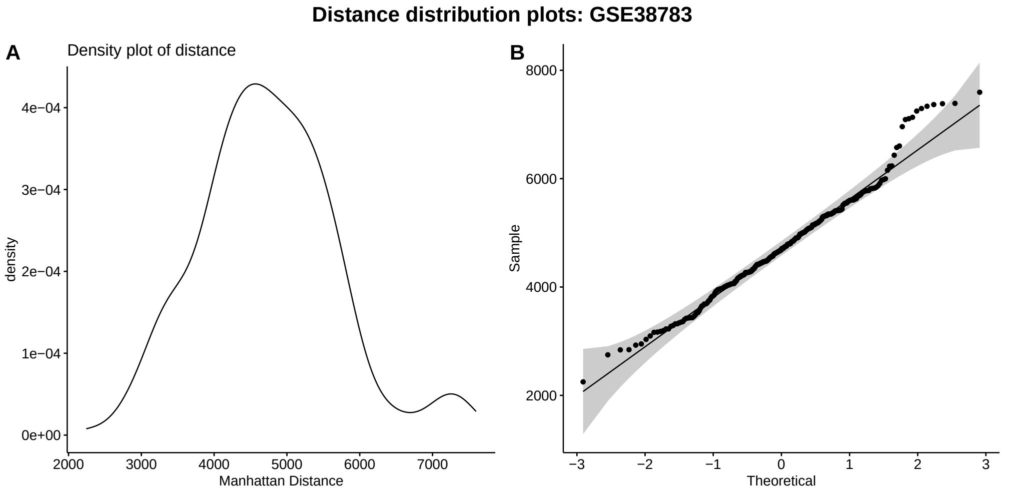

S78 Fig. Density and quantile-quantile plots for distances between samples in GSE38783. **A** Estimated density curve for distances. **B** Quantile-quantile plot between theoretical (standard normal) quantiles and sample distance quantiles.

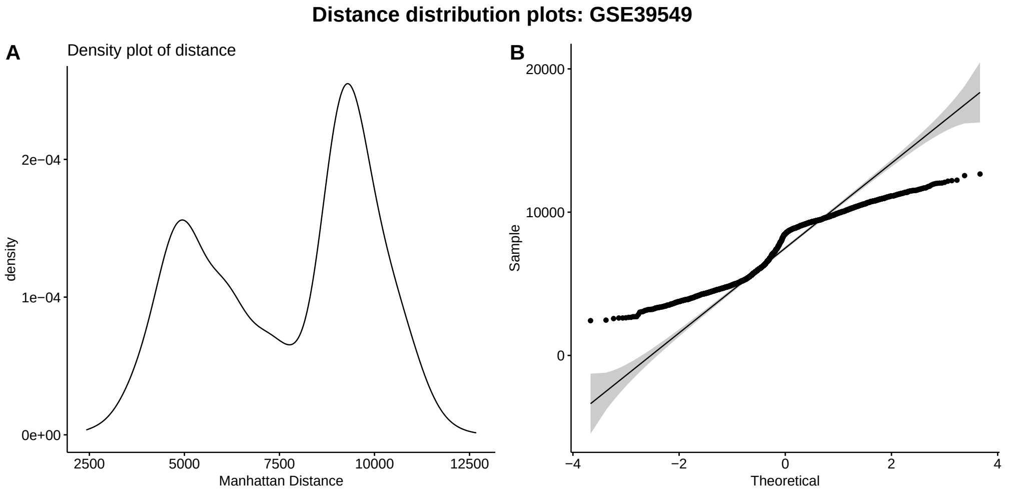

S79 Fig. Density and quantile-quantile plots for distances between samples in GSE39549. **A** Estimated density curve for distances. **B** Quantile-quantile plot between theoretical (standard normal) quantiles and sample distance quantiles.

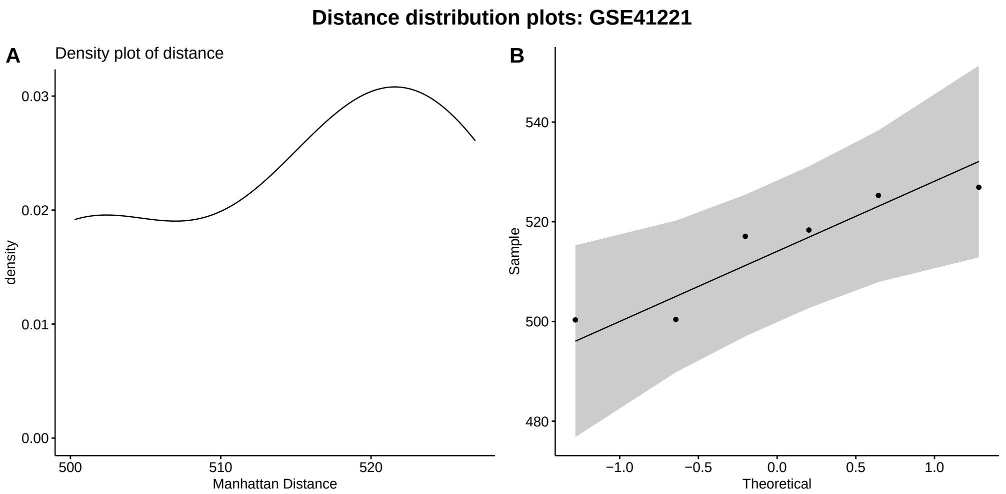

S80 Fig. Density and quantile-quantile plots for distances between samples in GSE41221. **A** Estimated density curve for distances. **B** Quantile-quantile plot between theoretical (standard normal) quantiles and sample distance quantiles.

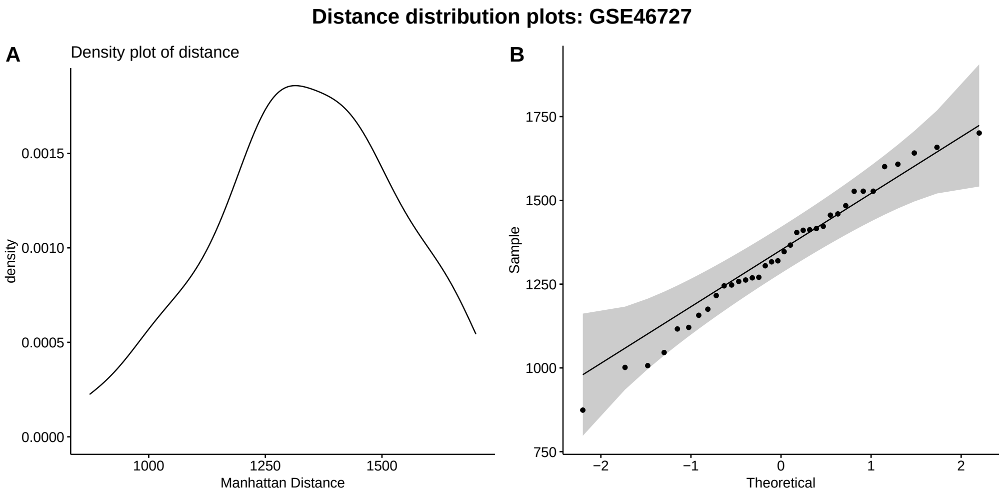

S81 Fig. Density and quantile-quantile plots for distances between samples in GSE46727. **A** Estimated density curve for distances. **B** Quantile-quantile plot between theoretical (standard normal) quantiles and sample distance quantiles.

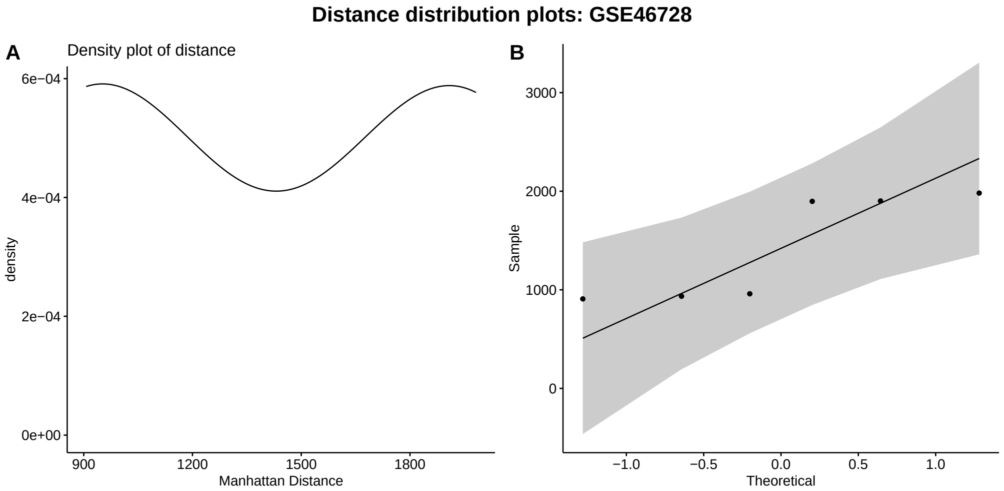

S82 Fig. Density and quantile-quantile plots for distances between samples in GSE46728. **A** Estimated density curve for distances. **B** Quantile-quantile plot between theoretical (standard normal) quantiles and sample distance quantiles.

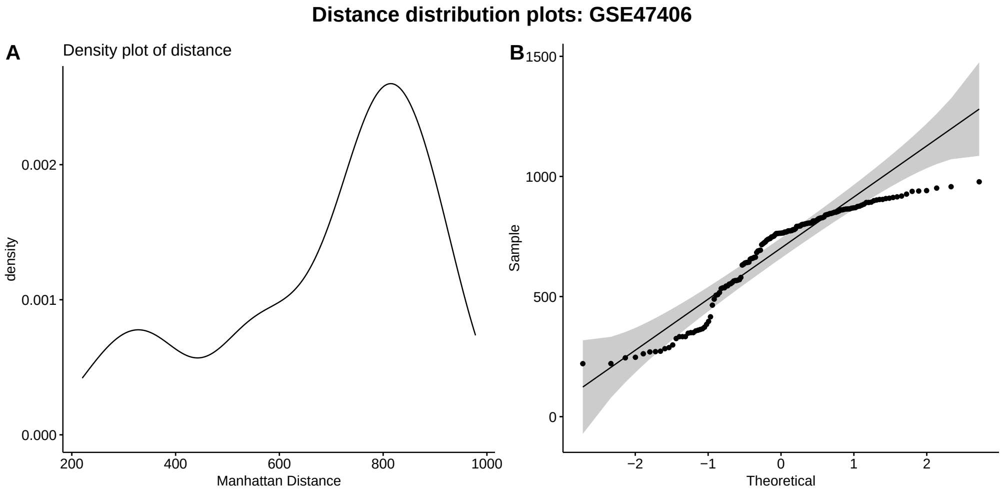

S83 Fig. Density and quantile-quantile plots for distances between samples in GSE47406. **A** Estimated density curve for distances. **B** Quantile-quantile plot between theoretical (standard normal) quantiles and sample distance quantiles.

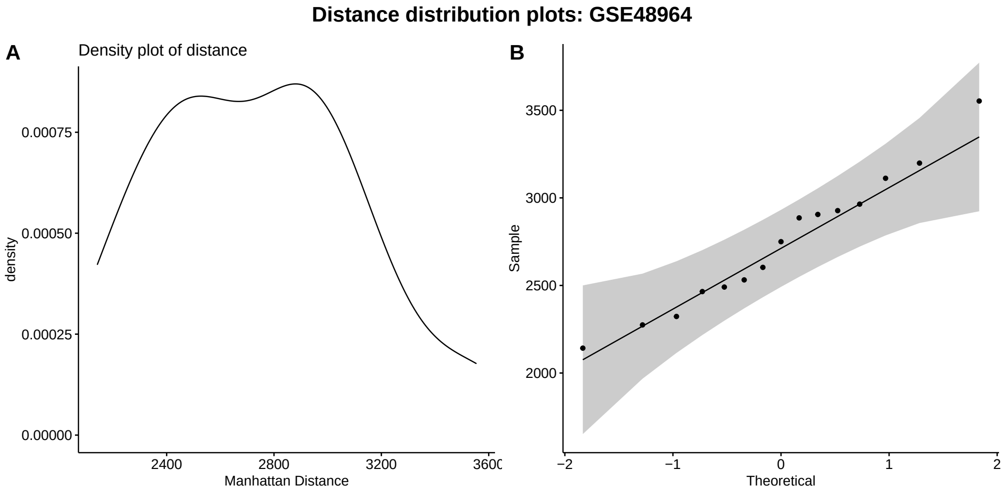

S84 Fig. Density and quantile-quantile plots for distances between samples in GSE48964. **A** Estimated density curve for distances. **B** Quantile-quantile plot between theoretical (standard normal) quantiles and sample distance quantiles.

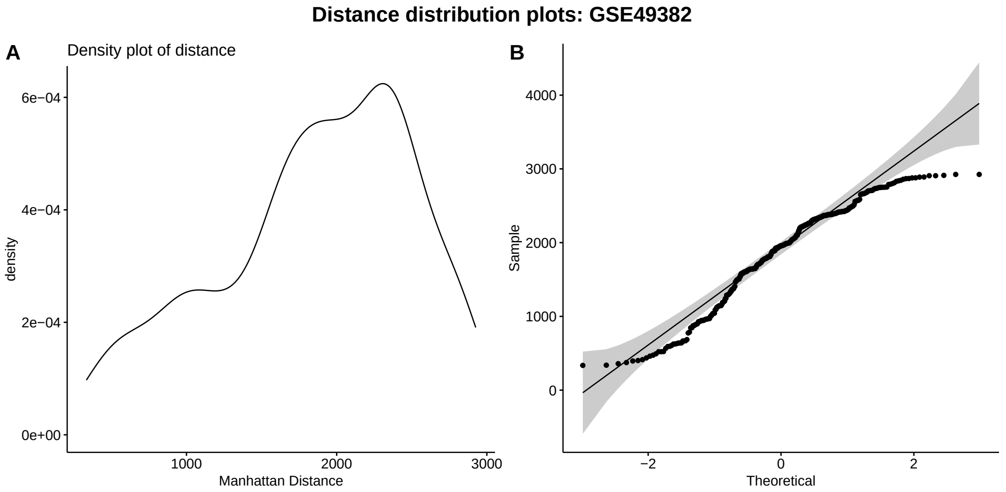

S85 Fig. Density and quantile-quantile plots for distances between samples in GSE49382. **A** Estimated density curve for distances. **B** Quantile-quantile plot between theoretical (standard normal) quantiles and sample distance quantiles.

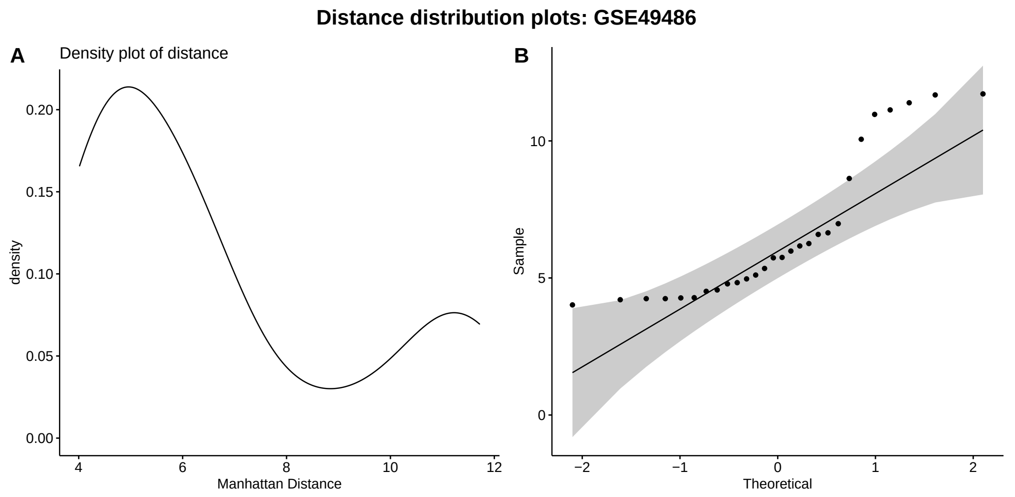

S86 Fig. Density and quantile-quantile plots for distances between samples in GSE49486. **A** Estimated density curve for distances. **B** Quantile-quantile plot between theoretical (standard normal) quantiles and sample distance quantiles.

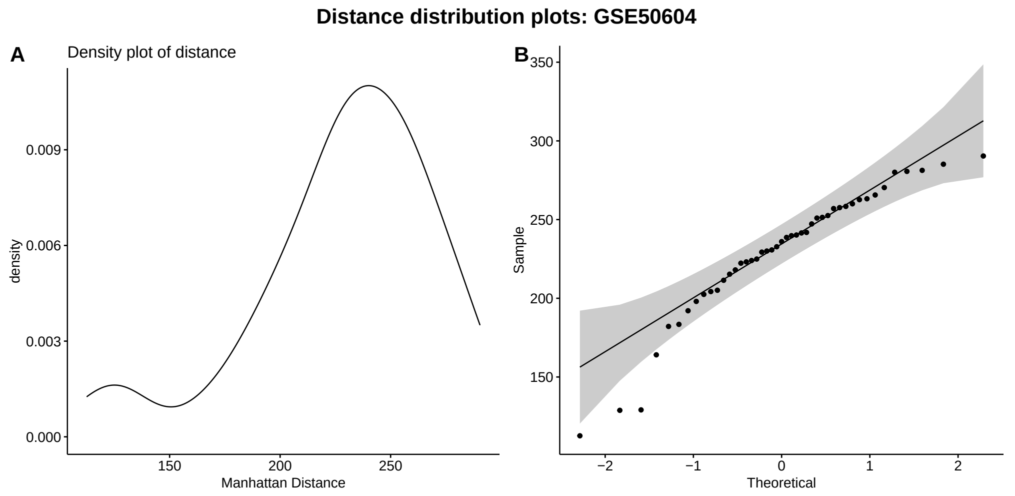

S87 Fig. Density and quantile-quantile plots for distances between samples in GSE50604. **A** Estimated density curve for distances. **B** Quantile-quantile plot between theoretical (standard normal) quantiles and sample distance quantiles.

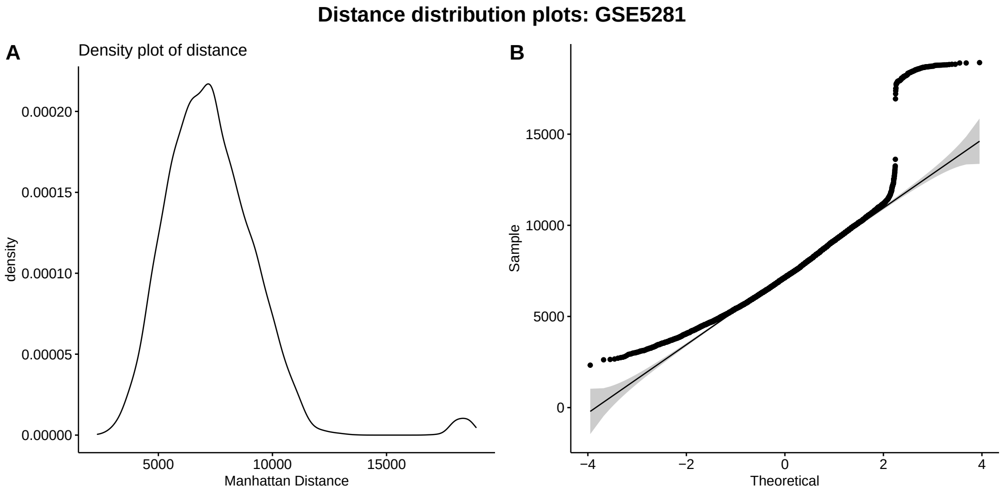

S88 Fig. Density and quantile-quantile plots for distances between samples in GSE5281. **A** Estimated density curve for distances. **B** Quantile-quantile plot between theoretical (standard normal) quantiles and sample distance quantiles.

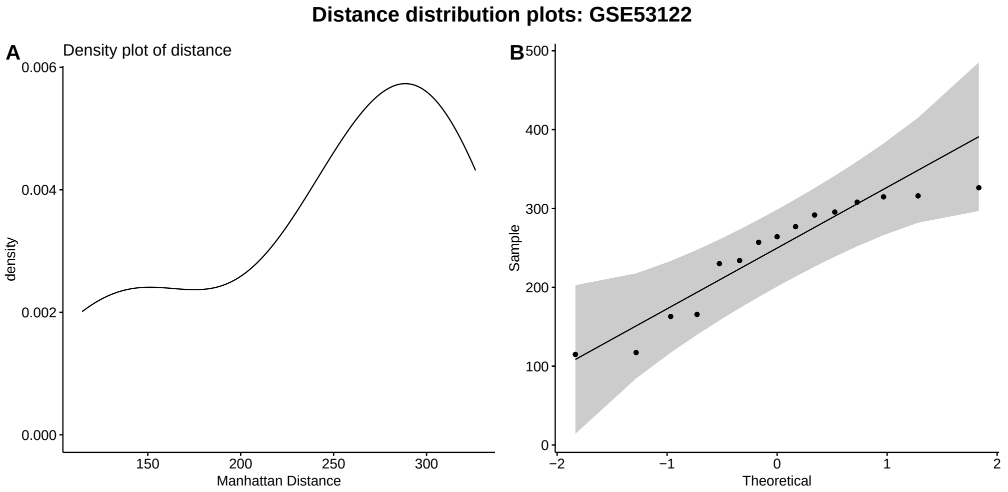

S89 Fig. Density and quantile-quantile plots for distances between samples in GSE53122. **A** Estimated density curve for distances. **B** Quantile-quantile plot between theoretical (standard normal) quantiles and sample distance quantiles.

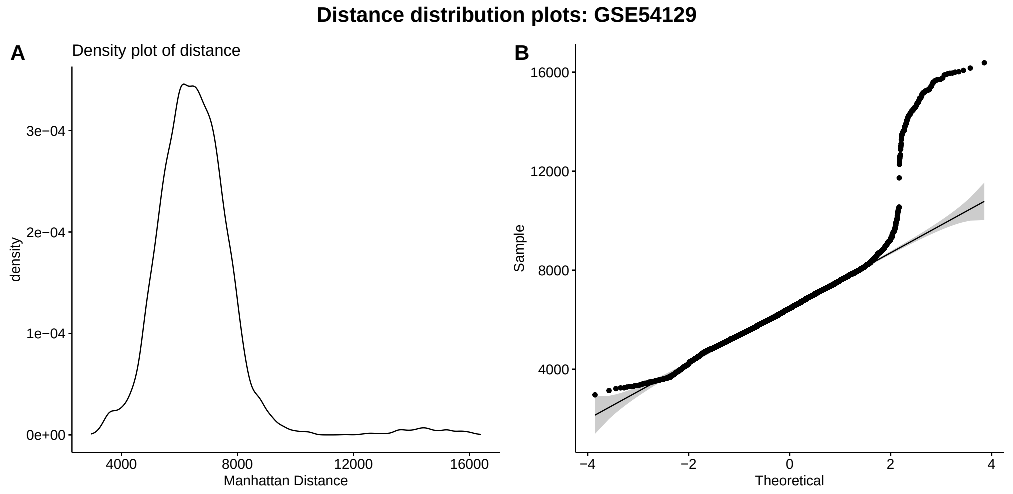

S90 Fig. Density and quantile-quantile plots for distances between samples in GSE54129. **A** Estimated density curve for distances. **B** Quantile-quantile plot between theoretical (standard normal) quantiles and sample distance quantiles.

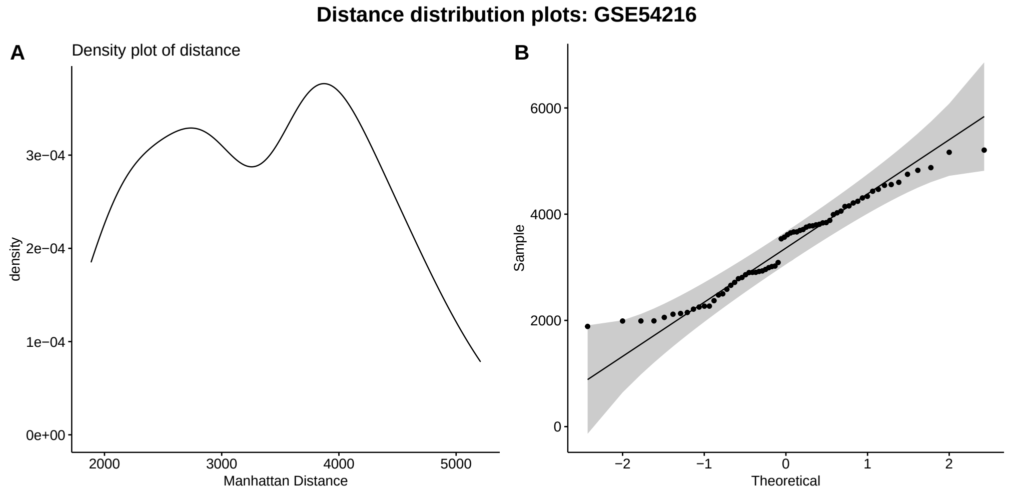

S91 Fig. Density and quantile-quantile plots for distances between samples in GSE54216. **A** Estimated density curve for distances. **B** Quantile-quantile plot between theoretical (standard normal) quantiles and sample distance quantiles.

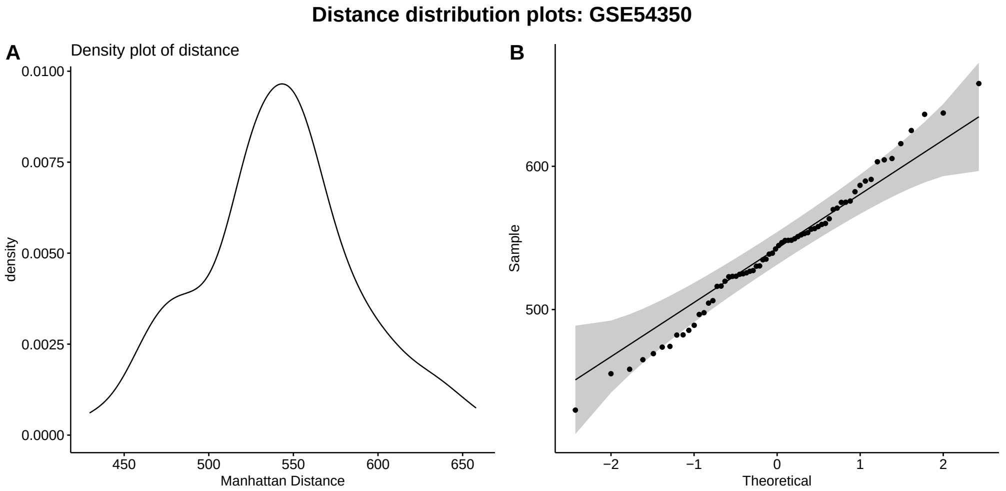

S92 Fig. Density and quantile-quantile plots for distances between samples in GSE54350. **A** Estimated density curve for distances. **B** Quantile-quantile plot between theoretical (standard normal) quantiles and sample distance quantiles.

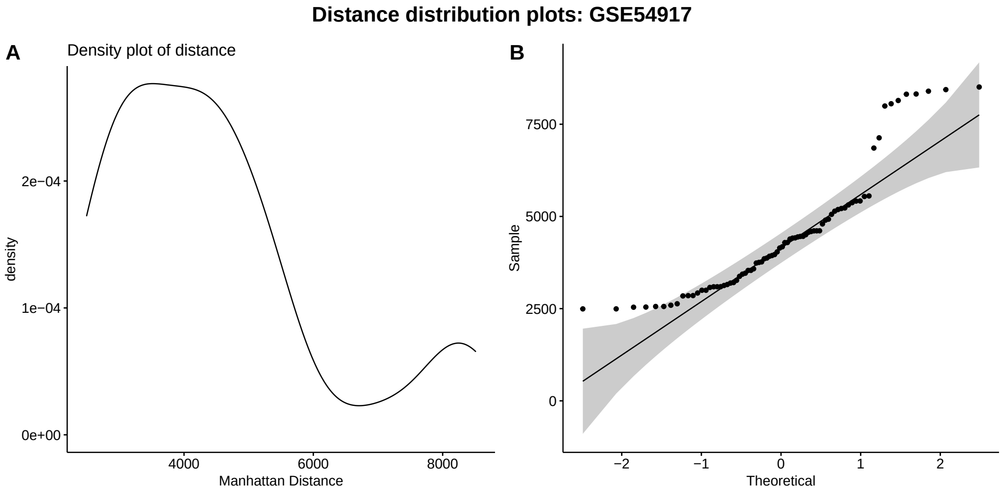

S93 Fig. Density and quantile-quantile plots for distances between samples in GSE54917. **A** Estimated density curve for distances. **B** Quantile-quantile plot between theoretical (standard normal) quantiles and sample distance quantiles.

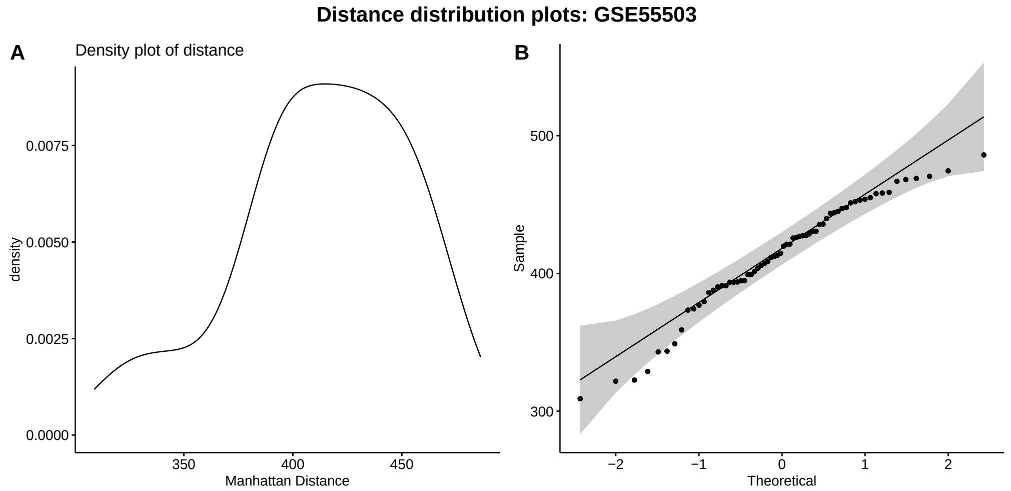

S94 Fig. Density and quantile-quantile plots for distances between samples in GSE55503. **A** Estimated density curve for distances. **B** Quantile-quantile plot between theoretical (standard normal) quantiles and sample distance quantiles.

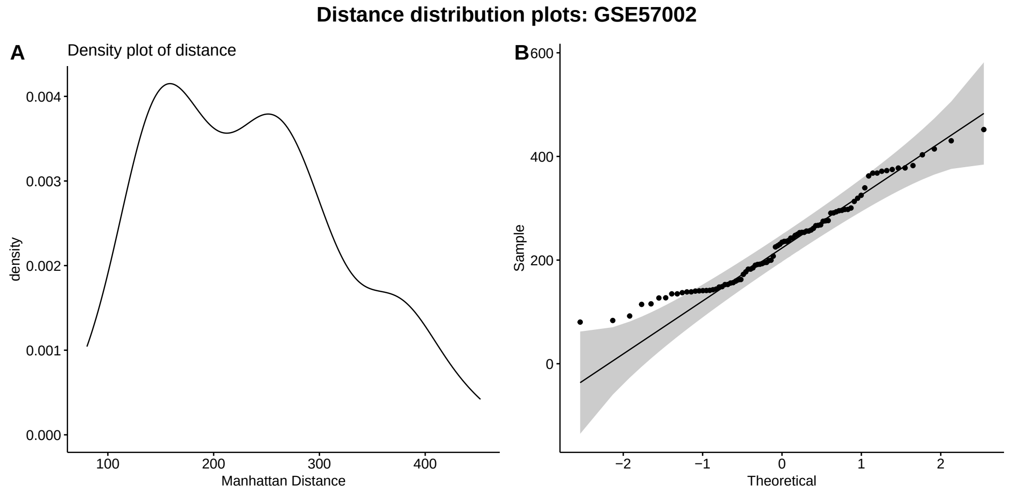

S95 Fig. Density and quantile-quantile plots for distances between samples in GSE57002. **A** Estimated density curve for distances. **B** Quantile-quantile plot between theoretical (standard normal) quantiles and sample distance quantiles.

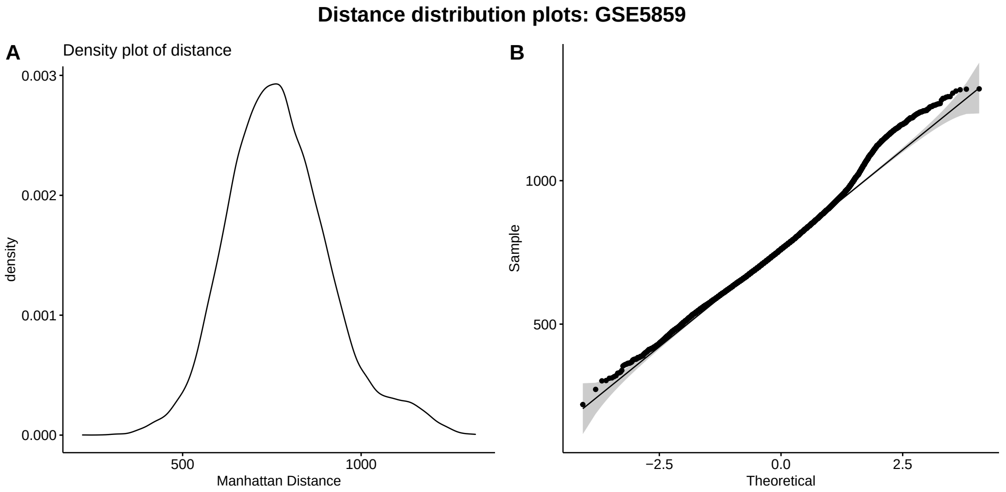

S96 Fig. Density and quantile-quantile plots for distances between samples in GSE5859. **A** Estimated density curve for distances. **B** Quantile-quantile plot between theoretical (standard normal) quantiles and sample distance quantiles.

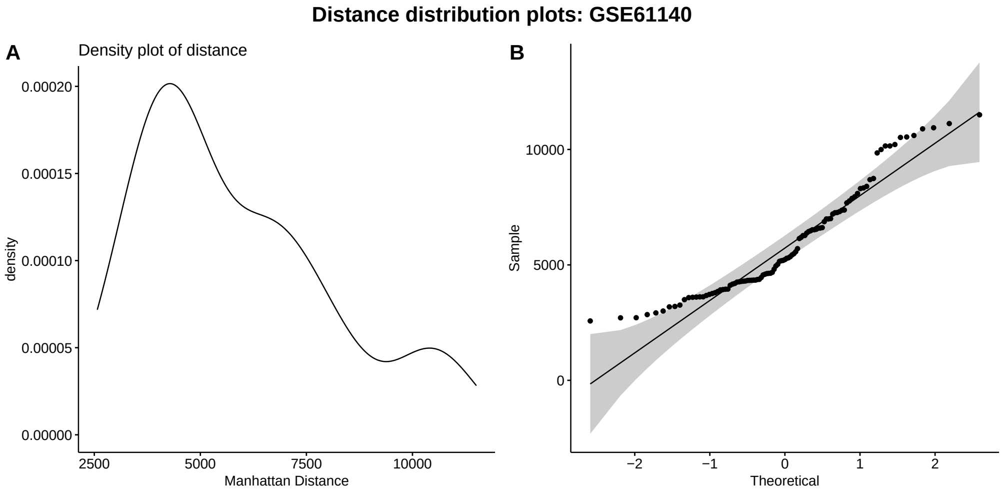

S97 Fig. Density and quantile-quantile plots for distances between samples in GSE61140. **A** Estimated density curve for distances. **B** Quantile-quantile plot between theoretical (standard normal) quantiles and sample distance quantiles.

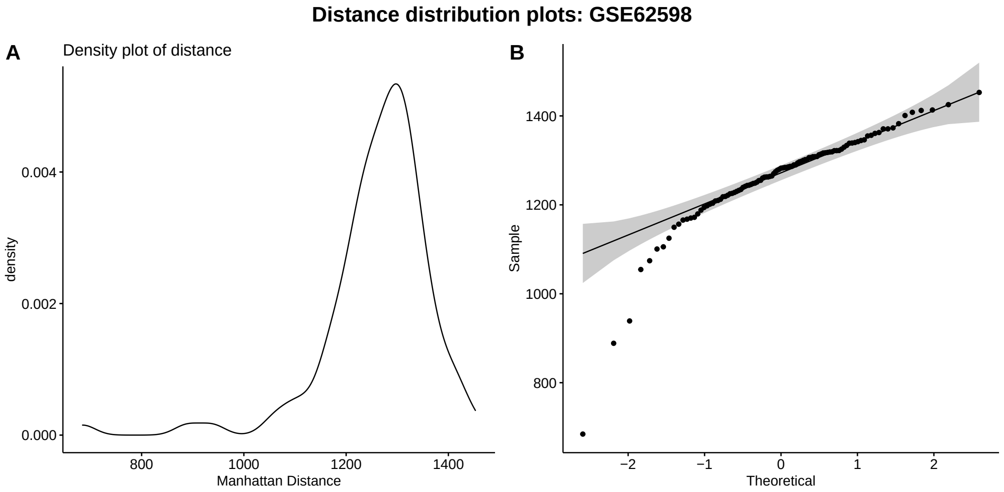

S98 Fig. Density and quantile-quantile plots for distances between samples in GSE62598. **A** Estimated density curve for distances. **B** Quantile-quantile plot between theoretical (standard normal) quantiles and sample distance quantiles.

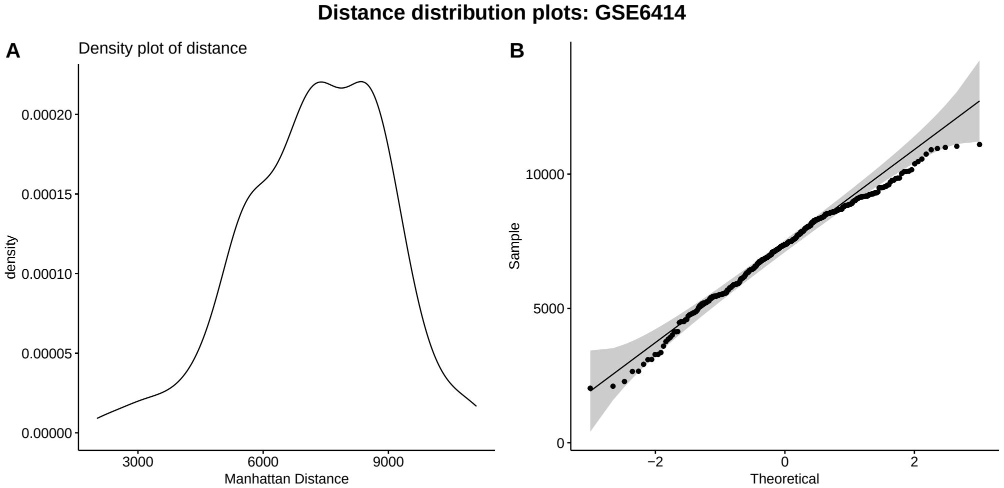

S99 Fig. Density and quantile-quantile plots for distances between samples in GSE6414. **A** Estimated density curve for distances. **B** Quantile-quantile plot between theoretical (standard normal) quantiles and sample distance quantiles.

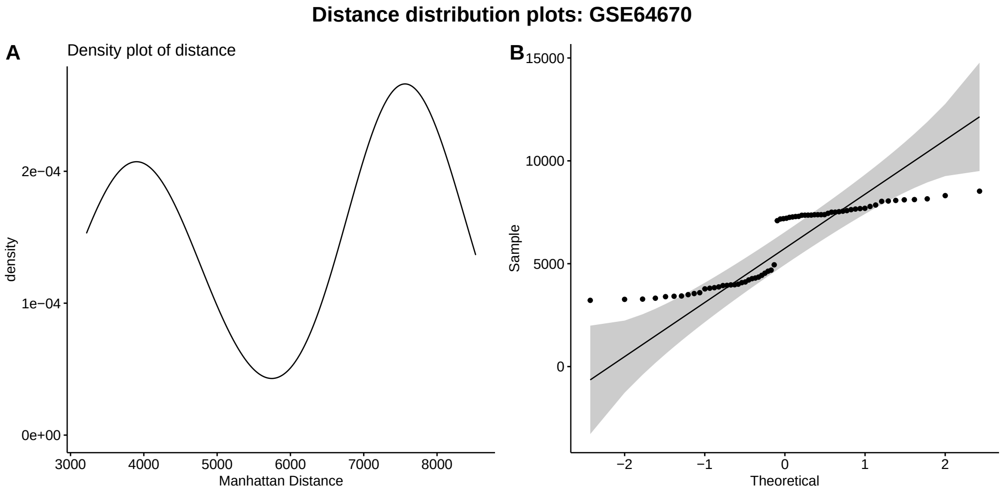

S100 Fig. Density and quantile-quantile plots for distances between samples in GSE64670. **A** Estimated density curve for distances. **B** Quantile-quantile plot between theoretical (standard normal) quantiles and sample distance quantiles.

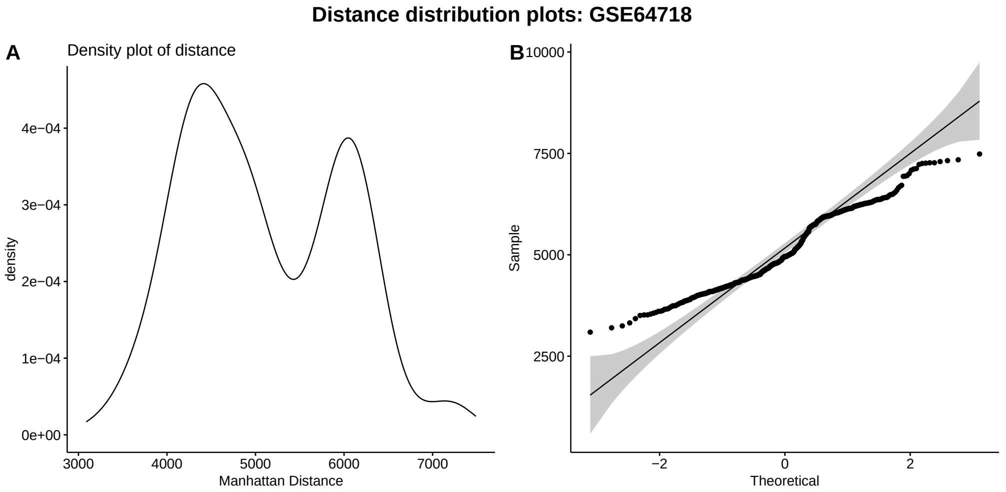

S101 Fig. Density and quantile-quantile plots for distances between samples in GSE64718. **A** Estimated density curve for distances. **B** Quantile-quantile plot between theoretical (standard normal) quantiles and sample distance quantiles.

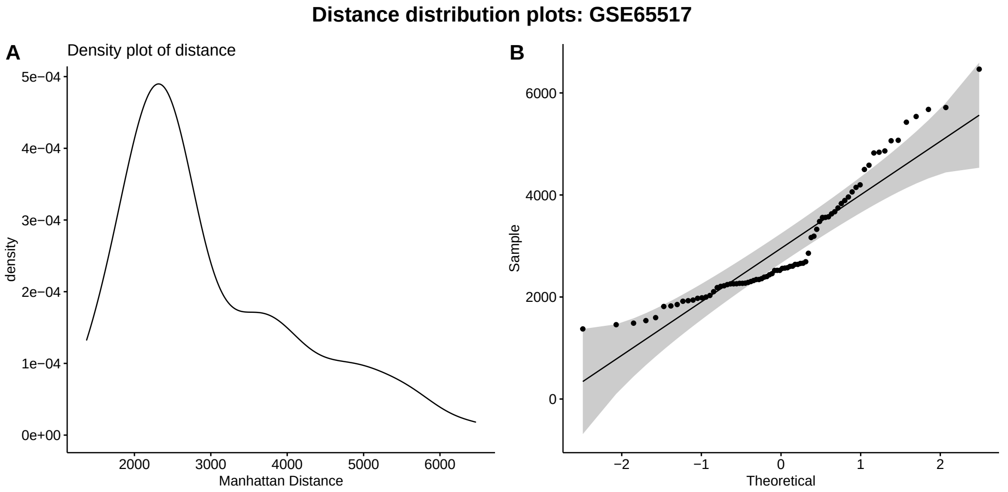

S102 Fig. Density and quantile-quantile plots for distances between samples in GSE65517. **A** Estimated density curve for distances. **B** Quantile-quantile plot between theoretical (standard normal) quantiles and sample distance quantiles.

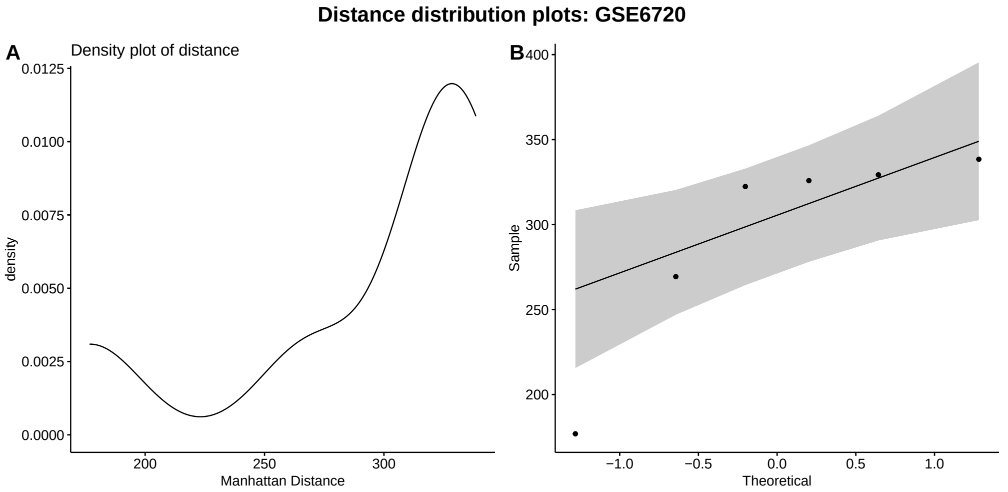

S103 Fig. Density and quantile-quantile plots for distances between samples in GSE6720. **A** Estimated density curve for distances. **B** Quantile-quantile plot between theoretical (standard normal) quantiles and sample distance quantiles.

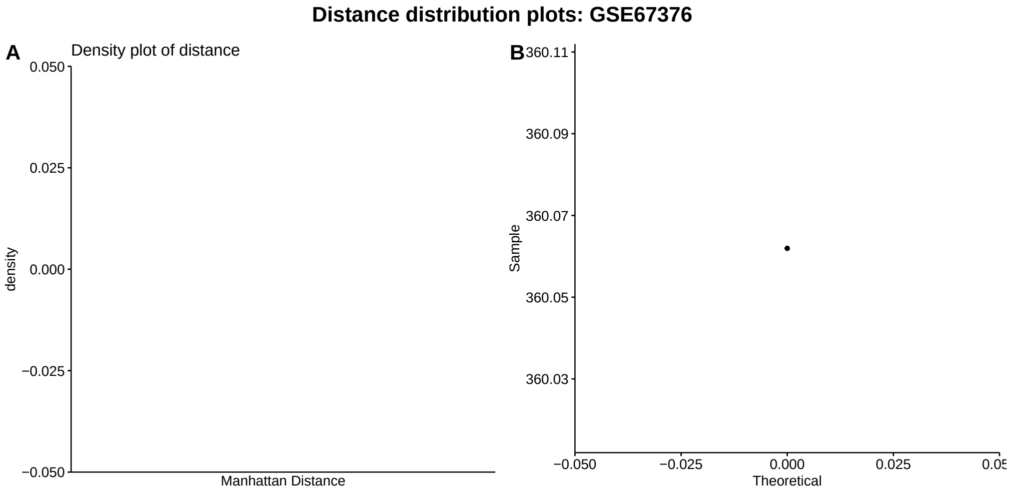

S104 Fig. Density and quantile-quantile plots for distances between samples in GSE67376. **A** Estimated density curve for distances. **B** Quantile-quantile plot between theoretical (standard normal) quantiles and sample distance quantiles.

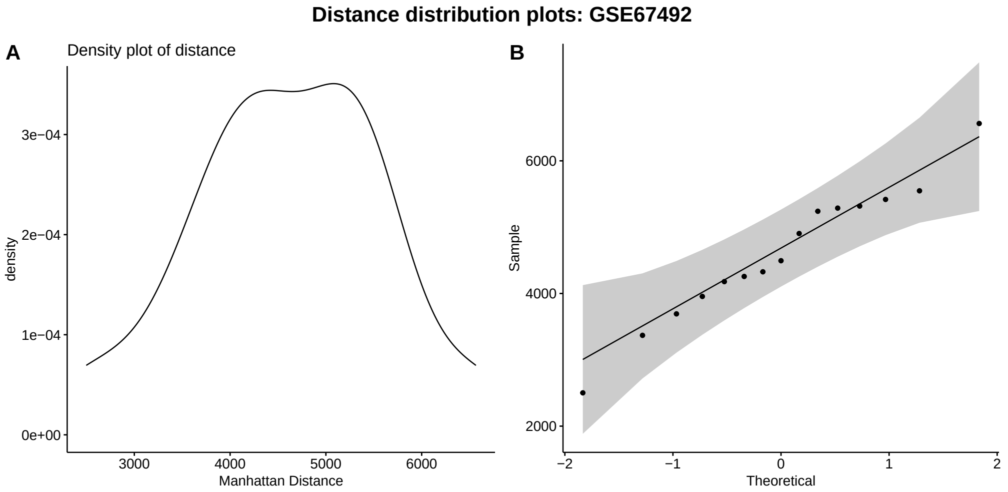

S105 Fig. Density and quantile-quantile plots for distances between samples in GSE67492. **A** Estimated density curve for distances. **B** Quantile-quantile plot between theoretical (standard normal) quantiles and sample distance quantiles.

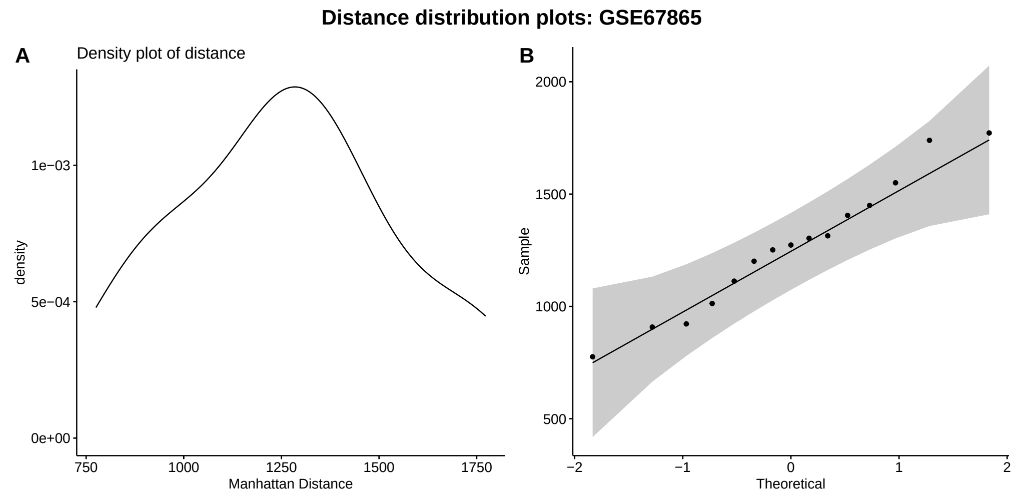

S106 Fig. Density and quantile-quantile plots for distances between samples in GSE67865. **A** Estimated density curve for distances. **B** Quantile-quantile plot between theoretical (standard normal) quantiles and sample distance quantiles.

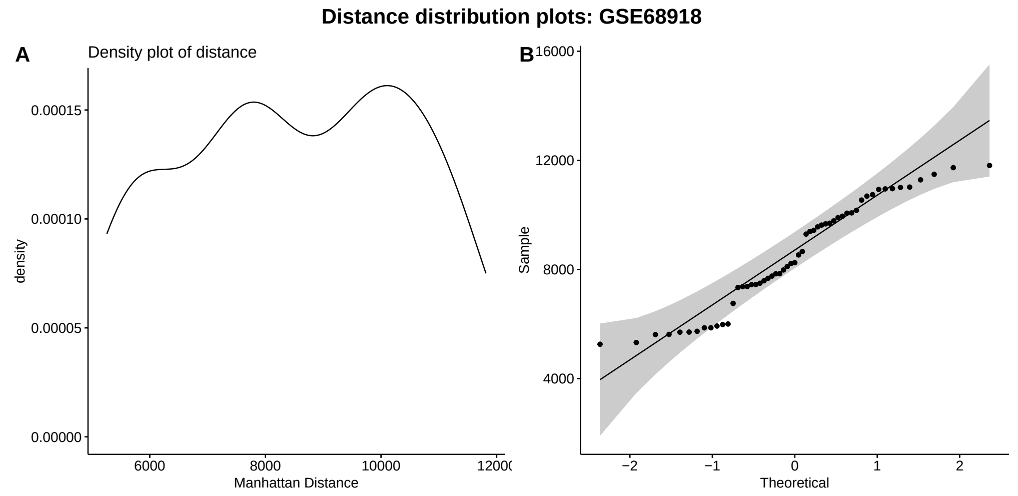

S107 Fig. Density and quantile-quantile plots for distances between samples in GSE68918. **A** Estimated density curve for distances. **B** Quantile-quantile plot between theoretical (standard normal) quantiles and sample distance quantiles.

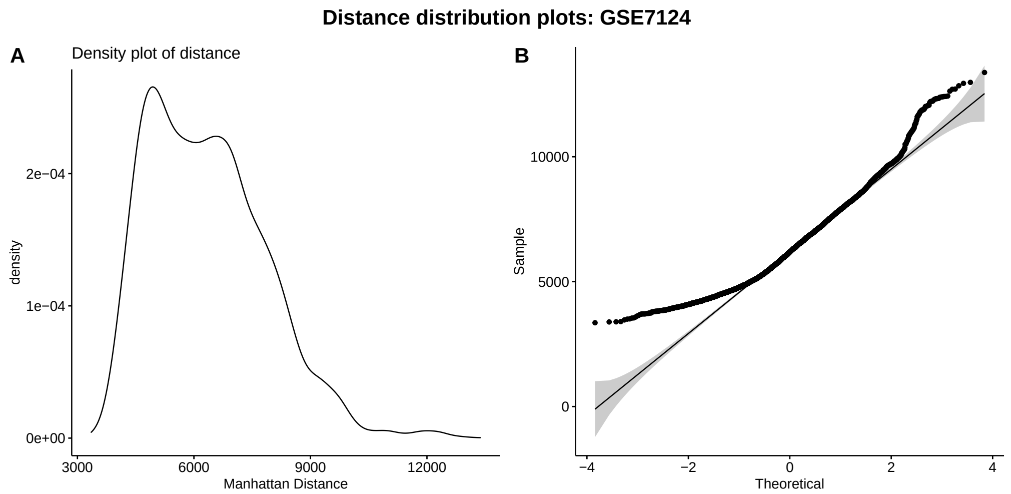

S108 Fig. Density and quantile-quantile plots for distances between samples in GSE7124. **A** Estimated density curve for distances. **B** Quantile-quantile plot between theoretical (standard normal) quantiles and sample distance quantiles.

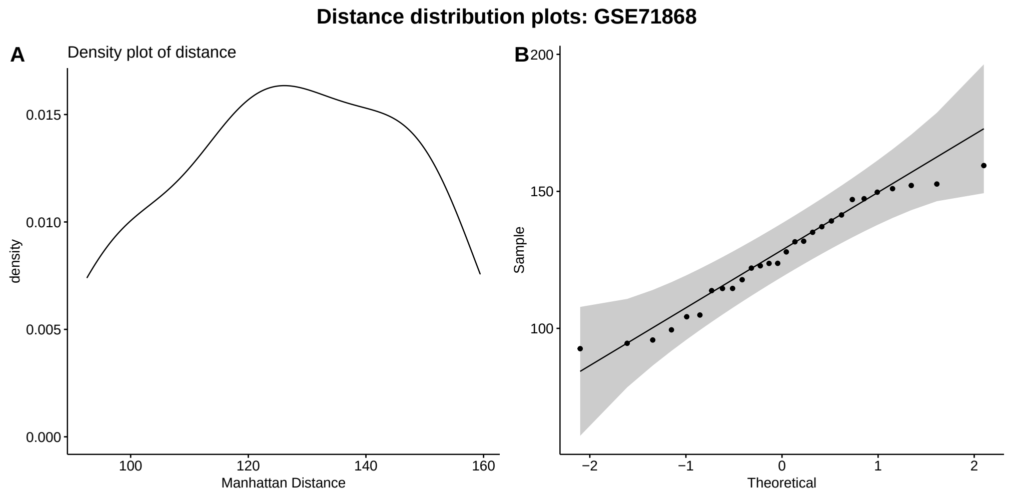

S109 Fig. Density and quantile-quantile plots for distances between samples in GSE71868. **A** Estimated density curve for distances. **B** Quantile-quantile plot between theoretical (standard normal) quantiles and sample distance quantiles.

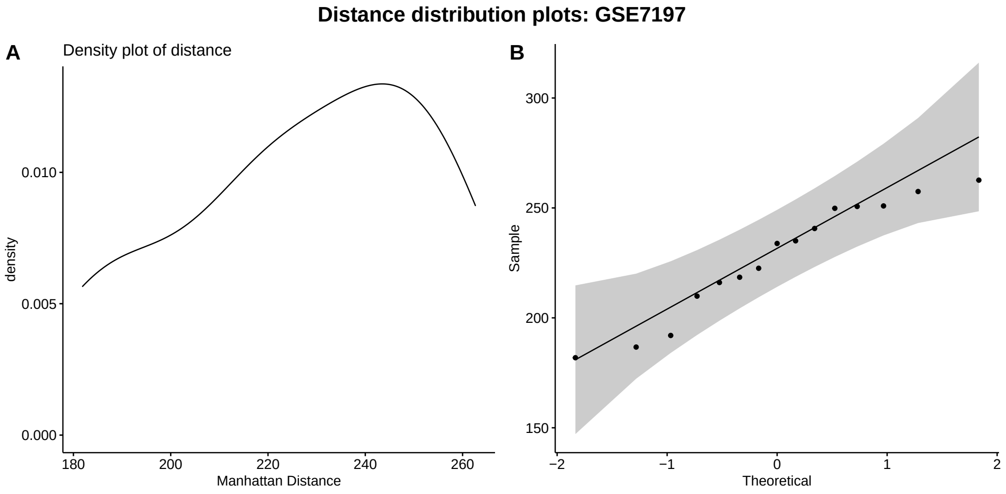

S110 Fig. Density and quantile-quantile plots for distances between samples in GSE7197. **A** Estimated density curve for distances. **B** Quantile-quantile plot between theoretical (standard normal) quantiles and sample distance quantiles.

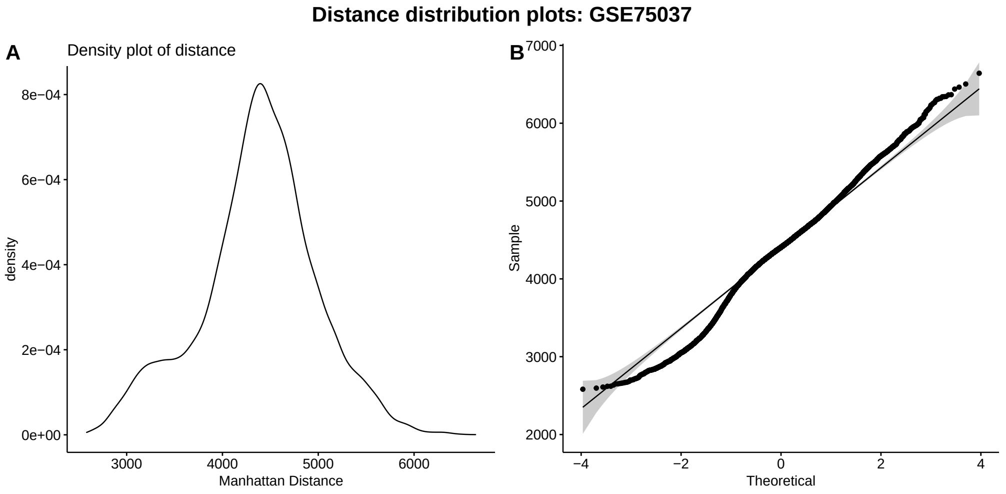

S111 Fig. Density and quantile-quantile plots for distances between samples in GSE75037. **A** Estimated density curve for distances. **B** Quantile-quantile plot between theoretical (standard normal) quantiles and sample distance quantiles.

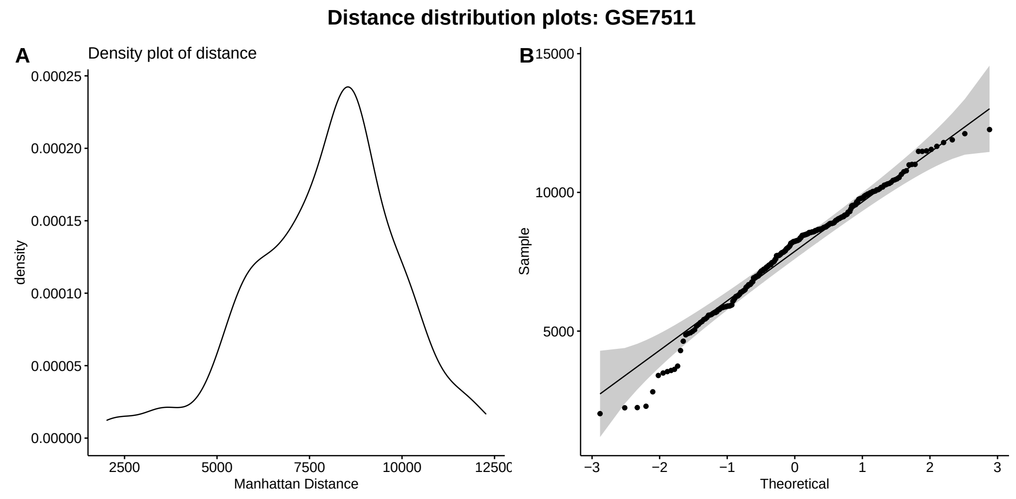

S112 Fig. Density and quantile-quantile plots for distances between samples in GSE7511. **A** Estimated density curve for distances. **B** Quantile-quantile plot between theoretical (standard normal) quantiles and sample distance quantiles.

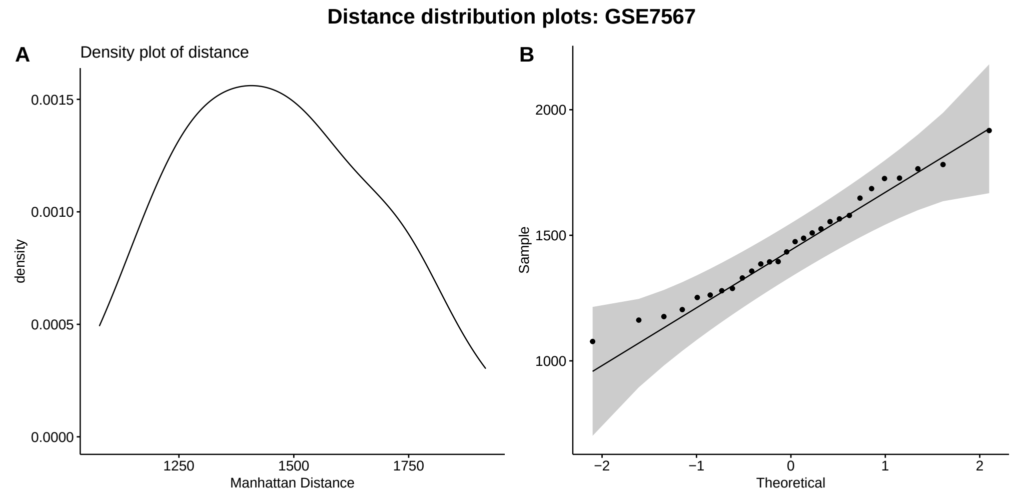

S113 Fig. Density and quantile-quantile plots for distances between samples in GSE7567. **A** Estimated density curve for distances. **B** Quantile-quantile plot between theoretical (standard normal) quantiles and sample distance quantiles.

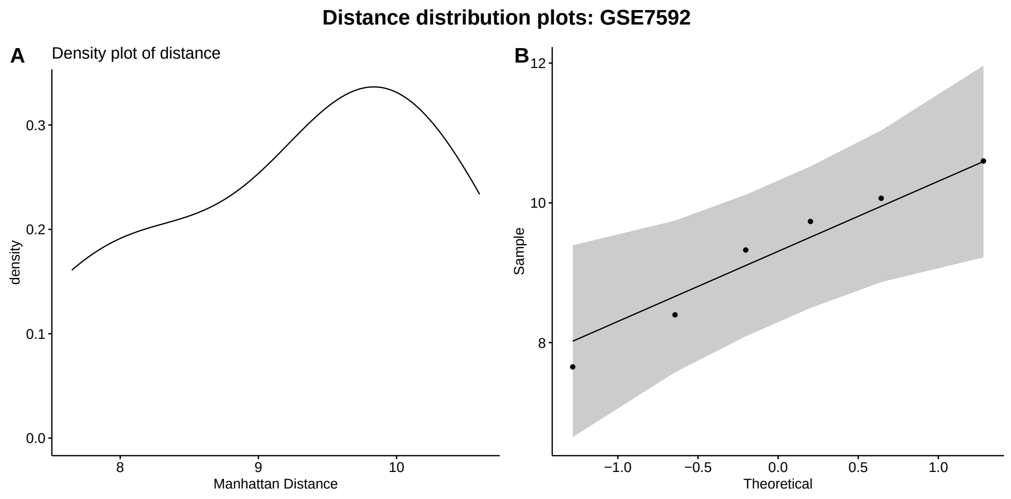

S114 Fig. Density and quantile-quantile plots for distances between samples in GSE7592. **A** Estimated density curve for distances. **B** Quantile-quantile plot between theoretical (standard normal) quantiles and sample distance quantiles.

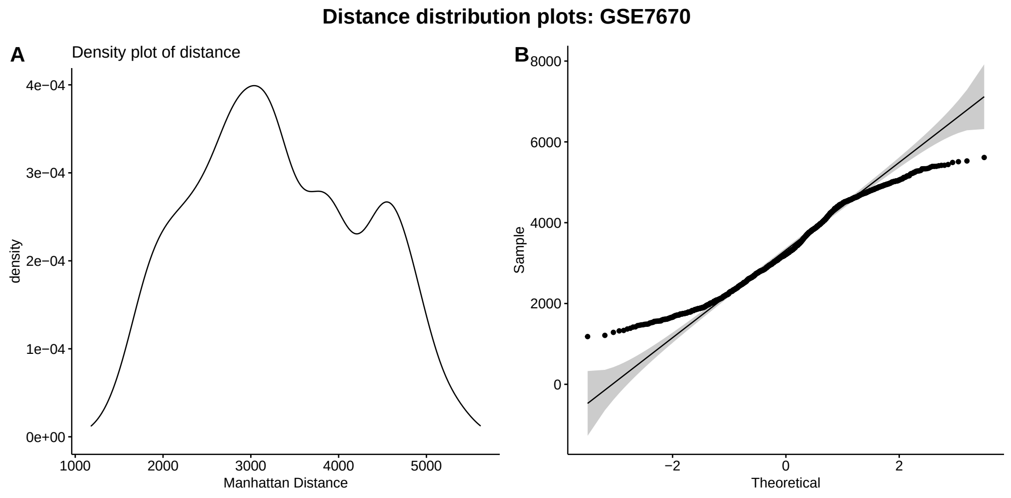

S115 Fig. Density and quantile-quantile plots for distances between samples in GSE7670. **A** Estimated density curve for distances. **B** Quantile-quantile plot between theoretical (standard normal) quantiles and sample distance quantiles.

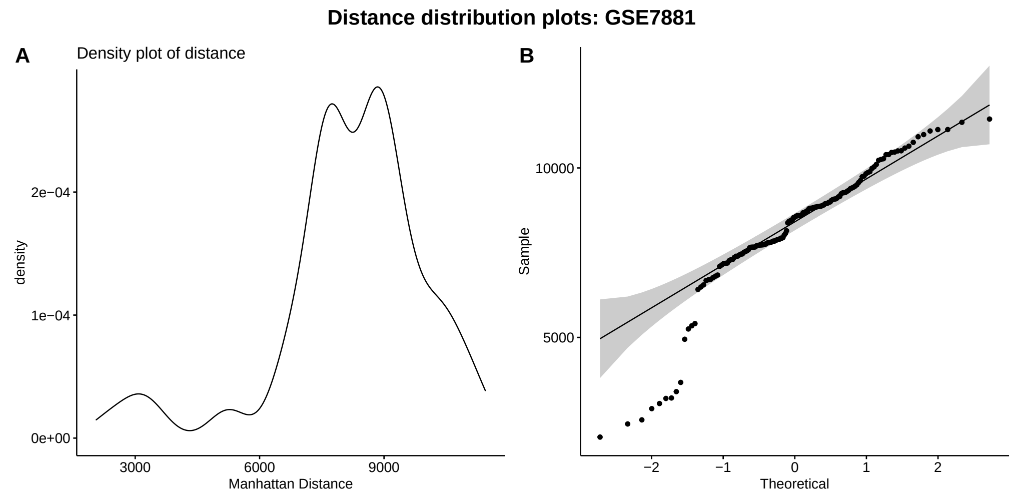

S116 Fig. Density and quantile-quantile plots for distances between samples in GSE7881. **A** Estimated density curve for distances. **B** Quantile-quantile plot between theoretical (standard normal) quantiles and sample distance quantiles.

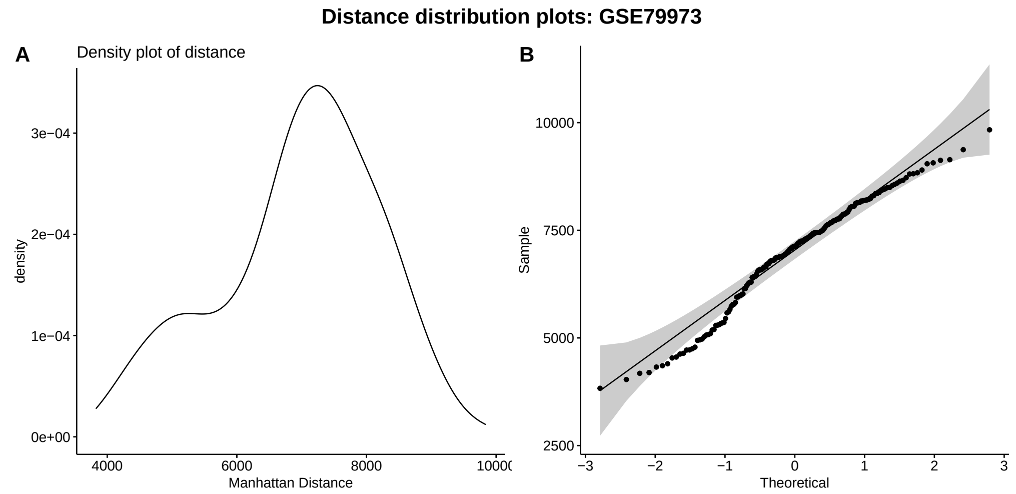

S117 Fig. Density and quantile-quantile plots for distances between samples in GSE79973. **A** Estimated density curve for distances. **B** Quantile-quantile plot between theoretical (standard normal) quantiles and sample distance quantiles.

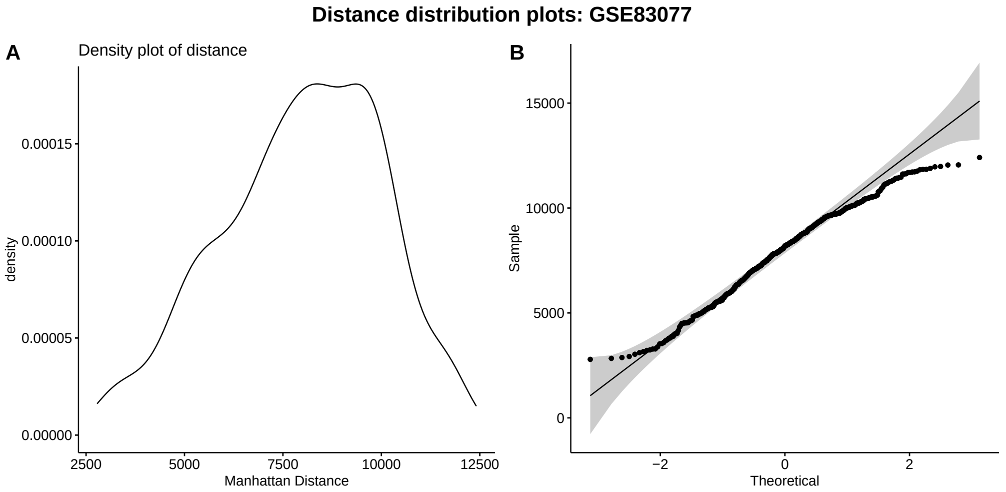

S118 Fig. Density and quantile-quantile plots for distances between samples in GSE83077. **A** Estimated density curve for distances. **B** Quantile-quantile plot between theoretical (standard normal) quantiles and sample distance quantiles.

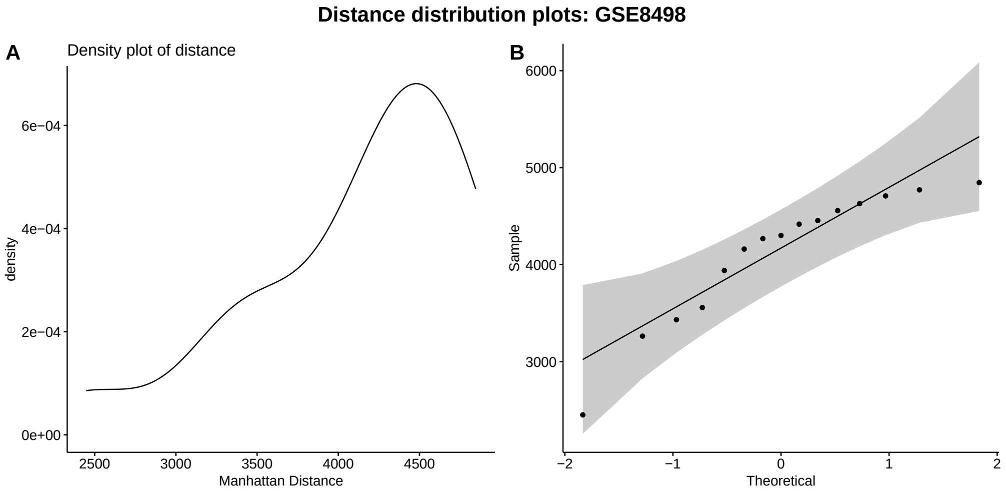

S119 Fig. Density and quantile-quantile plots for distances between samples in GSE8498. **A** Estimated density curve for distances. **B** Quantile-quantile plot between theoretical (standard normal) quantiles and sample distance quantiles.

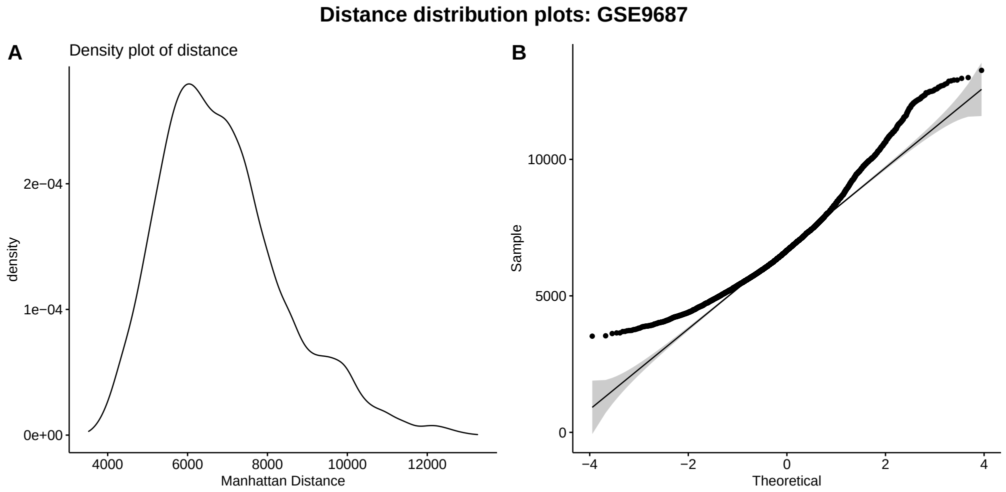

S120 Fig. Density and quantile-quantile plots for distances between samples in GSE9687. **A** Estimated density curve for distances. **B** Quantile-quantile plot between theoretical (standard normal) quantiles and sample distance quantiles.

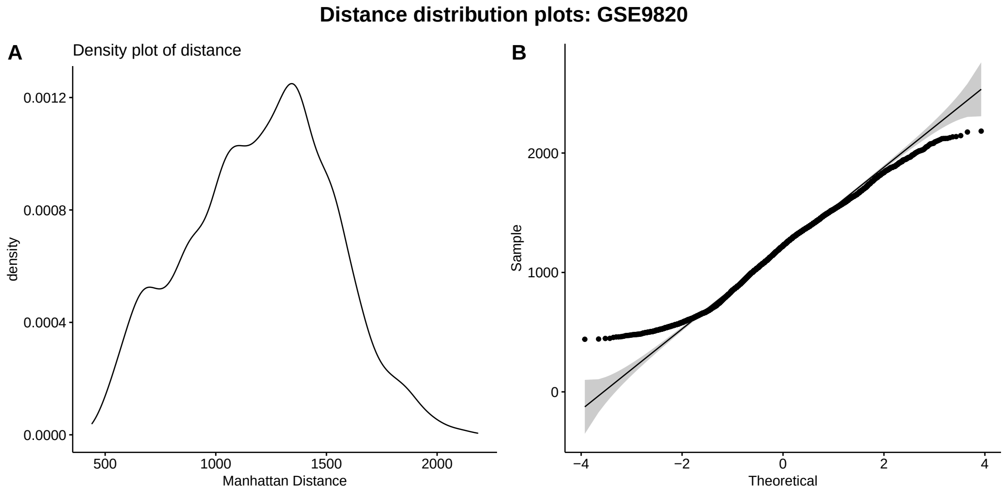

S121 Fig. Density and quantile-quantile plots for distances between samples in GSE9820. **A** Estimated density curve for distances. **B** Quantile-quantile plot between theoretical (standard normal) quantiles and sample distance quantiles.

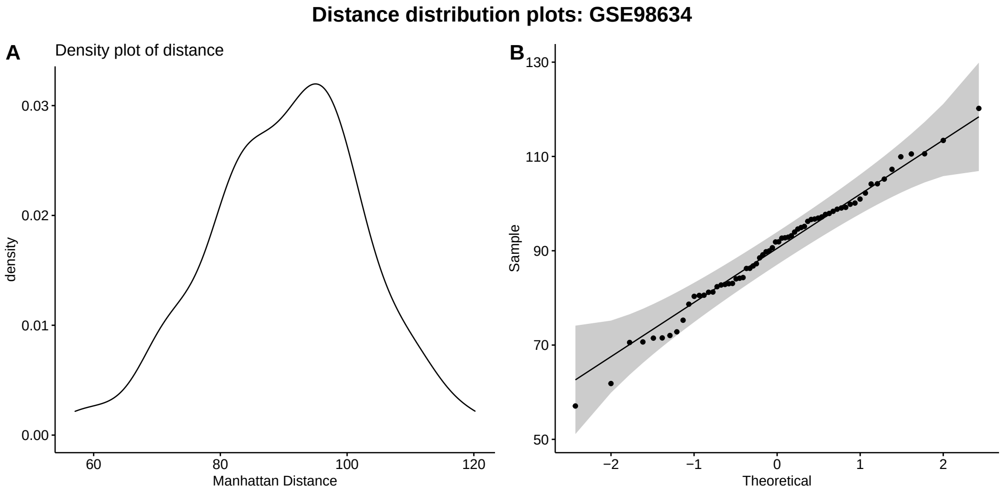

S122 Fig. Density and quantile-quantile plots for distances between samples in GSE98634. **A** Estimated density curve for distances. **B** Quantile-quantile plot between theoretical (standard normal) quantiles and sample distance quantiles.

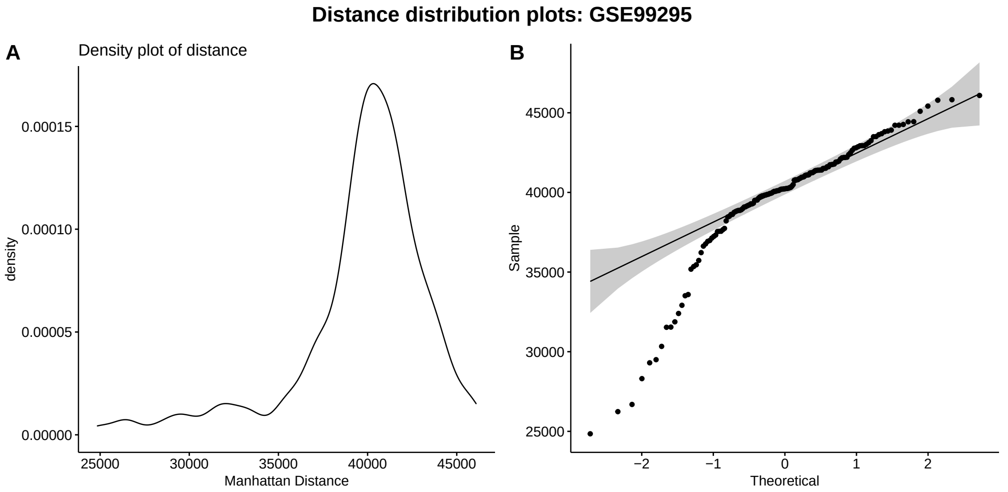

S123 Fig. Density and quantile-quantile plots for distances between samples in GSE99295. **A** Estimated density curve for distances. **B** Quantile-quantile plot between theoretical (standard normal) quantiles and sample distance quantiles.

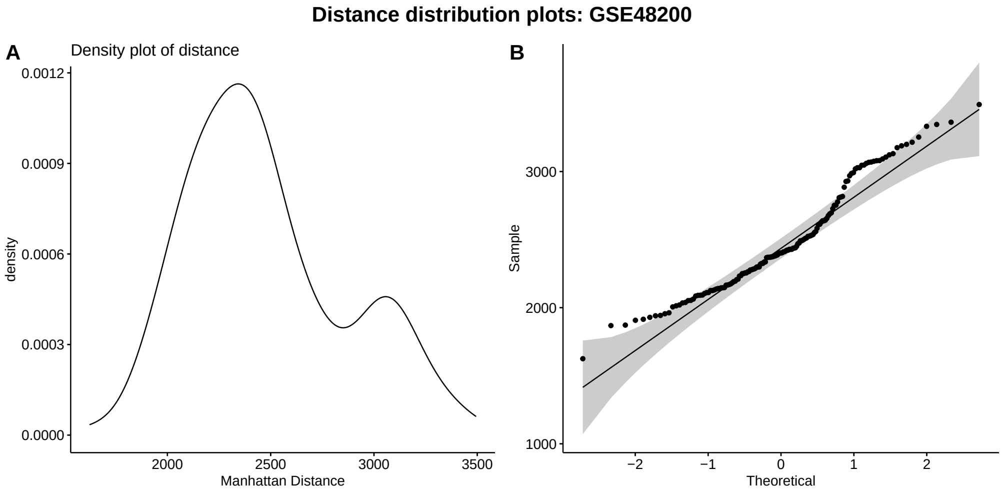

S124 Fig. Density and quantile-quantile plots for distances between samples in GSE48200. **A** Estimated density curve for distances. **B** Quantile-quantile plot between theoretical (standard normal) quantiles and sample distance quantiles.

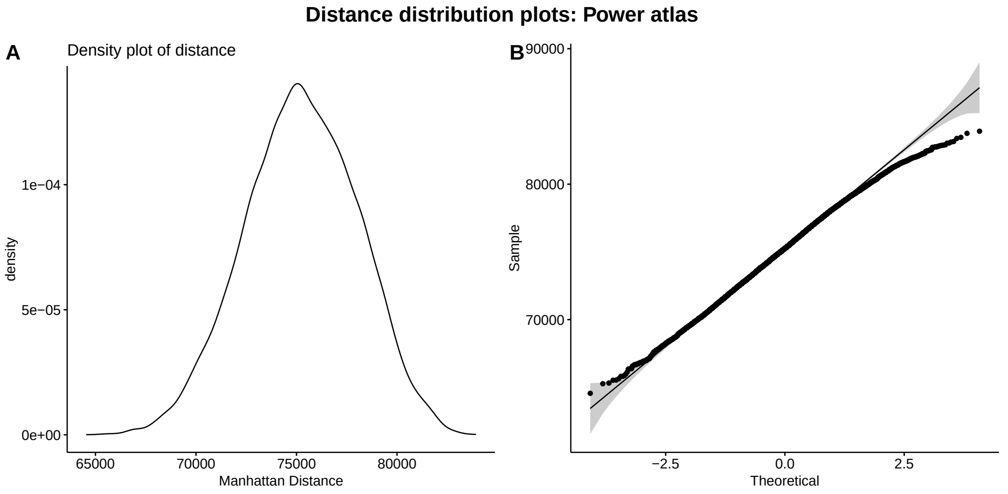

S125 Fig. Density and quantile-quantile plots for distances between samples in real resting-state fMRI data generated using a parcellation of spherical ROIs [1]. **A** Estimated density curve for distances. **B** Quantile-quantile plot between theoretical (standard normal) quantiles and sample distance quantiles.

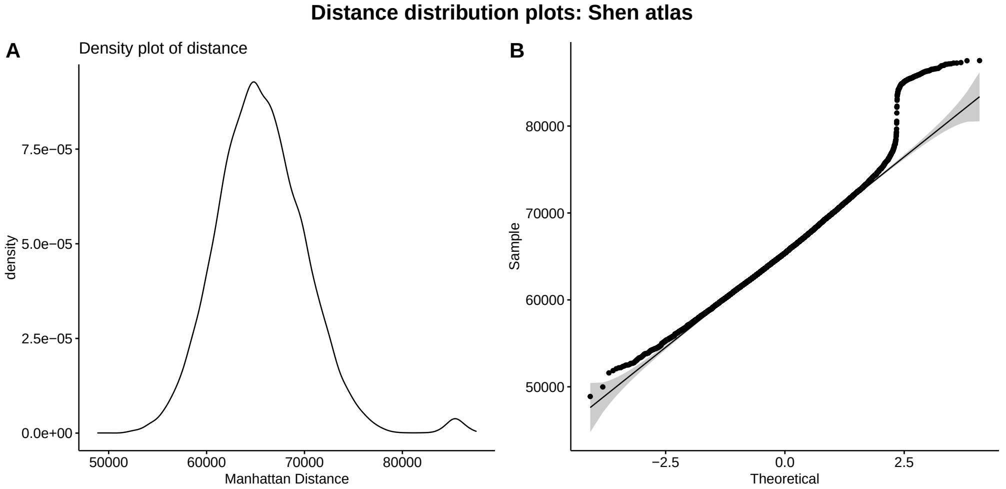

S126 Fig. Density and quantile-quantile plots for distances between samples in real resting-state fMRI data generated using a graph theoretic parcellation of ROIs [2]. **A** Estimated density curve for distances. **B** Quantile-quantile plot between theoretical (standard normal) quantiles and sample distance quantiles.

References

1. Jonathan D Power, Alexander L Cohen, Stephen M Nelson, Gagan S Wig, Kelly Anne Barnes, Jessica A Church, Alecia C Vogel, Timothy O Laumann, Fran M Miezin, Bradley L Schlaggar, and Steven E Peterson. Functional network organization of the human brain. *Neuron*, 72(4):665–678, November 2011.

2. X. Shen, F. Tokoglu, X. Papademetris, and R. T. Constable. Groupwise whole-brain parcellation from resting-state fMRI data for network node identification. *Neuroimage*, (0):403–415, November 2013.
